# Circ0085539 Promotes Osteosarcoma Progression by Suppressing miR-526b-5p and PHLDA1 Axis

**DOI:** 10.3389/fonc.2020.01250

**Published:** 2020-08-26

**Authors:** Pengcheng Liu, Wei Liu, Hang Gao, Yuanding Zhang, Ming Yan, Xu Wang

**Affiliations:** ^1^Department of Hand and Foot Surgery, The First Hospital of Jilin University, Changchun City, China; ^2^Department of Spine Surgery, The First Hospital of Jilin University, Changchun City, China; ^3^Department of Bone and Joint Surgery, The First Hospital of Jilin University, Changchun City, China; ^4^Department of Otolaryngology Head and Neck Surgery, The First Hospital of Jilin University, Changchun City, China; ^5^Department of Colorectal and Anal Surgery, The First Hospital of Jilin University, Changchun City, China

**Keywords:** osteosarcoma, oncogene, circ0085539, miR-526b-5p, PHLDA1

## Abstract

**Background:** We have previously found that circ0085539/miR-526b-5p axis participated in the progression of osteosarcoma (OS). We have been interested in expanding the networking involving circ0085539 and miR-526-5p. We identified another critical downstream target of this axis, pleckstrin homology-like domain family A member 1 (PHLDA1), thus intending to uncover the interaction between the axis and PHLDA1.

**Methods:** Live imaging of mice tumor xenografts was conducted. Immunohistochemistry (IHC) and H&E staining were performed for our *in vivo* experiment, while the CCK-8 assay, flow cytometry, wound healing, Transwell invasion, and clone formation were employed to assess cellular biological functions.

**Results:** Circ0085539 was first found to be upregulated in osteosarcoma tissues and cell lines, and circ0085539 knockdown obviously suppressed proliferation and induced apoptosis. Subsequently, miR-526b-5p functionally attenuated the tumor suppressive effects induced by circ0106714 silencing on OS cells. PHLDA1 silencing significantly led to proliferation suppression, apoptosis induction, as well as the inhibition of migration, invasion, and colony formation capabilities in OS cells, which also could be restored by the miR-526b-5p inhibitor.

**Conclusion:** Taken together, circ0085539 effectively promoted progression of osteosarcoma through sponging miR-526b-5p to release PHLDA1, strongly suggesting that *in vivo* intervention of circ0085539–miR-526b-5p–PHLDA1 axis could function as a promising OS-targeted therapy.

## Introduction

Osteosarcoma (OS), derived from mesenchymal cells, has been reported to basically have its onset in adolescents (1). The quality of life in adolescents is severely compromised by OS due to the disruption of bone development (2). Although a plenty of advanced surgical techniques have been applied for clinical practice of OS, the problem of its bloodcurdling fatality rate and unsatisfactory survival rate has not been effectively improved (3). There are still many patients with OS suffering from its recurrence on account of distant metastasis (4). Additionally, the pathogenesis of OS remains elusive. Thus, a novel gene regulation axis is urgently needed to be unearthed for diagnosis and treatment of OS.

With the extensive application of sequencing technologies, increasing potential biological function of unknown transcripts in cells has been determined. Noncoding RNAs (ncRNAs), which is abundant in cells, cannot participate in the translation process to form corresponding protein (5, 6). Circular RNA (circRNA) with closed loop structures is a member of ncRNAs (7). Based on their origins, circRNAs are divided into three types: the exonic circRNAs, which are typically located in the cytoplasm; the intronic circRNAs, which are mainly located in the cell nuclei; and the intergenic circRNAs (8). Owing to their abundance and stability, the exonic circRNAs have become the most frequently studied circRNAs.

Emerging evidence revealed that circRNAs could act as the sponge of microRNAs (miRNAs) in the cytoplasm to inhibit miRNA functions on account of possession of miRNA binding sites (7, 9). Besides, several circRNAs can participate in nuclear transcription (9, 10) and serve as sequestering agents of other proteins that constitute some crucial signaling pathways (11, 12). Moreover, multiple circRNAs have been identified as oncogenes or malignancy suppressors due to their regulations on gene expression and cell activities, such as proliferation, apoptosis, cell cycle, migration, invasion, and colony formation (13–15). In various cancers, circRNAs regulates tumorigenesis by sponging miRNAs to release messenger RNAs (mRNAs). For instance, circ-NSD2 induces migration and invasion of colorectal cancer cells through sponging miR-199b-5p, which positively regulates DDR1 and JAG1 (16). CircEPSTI1 promotes ovarian cancer progression through inhibiting miR942 to release EPSTI1 (17). Circular RNA_LARP4 suppresses the metastasis of gastric cancer cells through sponging miR-424-5p to target LATS1 (18). Circ0085539, another name for circPVT1, had proved to be overexpressed and promoted the metastasis in OS (19, 20). Previously, we reported that one of the PVT1-encoded circular RNAs could interact with miR-526b-5p and regulate a downstream gene FOXC2 to affect OS progression (21). We found that circ0085539 is one of the PVT1-encoded circular RNAs and is predicted to sponge miR-526b-5p. Whether circ0085539 regulates miR-526b-5p, therefore affecting OS progression, arouse our interest. In addition, we intended to find a novel downstream target of miR-526b-5p to enhance our comprehension of OS progression. The novelty of this study would be that a novel circular RNA encoded by PVT1 and a novel downstream target of miR-526b-5p were found to act through a competing endogenous RNA network to regulate the progression of OS.

We found that since 2014, there have been approximately 10 studies related to the role of miR-526b in cancer, and 2 of them reported the downregulation of miR-526b-5p in oral squamous cell carcinoma and esophageal squamous cell carcinoma as well as its tumor suppressive effect, respectively. The expression and role of miR-526b-5p in other cancers have not been explored. Our research will lead the way in studying the role of miR-526-5p in osteosarcoma and enrich the research results of miRNA in osteosarcoma.

Pleckstrin homology-like domain family A member 1 (PHLDA1) could encode a pleckstrin homolog (PHL) domain that participates in intracellular signaling and constitutes the cytoskeleton of cells (22). PHLDA1 has been reported to be expressed in numerous cancers and has different functions in different cancers, most of which are correlated with cell proliferation and apoptosis (23, 24). Whether PHLDA1 participates in cell proliferation and apoptosis regulation was intended to be investigated in our study.

In this paper, circ0085539 was identified to be upregulated in osteosarcoma tissues and cell lines. Notably, miR-526b-5p inhibitor not only facilitated cell proliferation, decreased cell apoptosis, as well as promoted the abilities of migration, invasion, and colony formation but also abrogated the tumor suppressive effect induced by circ0085539 and PHLDA1 silencing in OS cells. Collectively, circ0085539 functionally promoted osteosarcoma progression by sponging miR-526b-5p to release PHLDA1. Circ0085539–miR-526b-5p–PHLDA1 axis could act as a novel target for therapy of osteosarcoma.

## Materials and Methods

### Clinical Data

Thirty-five OS tissue samples and 30 adjacent tissue samples were collected from patients who came visiting The First Hospital of Jilin University in the context that all participants signed the written informed content. Osteosarcoma was pathologically diagnosed by three independent physicians. No patients received any anticancer treatment before the tissue sample collection. The hospital Ethics Committee approved our study. All the tissue samples were stored in liquid nitrogen until research experiments. Table 1 shows the baseline characteristics of all the participants including histological types, differentiation status, tumor–node–metastasis (TNM) stages, etc.

**Table 1:** The baseline characteristics of all participants.

	**OS group** **(*n* = 15)**	**Control group** **(*n* = 10)**	***P*-values**
Age, years	23 ± 13.5	24 ± 13.25	0.592
Gender, male/female	9/6	6/4	>0.9999
smoking, *n* (%)	7 (46.7%)	4 (40%)	0.742
Alcoholic intake, *n* (%)	5 (33.3%)	3 (30%)	0.861
Body mass index kg/m^2^	24.8 ± 6.1	24.3 ± 7.0	0.574
Site			
Femur, *n* (%)	10 (66.6%)		
Tibia, *n* (%)	4 (26.7%)		
Other, *n* (%)	1 (6.7%)		
Histologic type			
Osteoblastic, *n* (%)	7 (46.7%)		
Chondroblastic, *n* (%)	5 (33.3%)		
Fibroblastic, *n* (%)	2 (13.3%)		
Others, *n* (%)	1 (6.7%)		
Differentiation status			
High, *n* (%)	11 (73.3%)		
Low, *n* (%)	4 (26.7%)		
TNM stage			
I	4 (26.7%)		
II	8 (53.3%)		
III	3 (20%)		

### Cell Culture

Immortalized human osteoblast cell line hFOB1.19 and osteosarcoma cell lines HOS, 143B, U2OS, SJSA1, and Saos2 were purchased from the Chinese Academy of Sciences Cell Bank (Shanghai, China). Dulbecco's modified Eagle's medium (DMEM)/F12 (Gibco BRL, Grand Island, NY, USA) supplemented with 10% fetal bovine serum (FBS) (Gibco, Carlsbad, CA) was applied for cell culture, and the cell culture atmosphere was 37°C and 5% CO_2_.

### Vectors Construction and Cell Transfection

The circ0085539 short hairpin RNA (shRNA) lentiviral vectors (sh-circ0085539) and shRNA lentiviral vectors (sh-PHLDA1) were constructed by GeneChem (Shanghai, China). miR-526b-5p inhibitors were purchased from RiboBio (Guangzhou, China). The corresponding negative control (NC) was respectively, provided by the two companies. Briefly, the shRNAs were synthesized and inserted into the *Eco*RI and *Xho*I sites of pLV-CMV-puro-U6 lentiviral vector. The sequences of all shRNAs and inhibitor are shown in Supplementary Table 1.

For cell transfection, 20 MOI sh-circ0085539 and sh-PHLDA1 lentiviral vectors were added into HOS and U2OS cell culture to silence circ0085539 and PHLDA1, respectively. Since pLV-CMV-puro-U6 contains a puromycin gene, the puromycin was used to screen out the transfected OS cells that stably knocked down circ0085539 and PHLDA1. On the other hand, 40 nM miR-526b-5p inhibitor was transfected into the HOS and U2OS cells using Lipofectamine 3000 reagent (Invitrogen, Carlsbad, CA, USA) to inhibit miR-526b-5p. The transfection efficiency was measured by quantitative reverse transcription PCR (qRT-PCR) after a 48-h transfection.

### RNase R Digestion, qRT-PCR, and Western Blot Analysis

RNase R digestion experiment was carried out to confirm the stability feature of circ0085539. Briefly, RNase R (Epicentre Technologies, Madison, WI, USA) was used to digest RNA samples (5 μg) at 37°C for 15 min. Then, qRT-PCR was conducted to measure the relative expression of circ0085539 and PVT1 linear RNA.

For qRT-PCR, total RNA extracted by TRIzol (Invitrogen, CA, USA) was first reverse transcribed into complementary DNA (cDNA), and the following amplification was carried out using M-MLV and SYBR Green Master Mix kit (Guangzhou, China). In terms of miR-526b-5p expression, a stem-loop primer SYBR Green qRT-PCR kit (RiboBio, Co., Ltd., Guangzhou, China) was used. The primers are listed in Table 2. The 2^−ΔΔCt^ method was employed to calculate the relative expression. U6 was used to be the reference gene for miR-526b-5p, and glyceraldehyde 3-phosphate dehydrogenase (GAPDH) was used to be the reference gene for circ0085539 and PHLDA1.

**Table 2:** Overview of the sequences of all primers used in this study.

**QRT-PCR primers**
Circ0085539_ For: TATCGGAAGCTGCACATGGA
Circ0085539_Rev: GTCATGTAACACGGCCCTTG
PVT1_convergent_For: GGGGAATAACGCTGGTGGAA
PVT1_convergent_Rev: CCCATGGACATCCAAGCTGT
MiR-526b-5p_For: GTCTCTTGAGGGAAGCACT
miR-526b-5p_Rev: GTGCAGGGTCCGAGGT
PHLDA1 _ For: TGCCTGAAAGGGGCAGCTCC
PHLDA1 _ Rev: TGATCTGGTGCGGGGCGGA
GAPDH_ For: TCGGAGTCAACGGATTTGGT
GAPDH_Rev: TGGAATTTGCCATGGGTGGAA
U6_For: CGCTTCGGCAGCACATATACTA
U6_Rev: CGCTTCACGAATTTGCGTGTCA

For Western blot assay, HOS and U2OS cells were harvested to extract proteins by lysis buffer (Thermo Scientific, Rockford, IL, USA). After measuring the concentration, proteins were first separated by electrophoresis, then transferred to a polyvinylidene fluoride (PVDF) membrane, and incubated with primary antibodies against PHLDA1 (Cat#: ab133654, Abcam, UK, 1:10,000) and GAPDH (Cat#: ab181602, 1:10,000). After the incubation with primary antibodies, the membrane continued to incubate with the secondary antibody (Cat#: ab205718, 1:10,000). Eventually, the protein band was enhanced using a chemiluminescent kit (Thermo Fisher Scientific, USA), and the band intensity was read in Image J Software (Bio-Rad Laboratories, San Diego, CA, USA).

### Luciferase Reporter Assay and RNA Immunoprecipitation Assay

For the luciferase reporter assay that validated the regulatory association between circ0085539 and miR-526b-5p, the reporter plasmids, psiCHECK2-circ0085539-Mut and psiCHECK2-circ0085539-Wt, were constructed. For the luciferase reporter assay that validated the regulatory association between PHLDA1 and miR-526b-5p, the reporter plasmids, psiCHECK2-PHLDA1-Mut1, psiCHECK2-PHLDA1-Mut2, psiCHECK2-PHLDA1-Mut3, psiCHECK2-PHLDA1-co-Mut, and psiCHECK2-PHLDA1-Wt were constructed. All reporter plasmids were constructed with psiCHECK2 (Promega, Madison, WI, USA). The constructed reporter plasmids were transfected into HOS and U2OS cells in 96-well plates together with miR-526b-5p mimic or miR-NC for 48 h. Then luciferase activities were determined using a firefly-renilla assay system according to the manufacturer's instruction. The ratio of firefly and renilla luciferase was calculated.

RNA immunoprecipitation (RIP) assay was performed in HOS and U2OS cells using the Magna RIP RNA-Binding Protein Immunoprecipitation Kit. HOS and U2OS cells were transfected with miR-526b-5p mimic or miR-526b-5p NC. Cell suspension was centrifuged at 300 g for 5 min at 4°C for several rounds to pellet the cells. Five hundred microliters RIP lysis buffer combined with a protease inhibitor cocktail was added to the cells and vortexed. Lastly, the cell suspension was centrifuged at 12,000 g for 5 min, and the supernatant was incubated with Ago2 antibody- or rabbit immunoglobulin G (IgG)-coated beads at rotation for 60 min. The RNA was isolated after the antibody-immobilized beads were washed by centrifugation at 2,000 g for several times. Finally, the abundance of circ0085539 in extracted RNAs was analyzed by qRT-PCR. The kit and reagents were all purchased from Millipore.

### CCK-8 Cell Viability Assay and Flow Cytometry Apoptosis Assay

CCK-8, a very convenient assay with little harm to living cells, was used to detect the cell viability in this study. The transfected cells were cultured in a 96-well plate for 24, 48, and 72 h, respectively. Thereafter, 10 μl of the CCK-8 solution (Donjindo, Japan) was added to each well of the plate for 2 h. The optical absorbance at 450 nm was determined using an automatic microplate reader (ELx800, BioTek Instruments, USA).

The apoptosis assay was determined by flow cytometry. Approximately 1–5 × 10^5^ transfected cells were collected by centrifugation. Cells were washed with 1× phosphate-buffered saline (PBS), and the supernatant was removed. Finally, ~10,000 cells went through flow cytometric analysis. Five microliters Annexin-V-PE and PI solution (KeyGen Biotech) was added to the tubes and mixed by gentle swirling for 20 min in dark. Five hundred microliters 1× binding buffer was subsequently added to each tube. A flow cytometer (Beckman Coulter, Fullerton, CA, USA) was lastly used to analyze the apoptosis conditions of cells in every group. The sum of two right quadrants (Annexin V^+^/PI^+^ + Annexin V^+^/PI^−^) represented the cell apoptosis rate.

### Wound Healing Assay, Invasion Assay, and Colony Formation Assay

The migration ability of cells in this study was determined using wound-healing assay according to the lab protocol. Briefly, 1 × 10^5^ cells/ml transfected cells were seeded in a six-well plate and cultured in DMEM medium with FBS until 100% confluence. Then, the cell monolayers in every well were scratched using sterile pipette tips. The cells were then cultured in DMEM medium without FBS for 24 h. The wound healing results were photographed using a Leica DMi8-M microscope (Germany) at 40× magnification. The ratio of the difference between the scratch width at 24 and 0 h and the scratch width at 0 h represented the cell migration rate.

For cell invasion assay, 8-μm chambers (Corning, Beijing, China) were used as the Transwell chambers. The transfected cells were cultured in FBS-free medium 24 h for starvation treatment. Then, 1 × 10^5^ cells/ml transfected cells were inoculated into the upper chamber that was precoated with Matrigel (1:20, BD Biosciences, USA) that covered the upside of the membrane overnight and added the FBS-free medium, while 10% FBS was inoculated into the lower chambers. Four percent paraformaldehyde and 0.1% crystal violet were applied to fix and stain the cells that invaded through Transwell chambers. Then, the invasive cells could be observed, photographed, and counted under the Leica DMi8-M microscope (Germany) at 100× magnification. The number of invading cells in each chamber was counted as the mean from the five randomly selected fields photographed by Leica DMi8-M microscope.

For colony formation assay, HOS and U2OS cells were transfected with lentiviral sh-circ0085539 and lentiviral sh-PHLDA1 to stably knock down circ0085539 and PHLDA1, respectively. Then, the 1 × 10^3^ cells/well transfected cells were incubated in six-well plates for 14 days with or without transfection of the miR-526b-5p inhibitor. The miR-526b-5p inhibitor was transfected at 48-h intervals to maintain the low expression of miR-526b-5p. The culture medium was changed every 3 days to supply enough nutrients for cell growth. Ninety-five percent ethanol and crystal violet (0.1%) were employed to fix and stain the visible colonies, which were counted and photographed under a microscope.

### *In vivo* Tumorigenesis Assay

The Ethics Committee gave permission to all the operations in our animal experiments. Before the tumorigenesis assay, the HOS cells were stably transfected with circ0085539 silence lentiviral vectors or negative control lentiviral vectors containing firefly luciferase gene. The nude mice (male, 4 weeks old, two groups, *n* = 6 per group) obtained from the Animal Center of Shanghai Jiaotong University were subcutaneously injected in the left (one site) and right armpits (one site) with 1 × 10^6^ cells/site stably transfected HOS cells resuspended in 100 μl Hank's balanced salt solution (HBSS) with Matrigel at a volume of 1:1. After 4 weeks, the nude mice were injected with 150 mg/kg D-luciferin potassium salts (Wuhan, China). Fifteen minutes later, the IVIS 200 bioluminescence imaging system and Living Image software (Caliper Life Sciences, Hopkinton, MA) were employed for the live imaging of mice. The luminescence represented the growth of osteosarcoma. Mice were killed ultimately, and the tumor tissues were collected for ki67 immunostaining and H&E staining. We observed the growth situation and the number of proliferative cells in osteosarcoma tissues under a microscope.

### Bioinformatics Analysis

GSE49003 data downloaded from GEO DataSets (https://www.ncbi.nlm.nih.gov/gds/?term=) were the mRNA expression profile. After analysis, a total of 33 upregulated differentially expressed genes (DEGs) were screened out by Limma 3.26.8 with *p* < 0.05 and log fold change (logFC) > 2. The ENCORI starBase, Tarbase 8.0, and TargetScan Human 7.2 software were used to predict the target genes of miR-526b-5p, and 14 overlapping target genes of the three software were identified. Then, only 1 of the 14 overlapping target genes of miR-526b-5p predicted by the three software showed up in the GSE49003-upregulated DEGs.

### Rescue Experiment

Rescue experiments were performed to study whether circ0085539 regulated OS by miR-526b-5p/PHLDA1 axis. circ0085539 shRNA, miR-526b-5p inhibitor, and PHLDA1 shRNA constructs were first transfected in HOS and U2OS cells to establish cell lines with circ0085539 knockdown, miR-526-5p inhibition, and PHLDA1 knockdown, respectively. In the “rescue” groups, cells were cotransfected with miR-526-5p inhibitor and circ00855398 shRNA or PHLDA1 shRNA. Cell functional experiments such as CCK8, flow cytometry apoptosis, wound healing migration, Transwell invasion, and clone formation were performed in transfected HOS and U2OS cells to study how the cell viability, apoptosis, migration, invasion, and proliferation were regulated by circ0085539 knockdown, miR-526-5p inhibition, and PHLDA1 knockdown. In addition, the results of the “rescue” groups can ensure that the observed changes of cell viability, apoptosis, migration, invasion, and proliferation were caused by the knockdown of the genes of interest.

### Gel (2%) Electrophoresis

A 2% agarose gel was prepared in the lab. Loading buffer was added to every DNA sample. A molecular weight ladder was carefully loaded to the first lane of the gel. The DNA samples were added to the additional lanes of the gel. The electrophoresis was run at 5 V/cm. The gel was then placed to 1× Tris–acetate–ethylenediaminetetraacetic acid (TAE) buffer for 30 min and in water for 5 min. Lastly, the DNA fragments were visualized under a UV light device (a Bio-Rad gel imager). Convergent and divergent primers were synthesized by GenePharma (Shanghai, China). They were used to amplify the circular and linear transcripts of PVT1 in both cDNA and genomic DNA (gDNA) from osteosarcoma and adjacent healthy tissues, respectively. Theoretically, the circular transcript of PVT1 could be only amplified by divergent primers in cDNA but not gDNA (extracted using DNeasy Blood & Tissue Kit from Qiagen). GAPDH was used as a negative control. The sequences of the convergent and divergent primers are given in Table 2.

### Immunohistochemistry Staining for PHLDA1 Detection in Tissue Samples

The tissues were formalin fixed and paraffin embedded prior to the immunohistochemistry (IHC) staining. They were then sliced into 5-μm in-width slides, which were then placed in 55°C for 15 min. The slides were then deparaffinized and rehydrated. Trypsin (0.1%) in PBS was used to achieve antigen retrieval. Three percent hydrogen peroxide (5 min treatment) was used to inactivate the endogenous peroxidase. The slides were then incubated with anti-PHLDA1 primaries (Cat#: ab133654) for 1 h at 1/100 in humidified chamber with 37°C condition. Subsequently, secondary antibodies (Cat#: ab205718) were applied at 1:5,000 for 0.5 h. Alkaline phosphatase substrate solution was added and incubated for 10 min. Lastly, hematoxylin was added to the slides for 5 min. The slides were then photographed using a light microscope.

### Statistical Analysis

The GraphPad PRISM Version 7.0.1 statistical program (San Diego, CA, USA) was employed to analyze our data, expressed as mean ± SD (standard deviation), and to output histograms and scatterplots. Statistical significance was determined by Student's *t*-test and one-way ANOVA followed by Dunnett's *post hoc* tests; *p* < 0.05 were regarded as statistically significant, which are marked with ^*^ or # in figures. Each experiment was performed at least three times independently.

## Result

### Characteristic Analysis of Circ0085539 in OS Cells

**Figure 1:**
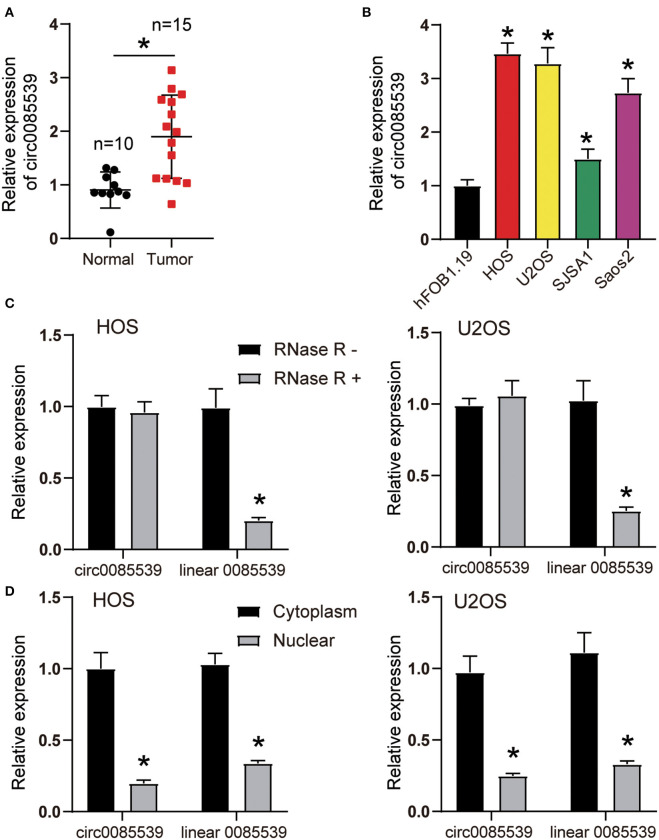
The expression of circ0085539 in human osteosarcoma tissues and the characterization of circ0085539 in human osteosarcoma cells. **(A)** The relative expression of circ0085539 in 15 human osteosarcoma and 10 adjacent tissues. Normal: adjacent tissues. *p < 0.05 vs. normal group. **(B)** The relative expression of circ0085539 in osteosarcoma cell lines (HOS, U2OS, SJSA1, Saos2) and the normal human osteoblast cell line (hFOB1.19) were detected by quantitative reverse transcription PCR (qRT-PCR). *p < 0.05 vs. hFOB1.19 cell line. **(C)** The qRT-PCR analysis confirmed that linear 0085539 could be easily digested by RNase R, while circ0085539 resisted to RNase R digestion. *p < 0.05 vs. RNase R group. **(D)** The qRT-PCR analysis showed that circ0085539 and linear 0085539 were predominately distributed in the cytoplasm of HOS and U2OS cells. *p < 0.05 vs. cytoplasm.

As shown in Supplementary Figure 4A, the existence of circ0085539 was validated in osteosarcoma and adjacent tissues using agarose gel electrophoresis. Divergent primers detected circ0085539 in cDNA but not in gDNA. Figure 1A shows that the relative expression of circ0085539 in OS tissues was twice that of adjacent tissues. In addition, another 20 pairs of osteosarcoma tissues and adjacent tissues were collected. Ten of these were randomly assigned to training dataset, while the other 10 were assigned to the validation dataset. In the two datasets, circ0085539 expression was detected. The use of training and validation datasets eliminated the false positives to a maximum in the detection of the upregulation of circ0085539 in osteosarcoma (Supplementary Figure 4B). The relative expression of circ0085539 in various OS cell lines (HOS, U2OS, SJSA1, and Saos2) was obviously higher than that in the normal human osteoblast cell line hFOB1.19 (Figure 1B). Among those OS cell lines, HOS and U2OS cells had the highest expression level of circ0085539, exceeding thrice that of the hFOB1.19 cell line. Thus, HOS and U2OS cells were selected for our further experiments. RNase R treatment was employed to verify the stability of circ0085539. circ0085539 resisted to the digestion of RNase R, while most of the linear 0085539 was digested in the two cell lines (Figure 1C). Subcellular localization results revealed that the majority of circ0085539 and linear 0085539 was localized in the cytoplasm of OS cells (Figure 1D).

### Silencing Circ0085539 Suppressed Xenografted Osteosarcoma Growth *in vivo*

**Figure 2:**
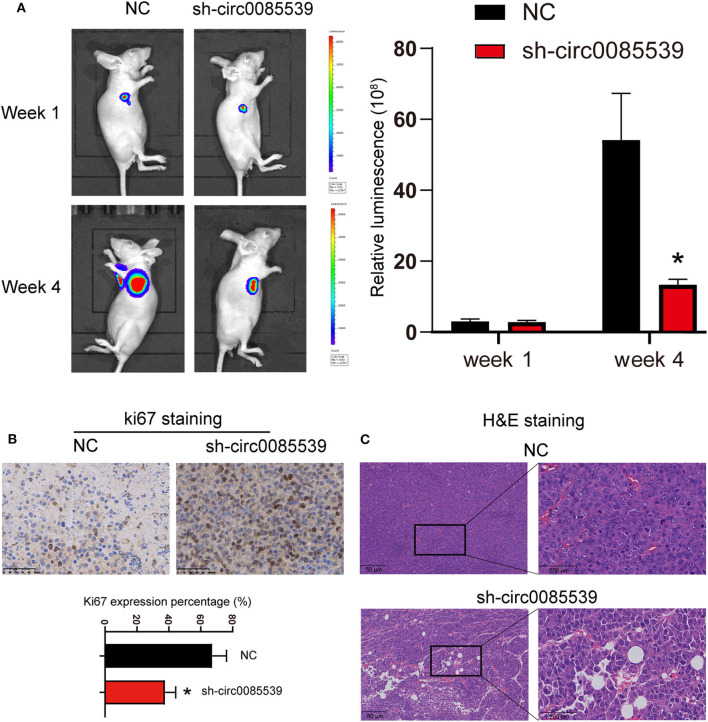
Silencing circ0085539 inhibited tumor growth *in vivo* (*n* = 6 each group). **(A)** Live imaging of the effects of circ0085539 knockdown on osteosarcoma growth *in vivo*. The luminescence was analyzed. **(B)** Ki67 staining and **(C)** H and E staining of xenografted tumor tissues with the knockdown of circ0085539 compared with the negative control (sh-NC) group. Scale bar for Ki67 IHC, 50 μm; scale bar for HandE, 50 μm (left panel) and 200 μm (right panel). *p < 0.05 vs. NC.

First, three circ0085539 shRNAs were respectively, transfected into HOS and U2OS cells. The transfection efficiency of circ0085539 shRNA-1 was more than 70% in both HOS and U2OS cells. shRNA-1 successfully targeted down circ0085539 rather than PVT1 mRNA (Supplementary Figure 1). To determine the function of circ0085539 in osteosarcoma, the nude mice were injected with HOS cells that were transfected with negative control or circ0085539 silencing vectors. At the beginning of the animal experiments, HOS cell line stably transfected with circ0085539 shRNA-1 (sh-circ0085539 from hereon) was established (Supplementary Figure 2A). Figure 2A shows that circ0085539 silencing significantly inhibited the growth of osteosarcoma *in vivo*. Besides, IHC results showed that the percentage of Ki67-positive cells in the sh-circ0085539 group was lower than half that of the NC group (Figure 2B). H&E staining results demonstrated the pathology of xenografted tumor tissues in sh-circ0085539 group compared with the NC group: loose arrangement and damaged texture (Figure 2C). Besides, the qRT-PCR proved that the circ0085539 expression was reduced in the xenografted tumor tissues after injection by transfected HOS cells (Supplementary Figure 2B). These results indicate that circ0085539 knockdown could effectively suppress proliferation of OS *in vivo*.

### Circ0085539 Functioned as a miR-526b-5p Sponge in OS

**Figure 3:**
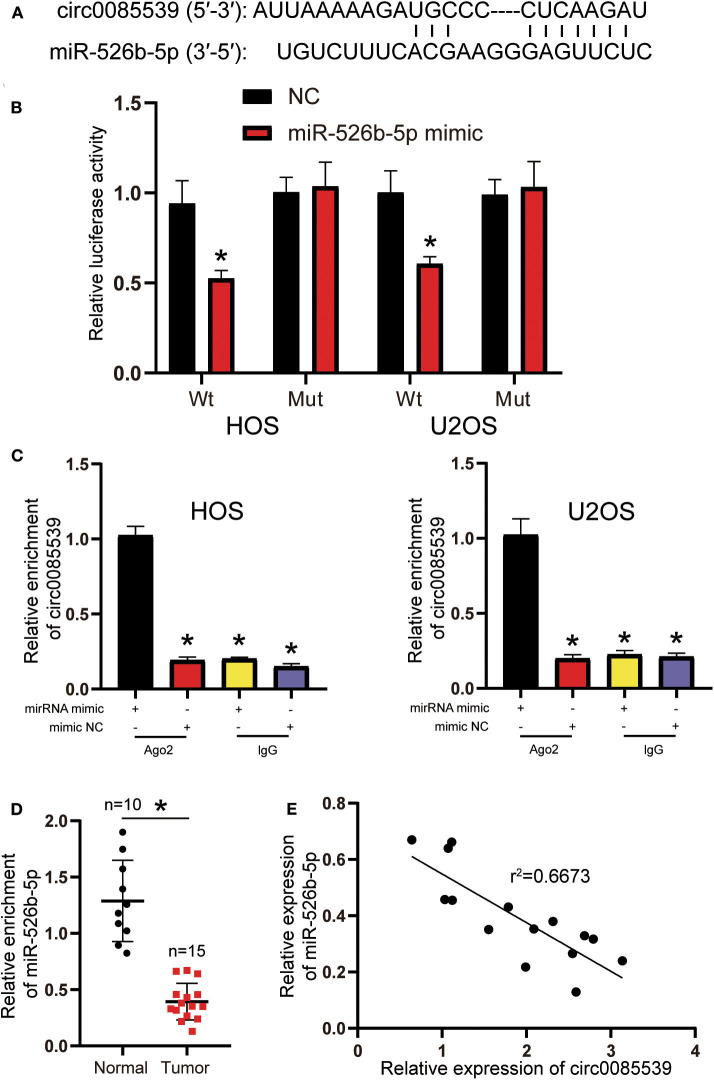
Circ0085539 acted as a sponge for miR-526b-5p. **(A)** Potential binding scheme between miR-526b-5p and circ0085539. **(B)** Luciferase reporter gene assay in osteosarcoma (OS) cells cotransfected with miR-526b-5p mimic and circ0085539-Wt or circ0085539-Mut reporter plasmids. **(C)** RNA immunoprecipitation (RIP) results showed that circ0085539 was abundantly pulled down in miR-526b-5p mimic group in the presence of anti-Ago2 antibodies in HOS and U2OS cells. Immunoglobulin G (IgG) was the negative control. (*p < 0.05 compared with miR-526b-5p mimic + Ago2 group). **(D)** The expression of miR-526b-5p in 15 osteosarcoma and 10 adjacent tissues was determined by quantitative reverse transcription PCR (qRT-PCR) analysis. Normal: adjacent tissues. *p < 0.05 compared with adjacent tissue. **(E)** Correlation analysis showed that miR-526b-5p expression was inversely associated with circ0085539 expression in OS tissues.

Circular RNA Interactome was used to predict the potential binding scheme between circ0085539 and miR-526b-5p. There was only one binding site between the two, and the result is illustrated in Figure 3A. The luciferase reporter gene and RIP assays were both applied to confirm the regulatory relationship between miR-526b-5p and circ0085539. The Wt + miR-526b-5p mimic group was the only group that showed significantly declined luciferase activity in HOS and U2OS cell lines, suggesting that miR-526b-5p could specifically bind to circ0085539 (Figure 3B). Only in HOS and U2OS cells that were transfected with miR-526b-5p mimic, but not in miR-526b-5p NC, that abundant circ0085539 was pulled down in the presence of anti-Ago2 antibody but not IgG (Figure 3C). Not surprisingly, the relative expression of miR-526b-5p in 15 osteosarcoma tissues was only one-third of that in 10 adjacent tissues (Figure 3D), and the correlation analysis implied a negative correlation between the expression of miR-526b-5p and the expression of circ0085539 in 15 OS tissues (Figure 3E). Our findings suggest that circ0085539 could potentially function as a miR-526b-5p sponge in OS.

### Suppression of OS Phenotypes by sh-circ0085539 Was Dependent on miR-526b-5p *in vitro*

**Figure 4:**
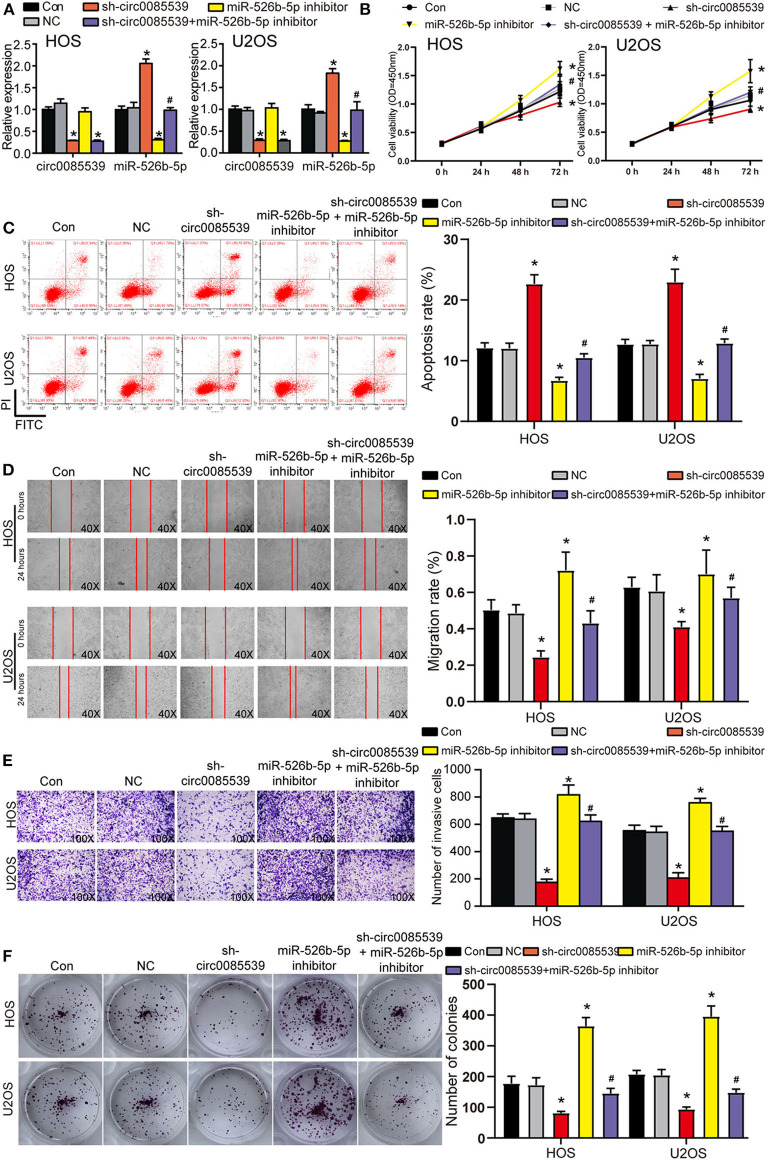
Silence of circ0085539 increased cell apoptosis rate but suppressed the viability, migration, invasion, and proliferation abilities of osteosarcoma (OS) cells in a miR-526b-5p-dependent way. **(A)** The transfection efficiency of sh-circ0085539 plasmids, miR-526b-5p inhibitor, and sh-circ0085539 + miR-526b-5p inhibitor in HOS and U2OS cells was determined by quantitative reverse transcription PCR (qRT-PCR). **(B)** The cell viability of OS cells in the five groups was analyzed by CCK8 assay. **(C)** The apoptosis rate of OS cells in the five groups was analyzed by flow cytometry analysis. **(D)** The migration ability of OS cells in the five groups was analyzed by wound healing assay. Magnification: 40×. **(E)** The invasion ability of OS cells in the five groups was analyzed by Transwell assay. Magnification: 100×. **(F)** The colony formation ability of OS cells in the five groups was analyzed by colony formation assay. Con, control; NC, negative control. *p < 0.05 vs. control. ^#^*p* < 0.05 vs. sh-circ0085539 group.

First, the transfection efficiency of sh-circ0085539 and miR-526b-5p inhibitor reached ~70% in HOS and U2OS cells (Figure 4A). We then studied the effects of sh-circ0085539 and miR-526b-5p inhibitor on cell viability. Circ0085539 silencing obviously suppressed cell viability, while miR-526b-5p inhibitor promoted it. The cotransfection of sh-circ0085539 and miR-526b-5p inhibitor led to an approximately even results with the control group (Figure 4B), suggesting that the enhanced cell viability was caused by the inhibition of miR-526b-5p. Then, circ0085539 knockdown increased cell apoptosis rate in HOS and U2OS cells, while miR-526b-5p inhibitor notably reduced it and abrogated the apoptosis rate increase in response to circ0085539 knockdown (Figure 4C). In addition, silencing circ0085539 significantly impaired the abilities of migration, invasion, and proliferation in OS cells, while miR-526b-5p inhibitor not only enhanced these phenotypes but also attenuated the suppressive effects induced by circ0085539 silencing (Figures 4D–F). It is worth noting that the expression of circ0085539 and miR-526b-5p in cells were detected 14 days after the clone formation, and the results are presented in Supplementary Figure 3A, suggesting that the knockdown of circ0085539 and inhibition of miR-526b-5p remained during the experiment. Our results strongly suggest that the suppression of OS phenotypes by sh-circ0085539 was dependent on miR-526b-5p.

### miR-526b-5p Directly Targeted PHLDA1 in OS Cells

**Figure 5:**
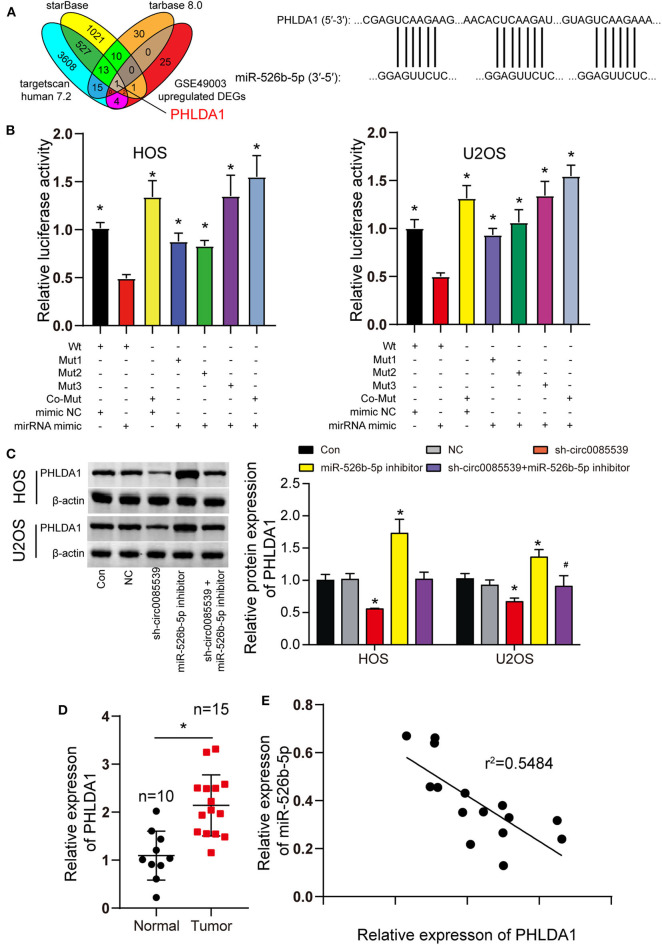
MiR-526b-5p directly targeted pleckstrin homology-like domain family A member 1 (PHLDA1) in osteosarcoma (OS) cells. **(A)** Potential binding sites between miR-526b-5p and PHLDA1 mRNA 3′ untranslated region (UTR). **(B)** Luciferase reporter assay in OS cells cotransfected with miR-526b-5p mimic and PHLDA1-Wt or PHLDA1-Mut reporter plasmids. *p < 0.05 compared with Wt + mimic group. **(C)** Protein expression of PHLDA1 was detected by Western blot analysis. *p < 0.05 compared with control group. ^#^*p* < 0.05 vs. sh-circ0085539 group. **(D)** Quantitative reverse transcription PCR (qRT-PCR) showed that PHLDA1 expression was significantly upregulated in OS tissues. Normal: adjacent tissues. *p < 0.05 compared with normal group. **(E)** Correlation analysis showed that miR-526b-5p expression was inversely associated with PHLDA1 expression in OS tissues.

We predicted the potential targets of miR-526b-5p using starBase, TargetScan Human 7.2, and TarBase 8.0 algorithms. Together with the DEGs from the GSE49003 data series, we identified PHLDA1. The complete results of the three algorithms and GSE49003 are provided in Supplementary Tables 2–5. PHLDA1 might be a downstream target of miR-526b-5p that significantly upregulated in human osteosarcoma. Three independent binding sites were found between miR-526b-5p and PHLDA1 (Figure 5A). Furthermore, the luciferase reporter gene assay results showed that cells that were cotransfected with miR-526b-5p mimic and wild-type PHLDA1 3′ untranslated region (UTR) showed the most significant reduction in luciferase activity (Figure 5B). Our Western blot results showed that miR-526b-5p inhibition led to a significant increase in PHLDA1 protein level. Interestingly, PHLDA1 protein level reduced along with the knockdown of circ0085539. The cotransfection of miR-526b-5p and sh-cir0085539 led to an about even level to the control group (Figure 5C). Not surprisingly, the relative expression of PHLDA1 in OS tissues was twice as high as that in adjacent tissues and was inversely related to the expression of miR-526b-5p in OS tissues (Figures 5D,E). Plus, we detected the expression IHC staining results of PHLDA1 in osteosarcoma tissues and the paired adjacent tissues: PHLDA1 was more in osteosarcoma tissues than in adjacent tissues (Supplementary Figure 4C). Thus, we concluded that there could be a regulatory relationship between miR-526b-5p and PHLDA1 mRNA.

### miR-526b-5p Inhibition Enhanced OS Phenotypes by Regulating PHLDA1 *in vitro*

**Figure 6:**
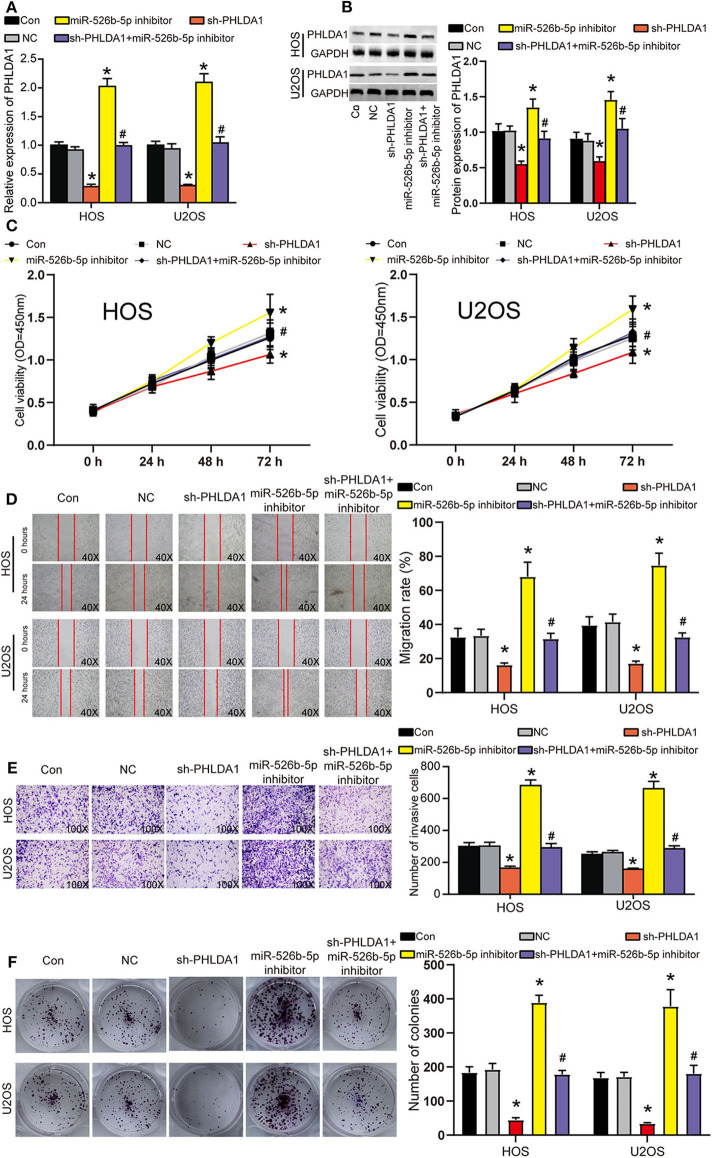
miR-526b-5p inhibition resulted in enhanced the viability, migration, invasion, and proliferation of osteosarcoma (OS) cells in a pleckstrin homology-like domain family A member 1 (PHLDA1)-dependent way. **(A)** The transfection efficiency of sh-PHLDA1, miR-526b-5p inhibitor, and sh-PHLDA1 + miR-526b-5p inhibitor in HOS and U2OS cells was determined at messenger RNA (mRNA) level. **(B)** The transfection efficiency of sh-PHLDA1, miR-526b-5p inhibitor, and sh-PHLDA1 + miR-526b-5p inhibitor in HOS and U2OS cells was determined at protein level. **(C)** The cell viability of OS cells in the five groups was analyzed by CCK8 assay. **(D)** The migration ability of OS cells in the five groups was analyzed by wound healing assay. The migration rate of every group was interpreted. Magnification: 40×. **(E)** The invasion ability of OS cells in the five groups was analyzed by Transwell assay. The invading cell number was counted in every selected field. Magnification: 100×. **(F)** Cell proliferation was determined using colony formation assay. The colony number in every group was analyzed. Con, control; NC, negative control. *p < 0.05 vs. control. ^#^*p* < 0.05 vs. sh-PHLDA1 group.

Before functional experiments, we detected the transfection efficiency of sh-PHLDA1 and miR-526b-5p. The transfection efficiency of sh-PHLDA1 and miR-526b-5p inhibitor reached almost 70% in HOS and U2OS cells (Figure 6A). Seen from the protein level, sh-PHLDA1 caused around half decrease in PHLDA1 protein, while miR-526b-5p inhibitor caused a one-third increase in PHLDA1 protein. The cotransfection of sh-PHLDA1 and miR-526b-5p causes an equal level of PHLDA1 to the control group (Figure 6B). We then studied the effects of sh-PHLDA1 and miR-526b-5p inhibitor on cell viability. PHLDA1 silencing obviously suppressed cell viability, while miR-526b-5p inhibitor promoted it. The cotransfection of sh-PHLDA1 and miR-526b-5p inhibitor led to an approximately equal results with the control group (Figure 6C), suggesting that the enhanced cell viability was caused by miR-526b-5p inhibition. In addition, silencing PHLDA1 significantly impaired the abilities of migration, invasion, and proliferation in OS cells, while miR-526b-5p inhibitor not only enhanced these phenotypes but also attenuated the suppressive effects induced by PHLDA1 silencing (Figures 6D–F, respectively), suggesting that the enhanced cell migration, invasion, and proliferation were caused by miR-526b-5p inhibition. It is worth noting that the expression of PHLDA1 and miR-526b-5p in cells were detected 14 days after the clone formation, and the results are presented in Supplementary Figure 3B, suggesting that the knockdown of PHLDA1 and inhibition of miR-526b-5p remained during the experiment.

## Discussion

Extensive studies have been conducted to discover potential therapeutic targets for osteosarcoma (25, 26). These promising targets cover a wide spectrum of RNAs including circular RNAs, lncRNAs, miRNAs, and mRNAs. With a deeper understanding on human genomics, accumulating circRNAs have been identified to participate in the epigenetic regulation of osteosarcoma and thus considered to be potential therapeutic targets. As a matter of fact, plenty of circRNAs have been reported to be driver genes or suppressors of osteosarcoma, and they were found to regulate osteosarcoma cell activities in an miRNA-dependent manner. For example, upregulated circ0001564 promoted osteosarcoma cell proliferation and impaired the cell apoptosis by sponging miR-29c-3p (27), suggesting its oncogenic role. CircTADA2A functionally promoted the tumorigenesis and metastasis of osteosarcoma via inhibiting miR-203a-3p and releasing CREB3 (28), suggesting its tumor promoter role in OS. Circ001569 functioned as an onco-circRNA in osteosarcoma and promoted cell proliferation through activating the Wnt/β-catenin signaling pathway (29). Inversely, as a suppressor of osteosarcoma, circHIPK3 was obviously downregulated in osteosarcoma, and its enhanced upregulation led to the inhibition of proliferation, migration, and invasion of OS cells (30). These circRNAs were encoded by different genes, and they possessed similar or opposite functions in osteosarcoma. Interestingly, circPVT1 was reported to be a potential new circular RNA biomarker in osteosarcoma and contributed to doxorubicin and cisplatin resistance of osteosarcoma cells by regulating ABCB1 (19). Liu et al. also proved that circPVT1 could promote the invasion and metastasis by sponging miR-205-5p (20). In our previous study, we found that the knockdown of circPVT1 suppressed the migration of OS cells by interacting with miR-526b-5p and FOXC2 *in vitro* (21). Herein, we reported another significantly upregulated circRNA, also encoded by PVT1, circ0085539, in osteosarcoma. It was found that the knockdown of circ0085539 significantly inhibited the progression of OS *in vivo* and *in vitro*, too. PVT1 has long been considered to be an oncogene (31). Our work contributed to a deeper comprehension of OS tumorigenesis pathology and possibly provided a potential therapeutic target for osteosarcoma. In addition, the present work also enriched our last one with further animal experiments and a novel circRNA encoded by the same gene, PVT1.

The study focusing on the role of miR-526b-5p in human cancers has been limitedly reported. We first reported its function in osteosarcoma in 2019 (21). Before us, miR-526b-5p was studied in breast cancer (32) and digestive system neoplasm such as oral squamous cell carcinoma (OSCC) (33), esophageal squamous cell carcinoma (ESCC) (34), gastric cancer (35), and hepatocellular cancer (HCC) (36). miR-526b-5p was found to suppress tumorigenesis phenotypes such as proliferation, migration, and invasion in these cancers. It is also worth noting that miR-526b-5p has been reported to act through a ceRNA network with circular RNA such as circUHRF1 (33), circSPECC1 (35), and circ0091581 (36). Yet, the interaction between miR-526b-5p and circ0085539 has never been reported before us. In addition, the role of miR-526b-5p in osteosarcoma has never been reported before, either. Thus, our current study not only proved that miR-526b-5p could work with a novel circRNA via a ceRNA network in osteosarcoma but also enhanced the comprehension of an underlying mechanism involving miR-526b-5p in osteosarcoma.

PHLDA1, a crucial death mediator, could enhance the apoptotic sensitivity and antiproliferative activity of cells (37, 38). It has been believed to be involved in the regulation of apoptosis including the detachment-mediated programmed cell death and the regulation of antiapoptotic effects of IGF1. There have been several studies regarding PHLDA1's role in human cancers. In breast cancer, PHLDA1 was identified as a strong inhibitor of the metastasis capability of breast cancer cells through regulating Aurora A deregulation, indicating that PHLDA1 functions as a suppressor of breast cancer (39). On the contrary, the migration ability of colorectal cancer cells with PHLDA1 inhibition was obviously weaker than those in the NC group (40). As for the effect of PHLDA1 in OS, limited research has been done. Besides, mounting researches have clarified the proapoptotic role of PHLDA1 (41, 42), while some have considered it as an antiapoptotic factor (24). The opposing views thus suggest that PHLDA1 shows different roles in different cell types and could cause different apoptosis susceptibility of different cancer cells. The apoptotic activation and apoptotic suppression capacity caused by PHLDA1 simultaneously existed in the cancer cell (43). In our research, we identified PHLDA1 as a potential downstream effector of miR-526b-5p and found that the apoptotic suppression capacity of PHLDA1 showed enormous advantages over its apoptotic activation capacity in osteosarcoma cells. PHLDA1 knockdown significantly increased apoptosis and reduced other phenotypes such as proliferation, migration, and invasion in OS cells. Meanwhile, the tumor inhibition in response to PHLDA1 knockdown could be restored by miR-526b-5p inhibition, strongly suggesting PHLDA1 as a downstream target of miR-526b-5p to promote osteosarcoma tumorigenesis. Our research not only provided evidence that PHLDA1 was osteosarcoma promotive but also enriched the downstream network of miR-526b-5p in osteosarcoma.

We have demonstrated the oncogenic effects of circ0085539 in osteosarcoma and enriched the findings that we previously reported. We found that circ0085539 could sponge miR-526b-5p to release PHLDA1, thereby playing the promotion effect on OS. However, PHLDA1 is believed to be involved in mitotic cell cycle especially G2/M phase transition according to Reactome database, which needs to be confirmed yet. On the other hand, a downstream signaling of PHLDA1 in osteosarcoma remains unstudied in this research, which is also worth further exploring. Lastly, whether PHLDA1 promotes metastasis of osteosarcoma remains to be further studied, as osteosarcoma is a highly metastatic malignancy. In the future, we will continue to focus on these issues and conduct in-depth research.

Collectively, our results revealed that circ0085539 was markedly upregulated in osteosarcoma. Functionally, circ0085539 significantly promoted the progression of osteosarcoma through sponging miR-526b-5p to release PHLDA1. Our findings not only identified a novel circRNA and a novel mRNA to be involved in osteosarcoma tumorigenesis but also enriched the network of miR-526b-5p for regulating the OS progression based on our previous study. Lastly, our research suggests that the intervention of circ0085539–miR-526b-5p–PHLDA1 axis can be a potential target in OS therapy.

## Data Availability Statement

The datasets generated for this study are available on request to the corresponding author.

## Ethics Statement

The studies involving human participants were reviewed and approved by the hospital Ethics Committee of The First Hospital of Jilin University. The patients/participants provided their written informed consent to participate in this study.

## Author Contributions

MY and XW contributed to the conception of the study. PL and XW contributed significantly to the analysis and manuscript preparation. HG and YZ performed the data analyses and wrote the manuscript. All authors contributed to the article and approved the submitted version.

## Conflict of Interest

The authors declare that the research was conducted in the absence of any commercial or financial relationships that could be construed as a potential conflict of interest.

## Supplementary Material

Supplementary Figure 1 The selection of the most efficient circ0085539 shRNA. **(A)** The expression of circ0085539 was detected by qRT-PCR in HOS and U2OS cells with the transfection of three shRNAs of circ0085539. ^**^*p* < 0.001 vs. control group. **(B)** The expression of linear 0085539 was detected by qRT-PCR in HOS and U2OS cells with the transfection of three shRNAs of circ0085539. ^**^*p* < 0.001 vs. control group.

Supplementary Figure 2 circ0085539 remained downregulated during the animal experiments. **(A)** The successful establishment of HOS cell line with stabe circ0085539 knockdown. sh-circ0085539 led to the decrease of circ0085539 expression in HOS cells. ^*^*p* < 0.05 vs. control. **(B)** The expression of circ0085539 in xenografted tumor tissues was significantly lower in the nude mice implanted with sh-circ0085539 transfected HOS cells than in those implanted with NC transfected HOS cells after 4 weeks. ^*^*p* < 0.05 vs. NC.

Supplementary Figure 3 circ0085539 and miR-526b-5p remained downregulated after the colony formation experiments. **(A)** The expression of circ0085539 and miR-526b-5p in cells after colony formation experiments in Figure 4. ^*^*p* < 0.05 vs. control. #*p* < 0.05 vs. sh-circ0085539 group. **(B)** The expression of miR-526b-5p and PHLDA1 in cells after colony formation experiments in Figure 6. ^*^*p* < 0.05 vs. control. #*p* < 0.05 vs. sh-PHLDA1 group.

Supplementary Figure 4 Confirmation of the circular structure of circ0085539, and the expression of circ0085539 and PHLDA1 in tissue samples. **(A)** The existence of circ0085539 was validated in osteosarcoma and adjacent tissues using agarose gel electrophoresis. Divergent primers detected circular RNAs in cDNA but not in gDNA. GAPDH was used as the negative control. ▸◂, convergent primer; ◂▸, divergent primer. **(B)** Another 20 pairs of osteosarcoma tissues and adjacent tissues were collected. 10 of them were randomly assigned to training dataset while the other 10 were assigned to the validation dataset. In the two datasets, circ0085539 expression was detected. In this way, the ensurance of the upregulation of circ0085539 in osteosarcoma was enhanced. The training set and validation set method eliminated the falses to a maximum. Normal: adjacent tissues. ^*^*p* < 0.05 vs. normal group. **(C)** The IHC results showing the expression of PHLDA1 in osteosarcoma and adjacent tissues.

Supplementary Table 1 The sequences of the siRNAs and inhibitor in this study.

Supplementary Table 2 the 33 upregulated DEGs were screened out from GSE49003 with *p*-value <0.05 and logFC >2.

Supplementary Table 3 The target genes of miR-526b-5p were predicted by ENCORI starBase.

Supplementary Table 4 The target genes of miR-526b-5p were predicted by Tarbase 8.0.

Supplementary Table 5 The target genes of miR-526b-5p were predicted by TargetScan Human 7.2.
