# Supplementary figures and images for: Circ0085539 Promotes Osteosarcoma Progression by Suppressing miR-526b-5p and PHLDA1 Axis
Source: Front Oncol. 2020 Aug 26;10:1250. doi: 10.3389/fonc.2020.01250 (PMC7479240; doi:10.3389/fonc.2020.01250)

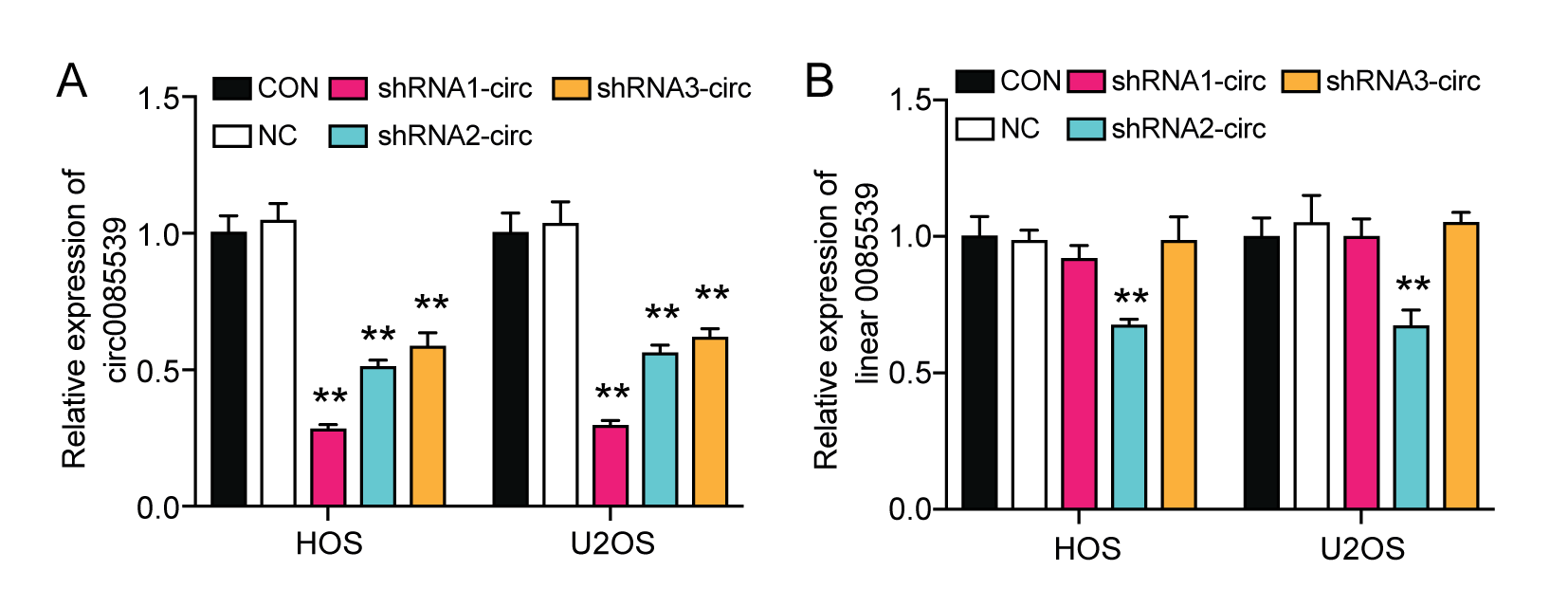

Supplement: Supplementary Figure 1 — The selection of the most efficient circ0085539 shRNA. (A) The expression of circ0085539 was detected by qRT-PCR in HOS and U2OS cells with the transfection of three shRNAs of circ0085539. **p < 0.001 vs. control group. (B) The expression of linear 0085539 was detected by qRT-PCR in HOS and U2OS cells with the transfection of three shRNAs of circ0085539. **p < 0.001 vs. control group. [file Image_1.TIF]

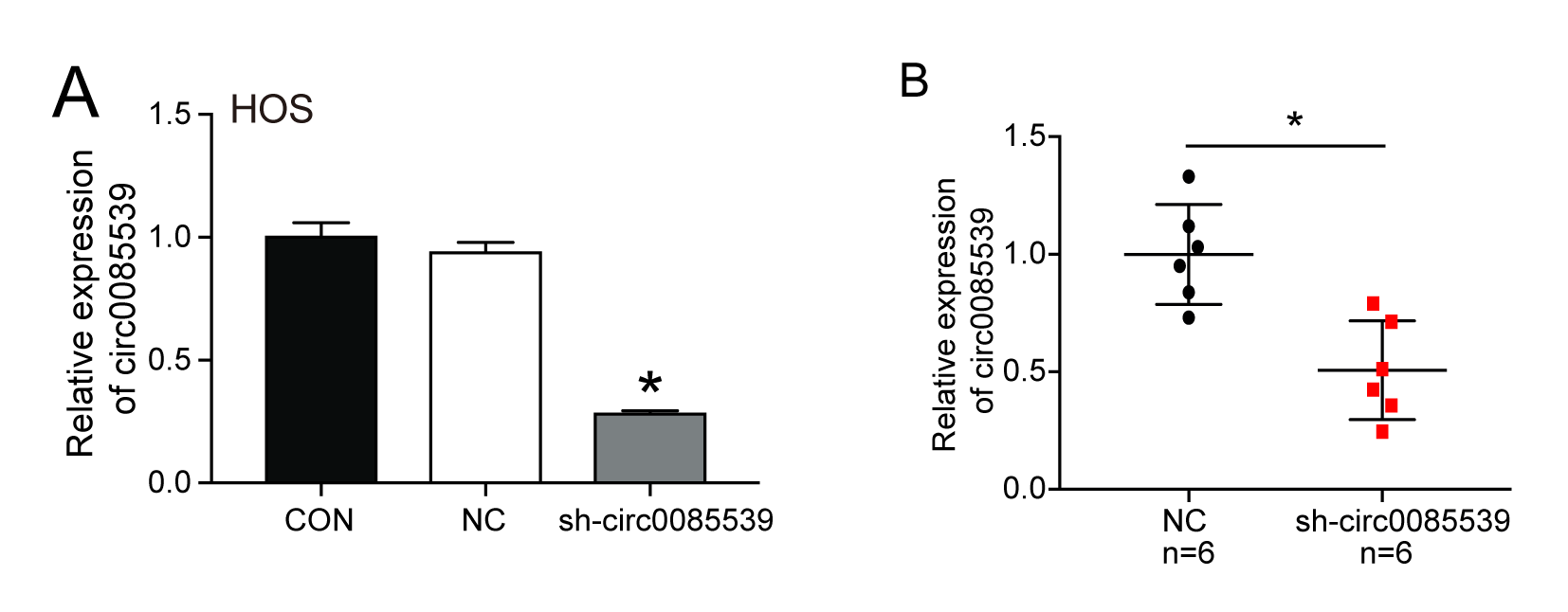

Supplement: Supplementary Figure 2 — circ0085539 remained downregulated during the animal experiments. (A) The successful establishment of HOS cell line with stabe circ0085539 knockdown. sh-circ0085539 led to the decrease of circ0085539 expression in HOS cells. *p < 0.05 vs. control. (B) The expression of circ0085539 in xenografted tumor tissues was significantly lower in the nude mice implanted with sh-circ0085539 transfected HOS cells than in those implanted with NC transfected HOS cells after 4 weeks. *p < 0.05 vs. NC. [file Image_2.TIF]

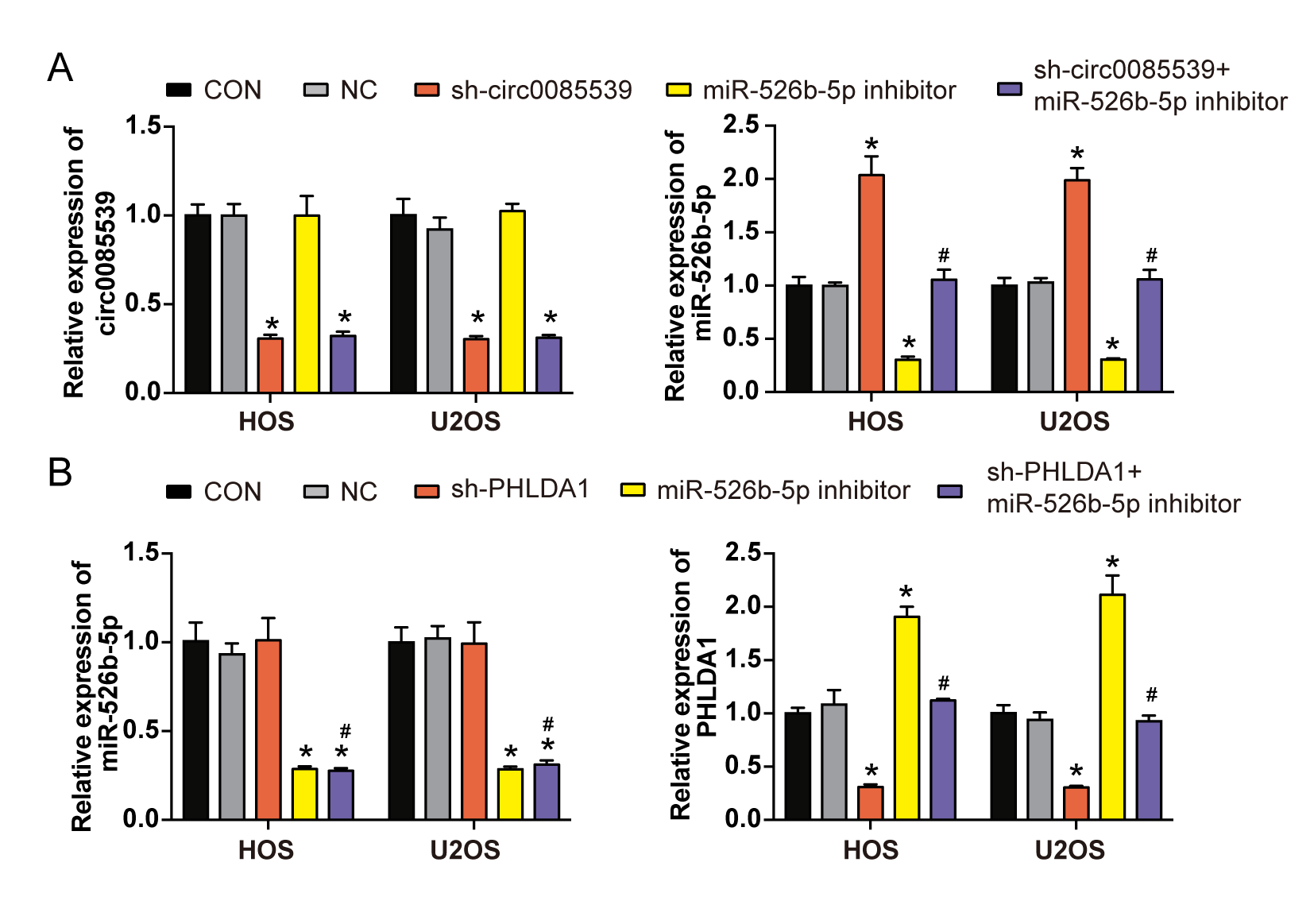

Supplement: Supplementary Figure 3 — circ0085539 and miR-526b-5p remained downregulated after the colony formation experiments. (A) The expression of circ0085539 and miR-526b-5p in cells after colony formation experiments in Figure 4. *p < 0.05 vs. control. #p < 0.05 vs. sh-circ0085539 group. (B) The expression of miR-526b-5p and PHLDA1 in cells after colony formation experiments in Figure 6. *p < 0.05 vs. control. #p < 0.05 vs. sh-PHLDA1 group. [file Image_3.TIF]

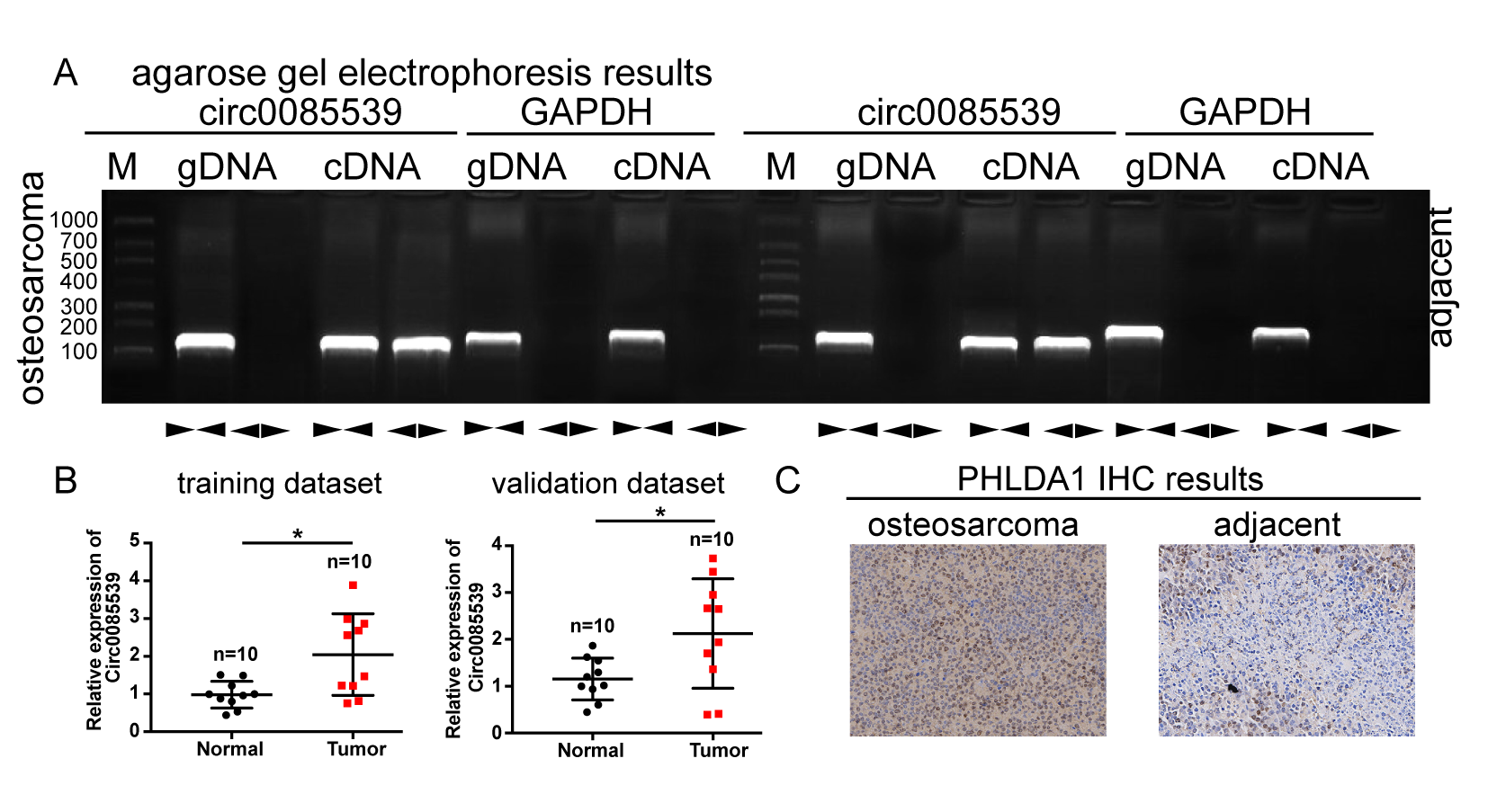

Supplement: Supplementary Figure 4 — Confirmation of the circular structure of circ0085539, and the expression of circ0085539 and PHLDA1 in tissue samples. (A) The existence of circ0085539 was validated in osteosarcoma and adjacent tissues using agarose gel electrophoresis. Divergent primers detected circular RNAs in cDNA but not in gDNA. GAPDH was used as the negative control. ▸◂, convergent primer; ◂▸, divergent primer. (B) Another 20 pairs of osteosarcoma tissues and adjacent tissues were collected. 10 of them were randomly assigned to training dataset while the other 10 were assigned to the validation dataset. In the two datasets, circ0085539 expression was detected. In this way, the ensurance of the upregulation of circ0085539 in osteosarcoma was enhanced. The training set and validation set method eliminated the falses to a maximum. Normal: adjacent tissues. *p < 0.05 vs. normal group. (C) The IHC results showing the expression of PHLDA1 in osteosarcoma and adjacent tissues. [file Image_4.TIF]
