# Supplementary material for: Circ0085539 Promotes Osteosarcoma Progression by Suppressing miR-526b-5p and PHLDA1 Axis
Source: Front Oncol. 2020 Aug 26;10:1250. doi: 10.3389/fonc.2020.01250 (PMC7479240; doi:10.3389/fonc.2020.01250)
Supplement: Supplementary Table 1 — The sequences of the siRNAs and inhibitor in this study. [file Table_1.DOCX]

Supplementary Table 1 The sequences of the siRNAs and inhibitor in this study.

| **Name** | **sequences** |
| --- | --- |
| **siRNA1-circ0085539** |  |
| Forward sequence | 5'-GCUUCUCCUGUUGCUGCUAGU-3' |
| Reverse sequence | 5'-UAGCAGCAACAGGAGAAGCAA-3' |
| **siRNA2-circ0085539** |  |
| Forward sequence | 5’-CUGUGUUCACCUGGUUCAUCU-3’ |
| Reverse sequence | 5’-AUGAACCAGGUGAACACAGAG-3’ |
| **siRNA3-circ0085539** |  |
| Forward sequence | 5’-UGUUGCUGCUAGUGGACAUGA-3’ |
| Reverse sequence | 5’-AUGUCCACUAGCAGCAACAGG-3’ |
| **siRNA-PHLDA1** |  |
| Forward sequence | 5’-GAGCGAUGAUGUACUGUAATT-3’ |
| Reverse sequence | 5-TTACAGUACAUCAUCGCUCCU-3’ |
| **miR-526b inhibitor** | 5’- ACAGAAAGTGCTTCCCTCAAGAG-3’ |
