# Supplementary material for: Circ0085539 Promotes Osteosarcoma Progression by Suppressing miR-526b-5p and PHLDA1 Axis
Source: Front Oncol. 2020 Aug 26;10:1250. doi: 10.3389/fonc.2020.01250 (PMC7479240; doi:10.3389/fonc.2020.01250)
Supplement: Supplementary Table 2 — the 33 upregulated DEGs were screened out from GSE49003 with p-value <0.05 and logFC >2. [file Table_2.DOCX]

Supplementary table 2 the 33 upregulated DEGs were screened out from GSE49003 with p-value <0.05 and logFC >2

| ID | adj.P.Val | P.Value | logFC | Gene.symbol |
| --- | --- | --- | --- | --- |
| ILMN_1656057 | 1.29E-08 | 2.64E-12 | 4.26 | PLAU |
| ILMN_1687978 | 1.18E-07 | 3.39E-11 | 4.09 | PHLDA1 |
| ILMN_1795342 | 1.96E-07 | 6.82E-11 | 3.48 | MLPH |
| ILMN_1745282 | 1.26E-03 | 1.61E-05 | 3.03 | MOK |
| ILMN_1665865 | 8.58E-04 | 9.50E-06 | 3.01 | IGFBP4 |
| ILMN_1792455 | 2.12E-06 | 2.36E-09 | 3 | TMEM158 |
| ILMN_1656501 | 1.05E-04 | 5.57E-07 | 2.88 | DUSP5 |
| ILMN_1770338 | 9.18E-08 | 2.07E-11 | 2.75 | TM4SF1 |
| ILMN_1698179 | 2.03E-06 | 2.17E-09 | 2.74 | TAGLN3 |
| ILMN_2201580 | 2.80E-02 | 1.11E-03 | 2.7 | GSTM2 |
| ILMN_1658494 | 7.46E-07 | 5.05E-10 | 2.63 | RGCC |
| ILMN_1706643 | 8.57E-07 | 6.15E-10 | 2.58 | COL6A3 |
| ILMN_1715401 | 1.72E-05 | 4.20E-08 | 2.53 | MT1G |
| ILMN_1668052 | 2.07E-05 | 5.61E-08 | 2.44 | FOXA2 |
| ILMN_2104141 | 8.53E-10 | 6.99E-14 | 2.39 | FGD5 |
| ILMN_2082209 | 3.72E-07 | 1.85E-10 | 2.35 | TOX2 |
| ILMN_1678143 | 4.89E-02 | 2.50E-03 | 2.33 | ARHGDIB |
| ILMN_1763638 | 1.44E-09 | 1.47E-13 | 2.3 | BCAR3 |
| ILMN_1704154 | 5.01E-06 | 6.78E-09 | 2.3 | TNFRSF19 |
| ILMN_1744604 | 2.12E-02 | 7.72E-04 | 2.29 | CYBA |
| ILMN_1769299 | 8.86E-09 | 1.27E-12 | 2.28 | MTMR11 |
| ILMN_1653856 | 3.80E-04 | 3.18E-06 | 2.27 | UBASH3B |
| ILMN_1726245 | 7.46E-07 | 5.02E-10 | 2.26 | TGFBR2 |
| ILMN_1752046 | 1.06E-05 | 2.14E-08 | 2.26 | SH2B3 |
| ILMN_1764850 | 7.08E-04 | 7.35E-06 | 2.24 | HPCAL1 |
| ILMN_1699695 | 1.04E-06 | 9.17E-10 | 2.22 | TNFRSF21 |
| ILMN_1761968 | 1.03E-03 | 1.24E-05 | 2.16 | PPP1R14A |
| ILMN_1665510 | 1.03E-03 | 1.22E-05 | 2.14 | ERRFI1 |
| ILMN_1763837 | 4.27E-03 | 8.46E-05 | 2.14 | ANPEP |
| ILMN_1703955 | 2.69E-03 | 4.40E-05 | 2.08 | FBXO32 |
| ILMN_1771841 | 5.85E-04 | 5.65E-06 | 2.04 | FOSL1 |
| ILMN_2086105 | 2.23E-05 | 6.18E-08 | 2.03 | SPRY4 |
| ILMN_1803811 | 2.77E-05 | 8.87E-08 | 2.02 | TRIB1 |
