# Supplementary material for: Circ0085539 Promotes Osteosarcoma Progression by Suppressing miR-526b-5p and PHLDA1 Axis
Source: Front Oncol. 2020 Aug 26;10:1250. doi: 10.3389/fonc.2020.01250 (PMC7479240; doi:10.3389/fonc.2020.01250)
Supplement: Supplementary Table 3 — The target genes of miR-526b-5p were predicted by ENCORI starBase. [file Table_3.DOCX]

Supplementary table 3 The target genes of miR-526b-5p were predicted by ENCORI starBase.

| miRNAname | geneID | geneName |
| --- | --- | --- |
| hsa-miR-526b-5p | NM_004421 | DVL1 |
| hsa-miR-526b-5p | NM_031921 | ATAD3B |
| hsa-miR-526b-5p | hsa_circ_0000005 | hsa_circ_002136 |
| hsa-miR-526b-5p | hsa_circ_0000006 | hsa_circ_001621 |
| hsa-miR-526b-5p | NM_002074 | GNB1 |
| hsa-miR-526b-5p | NM_012405 | ICMT |
| hsa-miR-526b-5p | NM_001428 | ENO1 |
| hsa-miR-526b-5p | NM_025106 | SPSB1 |
| hsa-miR-526b-5p | NM_001009566 | CLSTN1 |
| hsa-miR-526b-5p | NM_001286 | CLCN6 |
| hsa-miR-526b-5p | NM_014874 | MFN2 |
| hsa-miR-526b-5p | NM_017556 | FBLIM1 |
| hsa-miR-526b-5p | NM_015001 | SPEN |
| hsa-miR-526b-5p | NM_004431 | EPHA2 |
| hsa-miR-526b-5p | NM_001114600 | C1orf144 |
| hsa-miR-526b-5p | NM_020765 | UBR4 |
| hsa-miR-526b-5p | NM_016183 | MRTO4 |
| hsa-miR-526b-5p | NM_018584 | CAMK2N1 |
| hsa-miR-526b-5p | NM_032236 | USP48 |
| hsa-miR-526b-5p | NM_033631 | LUZP1 |
| hsa-miR-526b-5p | NM_006015 | ARID1A |
| hsa-miR-526b-5p | NM_006600 | NUDC |
| hsa-miR-526b-5p | NM_002946 | RPA2 |
| hsa-miR-526b-5p | NM_001048183 | PHACTR4 |
| hsa-miR-526b-5p | NM_001024732 | MECR |
| hsa-miR-526b-5p | NM_001020658 | PUM1 |
| hsa-miR-526b-5p | hsa_circ_0000045 | hsa_circ_000015 |
| hsa-miR-526b-5p | NM_022164 | TINAGL1 |
| hsa-miR-526b-5p | NM_080391 | PTP4A2 |
| hsa-miR-526b-5p | NM_018056 | TMEM39B |
| hsa-miR-526b-5p | NM_012316 | KPNA6 |
| hsa-miR-526b-5p | NM_175852 | TXLNA |
| hsa-miR-526b-5p | NM_003757 | EIF3I |
| hsa-miR-526b-5p | NM_033504 | TMEM54 |
| hsa-miR-526b-5p | NM_001127361 | RNF19B |
| hsa-miR-526b-5p | NM_004427 | PHC2 |
| hsa-miR-526b-5p | hsa_circ_0011526 | hsa_circ_0011526 |
| hsa-miR-526b-5p | hsa_circ_0011527 | hsa_circ_0011527 |
| hsa-miR-526b-5p | NM_005095 | ZMYM4 |
| hsa-miR-526b-5p | NM_022111 | CLSPN |
| hsa-miR-526b-5p | NM_012199 | EIF2C1 |
| hsa-miR-526b-5p | NM_024852 | EIF2C3 |
| hsa-miR-526b-5p | NM_005119 | THRAP3 |
| hsa-miR-526b-5p | NM_012090 | MACF1 |
| hsa-miR-526b-5p | NM_022733 | SMAP2 |
| hsa-miR-526b-5p | NM_001905 | CTPS |
| hsa-miR-526b-5p | NM_001080850 | CCDC30 |
| hsa-miR-526b-5p | NM_002840 | PTPRF |
| hsa-miR-526b-5p | NM_014663 | KDM4A |
| hsa-miR-526b-5p | NM_014652 | IPO13 |
| hsa-miR-526b-5p | NM_006369 | LRRC41 |
| hsa-miR-526b-5p | NM_199044 | NSUN4 |
| hsa-miR-526b-5p | NM_022745 | ATPAF1 |
| hsa-miR-526b-5p | NM_001048166 | STIL |
| hsa-miR-526b-5p | NM_004631 | LRP8 |
| hsa-miR-526b-5p | NM_176877 | INADL |
| hsa-miR-526b-5p | NM_033407 | DOCK7 |
| hsa-miR-526b-5p | NM_002227 | JAK1 |
| hsa-miR-526b-5p | NM_001902 | CTH |
| hsa-miR-526b-5p | NM_005455 | ZRANB2 |
| hsa-miR-526b-5p | NM_144573 | NEXN |
| hsa-miR-526b-5p | NM_001206651 | SH3GLB1 |
| hsa-miR-526b-5p | NM_001134476 | LRRC8B |
| hsa-miR-526b-5p | NR_036634 | TGFBR3 |
| hsa-miR-526b-5p | NM_001024948 | FNBP1L |
| hsa-miR-526b-5p | NM_002858 | ABCD3 |
| hsa-miR-526b-5p | NM_013296 | GPSM2 |
| hsa-miR-526b-5p | hsa_circ_0000101 | hsa_circ_001839 |
| hsa-miR-526b-5p | NM_018372 | LRIF1 |
| hsa-miR-526b-5p | NM_198268 | HIPK1 |
| hsa-miR-526b-5p | NM_198459 | DENND2C |
| hsa-miR-526b-5p | NM_138959 | VANGL1 |
| hsa-miR-526b-5p | NM_017686 | GDAP2 |
| hsa-miR-526b-5p | NR_037182 | LOC653513 |
| hsa-miR-526b-5p | NM_001037675 | NBPF9 |
| hsa-miR-526b-5p | NM_001039703 | NBPF10 |
| hsa-miR-526b-5p | NM_006472 | TXNIP |
| hsa-miR-526b-5p | hsa_circ_0000122 | hsa_circ_000534 |
| hsa-miR-526b-5p | NM_001097616 | GPR89C |
| hsa-miR-526b-5p | NM_004326 | BCL9 |
| hsa-miR-526b-5p | NM_004284 | CHD1L |
| hsa-miR-526b-5p | NM_030920 | ANP32E |
| hsa-miR-526b-5p | NM_001145415 | SETDB1 |
| hsa-miR-526b-5p | NM_181746 | CERS2 |
| hsa-miR-526b-5p | NM_001136543 | LYSMD1 |
| hsa-miR-526b-5p | NM_015100 | POGZ |
| hsa-miR-526b-5p | NM_020127 | TUFT1 |
| hsa-miR-526b-5p | NM_001083965 | TDRKH |
| hsa-miR-526b-5p | NM_004515 | ILF2 |
| hsa-miR-526b-5p | hsa_circ_0000133 | hsa_circ_001813 |
| hsa-miR-526b-5p | NM_207308 | NUP210L |
| hsa-miR-526b-5p | NM_020452 | ATP8B2 |
| hsa-miR-526b-5p | NM_014949 | KIAA0907 |
| hsa-miR-526b-5p | NM_020131 | UBQLN4 |
| hsa-miR-526b-5p | NM_006617 | NES |
| hsa-miR-526b-5p | NM_198236 | ARHGEF11 |
| hsa-miR-526b-5p | NR_028103 | DCAF8 |
| hsa-miR-526b-5p | NM_001098398 | COPA |
| hsa-miR-526b-5p | NM_007240 | DUSP12 |
| hsa-miR-526b-5p | NM_003666 | BLZF1 |
| hsa-miR-526b-5p | NM_015172 | PRRC2C |
| hsa-miR-526b-5p | NM_014283 | C1orf9 |
| hsa-miR-526b-5p | hsa_circ_0000160 | hsa_circ_001903 |
| hsa-miR-526b-5p | NM_001127181 | CENPL |
| hsa-miR-526b-5p | NM_003101 | SOAT1 |
| hsa-miR-526b-5p | NM_015602 | TOR1AIP1 |
| hsa-miR-526b-5p | NM_014810 | CEP350 |
| hsa-miR-526b-5p | NM_002826 | QSOX1 |
| hsa-miR-526b-5p | NM_004736 | XPR1 |
| hsa-miR-526b-5p | NM_002065 | GLUL |
| hsa-miR-526b-5p | NR_033302 | DHX9 |
| hsa-miR-526b-5p | NM_002293 | LAMC1 |
| hsa-miR-526b-5p | NM_005562 | LAMC2 |
| hsa-miR-526b-5p | NM_052966 | FAM129A |
| hsa-miR-526b-5p | NM_031935 | HMCN1 |
| hsa-miR-526b-5p | NM_003292 | TPR |
| hsa-miR-526b-5p | NM_024529 | CDC73 |
| hsa-miR-526b-5p | hsa_circ_0000167 | hsa_circ_001904 |
| hsa-miR-526b-5p | NM_018136 | ASPM |
| hsa-miR-526b-5p | NM_014875 | KIF14 |
| hsa-miR-526b-5p | NM_203459 | CAMSAP2 |
| hsa-miR-526b-5p | NM_018085 | IPO9 |
| hsa-miR-526b-5p | NM_001114309 | ELF3 |
| hsa-miR-526b-5p | NM_014176 | UBE2T |
| hsa-miR-526b-5p | NM_014827 | ZC3H11A |
| hsa-miR-526b-5p | NM_198447 | GOLT1A |
| hsa-miR-526b-5p | NM_002646 | PIK3C2B |
| hsa-miR-526b-5p | NM_030952 | NUAK2 |
| hsa-miR-526b-5p | NM_182663 | RASSF5 |
| hsa-miR-526b-5p | NM_014873 | LPGAT1 |
| hsa-miR-526b-5p | NR_037667 | INTS7 |
| hsa-miR-526b-5p | NM_016448 | DTL |
| hsa-miR-526b-5p | hsa_circ_0000180 | hsa_circ_001364 |
| hsa-miR-526b-5p | NM_012424 | RPS6KC1 |
| hsa-miR-526b-5p | NM_005401 | PTPN14 |
| hsa-miR-526b-5p | NM_016343 | CENPF |
| hsa-miR-526b-5p | NM_016121 | KCTD3 |
| hsa-miR-526b-5p | TCONS_l2_00001808 | TCONS_l2_00001808 |
| hsa-miR-526b-5p | NM_198551 | MIA3 |
| hsa-miR-526b-5p | NM_144695 | BROX |
| hsa-miR-526b-5p | NM_005426 | TP53BP2 |
| hsa-miR-526b-5p | NM_025160 | WDR26 |
| hsa-miR-526b-5p | NM_001130440 | SRP9 |
| hsa-miR-526b-5p | NM_001618 | PARP1 |
| hsa-miR-526b-5p | NM_003607 | CDC42BPA |
| hsa-miR-526b-5p | NM_001098623 | OBSCN |
| hsa-miR-526b-5p | NM_018230 | NUP133 |
| hsa-miR-526b-5p | NM_012089 | ABCB10 |
| hsa-miR-526b-5p | NM_014777 | URB2 |
| hsa-miR-526b-5p | NM_022051 | EGLN1 |
| hsa-miR-526b-5p | NM_080738 | EDARADD |
| hsa-miR-526b-5p | NM_130398 | EXO1 |
| hsa-miR-526b-5p | NM_014812 | CEP170 |
| hsa-miR-526b-5p | NM_005465 | AKT3 |
| hsa-miR-526b-5p | NM_015446 | AHCTF1 |
| hsa-miR-526b-5p | NM_003431 | ZNF124 |
| hsa-miR-526b-5p | NM_020738 | KIDINS220 |
| hsa-miR-526b-5p | NM_001165931 | RRM2 |
| hsa-miR-526b-5p | NM_134421 | HPCAL1 |
| hsa-miR-526b-5p | NM_002539 | ODC1 |
| hsa-miR-526b-5p | NM_015909 | NBAS |
| hsa-miR-526b-5p | NM_015317 | PUM2 |
| hsa-miR-526b-5p | NM_000384 | APOB |
| hsa-miR-526b-5p | NM_147223 | NCOA1 |
| hsa-miR-526b-5p | NM_018263 | ASXL2 |
| hsa-miR-526b-5p | NM_000182 | HADHA |
| hsa-miR-526b-5p | NM_002709 | PPP1CB |
| hsa-miR-526b-5p | NM_017910 | TRMT61B |
| hsa-miR-526b-5p | NM_015131 | WDR43 |
| hsa-miR-526b-5p | NM_182551 | LCLAT1 |
| hsa-miR-526b-5p | NM_016252 | BIRC6 |
| hsa-miR-526b-5p | hsa_circ_0000989 | hsa_circ_002146 |
| hsa-miR-526b-5p | NM_015475 | FAM98A |
| hsa-miR-526b-5p | NM_016441 | CRIM1 |
| hsa-miR-526b-5p | NM_003162 | STRN |
| hsa-miR-526b-5p | NM_005760 | CEBPZ |
| hsa-miR-526b-5p | NM_001142650 | HNRPLL |
| hsa-miR-526b-5p | NM_005633 | SOS1 |
| hsa-miR-526b-5p | NM_021097 | SLC8A1 |
| hsa-miR-526b-5p | hsa_circ_0000994 | hsa_circ_000303 |
| hsa-miR-526b-5p | NM_005400 | PRKCE |
| hsa-miR-526b-5p | NM_001201965 | ASB3 |
| hsa-miR-526b-5p | hsa_circ_0001005 | hsa_circ_001799 |
| hsa-miR-526b-5p | NM_003128 | SPTBN1 |
| hsa-miR-526b-5p | hsa_circ_0001010 | hsa_circ_001857 |
| hsa-miR-526b-5p | NM_014709 | USP34 |
| hsa-miR-526b-5p | NM_003400 | XPO1 |
| hsa-miR-526b-5p | hsa_circ_0001016 | hsa_circ_001767 |
| hsa-miR-526b-5p | hsa_circ_0001017 | hsa_circ_002020 |
| hsa-miR-526b-5p | NM_006430 | CCT4 |
| hsa-miR-526b-5p | NM_015252 | EHBP1 |
| hsa-miR-526b-5p | NM_006759 | UGP2 |
| hsa-miR-526b-5p | hsa_circ_0001020 | hsa_circ_001739 |
| hsa-miR-526b-5p | NM_203437 | AFTPH |
| hsa-miR-526b-5p | hsa_circ_0001021 | hsa_circ_000026* |
| hsa-miR-526b-5p | NM_014755 | SERTAD2 |
| hsa-miR-526b-5p | NM_001193493 | SLC1A4 |
| hsa-miR-526b-5p | NM_014911 | AAK1 |
| hsa-miR-526b-5p | NM_002357 | MXD1 |
| hsa-miR-526b-5p | NM_022173 | TIA1 |
| hsa-miR-526b-5p | NM_015120 | ALMS1 |
| hsa-miR-526b-5p | hsa_circ_0001031 | hsa_circ_001126 |
| hsa-miR-526b-5p | NM_213622 | STAMBP |
| hsa-miR-526b-5p | NM_080916 | DGUOK |
| hsa-miR-526b-5p | NM_144993 | TET3 |
| hsa-miR-526b-5p | NR_027405 | MTHFD2 |
| hsa-miR-526b-5p | NM_004082 | DCTN1 |
| hsa-miR-526b-5p | NM_032779 | CCDC142 |
| hsa-miR-526b-5p | NM_053050 | MRPL53 |
| hsa-miR-526b-5p | NM_181575 | AUP1 |
| hsa-miR-526b-5p | NM_000821 | GGCX |
| hsa-miR-526b-5p | NM_018433 | KDM3A |
| hsa-miR-526b-5p | NM_022780 | RMND5A |
| hsa-miR-526b-5p | NM_016316 | REV1 |
| hsa-miR-526b-5p | NM_002518 | NPAS2 |
| hsa-miR-526b-5p | NM_182588 | RGPD4 |
| hsa-miR-526b-5p | NM_006267 | RANBP2 |
| hsa-miR-526b-5p | NM_001193485 | LIMS1 |
| hsa-miR-526b-5p | NM_022336 | EDAR |
| hsa-miR-526b-5p | NM_001099289 | SH3RF3 |
| hsa-miR-526b-5p | NM_001164463 | RGPD8 |
| hsa-miR-526b-5p | NM_022662 | ANAPC1 |
| hsa-miR-526b-5p | NM_001037866 | RGPD6 |
| hsa-miR-526b-5p | NM_153712 | TTL |
| hsa-miR-526b-5p | NM_019014 | POLR1B |
| hsa-miR-526b-5p | NM_005721 | ACTR3 |
| hsa-miR-526b-5p | NM_182915 | STEAP3 |
| hsa-miR-526b-5p | NM_002830 | PTPN4 |
| hsa-miR-526b-5p | NM_015282 | CLASP1 |
| hsa-miR-526b-5p | NM_017969 | IWS1 |
| hsa-miR-526b-5p | NM_001006623 | WDR33 |
| hsa-miR-526b-5p | NR_027671 | UGGT1 |
| hsa-miR-526b-5p | NM_033416 | IMP4 |
| hsa-miR-526b-5p | NM_002410 | MGAT5 |
| hsa-miR-526b-5p | NM_058241 | CCNT2 |
| hsa-miR-526b-5p | NM_014607 | UBXN4 |
| hsa-miR-526b-5p | NM_001008540 | CXCR4 |
| hsa-miR-526b-5p | NM_015630 | EPC2 |
| hsa-miR-526b-5p | NM_015702 | MMADHC |
| hsa-miR-526b-5p | NM_001177665 | RIF1 |
| hsa-miR-526b-5p | NM_012097 | ARL5A |
| hsa-miR-526b-5p | NM_001083112 | GPD2 |
| hsa-miR-526b-5p | NM_003628 | PKP4 |
| hsa-miR-526b-5p | NM_033394 | TANC1 |
| hsa-miR-526b-5p | NM_013450 | BAZ2B |
| hsa-miR-526b-5p | NM_022826 | 7-Mar |
| hsa-miR-526b-5p | NM_024753 | TTC21B |
| hsa-miR-526b-5p | NM_172070 | UBR3 |
| hsa-miR-526b-5p | NM_025000 | DCAF17 |
| hsa-miR-526b-5p | hsa_circ_0001081 | hsa_circ_001794 |
| hsa-miR-526b-5p | NM_000210 | ITGA6 |
| hsa-miR-526b-5p | NM_001077269 | WIPF1 |
| hsa-miR-526b-5p | NM_003659 | AGPS |
| hsa-miR-526b-5p | NM_133378 | TTN |
| hsa-miR-526b-5p | NM_002210 | ITGAV |
| hsa-miR-526b-5p | NM_006287 | TFPI |
| hsa-miR-526b-5p | NM_000090 | COL3A1 |
| hsa-miR-526b-5p | NM_007315 | STAT1 |
| hsa-miR-526b-5p | NM_001130158 | MYO1B |
| hsa-miR-526b-5p | NM_004657 | SDPR |
| hsa-miR-526b-5p | NM_004226 | STK17B |
| hsa-miR-526b-5p | hsa_circ_0001086 | hsa_circ_000916 |
| hsa-miR-526b-5p | NM_001100422 | SPATS2L |
| hsa-miR-526b-5p | NM_001159 | AOX1 |
| hsa-miR-526b-5p | NM_203365 | RAPH1 |
| hsa-miR-526b-5p | NM_017759 | INO80D |
| hsa-miR-526b-5p | NM_199229 | RPE |
| hsa-miR-526b-5p | NM_001875 | CPS1 |
| hsa-miR-526b-5p | NM_021141 | XRCC5 |
| hsa-miR-526b-5p | NM_014140 | SMARCAL1 |
| hsa-miR-526b-5p | NM_007127 | VIL1 |
| hsa-miR-526b-5p | NM_020935 | USP37 |
| hsa-miR-526b-5p | NM_001105537 | ZNF142 |
| hsa-miR-526b-5p | NM_014640 | TTLL4 |
| hsa-miR-526b-5p | NM_014689 | DOCK10 |
| hsa-miR-526b-5p | NM_139072 | DNER |
| hsa-miR-526b-5p | NM_004238 | TRIP12 |
| hsa-miR-526b-5p | NM_138402 | SP140L |
| hsa-miR-526b-5p | hsa_circ_0001105 | hsa_circ_001735 |
| hsa-miR-526b-5p | hsa_circ_0001106 | hsa_circ_001283 |
| hsa-miR-526b-5p | NM_001080391 | SP100 |
| hsa-miR-526b-5p | NR_002908 | SNORD20 |
| hsa-miR-526b-5p | NM_152383 | DIS3L2 |
| hsa-miR-526b-5p | NM_004544 | NDUFA10 |
| hsa-miR-526b-5p | hsa_circ_0001118 | hsa_circ_001285 |
| hsa-miR-526b-5p | hsa_circ_0001119 | hsa_circ_002094 |
| hsa-miR-526b-5p | NM_001008491 | 2-Sep |
| hsa-miR-526b-5p | NM_016302 | CRBN |
| hsa-miR-526b-5p | NM_182760 | SUMF1 |
| hsa-miR-526b-5p | NM_001080517 | SETD5 |
| hsa-miR-526b-5p | NM_207351 | PRRT3 |
| hsa-miR-526b-5p | NM_014760 | TATDN2 |
| hsa-miR-526b-5p | NM_006395 | ATG7 |
| hsa-miR-526b-5p | NM_024923 | NUP210 |
| hsa-miR-526b-5p | NM_022340 | ZFYVE20 |
| hsa-miR-526b-5p | NM_004844 | SH3BP5 |
| hsa-miR-526b-5p | hsa_circ_0001274 | hsa_circ_001828 |
| hsa-miR-526b-5p | NM_001134380 | TBC1D5 |
| hsa-miR-526b-5p | NM_001199251 | SGOL1 |
| hsa-miR-526b-5p | NM_001068 | TOP2B |
| hsa-miR-526b-5p | NM_001024847 | TGFBR2 |
| hsa-miR-526b-5p | NM_017801 | CMTM6 |
| hsa-miR-526b-5p | NM_014517 | UBP1 |
| hsa-miR-526b-5p | NM_001162429 | PDCD6IP |
| hsa-miR-526b-5p | NM_001172567 | MYD88 |
| hsa-miR-526b-5p | NM_005109 | OXSR1 |
| hsa-miR-526b-5p | NM_002295 | RPSA |
| hsa-miR-526b-5p | NM_001098209 | CTNNB1 |
| hsa-miR-526b-5p | NM_005385 | NKTR |
| hsa-miR-526b-5p | NM_032806 | C3orf39 |
| hsa-miR-526b-5p | NM_020696 | KIAA1143 |
| hsa-miR-526b-5p | NM_014159 | SETD2 |
| hsa-miR-526b-5p | NM_001040454 | SLC26A6 |
| hsa-miR-526b-5p | NM_001005909 | IP6K2 |
| hsa-miR-526b-5p | NM_018031 | WDR6 |
| hsa-miR-526b-5p | NM_005051 | QARS |
| hsa-miR-526b-5p | NM_001199161 | USP19 |
| hsa-miR-526b-5p | NM_002292 | LAMB2 |
| hsa-miR-526b-5p | NM_001640 | APEH |
| hsa-miR-526b-5p | NM_013334 | GMPPB |
| hsa-miR-526b-5p | NM_024046 | CAMKV |
| hsa-miR-526b-5p | NM_005777 | RBM6 |
| hsa-miR-526b-5p | NM_004186 | SEMA3F |
| hsa-miR-526b-5p | NM_006764 | IFRD2 |
| hsa-miR-526b-5p | NM_007024 | TMEM115 |
| hsa-miR-526b-5p | NM_025222 | WDR82 |
| hsa-miR-526b-5p | NM_007184 | NISCH |
| hsa-miR-526b-5p | NM_018313 | PBRM1 |
| hsa-miR-526b-5p | NM_018397 | CHDH |
| hsa-miR-526b-5p | NM_022899 | ACTR8 |
| hsa-miR-526b-5p | NM_001112736 | FAM208A |
| hsa-miR-526b-5p | NM_002841 | PTPRG |
| hsa-miR-526b-5p | NM_198859 | PRICKLE2 |
| hsa-miR-526b-5p | NM_016206 | VGLL3 |
| hsa-miR-526b-5p | NM_173824 | C3orf38 |
| hsa-miR-526b-5p | NM_006100 | ST3GAL6 |
| hsa-miR-526b-5p | NM_001850 | COL8A1 |
| hsa-miR-526b-5p | NM_032359 | C3orf26 |
| hsa-miR-526b-5p | NM_014820 | TOMM70A |
| hsa-miR-526b-5p | NM_014415 | ZBTB11 |
| hsa-miR-526b-5p | NM_001690 | ATP6V1A |
| hsa-miR-526b-5p | NM_004487 | GOLGB1 |
| hsa-miR-526b-5p | NM_138287 | DTX3L |
| hsa-miR-526b-5p | NM_053025 | MYLK |
| hsa-miR-526b-5p | NM_024628 | SLC12A8 |
| hsa-miR-526b-5p | NM_032242 | PLXNA1 |
| hsa-miR-526b-5p | NM_021937 | EEFSEC |
| hsa-miR-526b-5p | NM_002950 | RPN1 |
| hsa-miR-526b-5p | NR_033426 | ACAD9 |
| hsa-miR-526b-5p | NM_052990 | IFT122 |
| hsa-miR-526b-5p | NM_015103 | PLXND1 |
| hsa-miR-526b-5p | NM_015268 | DNAJC13 |
| hsa-miR-526b-5p | NM_005862 | STAG1 |
| hsa-miR-526b-5p | NR_023350 | COPB2 |
| hsa-miR-526b-5p | NM_006506 | RASA2 |
| hsa-miR-526b-5p | NM_019001 | XRN1 |
| hsa-miR-526b-5p | NM_001184 | ATR |
| hsa-miR-526b-5p | NM_001080415 | U2SURP |
| hsa-miR-526b-5p | NM_004130 | GYG1 |
| hsa-miR-526b-5p | NM_001251962 | ARHGEF26 |
| hsa-miR-526b-5p | NM_015938 | NMD3 |
| hsa-miR-526b-5p | NM_024947 | PHC3 |
| hsa-miR-526b-5p | NM_018098 | ECT2 |
| hsa-miR-526b-5p | NM_016331 | ZNF639 |
| hsa-miR-526b-5p | NM_021629 | GNB4 |
| hsa-miR-526b-5p | hsa_circ_0001364 | hsa_circ_002097 |
| hsa-miR-526b-5p | NM_018358 | ABCF3 |
| hsa-miR-526b-5p | NM_130837 | OPA1 |
| hsa-miR-526b-5p | NR_003265 | SDHAP2 |
| hsa-miR-526b-5p | NM_152699 | SENP5 |
| hsa-miR-526b-5p | NM_004087 | DLG1 |
| hsa-miR-526b-5p | NM_001145642 | KIAA0226 |
| hsa-miR-526b-5p | NM_001011537 | FYTTD1 |
| hsa-miR-526b-5p | NM_001127178 | PIGG |
| hsa-miR-526b-5p | NM_133334 | WHSC1 |
| hsa-miR-526b-5p | NM_024511 | HAUS3 |
| hsa-miR-526b-5p | NM_002111 | HTT |
| hsa-miR-526b-5p | NM_001206996 | PPP2R2C |
| hsa-miR-526b-5p | NM_017491 | WDR1 |
| hsa-miR-526b-5p | NM_004249 | RAB28 |
| hsa-miR-526b-5p | NM_001193535 | FBXL5 |
| hsa-miR-526b-5p | NM_001166139 | LCORL |
| hsa-miR-526b-5p | NM_001204747 | RFC1 |
| hsa-miR-526b-5p | NM_001024921 | RPL9 |
| hsa-miR-526b-5p | NM_001100399 | PDS5A |
| hsa-miR-526b-5p | NM_014988 | LIMCH1 |
| hsa-miR-526b-5p | NM_138335 | GNPDA2 |
| hsa-miR-526b-5p | NM_015030 | FRYL |
| hsa-miR-526b-5p | NM_001134937 | FIP1L1 |
| hsa-miR-526b-5p | NM_006206 | PDGFRA |
| hsa-miR-526b-5p | NM_004898 | CLOCK |
| hsa-miR-526b-5p | NM_006947 | SRP72 |
| hsa-miR-526b-5p | NM_032217 | ANKRD17 |
| hsa-miR-526b-5p | NM_000477 | ALB |
| hsa-miR-526b-5p | NM_001657 | AREG |
| hsa-miR-526b-5p | hsa_circ_0069992 | hsa_circ_0069992 |
| hsa-miR-526b-5p | hsa_circ_0069994 | hsa_circ_0069994 |
| hsa-miR-526b-5p | NM_018115 | SDAD1 |
| hsa-miR-526b-5p | NM_025074 | FRAS1 |
| hsa-miR-526b-5p | NM_017593 | BMP2K |
| hsa-miR-526b-5p | NM_001077207 | SEC31A |
| hsa-miR-526b-5p | NM_024672 | THAP9 |
| hsa-miR-526b-5p | NM_006457 | PDLIM5 |
| hsa-miR-526b-5p | NM_003728 | UNC5C |
| hsa-miR-526b-5p | NM_001135147 | SLC39A8 |
| hsa-miR-526b-5p | NM_005908 | MANBA |
| hsa-miR-526b-5p | NM_001813 | CENPE |
| hsa-miR-526b-5p | NM_006323 | SEC24B |
| hsa-miR-526b-5p | NM_176824 | BBS7 |
| hsa-miR-526b-5p | NM_002006 | FGF2 |
| hsa-miR-526b-5p | NM_014264 | PLK4 |
| hsa-miR-526b-5p | NM_152778 | MFSD8 |
| hsa-miR-526b-5p | NM_001199282 | LRBA |
| hsa-miR-526b-5p | NM_001025595 | ARFIP1 |
| hsa-miR-526b-5p | NM_001131007 | KIAA0922 |
| hsa-miR-526b-5p | NM_014247 | RAPGEF2 |
| hsa-miR-526b-5p | NM_001100389 | TMEM192 |
| hsa-miR-526b-5p | NM_173872 | CLCN3 |
| hsa-miR-526b-5p | NM_001034845 | GALNTL6 |
| hsa-miR-526b-5p | NM_018359 | UFSP2 |
| hsa-miR-526b-5p | NM_005245 | FAT1 |
| hsa-miR-526b-5p | NM_007277 | EXOC3 |
| hsa-miR-526b-5p | NM_018140 | CEP72 |
| hsa-miR-526b-5p | NM_015325 | KIAA0947 |
| hsa-miR-526b-5p | NM_017755 | NSUN2 |
| hsa-miR-526b-5p | hsa_circ_0001466 | hsa_circ_000403 |
| hsa-miR-526b-5p | NM_002454 | MTRR |
| hsa-miR-526b-5p | NM_012073 | CCT5 |
| hsa-miR-526b-5p | NM_007118 | TRIO |
| hsa-miR-526b-5p | NM_138348 | FAM105B |
| hsa-miR-526b-5p | NM_006713 | SUB1 |
| hsa-miR-526b-5p | NM_023073 | C5orf42 |
| hsa-miR-526b-5p | NM_153485 | NUP155 |
| hsa-miR-526b-5p | NM_018034 | WDR70 |
| hsa-miR-526b-5p | NM_152756 | RICTOR |
| hsa-miR-526b-5p | NM_002495 | NDUFS4 |
| hsa-miR-526b-5p | NM_001008397 | GPX8 |
| hsa-miR-526b-5p | NM_002184 | IL6ST |
| hsa-miR-526b-5p | NM_005921 | MAP3K1 |
| hsa-miR-526b-5p | NM_001127236 | GPBP1 |
| hsa-miR-526b-5p | NM_001098728 | GTF2H2C |
| hsa-miR-526b-5p | TCONS_l2_00023407 | TCONS_l2_00023407 |
| hsa-miR-526b-5p | NM_017411 | SMN2 |
| hsa-miR-526b-5p | NR_033417 | GTF2H2B |
| hsa-miR-526b-5p | hsa_circ_0006721 | hsa_circ_0006721 |
| hsa-miR-526b-5p | hsa_circ_0072815 | hsa_circ_0072815 |
| hsa-miR-526b-5p | NM_018429 | BDP1 |
| hsa-miR-526b-5p | NM_005909 | MAP1B |
| hsa-miR-526b-5p | NM_002270 | TNPO1 |
| hsa-miR-526b-5p | NM_138782 | FCHO2 |
| hsa-miR-526b-5p | NM_001105251 | ZFYVE16 |
| hsa-miR-526b-5p | NM_004385 | VCAN |
| hsa-miR-526b-5p | NM_032119 | GPR98 |
| hsa-miR-526b-5p | NM_014899 | RHOBTB3 |
| hsa-miR-526b-5p | NM_012081 | ELL2 |
| hsa-miR-526b-5p | NM_018343 | RIOK2 |
| hsa-miR-526b-5p | NM_001270 | CHD1 |
| hsa-miR-526b-5p | NM_005246 | FER |
| hsa-miR-526b-5p | NM_014819 | PJA2 |
| hsa-miR-526b-5p | NM_022140 | EPB41L4A |
| hsa-miR-526b-5p | NM_001127510 | APC |
| hsa-miR-526b-5p | NM_005023 | PGGT1B |
| hsa-miR-526b-5p | NM_005509 | DMXL1 |
| hsa-miR-526b-5p | NM_153223 | CEP120 |
| hsa-miR-526b-5p | NM_032177 | PHAX |
| hsa-miR-526b-5p | NM_001198557 | LMNB1 |
| hsa-miR-526b-5p | NR_015360 | FLJ33630 |
| hsa-miR-526b-5p | NM_133638 | ADAMTS19 |
| hsa-miR-526b-5p | NM_001164387 | RAPGEF6 |
| hsa-miR-526b-5p | NM_002154 | HSPA4 |
| hsa-miR-526b-5p | NM_014829 | DDX46 |
| hsa-miR-526b-5p | NM_001135586 | C5orf24 |
| hsa-miR-526b-5p | TCONS_00009789 | TCONS_00009789 |
| hsa-miR-526b-5p | TCONS_00010097 | TCONS_00010097 |
| hsa-miR-526b-5p | NM_001037633 | SIL1 |
| hsa-miR-526b-5p | NM_018834 | MATR3 |
| hsa-miR-526b-5p | NM_017747 | ANKHD1 |
| hsa-miR-526b-5p | NM_020690 | ANKHD1-EIF4EBP3 |
| hsa-miR-526b-5p | hsa_circ_0001542 | hsa_circ_000418 |
| hsa-miR-526b-5p | NM_080670 | SLC35A4 |
| hsa-miR-526b-5p | NM_020117 | LARS |
| hsa-miR-526b-5p | NM_133263 | PPARGC1B |
| hsa-miR-526b-5p | NM_001543 | NDST1 |
| hsa-miR-526b-5p | NM_001135643 | DCTN4 |
| hsa-miR-526b-5p | NM_003118 | SPARC |
| hsa-miR-526b-5p | NM_198321 | GALNT10 |
| hsa-miR-526b-5p | NM_004821 | HAND1 |
| hsa-miR-526b-5p | NM_015315 | LARP1 |
| hsa-miR-526b-5p | NM_001199383 | RNF145 |
| hsa-miR-526b-5p | NM_001122679 | ODZ2 |
| hsa-miR-526b-5p | NM_017785 | CCDC99 |
| hsa-miR-526b-5p | NM_022897 | RANBP17 |
| hsa-miR-526b-5p | NM_012300 | FBXW11 |
| hsa-miR-526b-5p | NM_198567 | C5orf25 |
| hsa-miR-526b-5p | NM_014613 | FAF2 |
| hsa-miR-526b-5p | NM_022455 | NSD1 |
| hsa-miR-526b-5p | NR_024019 | FAM193B |
| hsa-miR-526b-5p | NM_017510 | TMED9 |
| hsa-miR-526b-5p | NR_026921 | LOC202181 |
| hsa-miR-526b-5p | NM_001114620 | MGAT1 |
| hsa-miR-526b-5p | NM_002460 | IRF4 |
| hsa-miR-526b-5p | NM_003913 | PRPF4B |
| hsa-miR-526b-5p | NM_002114 | HIVEP1 |
| hsa-miR-526b-5p | NM_016495 | TBC1D7 |
| hsa-miR-526b-5p | NM_005493 | RANBP9 |
| hsa-miR-526b-5p | hsa_circ_0001577 | hsa_circ_001598 |
| hsa-miR-526b-5p | hsa_circ_0001578 | hsa_circ_001173 |
| hsa-miR-526b-5p | NM_005124 | NUP153 |
| hsa-miR-526b-5p | NM_001105568 | KIF13A |
| hsa-miR-526b-5p | hsa_circ_0075955 | hsa_circ_0075955 |
| hsa-miR-526b-5p | hsa_circ_0075961 | hsa_circ_0075961 |
| hsa-miR-526b-5p | NM_001188 | BAK1 |
| hsa-miR-526b-5p | NM_002224 | ITPR3 |
| hsa-miR-526b-5p | NM_017754 | UHRF1BP1 |
| hsa-miR-526b-5p | NM_003427 | ZNF76 |
| hsa-miR-526b-5p | NM_022047 | DEF6 |
| hsa-miR-526b-5p | NM_007104 | RPL10A |
| hsa-miR-526b-5p | NM_003137 | SRPK1 |
| hsa-miR-526b-5p | NM_021943 | ZFAND3 |
| hsa-miR-526b-5p | NM_006708 | GLO1 |
| hsa-miR-526b-5p | NM_018322 | C6orf64 |
| hsa-miR-526b-5p | NM_004275 | MED20 |
| hsa-miR-526b-5p | NM_004053 | BYSL |
| hsa-miR-526b-5p | NM_033502 | TRERF1 |
| hsa-miR-526b-5p | NM_006586 | CNPY3 |
| hsa-miR-526b-5p | NM_014345 | ZNF318 |
| hsa-miR-526b-5p | NM_020750 | XPO5 |
| hsa-miR-526b-5p | NM_018135 | MRPS18A |
| hsa-miR-526b-5p | NM_001024630 | RUNX2 |
| hsa-miR-526b-5p | NM_015548 | DST |
| hsa-miR-526b-5p | hsa_circ_0076872 | hsa_circ_0076872 |
| hsa-miR-526b-5p | NM_001402 | EEF1A1 |
| hsa-miR-526b-5p | NM_133493 | CD109 |
| hsa-miR-526b-5p | NM_004370 | COL12A1 |
| hsa-miR-526b-5p | NM_015571 | SENP6 |
| hsa-miR-526b-5p | hsa_circ_0001613 | hsa_circ_000747 |
| hsa-miR-526b-5p | NM_001010844 | IRAK1BP1 |
| hsa-miR-526b-5p | NM_001199917 | PGM3 |
| hsa-miR-526b-5p | NM_002395 | ME1 |
| hsa-miR-526b-5p | NM_015021 | ZNF292 |
| hsa-miR-526b-5p | NM_020320 | RARS2 |
| hsa-miR-526b-5p | NM_001010853 | PM20D2 |
| hsa-miR-526b-5p | NM_014611 | MDN1 |
| hsa-miR-526b-5p | NM_012160 | FBXL4 |
| hsa-miR-526b-5p | hsa_circ_0001629 | hsa_circ_001181 |
| hsa-miR-526b-5p | NM_001004317 | LIN28B |
| hsa-miR-526b-5p | NM_001624 | AIM1 |
| hsa-miR-526b-5p | NM_032131 | ARMC2 |
| hsa-miR-526b-5p | NM_001111298 | PPIL6 |
| hsa-miR-526b-5p | NM_002912 | REV3L |
| hsa-miR-526b-5p | NM_001135648 | PTPRK |
| hsa-miR-526b-5p | NM_001130173 | MYB |
| hsa-miR-526b-5p | hsa_circ_0001642 | hsa_circ_001864 |
| hsa-miR-526b-5p | NM_001134831 | AHI1 |
| hsa-miR-526b-5p | NM_005923 | MAP3K5 |
| hsa-miR-526b-5p | NM_020464 | NHSL1 |
| hsa-miR-526b-5p | NM_031922 | REPS1 |
| hsa-miR-526b-5p | hsa_circ_0078031 | hsa_circ_0078031 |
| hsa-miR-526b-5p | NM_016485 | VTA1 |
| hsa-miR-526b-5p | NM_006734 | HIVEP2 |
| hsa-miR-526b-5p | NM_004690 | LATS1 |
| hsa-miR-526b-5p | NM_005100 | AKAP12 |
| hsa-miR-526b-5p | NM_182961 | SYNE1 |
| hsa-miR-526b-5p | NM_014892 | SCAF8 |
| hsa-miR-526b-5p | NM_012454 | TIAM2 |
| hsa-miR-526b-5p | NM_020245 | TULP4 |
| hsa-miR-526b-5p | NM_004906 | WTAP |
| hsa-miR-526b-5p | NM_000876 | IGF2R |
| hsa-miR-526b-5p | NM_001040000 | MLLT4 |
| hsa-miR-526b-5p | NM_003247 | THBS2 |
| hsa-miR-526b-5p | NM_032448 | FAM120B |
| hsa-miR-526b-5p | NM_144781 | PDCD2 |
| hsa-miR-526b-5p | NM_001134340 | PSMG3 |
| hsa-miR-526b-5p | NM_001080495 | TNRC18 |
| hsa-miR-526b-5p | TCONS_l2_00025633 | TCONS_l2_00025633 |
| hsa-miR-526b-5p | NM_207111 | RNF216 |
| hsa-miR-526b-5p | NM_018890 | RAC1 |
| hsa-miR-526b-5p | NM_139179 | DAGLB |
| hsa-miR-526b-5p | hsa_circ_0001674 | hsa_circ_000982 |
| hsa-miR-526b-5p | NM_024067 | C7orf26 |
| hsa-miR-526b-5p | NM_001159767 | BZW2 |
| hsa-miR-526b-5p | NM_015132 | SNX13 |
| hsa-miR-526b-5p | NM_000474 | TWIST1 |
| hsa-miR-526b-5p | NR_037598 | INMT-FAM188B |
| hsa-miR-526b-5p | NM_015060 | AVL9 |
| hsa-miR-526b-5p | hsa_circ_0007400 | hsa_circ_0007400 |
| hsa-miR-526b-5p | NM_018685 | ANLN |
| hsa-miR-526b-5p | NM_032016 | STARD3NL |
| hsa-miR-526b-5p | NM_004760 | STK17A |
| hsa-miR-526b-5p | NM_019082 | DDX56 |
| hsa-miR-526b-5p | NM_001165036 | OGDH |
| hsa-miR-526b-5p | hsa_circ_0001701 | hsa_circ_000980 |
| hsa-miR-526b-5p | NM_022748 | TNS3 |
| hsa-miR-526b-5p | NM_001042762 | FIGNL1 |
| hsa-miR-526b-5p | NR_027393 | INTS4L1 |
| hsa-miR-526b-5p | NR_033416 | CCT6P3 |
| hsa-miR-526b-5p | NM_152626 | ZNF92 |
| hsa-miR-526b-5p | hsa_circ_0001711 | hsa_circ_001947 |
| hsa-miR-526b-5p | TCONS_l2_00027391 | TCONS_l2_00027391 |
| hsa-miR-526b-5p | NM_153033 | KCTD7 |
| hsa-miR-526b-5p | NM_001145440 | TYW1B |
| hsa-miR-526b-5p | hsa_circ_0080464 | hsa_circ_0080464 |
| hsa-miR-526b-5p | hsa_circ_0080465 | hsa_circ_0080465 |
| hsa-miR-526b-5p | NM_003388 | CLIP2 |
| hsa-miR-526b-5p | NM_032999 | GTF2I |
| hsa-miR-526b-5p | NM_001168348 | NSUN5 |
| hsa-miR-526b-5p | NM_032408 | BAZ1B |
| hsa-miR-526b-5p | NR_002164 | GTF2IRD2P1 |
| hsa-miR-526b-5p | NR_040582 | STAG3L3 |
| hsa-miR-526b-5p | NM_002314 | LIMK1 |
| hsa-miR-526b-5p | NM_001099415 | POM121C |
| hsa-miR-526b-5p | NM_005338 | HIP1 |
| hsa-miR-526b-5p | NM_152754 | SEMA3D |
| hsa-miR-526b-5p | NM_006716 | DBF4 |
| hsa-miR-526b-5p | NM_012395 | CDK14 |
| hsa-miR-526b-5p | NM_005751 | AKAP9 |
| hsa-miR-526b-5p | NM_000786 | CYP51A1 |
| hsa-miR-526b-5p | NM_194455 | KRIT1 |
| hsa-miR-526b-5p | NM_022900 | CASD1 |
| hsa-miR-526b-5p | NM_001166160 | PPP1R9A |
| hsa-miR-526b-5p | NM_005720 | ARPC1B |
| hsa-miR-526b-5p | NM_145115 | ZNF498 |
| hsa-miR-526b-5p | NM_003439 | ZKSCAN1 |
| hsa-miR-526b-5p | NM_005273 | GNB2 |
| hsa-miR-526b-5p | NM_020246 | SLC12A9 |
| hsa-miR-526b-5p | NM_002803 | PSMC2 |
| hsa-miR-526b-5p | NM_005746 | NAMPT |
| hsa-miR-526b-5p | NM_014705 | DOCK4 |
| hsa-miR-526b-5p | NM_001127500 | MET |
| hsa-miR-526b-5p | NM_018077 | RBM28 |
| hsa-miR-526b-5p | TCONS_l2_00026196 | TCONS_l2_00026196 |
| hsa-miR-526b-5p | NM_001199672 | CALU |
| hsa-miR-526b-5p | NM_001458 | FLNC |
| hsa-miR-526b-5p | NR_034053 | TNPO3 |
| hsa-miR-526b-5p | NR_015431 | FLJ43663 |
| hsa-miR-526b-5p | hsa_circ_0001748 | hsa_circ_002039 |
| hsa-miR-526b-5p | NM_021807 | EXOC4 |
| hsa-miR-526b-5p | NM_001628 | AKR1B1 |
| hsa-miR-526b-5p | NM_015135 | NUP205 |
| hsa-miR-526b-5p | NM_024926 | TTC26 |
| hsa-miR-526b-5p | NM_016019 | LUC7L2 |
| hsa-miR-526b-5p | hsa_circ_0001755 | hsa_circ_001184 |
| hsa-miR-526b-5p | NM_022740 | HIPK2 |
| hsa-miR-526b-5p | NM_004333 | BRAF |
| hsa-miR-526b-5p | NM_001080392 | KIAA1147 |
| hsa-miR-526b-5p | NM_005435 | ARHGEF5 |
| hsa-miR-526b-5p | NM_001203247 | EZH2 |
| hsa-miR-526b-5p | NM_006712 | FASTK |
| hsa-miR-526b-5p | NM_170606 | MLL3 |
| hsa-miR-526b-5p | NM_018051 | WDR60 |
| hsa-miR-526b-5p | NM_015458 | MTMR9 |
| hsa-miR-526b-5p | NM_004462 | FDFT1 |
| hsa-miR-526b-5p | NM_182643 | DLC1 |
| hsa-miR-526b-5p | NM_013354 | CNOT7 |
| hsa-miR-526b-5p | NM_001001924 | MTUS1 |
| hsa-miR-526b-5p | NM_015310 | PSD3 |
| hsa-miR-526b-5p | NM_024815 | NUDT18 |
| hsa-miR-526b-5p | NM_016612 | SLC25A37 |
| hsa-miR-526b-5p | NM_004331 | BNIP3L |
| hsa-miR-526b-5p | NM_018660 | ZNF395 |
| hsa-miR-526b-5p | NM_001440 | EXTL3 |
| hsa-miR-526b-5p | NM_015344 | LEPROTL1 |
| hsa-miR-526b-5p | NM_000553 | WRN |
| hsa-miR-526b-5p | NM_001102559 | PPAPDC1B |
| hsa-miR-526b-5p | NM_023034 | WHSC1L1 |
| hsa-miR-526b-5p | NM_021623 | PLEKHA2 |
| hsa-miR-526b-5p | NM_001099412 | KAT6A |
| hsa-miR-526b-5p | NM_006749 | SLC20A2 |
| hsa-miR-526b-5p | NM_032410 | HOOK3 |
| hsa-miR-526b-5p | NM_001080394 | KIAA0146 |
| hsa-miR-526b-5p | NM_003350 | UBE2V2 |
| hsa-miR-526b-5p | NM_006756 | TCEA1 |
| hsa-miR-526b-5p | NM_006330 | LYPLA1 |
| hsa-miR-526b-5p | NM_024831 | TGS1 |
| hsa-miR-526b-5p | NM_017780 | CHD7 |
| hsa-miR-526b-5p | NM_152758 | YTHDF3 |
| hsa-miR-526b-5p | NM_014637 | MTFR1 |
| hsa-miR-526b-5p | NM_006421 | ARFGEF1 |
| hsa-miR-526b-5p | NM_024721 | ZFHX4 |
| hsa-miR-526b-5p | NM_015496 | KIAA1429 |
| hsa-miR-526b-5p | NM_181787 | DPY19L4 |
| hsa-miR-526b-5p | NM_001145860 | POP1 |
| hsa-miR-526b-5p | NM_006281 | STK3 |
| hsa-miR-526b-5p | NM_017890 | VPS13B |
| hsa-miR-526b-5p | NM_002568 | PABPC1 |
| hsa-miR-526b-5p | NM_015902 | UBR5 |
| hsa-miR-526b-5p | NM_001568 | EIF3E |
| hsa-miR-526b-5p | NM_014112 | TRPS1 |
| hsa-miR-526b-5p | NM_001204180 | ZHX1-C8ORF76 |
| hsa-miR-526b-5p | NM_032847 | C8orf76 |
| hsa-miR-526b-5p | NM_014109 | ATAD2 |
| hsa-miR-526b-5p | NM_002467 | MYC |
| hsa-miR-526b-5p | NR_003367 | PVT1 |
| hsa-miR-526b-5p | TCONS_00015354 | TCONS_00015354 |
| hsa-miR-526b-5p | hsa_circ_0001821 | hsa_circ_000006 |
| hsa-miR-526b-5p | NM_001247996 | ASAP1 |
| hsa-miR-526b-5p | NM_016018 | PHF20L1 |
| hsa-miR-526b-5p | NM_001029939 | ZFAT |
| hsa-miR-526b-5p | NM_001199649 | PTK2 |
| hsa-miR-526b-5p | NM_078480 | PUF60 |
| hsa-miR-526b-5p | NM_201380 | PLEC |
| hsa-miR-526b-5p | NM_138496 | CYHR1 |
| hsa-miR-526b-5p | NM_004260 | RECQL4 |
| hsa-miR-526b-5p | NM_153186 | KANK1 |
| hsa-miR-526b-5p | NM_002919 | RFX3 |
| hsa-miR-526b-5p | NM_001042413 | GLIS3 |
| hsa-miR-526b-5p | NM_033222 | PSIP1 |
| hsa-miR-526b-5p | NM_017738 | CNTLN |
| hsa-miR-526b-5p | NM_017925 | DENND4C |
| hsa-miR-526b-5p | NM_002197 | ACO1 |
| hsa-miR-526b-5p | NM_001497 | B4GALT1 |
| hsa-miR-526b-5p | NM_001244752 | ANKRD18B |
| hsa-miR-526b-5p | NM_006289 | TLN1 |
| hsa-miR-526b-5p | NM_005476 | GNE |
| hsa-miR-526b-5p | NM_032226 | ZCCHC7 |
| hsa-miR-526b-5p | NM_003028 | SHB |
| hsa-miR-526b-5p | NM_000692 | ALDH1B1 |
| hsa-miR-526b-5p | NM_147195 | ANKRD18A |
| hsa-miR-526b-5p | NM_004817 | TJP2 |
| hsa-miR-526b-5p | NM_015225 | PRUNE2 |
| hsa-miR-526b-5p | NM_007005 | TLE4 |
| hsa-miR-526b-5p | NM_005226 | S1PR3 |
| hsa-miR-526b-5p | NM_024077 | SECISBP2 |
| hsa-miR-526b-5p | NM_006415 | SPTLC1 |
| hsa-miR-526b-5p | NR_024020 | NOL8 |
| hsa-miR-526b-5p | NM_000136 | FANCC |
| hsa-miR-526b-5p | NM_001010895 | C9orf102 |
| hsa-miR-526b-5p | NM_007001 | SLC35D2 |
| hsa-miR-526b-5p | NM_018946 | NANS |
| hsa-miR-526b-5p | NM_017919 | STX17 |
| hsa-miR-526b-5p | hsa_circ_0001876 | hsa_circ_000492 |
| hsa-miR-526b-5p | NM_001042550 | SMC2 |
| hsa-miR-526b-5p | NM_005502 | ABCA1 |
| hsa-miR-526b-5p | NM_001079802 | FKTN |
| hsa-miR-526b-5p | NM_001136562 | AKAP2 |
| hsa-miR-526b-5p | NM_007203 | PALM2-AKAP2 |
| hsa-miR-526b-5p | NM_015258 | FKBP15 |
| hsa-miR-526b-5p | NM_001859 | SLC31A1 |
| hsa-miR-526b-5p | NM_002581 | PAPPA |
| hsa-miR-526b-5p | NM_005047 | PSMD5 |
| hsa-miR-526b-5p | NM_001127663 | GSN |
| hsa-miR-526b-5p | NM_014222 | NDUFA8 |
| hsa-miR-526b-5p | NM_015635 | GAPVD1 |
| hsa-miR-526b-5p | NM_022833 | FAM129B |
| hsa-miR-526b-5p | NM_000118 | ENG |
| hsa-miR-526b-5p | NR_027811 | PTGES2 |
| hsa-miR-526b-5p | NM_001130438 | SPTAN1 |
| hsa-miR-526b-5p | NM_052844 | WDR34 |
| hsa-miR-526b-5p | NM_001127244 | LRRC8A |
| hsa-miR-526b-5p | NM_015354 | NUP188 |
| hsa-miR-526b-5p | NM_015033 | FNBP1 |
| hsa-miR-526b-5p | NM_001136557 | GPR107 |
| hsa-miR-526b-5p | NM_198679 | RAPGEF1 |
| hsa-miR-526b-5p | NM_015046 | SETX |
| hsa-miR-526b-5p | NM_014811 | PPP1R26 |
| hsa-miR-526b-5p | NM_018998 | FBXW5 |
| hsa-miR-526b-5p | NM_003731 | SSNA1 |
| hsa-miR-526b-5p | NM_001128228 | TPRN |
| hsa-miR-526b-5p | NM_001130969 | NELF |
| hsa-miR-526b-5p | NM_014974 | DIP2C |
| hsa-miR-526b-5p | NM_004508 | IDI1 |
| hsa-miR-526b-5p | NM_014023 | WDR37 |
| hsa-miR-526b-5p | NM_001242307 | PITRM1 |
| hsa-miR-526b-5p | NM_017782 | FAM208B |
| hsa-miR-526b-5p | NM_001494 | GDI2 |
| hsa-miR-526b-5p | NM_080599 | UPF2 |
| hsa-miR-526b-5p | hsa_circ_0000213 | hsa_circ_001550 |
| hsa-miR-526b-5p | NM_006023 | CDC123 |
| hsa-miR-526b-5p | NM_012247 | SEPHS1 |
| hsa-miR-526b-5p | NM_020824 | ARHGAP21 |
| hsa-miR-526b-5p | NM_021738 | SVIL |
| hsa-miR-526b-5p | NM_020848 | KIAA1462 |
| hsa-miR-526b-5p | NM_018109 | MTPAP |
| hsa-miR-526b-5p | NM_004521 | KIF5B |
| hsa-miR-526b-5p | NM_001184785 | PARD3 |
| hsa-miR-526b-5p | NM_001007094 | ZNF37A |
| hsa-miR-526b-5p | NM_002750 | MAPK8 |
| hsa-miR-526b-5p | NM_000124 | ERCC6 |
| hsa-miR-526b-5p | NM_001145260 | NCOA4 |
| hsa-miR-526b-5p | NR_024555 | RHOBTB1 |
| hsa-miR-526b-5p | NM_030759 | NRBF2 |
| hsa-miR-526b-5p | NM_022079 | HERC4 |
| hsa-miR-526b-5p | NM_001080449 | DNA2 |
| hsa-miR-526b-5p | NM_030625 | TET1 |
| hsa-miR-526b-5p | NM_018237 | CCAR1 |
| hsa-miR-526b-5p | NM_004728 | DDX21 |
| hsa-miR-526b-5p | NM_001142648 | SAR1A |
| hsa-miR-526b-5p | NM_018239 | LRRC20 |
| hsa-miR-526b-5p | NM_004273 | CHST3 |
| hsa-miR-526b-5p | NM_006077 | MICU1 |
| hsa-miR-526b-5p | NM_152635 | OIT3 |
| hsa-miR-526b-5p | NM_015037 | KIAA0913 |
| hsa-miR-526b-5p | hsa_circ_0000249 | hsa_circ_001838 |
| hsa-miR-526b-5p | NM_007055 | POLR3A |
| hsa-miR-526b-5p | NM_001142285 | RPS24 |
| hsa-miR-526b-5p | NM_020338 | ZMIZ1 |
| hsa-miR-526b-5p | NM_014394 | GHITM |
| hsa-miR-526b-5p | NM_018999 | FAM190B |
| hsa-miR-526b-5p | NM_032373 | PCGF5 |
| hsa-miR-526b-5p | NM_003972 | BTAF1 |
| hsa-miR-526b-5p | NM_017824 | 5-Mar |
| hsa-miR-526b-5p | NM_004523 | KIF11 |
| hsa-miR-526b-5p | NM_013451 | MYOF |
| hsa-miR-526b-5p | NM_152309 | PIK3AP1 |
| hsa-miR-526b-5p | NM_032440 | LCOR |
| hsa-miR-526b-5p | NM_138413 | HOGA1 |
| hsa-miR-526b-5p | NM_021732 | AVPI1 |
| hsa-miR-526b-5p | NM_031484 | MARVELD1 |
| hsa-miR-526b-5p | NM_015221 | DNMBP |
| hsa-miR-526b-5p | NM_001278 | CHUK |
| hsa-miR-526b-5p | NM_005063 | SCD |
| hsa-miR-526b-5p | NM_017902 | HIF1AN |
| hsa-miR-526b-5p | NM_001203244 | SEMA4G |
| hsa-miR-526b-5p | NM_001163812 | C10orf2 |
| hsa-miR-526b-5p | NM_012215 | MGEA5 |
| hsa-miR-526b-5p | NM_003893 | LDB1 |
| hsa-miR-526b-5p | NM_024326 | FBXL15 |
| hsa-miR-526b-5p | NM_014976 | PDCD11 |
| hsa-miR-526b-5p | NM_033397 | ITPRIP |
| hsa-miR-526b-5p | NM_001244949 | GPAM |
| hsa-miR-526b-5p | NM_001127211 | KIAA1598 |
| hsa-miR-526b-5p | NM_014904 | RAB11FIP2 |
| hsa-miR-526b-5p | NM_022063 | FAM204A |
| hsa-miR-526b-5p | NM_153810 | C10orf46 |
| hsa-miR-526b-5p | NM_003750 | EIF3A |
| hsa-miR-526b-5p | NM_001005339 | RGS10 |
| hsa-miR-526b-5p | NM_001033925 | TIAL1 |
| hsa-miR-526b-5p | NM_018117 | WDR11 |
| hsa-miR-526b-5p | NM_001195608 | PLEKHA1 |
| hsa-miR-526b-5p | NM_002775 | HTRA1 |
| hsa-miR-526b-5p | NM_014661 | FAM53B |
| hsa-miR-526b-5p | NM_017580 | ZRANB1 |
| hsa-miR-526b-5p | hsa_circ_0000268 | hsa_circ_000002 |
| hsa-miR-526b-5p | NM_001083914 | CTBP2 |
| hsa-miR-526b-5p | NM_001380 | DOCK1 |
| hsa-miR-526b-5p | NM_002417 | MKI67 |
| hsa-miR-526b-5p | NM_001199868 | GLRX3 |
| hsa-miR-526b-5p | NM_016526 | BET1L |
| hsa-miR-526b-5p | NM_004357 | CD151 |
| hsa-miR-526b-5p | NM_001909 | CTSD |
| hsa-miR-526b-5p | NM_004356 | CD81 |
| hsa-miR-526b-5p | NM_001194997 | CARS |
| hsa-miR-526b-5p | NM_016320 | NUP98 |
| hsa-miR-526b-5p | NM_001033 | RRM1 |
| hsa-miR-526b-5p | NM_005418 | ST5 |
| hsa-miR-526b-5p | NM_015213 | DENND5A |
| hsa-miR-526b-5p | NM_003442 | ZNF143 |
| hsa-miR-526b-5p | NM_030962 | SBF2 |
| hsa-miR-526b-5p | NM_001145811 | SOX6 |
| hsa-miR-526b-5p | NM_001142307 | GTF2H1 |
| hsa-miR-526b-5p | NM_006292 | TSG101 |
| hsa-miR-526b-5p | NM_194285 | SPTY2D1 |
| hsa-miR-526b-5p | NM_031217 | KIF18A |
| hsa-miR-526b-5p | NM_024081 | PRRG4 |
| hsa-miR-526b-5p | NM_001076786 | QSER1 |
| hsa-miR-526b-5p | NM_001326 | CSTF3 |
| hsa-miR-526b-5p | NM_024662 | NAT10 |
| hsa-miR-526b-5p | NM_174902 | LDLRAD3 |
| hsa-miR-526b-5p | NM_018259 | TTC17 |
| hsa-miR-526b-5p | NM_001142673 | ATG13 |
| hsa-miR-526b-5p | NM_004308 | ARHGAP1 |
| hsa-miR-526b-5p | NM_002804 | PSMC3 |
| hsa-miR-526b-5p | NM_175732 | PTPMT1 |
| hsa-miR-526b-5p | NM_002843 | PTPRJ |
| hsa-miR-526b-5p | NM_001085458 | CTNND1 |
| hsa-miR-526b-5p | NM_001923 | DDB1 |
| hsa-miR-526b-5p | NM_006133 | DAGLA |
| hsa-miR-526b-5p | NM_022830 | TUT1 |
| hsa-miR-526b-5p | NM_024099 | C11orf48 |
| hsa-miR-526b-5p | NM_001039469 | MARK2 |
| hsa-miR-526b-5p | NM_001198868 | CAPN1 |
| hsa-miR-526b-5p | NM_006779 | CDC42EP2 |
| hsa-miR-526b-5p | NM_031904 | FRMD8 |
| hsa-miR-526b-5p | NM_002419 | MAP3K11 |
| hsa-miR-526b-5p | NM_018026 | PACS1 |
| hsa-miR-526b-5p | NM_030981 | RAB1B |
| hsa-miR-526b-5p | NM_015399 | BRMS1 |
| hsa-miR-526b-5p | NM_006946 | SPTBN2 |
| hsa-miR-526b-5p | NM_012308 | KDM2A |
| hsa-miR-526b-5p | NM_002708 | PPP1CA |
| hsa-miR-526b-5p | NM_003977 | AIP |
| hsa-miR-526b-5p | NM_030930 | UNC93B1 |
| hsa-miR-526b-5p | NM_001164160 | PPP6R3 |
| hsa-miR-526b-5p | NM_001876 | CPT1A |
| hsa-miR-526b-5p | NM_003626 | PPFIA1 |
| hsa-miR-526b-5p | NR_024148 | RNF121 |
| hsa-miR-526b-5p | NM_025155 | PAAF1 |
| hsa-miR-526b-5p | NM_015531 | C2CD3 |
| hsa-miR-526b-5p | NM_006591 | POLD3 |
| hsa-miR-526b-5p | NM_004041 | ARRB1 |
| hsa-miR-526b-5p | NM_001005 | RPS3 |
| hsa-miR-526b-5p | NM_018367 | ACER3 |
| hsa-miR-526b-5p | NM_001128620 | PAK1 |
| hsa-miR-526b-5p | NM_024684 | C11orf67 |
| hsa-miR-526b-5p | NM_033547 | INTS4 |
| hsa-miR-526b-5p | NM_130847 | AMOTL1 |
| hsa-miR-526b-5p | hsa_circ_0000350 | hsa_circ_001723 |
| hsa-miR-526b-5p | NM_144665 | SESN3 |
| hsa-miR-526b-5p | hsa_circ_0000353 | hsa_circ_001720 |
| hsa-miR-526b-5p | NM_001145938 | MMP1 |
| hsa-miR-526b-5p | NM_000051 | ATM |
| hsa-miR-526b-5p | hsa_circ_0000356 | hsa_circ_001219 |
| hsa-miR-526b-5p | NM_006169 | NNMT |
| hsa-miR-526b-5p | NM_001197104 | MLL |
| hsa-miR-526b-5p | NM_001655 | ARCN1 |
| hsa-miR-526b-5p | NM_016146 | TRAPPC4 |
| hsa-miR-526b-5p | NM_001243259 | HINFP |
| hsa-miR-526b-5p | NM_003105 | SORL1 |
| hsa-miR-526b-5p | NM_032873 | UBASH3B |
| hsa-miR-526b-5p | NM_020228 | PRDM10 |
| hsa-miR-526b-5p | NM_152640 | DCP1B |
| hsa-miR-526b-5p | NM_002014 | FKBP4 |
| hsa-miR-526b-5p | NM_001759 | CCND2 |
| hsa-miR-526b-5p | NM_001065 | TNFRSF1A |
| hsa-miR-526b-5p | NM_014865 | NCAPD2 |
| hsa-miR-526b-5p | NM_002046 | GAPDH |
| hsa-miR-526b-5p | NM_001033714 | NOP2 |
| hsa-miR-526b-5p | NM_001273 | CHD4 |
| hsa-miR-526b-5p | NM_001144831 | PHB2 |
| hsa-miR-526b-5p | NM_020734 | RIMKLB |
| hsa-miR-526b-5p | NM_000014 | A2M |
| hsa-miR-526b-5p | NR_037918 | PRH1-PRR4 |
| hsa-miR-526b-5p | NM_176884 | TAS2R43 |
| hsa-miR-526b-5p | hsa_circ_0000376 | hsa_circ_000554 |
| hsa-miR-526b-5p | NM_003979 | GPRC5A |
| hsa-miR-526b-5p | NM_004447 | EPS8 |
| hsa-miR-526b-5p | NM_001174097 | LDHB |
| hsa-miR-526b-5p | NM_014802 | KIAA0528 |
| hsa-miR-526b-5p | NM_001164747 | RASSF8 |
| hsa-miR-526b-5p | NM_002223 | ITPR2 |
| hsa-miR-526b-5p | NM_016551 | TM7SF3 |
| hsa-miR-526b-5p | NM_177444 | PPFIBP1 |
| hsa-miR-526b-5p | NM_018099 | FAR2 |
| hsa-miR-526b-5p | NM_175861 | TMTC1 |
| hsa-miR-526b-5p | NM_032156 | CAPRIN2 |
| hsa-miR-526b-5p | NM_144973 | DENND5B |
| hsa-miR-526b-5p | NM_201515 | PPHLN1 |
| hsa-miR-526b-5p | NM_004719 | SCAF11 |
| hsa-miR-526b-5p | hsa_circ_0000395 | hsa_circ_001046 |
| hsa-miR-526b-5p | NM_024604 | RPAP3 |
| hsa-miR-526b-5p | NM_001659 | ARF3 |
| hsa-miR-526b-5p | NM_003482 | MLL2 |
| hsa-miR-526b-5p | NM_023071 | SPATS2 |
| hsa-miR-526b-5p | NM_001126103 | RACGAP1 |
| hsa-miR-526b-5p | NR_045072 | CSRNP2 |
| hsa-miR-526b-5p | NM_173086 | KRT6C |
| hsa-miR-526b-5p | NM_015319 | TENC1 |
| hsa-miR-526b-5p | NM_001170790 | MFSD5 |
| hsa-miR-526b-5p | NM_002624 | PFDN5 |
| hsa-miR-526b-5p | NM_018457 | PRR13 |
| hsa-miR-526b-5p | NM_001130059 | ATF7 |
| hsa-miR-526b-5p | NM_001127322 | CBX5 |
| hsa-miR-526b-5p | NM_032364 | DNAJC14 |
| hsa-miR-526b-5p | NM_201444 | DGKA |
| hsa-miR-526b-5p | NM_001982 | ERBB3 |
| hsa-miR-526b-5p | NM_173595 | ANKRD52 |
| hsa-miR-526b-5p | NM_004077 | CS |
| hsa-miR-526b-5p | NM_005419 | STAT2 |
| hsa-miR-526b-5p | NM_002332 | LRP1 |
| hsa-miR-526b-5p | NM_014925 | R3HDM2 |
| hsa-miR-526b-5p | NM_182947 | ARHGEF25 |
| hsa-miR-526b-5p | NM_000075 | CDK4 |
| hsa-miR-526b-5p | NM_015026 | MON2 |
| hsa-miR-526b-5p | NM_020762 | SRGAP1 |
| hsa-miR-526b-5p | NM_007235 | XPOT |
| hsa-miR-526b-5p | NM_003483 | HMGA2 |
| hsa-miR-526b-5p | NM_020401 | NUP107 |
| hsa-miR-526b-5p | NM_001042555 | FRS2 |
| hsa-miR-526b-5p | NM_175623 | RAB3IP |
| hsa-miR-526b-5p | NM_014515 | CNOT2 |
| hsa-miR-526b-5p | NM_007350 | PHLDA1 |
| hsa-miR-526b-5p | NM_152588 | TMTC2 |
| hsa-miR-526b-5p | NM_001920 | DCN |
| hsa-miR-526b-5p | NM_213611 | SLC25A3 |
| hsa-miR-526b-5p | NM_181861 | APAF1 |
| hsa-miR-526b-5p | NM_014503 | UTP20 |
| hsa-miR-526b-5p | NM_024057 | NUP37 |
| hsa-miR-526b-5p | NM_017915 | C12orf48 |
| hsa-miR-526b-5p | NM_001031701 | NT5DC3 |
| hsa-miR-526b-5p | NM_032148 | SLC41A2 |
| hsa-miR-526b-5p | NM_012406 | PRDM4 |
| hsa-miR-526b-5p | NM_014055 | IFT81 |
| hsa-miR-526b-5p | NM_001137664 | ANAPC7 |
| hsa-miR-526b-5p | NM_015267 | CUX2 |
| hsa-miR-526b-5p | NM_000690 | ALDH2 |
| hsa-miR-526b-5p | NM_001143906 | TRAFD1 |
| hsa-miR-526b-5p | NM_006187 | OAS3 |
| hsa-miR-526b-5p | NM_001111322 | DDX54 |
| hsa-miR-526b-5p | NM_015335 | MED13L |
| hsa-miR-526b-5p | NM_019086 | VSIG10 |
| hsa-miR-526b-5p | NM_016281 | TAOK3 |
| hsa-miR-526b-5p | NM_006836 | GCN1L1 |
| hsa-miR-526b-5p | NM_014868 | RNF10 |
| hsa-miR-526b-5p | NM_001080825 | TMEM120B |
| hsa-miR-526b-5p | NM_001247997 | CLIP1 |
| hsa-miR-526b-5p | NR_036435 | RSRC2 |
| hsa-miR-526b-5p | NM_023928 | AACS |
| hsa-miR-526b-5p | NM_025215 | PUS1 |
| hsa-miR-526b-5p | NM_006231 | POLE |
| hsa-miR-526b-5p | NM_001161344 | CHFR |
| hsa-miR-526b-5p | TCONS_l2_00007055 | TCONS_l2_00007055 |
| hsa-miR-526b-5p | NM_001042414 | PSPC1 |
| hsa-miR-526b-5p | hsa_circ_0000464 | hsa_circ_000830 |
| hsa-miR-526b-5p | NM_153251 | ZDHHC20 |
| hsa-miR-526b-5p | NM_014363 | SACS |
| hsa-miR-526b-5p | NM_175854 | PAN3 |
| hsa-miR-526b-5p | NM_006644 | HSPH1 |
| hsa-miR-526b-5p | NM_000059 | BRCA2 |
| hsa-miR-526b-5p | NM_014887 | N4BP2L2 |
| hsa-miR-526b-5p | NM_006475 | POSTN |
| hsa-miR-526b-5p | NM_015058 | KIAA0564 |
| hsa-miR-526b-5p | NM_016248 | AKAP11 |
| hsa-miR-526b-5p | NM_018559 | KIAA1704 |
| hsa-miR-526b-5p | NM_031431 | COG3 |
| hsa-miR-526b-5p | NM_015070 | ZC3H13 |
| hsa-miR-526b-5p | NM_018191 | RCBTB1 |
| hsa-miR-526b-5p | hsa_circ_0000484 | hsa_circ_000160 |
| hsa-miR-526b-5p | NM_213590 | TRIM13 |
| hsa-miR-526b-5p | NM_012141 | INTS6 |
| hsa-miR-526b-5p | NR_002793 | TPTE2P3 |
| hsa-miR-526b-5p | hsa_circ_0000492 | hsa_circ_001561 |
| hsa-miR-526b-5p | NM_005358 | LMO7 |
| hsa-miR-526b-5p | NM_024546 | RNF219 |
| hsa-miR-526b-5p | NM_020121 | UGGT2 |
| hsa-miR-526b-5p | NM_005766 | FARP1 |
| hsa-miR-526b-5p | NM_000282 | PCCA |
| hsa-miR-526b-5p | hsa_circ_0000500 | hsa_circ_001876 |
| hsa-miR-526b-5p | NM_003749 | IRS2 |
| hsa-miR-526b-5p | NM_015205 | ATP11A |
| hsa-miR-526b-5p | NM_001143946 | GAS6 |
| hsa-miR-526b-5p | NM_007192 | SUPT16H |
| hsa-miR-526b-5p | NM_001170629 | CHD8 |
| hsa-miR-526b-5p | NM_019852 | METTL3 |
| hsa-miR-526b-5p | hsa_circ_0000523 | hsa_circ_000169 |
| hsa-miR-526b-5p | NM_005407 | SALL2 |
| hsa-miR-526b-5p | NM_001039619 | PRMT5 |
| hsa-miR-526b-5p | NM_001166269 | HAUS4 |
| hsa-miR-526b-5p | NM_017924 | C14orf119 |
| hsa-miR-526b-5p | NM_176783 | PSME1 |
| hsa-miR-526b-5p | NM_024658 | IPO4 |
| hsa-miR-526b-5p | NM_012461 | TINF2 |
| hsa-miR-526b-5p | NM_004581 | RABGGTA |
| hsa-miR-526b-5p | NM_014178 | STXBP6 |
| hsa-miR-526b-5p | NM_015382 | HECTD1 |
| hsa-miR-526b-5p | NM_015473 | HEATR5A |
| hsa-miR-526b-5p | NM_138288 | SPTSSA |
| hsa-miR-526b-5p | NM_013448 | BAZ1A |
| hsa-miR-526b-5p | NM_014990 | RALGAPA1 |
| hsa-miR-526b-5p | NM_001195296 | MIPOL1 |
| hsa-miR-526b-5p | NM_015091 | FAM179B |
| hsa-miR-526b-5p | NM_002408 | MGAT2 |
| hsa-miR-526b-5p | NM_014315 | KLHDC2 |
| hsa-miR-526b-5p | NM_004713 | NEMF |
| hsa-miR-526b-5p | NM_015589 | SAMD4A |
| hsa-miR-526b-5p | NM_199421 | SOCS4 |
| hsa-miR-526b-5p | NM_182926 | KTN1 |
| hsa-miR-526b-5p | NM_002892 | ARID4A |
| hsa-miR-526b-5p | NM_001244189 | KIAA0586 |
| hsa-miR-526b-5p | NM_021003 | PPM1A |
| hsa-miR-526b-5p | NM_182914 | SYNE2 |
| hsa-miR-526b-5p | NM_005956 | MTHFD1 |
| hsa-miR-526b-5p | NM_001202559 | CHURC1-FNTB |
| hsa-miR-526b-5p | NM_020806 | GPHN |
| hsa-miR-526b-5p | hsa_circ_0000547 | hsa_circ_001250 |
| hsa-miR-526b-5p | NM_001130004 | ACTN1 |
| hsa-miR-526b-5p | NM_004450 | ERH |
| hsa-miR-526b-5p | NM_001039465 | SRSF5 |
| hsa-miR-526b-5p | NM_005466 | MED6 |
| hsa-miR-526b-5p | NM_014982 | PCNX |
| hsa-miR-526b-5p | NM_021260 | ZFYVE1 |
| hsa-miR-526b-5p | NM_001242924 | ZNF410 |
| hsa-miR-526b-5p | NM_152444 | PTGR2 |
| hsa-miR-526b-5p | NM_001249 | ENTPD5 |
| hsa-miR-526b-5p | NM_014239 | EIF2B2 |
| hsa-miR-526b-5p | NM_015305 | ANGEL1 |
| hsa-miR-526b-5p | NM_004863 | SPTLC2 |
| hsa-miR-526b-5p | NM_005065 | SEL1L |
| hsa-miR-526b-5p | NM_024824 | ZC3H14 |
| hsa-miR-526b-5p | NM_004755 | RPS6KA5 |
| hsa-miR-526b-5p | NM_004239 | TRIP11 |
| hsa-miR-526b-5p | NM_005113 | GOLGA5 |
| hsa-miR-526b-5p | NM_014216 | ITPK1 |
| hsa-miR-526b-5p | NM_175748 | UBR7 |
| hsa-miR-526b-5p | NM_020414 | DDX24 |
| hsa-miR-526b-5p | NM_004184 | WARS |
| hsa-miR-526b-5p | NM_001242414 | WDR20 |
| hsa-miR-526b-5p | NM_015156 | RCOR1 |
| hsa-miR-526b-5p | NM_006035 | CDC42BPB |
| hsa-miR-526b-5p | NM_001823 | CKB |
| hsa-miR-526b-5p | NM_032374 | APOPT1 |
| hsa-miR-526b-5p | NM_001014432 | AKT1 |
| hsa-miR-526b-5p | NM_138420 | AHNAK2 |
| hsa-miR-526b-5p | NR_002224 | ADAM6 |
| hsa-miR-526b-5p | NR_027457 | LINC00221 |
| hsa-miR-526b-5p | NR_033375 | LINC00226 |
| hsa-miR-526b-5p | NM_000462 | UBE3A |
| hsa-miR-526b-5p | NM_003257 | TJP1 |
| hsa-miR-526b-5p | NM_001191323 | GREM1 |
| hsa-miR-526b-5p | NM_003246 | THBS1 |
| hsa-miR-526b-5p | NM_020857 | VPS18 |
| hsa-miR-526b-5p | NM_017553 | INO80 |
| hsa-miR-526b-5p | NM_016359 | NUSAP1 |
| hsa-miR-526b-5p | NM_001164273 | MGA |
| hsa-miR-526b-5p | NM_153260 | LRRC57 |
| hsa-miR-526b-5p | NM_174916 | UBR1 |
| hsa-miR-526b-5p | NM_002373 | MAP1A |
| hsa-miR-526b-5p | NM_020990 | CKMT1B |
| hsa-miR-526b-5p | NM_025137 | SPG11 |
| hsa-miR-526b-5p | NM_021199 | SQRDL |
| hsa-miR-526b-5p | NM_001001556 | GALK2 |
| hsa-miR-526b-5p | NM_020234 | DTWD1 |
| hsa-miR-526b-5p | NM_017672 | TRPM7 |
| hsa-miR-526b-5p | NM_001252127 | AP4E1 |
| hsa-miR-526b-5p | NM_014547 | TMOD3 |
| hsa-miR-526b-5p | NM_006628 | ARPP19 |
| hsa-miR-526b-5p | NM_019600 | FAM214A |
| hsa-miR-526b-5p | NM_001110 | ADAM10 |
| hsa-miR-526b-5p | NM_001018088 | VPS13C |
| hsa-miR-526b-5p | hsa_circ_0000607 | hsa_circ_001567 |
| hsa-miR-526b-5p | NM_001218 | CA12 |
| hsa-miR-526b-5p | NM_003099 | SNX1 |
| hsa-miR-526b-5p | NM_001144823 | DENND4A |
| hsa-miR-526b-5p | NM_000968 | RPL4 |
| hsa-miR-526b-5p | NM_005902 | SMAD3 |
| hsa-miR-526b-5p | NM_024666 | AAGAB |
| hsa-miR-526b-5p | hsa_circ_0000620 | hsa_circ_000603 |
| hsa-miR-526b-5p | NM_006305 | ANP32A |
| hsa-miR-526b-5p | NM_138555 | KIF23 |
| hsa-miR-526b-5p | NM_001199017 | LRRC49 |
| hsa-miR-526b-5p | NM_005744 | ARIH1 |
| hsa-miR-526b-5p | hsa_circ_0000627 | hsa_circ_001066 |
| hsa-miR-526b-5p | NM_005545 | ISLR |
| hsa-miR-526b-5p | NM_024776 | PEAK1 |
| hsa-miR-526b-5p | NM_001242913 | ZFAND6 |
| hsa-miR-526b-5p | hsa_circ_0000642 | hsa_circ_002031 |
| hsa-miR-526b-5p | hsa_circ_0000643 | hsa_circ_001681 |
| hsa-miR-526b-5p | NM_024580 | EFTUD1 |
| hsa-miR-526b-5p | NM_006738 | AKAP13 |
| hsa-miR-526b-5p | NM_005928 | MFGE8 |
| hsa-miR-526b-5p | NM_001113378 | FANCI |
| hsa-miR-526b-5p | NM_198526 | ZNF710 |
| hsa-miR-526b-5p | NM_001029964 | TTLL13 |
| hsa-miR-526b-5p | NM_022769 | CRTC3 |
| hsa-miR-526b-5p | NM_021259 | TMEM8A |
| hsa-miR-526b-5p | hsa_circ_0000663 | hsa_circ_001482 |
| hsa-miR-526b-5p | NM_016111 | TELO2 |
| hsa-miR-526b-5p | NM_004424 | E4F1 |
| hsa-miR-526b-5p | NM_016333 | SRRM2 |
| hsa-miR-526b-5p | NM_015041 | CLUAP1 |
| hsa-miR-526b-5p | NM_014153 | ZC3H7A |
| hsa-miR-526b-5p | NM_001130007 | GSPT1 |
| hsa-miR-526b-5p | NM_016561 | BFAR |
| hsa-miR-526b-5p | NM_004996 | ABCC1 |
| hsa-miR-526b-5p | NM_006985 | NPIP |
| hsa-miR-526b-5p | NM_014287 | NOMO1 |
| hsa-miR-526b-5p | hsa_circ_0038026 | hsa_circ_0038026 |
| hsa-miR-526b-5p | hsa_circ_0038027 | hsa_circ_0038027 |
| hsa-miR-526b-5p | hsa_circ_0038028 | hsa_circ_0038028 |
| hsa-miR-526b-5p | hsa_circ_0038029 | hsa_circ_0038029 |
| hsa-miR-526b-5p | NM_033201 | C16orf45 |
| hsa-miR-526b-5p | NM_001184998 | KIAA0430 |
| hsa-miR-526b-5p | NM_001004067 | NOMO3 |
| hsa-miR-526b-5p | NR_036447 | PKD1P1 |
| hsa-miR-526b-5p | hsa_circ_0038208 | hsa_circ_0038208 |
| hsa-miR-526b-5p | hsa_circ_0038209 | hsa_circ_0038209 |
| hsa-miR-526b-5p | NM_173614 | NOMO2 |
| hsa-miR-526b-5p | NM_001019 | RPS15A |
| hsa-miR-526b-5p | NM_015092 | SMG1 |
| hsa-miR-526b-5p | NM_001034841 | ITPRIPL2 |
| hsa-miR-526b-5p | NM_153208 | IQCK |
| hsa-miR-526b-5p | NM_130464 | NPIPL3 |
| hsa-miR-526b-5p | NR_027155 | LOC100271836 |
| hsa-miR-526b-5p | NR_002594 | SLC7A5P2 |
| hsa-miR-526b-5p | hsa_circ_0038445 | hsa_circ_0038445 |
| hsa-miR-526b-5p | hsa_circ_0038450 | hsa_circ_0038450 |
| hsa-miR-526b-5p | NM_018119 | POLR3E |
| hsa-miR-526b-5p | NR_027154 | LOC641298 |
| hsa-miR-526b-5p | NM_020718 | USP31 |
| hsa-miR-526b-5p | NM_014494 | TNRC6A |
| hsa-miR-526b-5p | NM_001199142 | EIF3C |
| hsa-miR-526b-5p | NM_001145812 | SH2B1 |
| hsa-miR-526b-5p | NR_002453 | LOC595101 |
| hsa-miR-526b-5p | hsa_circ_0004082 | hsa_circ_0004082 |
| hsa-miR-526b-5p | hsa_circ_0038892 | hsa_circ_0038892 |
| hsa-miR-526b-5p | hsa_circ_0038893 | hsa_circ_0038893 |
| hsa-miR-526b-5p | hsa_circ_0038894 | hsa_circ_0038894 |
| hsa-miR-526b-5p | hsa_circ_0038895 | hsa_circ_0038895 |
| hsa-miR-526b-5p | hsa_circ_0038896 | hsa_circ_0038896 |
| hsa-miR-526b-5p | hsa_circ_0038907 | hsa_circ_0038907 |
| hsa-miR-526b-5p | hsa_circ_0038908 | hsa_circ_0038908 |
| hsa-miR-526b-5p | hsa_circ_0038909 | hsa_circ_0038909 |
| hsa-miR-526b-5p | NM_001031827 | BOLA2 |
| hsa-miR-526b-5p | TCONS_l2_00010452 | TCONS_l2_00010452 |
| hsa-miR-526b-5p | NM_006662 | SRCAP |
| hsa-miR-526b-5p | NM_014771 | RNF40 |
| hsa-miR-526b-5p | NM_024706 | ZNF668 |
| hsa-miR-526b-5p | NM_001122957 | BCKDK |
| hsa-miR-526b-5p | NM_013258 | PYCARD |
| hsa-miR-526b-5p | NM_003414 | ZNF267 |
| hsa-miR-526b-5p | NM_018206 | VPS35 |
| hsa-miR-526b-5p | NM_001001436 | C16orf87 |
| hsa-miR-526b-5p | NM_004530 | MMP2 |
| hsa-miR-526b-5p | NM_001242795 | NUP93 |
| hsa-miR-526b-5p | NM_024946 | FAM192A |
| hsa-miR-526b-5p | NM_002080 | GOT2 |
| hsa-miR-526b-5p | NM_014329 | EDC4 |
| hsa-miR-526b-5p | NM_017803 | DUS2L |
| hsa-miR-526b-5p | NM_004555 | NFATC3 |
| hsa-miR-526b-5p | hsa_circ_0000712 | hsa_circ_002060 |
| hsa-miR-526b-5p | NM_133458 | ZFP90 |
| hsa-miR-526b-5p | NM_138714 | NFAT5 |
| hsa-miR-526b-5p | hsa_circ_0040153 | hsa_circ_0040153 |
| hsa-miR-526b-5p | NM_001605 | AARS |
| hsa-miR-526b-5p | hsa_circ_0000714 | hsa_circ_001572 |
| hsa-miR-526b-5p | NM_015386 | COG4 |
| hsa-miR-526b-5p | NM_018052 | VAC14 |
| hsa-miR-526b-5p | NM_006885 | ZFHX3 |
| hsa-miR-526b-5p | NM_012201 | GLG1 |
| hsa-miR-526b-5p | NM_001100624 | CENPN |
| hsa-miR-526b-5p | NM_015144 | ZCCHC14 |
| hsa-miR-526b-5p | NR_024402 | MGC23284 |
| hsa-miR-526b-5p | NM_001142864 | PIEZO1 |
| hsa-miR-526b-5p | NM_013275 | ANKRD11 |
| hsa-miR-526b-5p | hsa_circ_0000727 | hsa_circ_002048 |
| hsa-miR-526b-5p | NM_001197181 | TUBB3 |
| hsa-miR-526b-5p | NM_024792 | FAM57A |
| hsa-miR-526b-5p | NM_016823 | CRK |
| hsa-miR-526b-5p | NM_006445 | PRPF8 |
| hsa-miR-526b-5p | hsa_circ_0000733 | hsa_circ_000221 |
| hsa-miR-526b-5p | NM_014853 | SGSM2 |
| hsa-miR-526b-5p | NM_000430 | PAFAH1B1 |
| hsa-miR-526b-5p | NM_015229 | KIAA0664 |
| hsa-miR-526b-5p | NM_015113 | ZZEF1 |
| hsa-miR-526b-5p | NM_014520 | MYBBP1A |
| hsa-miR-526b-5p | NM_002798 | PSMB6 |
| hsa-miR-526b-5p | NM_002663 | PLD2 |
| hsa-miR-526b-5p | NM_006612 | KIF1C |
| hsa-miR-526b-5p | NM_004703 | RABEP1 |
| hsa-miR-526b-5p | NM_001212 | C1QBP |
| hsa-miR-526b-5p | NM_000018 | ACADVL |
| hsa-miR-526b-5p | NM_004422 | DVL2 |
| hsa-miR-526b-5p | NM_001005271 | CHD3 |
| hsa-miR-526b-5p | NM_003010 | MAP2K4 |
| hsa-miR-526b-5p | NM_001190440 | NCOR1 |
| hsa-miR-526b-5p | NM_201274 | MPRIP |
| hsa-miR-526b-5p | NM_004140 | LLGL1 |
| hsa-miR-526b-5p | NM_000691 | ALDH3A1 |
| hsa-miR-526b-5p | NM_001243439 | SPECC1 |
| hsa-miR-526b-5p | hsa_circ_0000745 | hsa_circ_000013 |
| hsa-miR-526b-5p | NM_015276 | USP22 |
| hsa-miR-526b-5p | NM_145109 | MAP2K3 |
| hsa-miR-526b-5p | NM_003170 | SUPT6H |
| hsa-miR-526b-5p | NM_005702 | ERAL1 |
| hsa-miR-526b-5p | NM_020791 | TAOK1 |
| hsa-miR-526b-5p | hsa_circ_0000752 | hsa_circ_000873 |
| hsa-miR-526b-5p | NM_024857 | ATAD5 |
| hsa-miR-526b-5p | NR_015341 | LRRC37BP1 |
| hsa-miR-526b-5p | NM_015355 | SUZ12 |
| hsa-miR-526b-5p | NM_032932 | RAB11FIP4 |
| hsa-miR-526b-5p | NM_018405 | C17orf79 |
| hsa-miR-526b-5p | NM_001033568 | RHOT1 |
| hsa-miR-526b-5p | NM_001104587 | SLFN11 |
| hsa-miR-526b-5p | NM_001030006 | AP2B1 |
| hsa-miR-526b-5p | NM_024835 | GGNBP2 |
| hsa-miR-526b-5p | NM_001166105 | TADA2A |
| hsa-miR-526b-5p | NM_001199417 | ARHGAP23 |
| hsa-miR-526b-5p | NM_003250 | THRA |
| hsa-miR-526b-5p | NM_001067 | TOP2A |
| hsa-miR-526b-5p | NM_002230 | JUP |
| hsa-miR-526b-5p | NM_002276 | KRT19 |
| hsa-miR-526b-5p | NM_001096 | ACLY |
| hsa-miR-526b-5p | NM_001144927 | NKIRAS2 |
| hsa-miR-526b-5p | NM_012448 | STAT5B |
| hsa-miR-526b-5p | NM_139276 | STAT3 |
| hsa-miR-526b-5p | NM_012232 | PTRF |
| hsa-miR-526b-5p | NM_001070 | TUBG1 |
| hsa-miR-526b-5p | hsa_circ_0000766 | hsa_circ_001851 |
| hsa-miR-526b-5p | NM_016437 | TUBG2 |
| hsa-miR-526b-5p | hsa_circ_0000767 | hsa_circ_002062 |
| hsa-miR-526b-5p | hsa_circ_0000768 | hsa_circ_000877 |
| hsa-miR-526b-5p | hsa_circ_0000769 | hsa_circ_000878 |
| hsa-miR-526b-5p | NM_003632 | CNTNAP1 |
| hsa-miR-526b-5p | NM_005533 | IFI35 |
| hsa-miR-526b-5p | NM_007300 | BRCA1 |
| hsa-miR-526b-5p | NM_005899 | NBR1 |
| hsa-miR-526b-5p | NM_005374 | MPP2 |
| hsa-miR-526b-5p | hsa_circ_0044175 | hsa_circ_0044175 |
| hsa-miR-526b-5p | hsa_circ_0044176 | hsa_circ_0044176 |
| hsa-miR-526b-5p | hsa_circ_0008067 | hsa_circ_0008067 |
| hsa-miR-526b-5p | NM_030753 | WNT3 |
| hsa-miR-526b-5p | hsa_circ_0044187 | hsa_circ_0044187 |
| hsa-miR-526b-5p | hsa_circ_0044189 | hsa_circ_0044189 |
| hsa-miR-526b-5p | hsa_circ_0044190 | hsa_circ_0044190 |
| hsa-miR-526b-5p | hsa_circ_0044191 | hsa_circ_0044191 |
| hsa-miR-526b-5p | NM_023079 | UBE2Z |
| hsa-miR-526b-5p | NM_001145365 | ZNF652 |
| hsa-miR-526b-5p | NM_000088 | COL1A1 |
| hsa-miR-526b-5p | NM_001130528 | SPAG9 |
| hsa-miR-526b-5p | NM_005082 | TRIM25 |
| hsa-miR-526b-5p | hsa_circ_0000787 | hsa_circ_001081 |
| hsa-miR-526b-5p | NM_003488 | AKAP1 |
| hsa-miR-526b-5p | NM_001078166 | SRSF1 |
| hsa-miR-526b-5p | NM_030938 | VMP1 |
| hsa-miR-526b-5p | NM_032582 | USP32 |
| hsa-miR-526b-5p | NM_025185 | TANC2 |
| hsa-miR-526b-5p | NM_005828 | DCAF7 |
| hsa-miR-526b-5p | NR_024386 | PLEKHM1P |
| hsa-miR-526b-5p | NM_002737 | PRKCA |
| hsa-miR-526b-5p | NM_014877 | HELZ |
| hsa-miR-526b-5p | NM_030630 | C17orf28 |
| hsa-miR-526b-5p | NM_001031803 | LLGL2 |
| hsa-miR-526b-5p | NM_000213 | ITGB4 |
| hsa-miR-526b-5p | NM_014230 | SRP68 |
| hsa-miR-526b-5p | NM_022066 | UBE2O |
| hsa-miR-526b-5p | NM_001142640 | TNRC6C |
| hsa-miR-526b-5p | NM_001127198 | TMC6 |
| hsa-miR-526b-5p | NM_025090 | USP36 |
| hsa-miR-526b-5p | NM_020914 | RNF213 |
| hsa-miR-526b-5p | NM_014984 | AZI1 |
| hsa-miR-526b-5p | NM_032711 | MAFG |
| hsa-miR-526b-5p | NM_004127 | GPS1 |
| hsa-miR-526b-5p | NM_004104 | FASN |
| hsa-miR-526b-5p | NM_006796 | AFG3L2 |
| hsa-miR-526b-5p | NM_031216 | SEH1L |
| hsa-miR-526b-5p | NM_032142 | CEP192 |
| hsa-miR-526b-5p | NR_033354 | ZNF519 |
| hsa-miR-526b-5p | NM_005406 | ROCK1 |
| hsa-miR-526b-5p | NM_001142966 | GREB1L |
| hsa-miR-526b-5p | NM_002894 | RBBP8 |
| hsa-miR-526b-5p | hsa_circ_0000837 | hsa_circ_001633 |
| hsa-miR-526b-5p | NM_001100619 | CABLES1 |
| hsa-miR-526b-5p | NM_000227 | LAMA3 |
| hsa-miR-526b-5p | NM_001792 | CDH2 |
| hsa-miR-526b-5p | NM_016271 | RNF138 |
| hsa-miR-526b-5p | NM_020474 | GALNT1 |
| hsa-miR-526b-5p | NM_018170 | RPRD1A |
| hsa-miR-526b-5p | NM_002647 | PIK3C3 |
| hsa-miR-526b-5p | NM_004671 | PIAS2 |
| hsa-miR-526b-5p | NM_005359 | SMAD4 |
| hsa-miR-526b-5p | NM_052947 | ALPK2 |
| hsa-miR-526b-5p | NM_176787 | PIGN |
| hsa-miR-526b-5p | NM_002035 | KDSR |
| hsa-miR-526b-5p | NM_014643 | ZNF516 |
| hsa-miR-526b-5p | NM_004715 | CTDP1 |
| hsa-miR-526b-5p | NM_005481 | MED16 |
| hsa-miR-526b-5p | NM_001687 | ATP5D |
| hsa-miR-526b-5p | NM_001280 | CIRBP |
| hsa-miR-526b-5p | NM_003938 | AP3D1 |
| hsa-miR-526b-5p | NM_032482 | DOT1L |
| hsa-miR-526b-5p | NM_032737 | LMNB2 |
| hsa-miR-526b-5p | hsa_circ_0000868 | hsa_circ_001499 |
| hsa-miR-526b-5p | NM_003021 | SGTA |
| hsa-miR-526b-5p | NM_002067 | GNA11 |
| hsa-miR-526b-5p | NM_133261 | GIPC3 |
| hsa-miR-526b-5p | NM_015898 | ZBTB7A |
| hsa-miR-526b-5p | NM_006012 | CLPP |
| hsa-miR-526b-5p | NM_024103 | SLC25A23 |
| hsa-miR-526b-5p | NM_001130955 | ARHGEF18 |
| hsa-miR-526b-5p | NM_145185 | MAP2K7 |
| hsa-miR-526b-5p | NM_024690 | MUC16 |
| hsa-miR-526b-5p | NM_152289 | ZNF561 |
| hsa-miR-526b-5p | NM_000201 | ICAM1 |
| hsa-miR-526b-5p | NM_001145056 | SLC44A2 |
| hsa-miR-526b-5p | NM_001005360 | DNM2 |
| hsa-miR-526b-5p | NM_004283 | RAB3D |
| hsa-miR-526b-5p | hsa_circ_0000892 | hsa_circ_001760 |
| hsa-miR-526b-5p | NM_001136195 | TNPO2 |
| hsa-miR-526b-5p | NM_024038 | C19orf43 |
| hsa-miR-526b-5p | NM_052850 | GADD45GIP1 |
| hsa-miR-526b-5p | NM_017722 | TRMT1 |
| hsa-miR-526b-5p | NM_058243 | BRD4 |
| hsa-miR-526b-5p | NM_021187 | CYP4F11 |
| hsa-miR-526b-5p | NM_001145160 | TPM4 |
| hsa-miR-526b-5p | NM_001130065 | MYO9B |
| hsa-miR-526b-5p | NM_005234 | NR2F6 |
| hsa-miR-526b-5p | NM_138442 | CCDC124 |
| hsa-miR-526b-5p | NM_005027 | PIK3R2 |
| hsa-miR-526b-5p | NM_001017392 | SUGP2 |
| hsa-miR-526b-5p | NM_017814 | TMEM161A |
| hsa-miR-526b-5p | NM_020410 | ATP13A1 |
| hsa-miR-526b-5p | NR_027130 | ZNF738 |
| hsa-miR-526b-5p | TCONS_l2_00012430 | TCONS_l2_00012430 |
| hsa-miR-526b-5p | NM_032816 | CEP89 |
| hsa-miR-526b-5p | NM_001114093 | LSM14A |
| hsa-miR-526b-5p | NM_000175 | GPI |
| hsa-miR-526b-5p | NM_005499 | UBA2 |
| hsa-miR-526b-5p | NM_015302 | HAUS5 |
| hsa-miR-526b-5p | NM_014727 | MLL4 |
| hsa-miR-526b-5p | NM_001199570 | CLIP3 |
| hsa-miR-526b-5p | NM_152279 | ZNF585B |
| hsa-miR-526b-5p | NM_152655 | ZNF585A |
| hsa-miR-526b-5p | NM_003169 | SUPT5H |
| hsa-miR-526b-5p | NM_001001563 | TIMM50 |
| hsa-miR-526b-5p | NM_005178 | BCL3 |
| hsa-miR-526b-5p | NM_000041 | APOE |
| hsa-miR-526b-5p | hsa_circ_0000939 | hsa_circ_002169 |
| hsa-miR-526b-5p | NM_000400 | ERCC2 |
| hsa-miR-526b-5p | NM_001983 | ERCC1 |
| hsa-miR-526b-5p | NM_004819 | SYMPK |
| hsa-miR-526b-5p | NM_005184 | CALM3 |
| hsa-miR-526b-5p | hsa_circ_0000943 | hsa_circ_001101 |
| hsa-miR-526b-5p | NM_004491 | ARHGAP35 |
| hsa-miR-526b-5p | NM_014681 | DHX34 |
| hsa-miR-526b-5p | NM_003009 | SEPW1 |
| hsa-miR-526b-5p | NM_002103 | GYS1 |
| hsa-miR-526b-5p | NM_006666 | RUVBL2 |
| hsa-miR-526b-5p | NM_012423 | RPL13A |
| hsa-miR-526b-5p | NM_020719 | PRR12 |
| hsa-miR-526b-5p | NM_007254 | PNKP |
| hsa-miR-526b-5p | NM_001145809 | MYH14 |
| hsa-miR-526b-5p | NM_007121 | NR1H2 |
| hsa-miR-526b-5p | NM_001199324 | ZNF615 |
| hsa-miR-526b-5p | NM_144684 | ZNF480 |
| hsa-miR-526b-5p | NM_014501 | UBE2S |
| hsa-miR-526b-5p | NM_016535 | ZNF581 |
| hsa-miR-526b-5p | NM_001130071 | EPN1 |
| hsa-miR-526b-5p | hsa_circ_0000962 | hsa_circ_001634 |
| hsa-miR-526b-5p | NM_001204814 | ZNF586 |
| hsa-miR-526b-5p | NM_032828 | ZNF587 |
| hsa-miR-526b-5p | NM_014480 | ZNF544 |
| hsa-miR-526b-5p | NM_005762 | TRIM28 |
| hsa-miR-526b-5p | NM_014453 | CHMP2A |
| hsa-miR-526b-5p | NM_021158 | TRIB3 |
| hsa-miR-526b-5p | NM_021873 | CDC25B |
| hsa-miR-526b-5p | NM_002592 | PCNA |
| hsa-miR-526b-5p | NM_015939 | TRMT6 |
| hsa-miR-526b-5p | NM_032485 | MCM8 |
| hsa-miR-526b-5p | NM_182734 | PLCB1 |
| hsa-miR-526b-5p | NM_001009608 | C20orf94 |
| hsa-miR-526b-5p | NM_020536 | CSRP2BP |
| hsa-miR-526b-5p | NM_032985 | SEC23B |
| hsa-miR-526b-5p | NM_001242581 | RIN2 |
| hsa-miR-526b-5p | NM_018474 | PLK1S1 |
| hsa-miR-526b-5p | NM_004798 | KIF3B |
| hsa-miR-526b-5p | NM_176812 | CHMP4B |
| hsa-miR-526b-5p | NM_001076552 | ACSS2 |
| hsa-miR-526b-5p | NM_000178 | GSS |
| hsa-miR-526b-5p | NM_018244 | UQCC |
| hsa-miR-526b-5p | hsa_circ_0001146 | hsa_circ_000657 |
| hsa-miR-526b-5p | NR_037188 | CPNE1 |
| hsa-miR-526b-5p | NR_040722 | RBM39 |
| hsa-miR-526b-5p | NM_016436 | PHF20 |
| hsa-miR-526b-5p | NM_032013 | NDRG3 |
| hsa-miR-526b-5p | NM_080627 | KIAA0889 |
| hsa-miR-526b-5p | NM_000457 | HNF4A |
| hsa-miR-526b-5p | NM_024331 | TTPAL |
| hsa-miR-526b-5p | NM_033550 | TP53RK |
| hsa-miR-526b-5p | NM_017895 | DDX27 |
| hsa-miR-526b-5p | NM_015339 | ADNP |
| hsa-miR-526b-5p | NM_020436 | SALL4 |
| hsa-miR-526b-5p | NM_199427 | ZFP64 |
| hsa-miR-526b-5p | NM_020673 | RAB22A |
| hsa-miR-526b-5p | NM_004738 | VAPB |
| hsa-miR-526b-5p | NM_144703 | LSM14B |
| hsa-miR-526b-5p | NM_031215 | CABLES2 |
| hsa-miR-526b-5p | NM_033081 | DIDO1 |
| hsa-miR-526b-5p | NR_037882 | RTEL1-TNFRSF6B |
| hsa-miR-526b-5p | NM_199360 | TPD52L2 |
| hsa-miR-526b-5p | TCONS_l2_00017148 | TCONS_l2_00017148 |
| hsa-miR-526b-5p | NM_003489 | NRIP1 |
| hsa-miR-526b-5p | NM_004540 | NCAM2 |
| hsa-miR-526b-5p | NM_000484 | APP |
| hsa-miR-526b-5p | NM_006585 | CCT8 |
| hsa-miR-526b-5p | NM_206866 | BACH1 |
| hsa-miR-526b-5p | hsa_circ_0001180 | hsa_circ_000338 |
| hsa-miR-526b-5p | hsa_circ_0001181 | hsa_circ_000666 |
| hsa-miR-526b-5p | NM_138927 | SON |
| hsa-miR-526b-5p | NM_001697 | ATP5O |
| hsa-miR-526b-5p | NM_006933 | SLC5A3 |
| hsa-miR-526b-5p | NM_003316 | TTC3 |
| hsa-miR-526b-5p | NM_101395 | DYRK1A |
| hsa-miR-526b-5p | hsa_circ_0001191 | hsa_circ_001503 |
| hsa-miR-526b-5p | NM_001098402 | ZNF295 |
| hsa-miR-526b-5p | NM_021075 | NDUFV3 |
| hsa-miR-526b-5p | NM_003274 | TRAPPC10 |
| hsa-miR-526b-5p | NM_005049 | PWP2 |
| hsa-miR-526b-5p | NR_024108 | PFKL |
| hsa-miR-526b-5p | NM_030582 | COL18A1 |
| hsa-miR-526b-5p | NM_001848 | COL6A1 |
| hsa-miR-526b-5p | NM_006031 | PCNT |
| hsa-miR-526b-5p | NM_015367 | BCL2L13 |
| hsa-miR-526b-5p | NM_015241 | MICAL3 |
| hsa-miR-526b-5p | NM_022719 | DGCR14 |
| hsa-miR-526b-5p | NM_003776 | MRPL40 |
| hsa-miR-526b-5p | NM_003325 | HIRA |
| hsa-miR-526b-5p | NM_032775 | KLHL22 |
| hsa-miR-526b-5p | NM_004327 | BCR |
| hsa-miR-526b-5p | NM_015330 | SPECC1L |
| hsa-miR-526b-5p | NM_001145418 | TTC28 |
| hsa-miR-526b-5p | NM_001005735 | CHEK2 |
| hsa-miR-526b-5p | NM_005080 | XBP1 |
| hsa-miR-526b-5p | NM_001127 | AP1B1 |
| hsa-miR-526b-5p | hsa_circ_0001217 | hsa_circ_000919 |
| hsa-miR-526b-5p | NM_032204 | ASCC2 |
| hsa-miR-526b-5p | NM_021090 | MTMR3 |
| hsa-miR-526b-5p | NM_014941 | MORC2 |
| hsa-miR-526b-5p | hsa_circ_0062927 | hsa_circ_0062927 |
| hsa-miR-526b-5p | NM_173566 | PRR14L |
| hsa-miR-526b-5p | NM_001031695 | RBFOX2 |
| hsa-miR-526b-5p | NM_002473 | MYH9 |
| hsa-miR-526b-5p | NM_003753 | EIF3D |
| hsa-miR-526b-5p | NM_003312 | TST |
| hsa-miR-526b-5p | NM_152221 | CSNK1E |
| hsa-miR-526b-5p | NM_001098504 | DDX17 |
| hsa-miR-526b-5p | NM_014876 | JOSD1 |
| hsa-miR-526b-5p | NM_014292 | CBX6 |
| hsa-miR-526b-5p | NM_019008 | SMCR7L |
| hsa-miR-526b-5p | NM_020831 | MKL1 |
| hsa-miR-526b-5p | NM_003932 | ST13 |
| hsa-miR-526b-5p | NM_022098 | XPNPEP3 |
| hsa-miR-526b-5p | NM_002883 | RANGAP1 |
| hsa-miR-526b-5p | NM_004599 | SREBF2 |
| hsa-miR-526b-5p | NM_005650 | TCF20 |
| hsa-miR-526b-5p | hsa_circ_0001241 | hsa_circ_001641 |
| hsa-miR-526b-5p | hsa_circ_0001242 | hsa_circ_002132 |
| hsa-miR-526b-5p | hsa_circ_0001243 | hsa_circ_002175 |
| hsa-miR-526b-5p | NM_001184970 | PACSIN2 |
| hsa-miR-526b-5p | NM_001003828 | PARVB |
| hsa-miR-526b-5p | NM_001009880 | KIAA0930 |
| hsa-miR-526b-5p | NM_001104595 | FAM118A |
| hsa-miR-526b-5p | hsa_circ_0001245 | hsa_circ_002137 |
| hsa-miR-526b-5p | NM_207327 | C22orf40 |
| hsa-miR-526b-5p | hsa_circ_0063867 | hsa_circ_0063867 |
| hsa-miR-526b-5p | NM_012401 | PLXNB2 |
| hsa-miR-526b-5p | NM_001242898 | PPP6R2 |
| hsa-miR-526b-5p | NM_033200 | LMF2 |
| hsa-miR-526b-5p | hsa_circ_0089786 | hsa_circ_0089786 |
| hsa-miR-526b-5p | hsa_circ_0089787 | hsa_circ_0089787 |
| hsa-miR-526b-5p | hsa_circ_0089788 | hsa_circ_0089788 |
| hsa-miR-526b-5p | hsa_circ_0089789 | hsa_circ_0089789 |
| hsa-miR-526b-5p | NM_001039091 | PRPS2 |
| hsa-miR-526b-5p | NM_003611 | OFD1 |
| hsa-miR-526b-5p | NM_001144002 | CTPS2 |
| hsa-miR-526b-5p | NM_001178085 | ZFX |
| hsa-miR-526b-5p | NM_016937 | POLA1 |
| hsa-miR-526b-5p | NM_001039590 | USP9X |
| hsa-miR-526b-5p | NM_001356 | DDX3X |
| hsa-miR-526b-5p | NR_024234 | NDUFB11 |
| hsa-miR-526b-5p | NM_003334 | UBA1 |
| hsa-miR-526b-5p | NM_002536 | TBC1D25 |
| hsa-miR-526b-5p | NM_006306 | SMC1A |
| hsa-miR-526b-5p | NM_031407 | HUWE1 |
| hsa-miR-526b-5p | NM_015107 | PHF8 |
| hsa-miR-526b-5p | NM_017848 | FAM120C |
| hsa-miR-526b-5p | NM_004463 | FGD1 |
| hsa-miR-526b-5p | NM_001184819 | GNL3L |
| hsa-miR-526b-5p | NM_031206 | LAS1L |
| hsa-miR-526b-5p | NM_002444 | MSN |
| hsa-miR-526b-5p | NM_001011645 | AR |
| hsa-miR-526b-5p | NM_012310 | KIF4A |
| hsa-miR-526b-5p | NM_181672 | OGT |
| hsa-miR-526b-5p | NM_001013627 | NHSL2 |
| hsa-miR-526b-5p | TCONS_00016926 | TCONS_00016926 |
| hsa-miR-526b-5p | NM_000052 | ATP7A |
| hsa-miR-526b-5p | NM_006729 | DIAPH2 |
| hsa-miR-526b-5p | NR_038988 | LOC100287765 |
| hsa-miR-526b-5p | NM_194324 | TMSB15B |
| hsa-miR-526b-5p | NM_024657 | MORC4 |
| hsa-miR-526b-5p | NM_012216 | MID2 |
| hsa-miR-526b-5p | NM_033380 | COL4A5 |
| hsa-miR-526b-5p | NM_001560 | IL13RA1 |
| hsa-miR-526b-5p | NM_024528 | NKAP |
| hsa-miR-526b-5p | NM_013995 | LAMP2 |
| hsa-miR-526b-5p | NM_001167 | XIAP |
| hsa-miR-526b-5p | NM_021946 | BCORL1 |
| hsa-miR-526b-5p | NM_021183 | RAP2C |
| hsa-miR-526b-5p | NM_001164617 | GPC3 |
| hsa-miR-526b-5p | NM_032458 | PHF6 |
| hsa-miR-526b-5p | NM_173694 | ATP11C |
| hsa-miR-526b-5p | NM_001184750 | SLITRK4 |
| hsa-miR-526b-5p | NM_001139457 | BCAP31 |
| hsa-miR-526b-5p | NM_005334 | HCFC1 |
| hsa-miR-526b-5p | NM_001569 | IRAK1 |
| hsa-miR-526b-5p | NM_001183 | ATP6AP1 |
| hsa-miR-526b-5p | NM_001636 | SLC25A6 |
