# Supplementary material for: Circ0085539 Promotes Osteosarcoma Progression by Suppressing miR-526b-5p and PHLDA1 Axis
Source: Front Oncol. 2020 Aug 26;10:1250. doi: 10.3389/fonc.2020.01250 (PMC7479240; doi:10.3389/fonc.2020.01250)
Supplement: Supplementary Table 4 — The target genes of miR-526b-5p were predicted by Tarbase 8.0. [file Table_4.DOCX]

Supplementary table 4 The target genes of miR-526b-5p were predicted by Tarbase 8.0.

| Gene name | miRNA |
| --- | --- |
| MTA2 | hsa-miR-526b-5p |
| RRM2 | hsa-miR-526b-5p |
| HIST1H1B | hsa-miR-526b-5p |
| MATR3 | hsa-miR-526b-5p |
| ZSWIM6 | hsa-miR-526b-5p |
| APPL2 | hsa-miR-526b-5p |
| PHLDA1 | hsa-miR-526b-5p |
| TXLNA | hsa-miR-526b-5p |
| GATA6 | hsa-miR-526b-5p |
| NDUFS1 | hsa-miR-526b-5p |
| ABCF2 | hsa-miR-526b-5p |
| CDK13 | hsa-miR-526b-5p |
| PKM | hsa-miR-526b-5p |
| ACADVL | hsa-miR-526b-5p |
| DDX24 | hsa-miR-526b-5p |
| ICAM1 | hsa-miR-526b-5p |
| ZAK | hsa-miR-526b-5p |
| DICER1 | hsa-miR-526b-5p |
| VAPA | hsa-miR-526b-5p |
| ARMCX3 | hsa-miR-526b-5p |
| RPL19 | hsa-miR-526b-5p |
| CORO1C | hsa-miR-526b-5p |
| COL12A1 | hsa-miR-526b-5p |
| SASH1 | hsa-miR-526b-5p |
| VEGFA | hsa-miR-526b-5p |
| FOXP1 | hsa-miR-526b-5p |
| LMAN2L | hsa-miR-526b-5p |
| SPTBN1 | hsa-miR-526b-5p |
| CTGF | hsa-miR-526b-5p |
| YPEL5 | hsa-miR-526b-5p |
| TMEM189-UBE2V1 | hsa-miR-526b-5p |
| AHNAK | hsa-miR-526b-5p |
| YWHAH | hsa-miR-526b-5p |
| SLC35D2 | hsa-miR-526b-5p |
| RPL27 | hsa-miR-526b-5p |
| MAP1B | hsa-miR-526b-5p |
| PLAA | hsa-miR-526b-5p |
| SEC24B | hsa-miR-526b-5p |
| ARHGDIA | hsa-miR-526b-5p |
| RPS8 | hsa-miR-526b-5p |
| ECM1 | hsa-miR-526b-5p |
| MCL1 | hsa-miR-526b-5p |
| PM20D2 | hsa-miR-526b-5p |
| MKI67 | hsa-miR-526b-5p |
| FCHO2 | hsa-miR-526b-5p |
| SV2A | hsa-miR-526b-5p |
| ZNF281 | hsa-miR-526b-5p |
| PBRM1 | hsa-miR-526b-5p |
| WEE1 | hsa-miR-526b-5p |
| TPM4 | hsa-miR-526b-5p |
| SRRM2 | hsa-miR-526b-5p |
| HSPA4 | hsa-miR-526b-5p |
| PLRG1 | hsa-miR-526b-5p |
| RAPH1 | hsa-miR-526b-5p |
| TGIF1 | hsa-miR-526b-5p |
| PTRF | hsa-miR-526b-5p |
| SUZ12 | hsa-miR-526b-5p |
| SRSF10 | hsa-miR-526b-5p |
| TUBB | hsa-miR-526b-5p |
| LONP1 | hsa-miR-526b-5p |
| HIST1H3D | hsa-miR-526b-5p |
| ZNF652 | hsa-miR-526b-5p |
| PHB2 | hsa-miR-526b-5p |
| NAMPTL | hsa-miR-526b-5p |
| RPS18 | hsa-miR-526b-5p |
| RBM12 | hsa-miR-526b-5p |
| ZHX1-C8orf76 | hsa-miR-526b-5p |
| AK6 | hsa-miR-526b-5p |
| HIST1H3H | hsa-miR-526b-5p |
