# Supplementary material for: Circ0085539 Promotes Osteosarcoma Progression by Suppressing miR-526b-5p and PHLDA1 Axis
Source: Front Oncol. 2020 Aug 26;10:1250. doi: 10.3389/fonc.2020.01250 (PMC7479240; doi:10.3389/fonc.2020.01250)
Supplement: Supplementary Table 5 — The target genes of miR-526b-5p were predicted by TargetScan Human 7.2. [file Table_5.DOCX]

Supplementary table 5 The target genes of miR-526b-5p were predicted by TargetScan Human 7.2.

| Target gene | Representative transcript | Gene name |
| --- | --- | --- |
| RP11-1212A22.4 | ENST00000545114.1 | NPIP-like protein 1 |
| CDKN2AIP | ENST00000302350.4 | CDKN2A interacting protein |
| VRK2 | ENST00000412104.2 | vaccinia related kinase 2 |
| RAB11FIP3 | ENST00000262305.4 | RAB11 family interacting protein 3 (class II) |
| C12orf75 | ENST00000443585.1 | chromosome 12 open reading frame 75 |
| AL590452.1 | ENST00000596396.1 | Uncharacterized protein |
| SIGLECL1 | ENST00000316401.7 | SIGLEC family like 1 |
| INIP | ENST00000374242.4 | INTS3 and NABP interacting protein |
| DCN | ENST00000456569.2 | decorin |
| MRPS28 | ENST00000521605.1 | mitochondrial ribosomal protein S28 |
| CHEK2 | ENST00000382566.1 | checkpoint kinase 2 |
| ACTR3C | ENST00000252071.4 | ARP3 actin-related protein 3 homolog C (yeast) |
| AGXT2 | ENST00000231420.6 | alanine--glyoxylate aminotransferase 2 |
| NAP1L3 | ENST00000373079.3 | nucleosome assembly protein 1-like 3 |
| AGO1 | ENST00000373204.4 | argonaute RISC catalytic component 1 |
| GRB14 | ENST00000263915.3 | growth factor receptor-bound protein 14 |
| PPP3CC | ENST00000289963.8 | protein phosphatase 3, catalytic subunit, gamma isozyme |
| CCT8 | ENST00000286788.4 | chaperonin containing TCP1, subunit 8 (theta) |
| HENMT1 | ENST00000402983.1 | HEN1 methyltransferase homolog 1 (Arabidopsis) |
| ZNF534 | ENST00000301085.4 | zinc finger protein 534 |
| AL353698.1 | ENST00000433279.1 | Uncharacterized protein |
| TMEM27 | ENST00000380342.3 | transmembrane protein 27 |
| PDIA6 | ENST00000272227.3 | protein disulfide isomerase family A, member 6 |
| RARB | ENST00000437042.2 | retinoic acid receptor, beta |
| EBPL | ENST00000378284.2 | emopamil binding protein-like |
| C6orf123 | ENST00000366822.2 | chromosome 6 open reading frame 123 |
| VPS52 | ENST00000482399.1 | vacuolar protein sorting 52 homolog (S. cerevisiae) |
| UBE2V2 | ENST00000523111.2 | ubiquitin-conjugating enzyme E2 variant 2 |
| CDADC1 | ENST00000251108.6 | cytidine and dCMP deaminase domain containing 1 |
| CDK6 | ENST00000265734.4 | cyclin-dependent kinase 6 |
| GFRAL | ENST00000340465.2 | GDNF family receptor alpha like |
| MRPS18A | ENST00000372116.1 | mitochondrial ribosomal protein S18A |
| FAM86B2 | ENST00000393715.3 | family with sequence similarity 86, member B2 |
| LYPLA1 | ENST00000316963.3 | lysophospholipase I |
| IYD | ENST00000344419.3 | iodotyrosine deiodinase |
| GNG10 | ENST00000374293.4 | guanine nucleotide binding protein (G protein), gamma 10 |
| KLHDC1 | ENST00000359332.2 | kelch domain containing 1 |
| C14orf119 | ENST00000319074.4 | chromosome 14 open reading frame 119 |
| CR2 | ENST00000367058.3 | complement component (3d/Epstein Barr virus) receptor 2 |
| PYROXD2 | ENST00000370575.4 | pyridine nucleotide-disulphide oxidoreductase domain 2 |
| TPMT | ENST00000309983.4 | thiopurine S-methyltransferase |
| XRCC5 | ENST00000392133.3 | X-ray repair complementing defective repair in Chinese hamster cells 5 (double-strand-break rejoining) |
| RAP2C | ENST00000342983.2 | RAP2C, member of RAS oncogene family |
| NDFIP1 | ENST00000253814.4 | Nedd4 family interacting protein 1 |
| SRD5A3 | ENST00000264228.4 | steroid 5 alpha-reductase 3 |
| MTCP1 | ENST00000369476.3 | mature T-cell proliferation 1 |
| C1orf52 | ENST00000471115.1 | chromosome 1 open reading frame 52 |
| CSHL1 | ENST00000561003.1 | chorionic somatomammotropin hormone-like 1 |
| SULT1A1 | ENST00000350842.4 | sulfotransferase family, cytosolic, 1A, phenol-preferring, member 1 |
| MAGEA3 | ENST00000370278.3 | melanoma antigen family A, 3 |
| KIAA0226L | ENST00000378781.3 | KIAA0226-like |
| MAGEA6 | ENST00000329342.5 | melanoma antigen family A, 6 |
| AKR1B15 | ENST00000457545.2 | aldo-keto reductase family 1, member B15 |
| FKTN | ENST00000223528.2 | fukutin |
| NR1H4 | ENST00000548884.1 | nuclear receptor subfamily 1, group H, member 4 |
| TNFAIP8L1 | ENST00000327473.4 | tumor necrosis factor, alpha-induced protein 8-like 1 |
| ZNF124 | ENST00000340684.6 | zinc finger protein 124 |
| CASP14 | ENST00000427043.3 | caspase 14, apoptosis-related cysteine peptidase |
| C11orf34 | ENST00000338832.2 | chromosome 11 open reading frame 34 |
| DMTN | ENST00000265800.5 | dematin actin binding protein |
| RP11-247C2.2 | ENST00000563727.1 | HCG2004779; Uncharacterized protein |
| ATF3 | ENST00000366983.1 | activating transcription factor 3 |
| GH2 | ENST00000332800.7 | growth hormone 2 |
| APOBEC3H | ENST00000401756.1 | apolipoprotein B mRNA editing enzyme, catalytic polypeptide-like 3H |
| APOPT1 | ENST00000556253.2 | apoptogenic 1, mitochondrial |
| FGF1 | ENST00000360966.5 | fibroblast growth factor 1 (acidic) |
| CSH1 | ENST00000329882.8 | chorionic somatomammotropin hormone 1 (placental lactogen) |
| PKP2 | ENST00000340811.4 | plakophilin 2 |
| FAM180A | ENST00000338588.3 | family with sequence similarity 180, member A |
| CHST15 | ENST00000346248.5 | carbohydrate (N-acetylgalactosamine 4-sulfate 6-O) sulfotransferase 15 |
| C21orf49 | ENST00000382375.4 | chromosome 21 open reading frame 49 |
| JAK1 | ENST00000342505.4 | Janus kinase 1 |
| RIMBP3B | ENST00000434111.1 | RIMS binding protein 3B |
| RIMBP3 | ENST00000426804.1 | RIMS binding protein 3 |
| ZSWIM7 | ENST00000486655.1 | zinc finger, SWIM-type containing 7 |
| DNAJC25 | ENST00000556107.1 | DnaJ (Hsp40) homolog, subfamily C , member 25 |
| GLRA3 | ENST00000274093.3 | glycine receptor, alpha 3 |
| DNAJC25-GNG10 | ENST00000374294.3 | DNAJC25-GNG10 readthrough |
| MED20 | ENST00000409312.1 | mediator complex subunit 20 |
| POTEE | ENST00000358087.5 | POTE ankyrin domain family, member E |
| CREB1 | ENST00000432329.2 | cAMP responsive element binding protein 1 |
| GABRG1 | ENST00000295452.4 | gamma-aminobutyric acid (GABA) A receptor, gamma 1 |
| SAYSD1 | ENST00000373249.1 | SAYSVFN motif domain containing 1 |
| TPM4 | ENST00000300933.4 | tropomyosin 4 |
| AC026703.1 | ENST00000326958.1 |  |
| SYTL4 | ENST00000372989.1 | synaptotagmin-like 4 |
| NANOS2 | ENST00000341294.2 | nanos homolog 2 (Drosophila) |
| LIX1 | ENST00000274382.4 | Lix1 homolog (chicken) |
| RPN2 | ENST00000237530.6 | ribophorin II |
| CNOT2 | ENST00000229195.3 | CCR4-NOT transcription complex, subunit 2 |
| PM20D2 | ENST00000275072.4 | peptidase M20 domain containing 2 |
| CASD1 | ENST00000297273.4 | CAS1 domain containing 1 |
| PHF6 | ENST00000332070.3 | PHD finger protein 6 |
| SDR42E1 | ENST00000328945.5 | short chain dehydrogenase/reductase family 42E, member 1 |
| TFPI | ENST00000392365.1 | tissue factor pathway inhibitor (lipoprotein-associated coagulation inhibitor) |
| MGAT4C | ENST00000604798.1 | mannosyl (alpha-1,3-)-glycoprotein beta-1,4-N-acetylglucosaminyltransferase, isozyme C (putative) |
| OR9Q1 | ENST00000335397.3 | olfactory receptor, family 9, subfamily Q, member 1 |
| UCP3 | ENST00000314032.4 | uncoupling protein 3 (mitochondrial, proton carrier) |
| CTNNB1 | ENST00000349496.5 | catenin (cadherin-associated protein), beta 1, 88kDa |
| AC093157.1 | ENST00000593496.1 | Uncharacterized protein |
| CAPN13 | ENST00000295055.8 | calpain 13 |
| AC093802.1 | ENST00000407524.1 | Uncharacterized protein |
| SNX31 | ENST00000311812.2 | sorting nexin 31 |
| FBXO47 | ENST00000378079.2 | F-box protein 47 |
| RPTN | ENST00000316073.3 | repetin |
| ACBD5 | ENST00000396271.3 | acyl-CoA binding domain containing 5 |
| CCBL2 | ENST00000370485.2 | cysteine conjugate-beta lyase 2 |
| CNTN6 | ENST00000446702.2 | contactin 6 |
| NDUFB11 | ENST00000377811.3 | NADH dehydrogenase (ubiquinone) 1 beta subcomplex, 11, 17.3kDa |
| XRN1 | ENST00000264951.4 | 5'-3' exoribonuclease 1 |
| PSME1 | ENST00000382708.3 | proteasome (prosome, macropain) activator subunit 1 (PA28 alpha) |
| MAGEA12 | ENST00000357916.4 | melanoma antigen family A, 12 |
| SLC13A4 | ENST00000354042.4 | solute carrier family 13 (sodium/sulfate symporter), member 4 |
| FAM86B1 | ENST00000448228.2 | family with sequence similarity 86, member B1 |
| ALPK1 | ENST00000458497.1 | alpha-kinase 1 |
| PLGRKT | ENST00000223864.2 | plasminogen receptor, C-terminal lysine transmembrane protein |
| TPD52L3 | ENST00000344545.5 | tumor protein D52-like 3 |
| DNAJC5G | ENST00000296097.3 | DnaJ (Hsp40) homolog, subfamily C, member 5 gamma |
| SRP9 | ENST00000366838.1 | signal recognition particle 9kDa |
| DTX3L | ENST00000296161.4 | deltex 3-like (Drosophila) |
| CYP3A5 | ENST00000339843.2 | cytochrome P450, family 3, subfamily A, polypeptide 5 |
| COL18A1 | ENST00000400337.2 | collagen, type XVIII, alpha 1 |
| RP11-127H5.1 | ENST00000521923.1 | Uncharacterized protein |
| FAM19A1 | ENST00000478136.1 | family with sequence similarity 19 (chemokine (C-C motif)-like), member A1 |
| SERPINB10 | ENST00000238508.3 | serpin peptidase inhibitor, clade B (ovalbumin), member 10 |
| CNPY3 | ENST00000394142.3 | canopy FGF signaling regulator 3 |
| FAM228B | ENST00000407625.1 | family with sequence similarity 228, member B |
| LPAR1 | ENST00000374431.3 | lysophosphatidic acid receptor 1 |
| EPHB1 | ENST00000398015.3 | EPH receptor B1 |
| NANOG | ENST00000229307.4 | Nanog homeobox |
| PRKCSH | ENST00000252455.2 | protein kinase C substrate 80K-H |
| PILRA | ENST00000394000.2 | paired immunoglobin-like type 2 receptor alpha |
| EIF4E3 | ENST00000425534.3 | eukaryotic translation initiation factor 4E family member 3 |
| PRTFDC1 | ENST00000376378.1 | phosphoribosyl transferase domain containing 1 |
| ABCB10 | ENST00000344517.4 | ATP-binding cassette, sub-family B (MDR/TAP), member 10 |
| PRDM7 | ENST00000325921.6 | PR domain containing 7 |
| PXMP4 | ENST00000344022.3 | peroxisomal membrane protein 4, 24kDa |
| GDPD4 | ENST00000315938.4 | glycerophosphodiester phosphodiesterase domain containing 4 |
| TMEM232 | ENST00000429839.2 | transmembrane protein 232 |
| HTN3 | ENST00000530128.1 | histatin 3 |
| CSH2 | ENST00000336844.5 | chorionic somatomammotropin hormone 2 |
| EID1 | ENST00000530028.2 | EP300 interacting inhibitor of differentiation 1 |
| CD46 | ENST00000358170.2 | CD46 molecule, complement regulatory protein |
| CD200R1 | ENST00000471858.1 | CD200 receptor 1 |
| ZNF589 | ENST00000427617.2 | zinc finger protein 589 |
| DCAF8L1 | ENST00000441525.1 | DDB1 and CUL4 associated factor 8-like 1 |
| TACR3 | ENST00000304883.2 | tachykinin receptor 3 |
| SYNJ2 | ENST00000449859.2 | synaptojanin 2 |
| TMPRSS15 | ENST00000284885.3 | transmembrane protease, serine 15 |
| ERAP2 | ENST00000437043.3 | endoplasmic reticulum aminopeptidase 2 |
| LAMTOR3 | ENST00000499666.2 | late endosomal/lysosomal adaptor, MAPK and MTOR activator 3 |
| SULT1A3 | ENST00000395138.2 | sulfotransferase family, cytosolic, 1A, phenol-preferring, member 3 |
| C12orf65 | ENST00000253233.1 | chromosome 12 open reading frame 65 |
| HMG20A | ENST00000336216.4 | high mobility group 20A |
| NDST4 | ENST00000264363.2 | N-deacetylase/N-sulfotransferase (heparan glucosaminyl) 4 |
| SLC35D2 | ENST00000253270.7 | solute carrier family 35 (UDP-GlcNAc/UDP-glucose transporter), member D2 |
| CA1 | ENST00000523953.1 | carbonic anhydrase I |
| SULT1A4 | ENST00000344620.6 | sulfotransferase family, cytosolic, 1A, phenol-preferring, member 4 |
| LRIF1 | ENST00000369763.4 | ligand dependent nuclear receptor interacting factor 1 |
| TTLL7 | ENST00000260505.8 | tubulin tyrosine ligase-like family, member 7 |
| SAE1 | ENST00000392776.3 | SUMO1 activating enzyme subunit 1 |
| ZMAT1 | ENST00000372782.3 | zinc finger, matrin-type 1 |
| KTN1 | ENST00000416613.1 | kinectin 1 (kinesin receptor) |
| ACTRT3 | ENST00000330368.2 | actin-related protein T3 |
| B3GALT2 | ENST00000367434.4 | UDP-Gal:betaGlcNAc beta 1,3-galactosyltransferase, polypeptide 2 |
| NT5DC3 | ENST00000392876.3 | 5'-nucleotidase domain containing 3 |
| GRIN2B | ENST00000609686.1 | glutamate receptor, ionotropic, N-methyl D-aspartate 2B |
| FAR2 | ENST00000536681.3 | fatty acyl CoA reductase 2 |
| CD177 | ENST00000378009.4 | CD177 molecule |
| ERH | ENST00000557016.1 | enhancer of rudimentary homolog (Drosophila) |
| C12orf39 | ENST00000256969.2 | chromosome 12 open reading frame 39 |
| PRELP | ENST00000343110.2 | proline/arginine-rich end leucine-rich repeat protein |
| FAM47E | ENST00000515604.1 | Protein FAM47E |
| DERL2 | ENST00000572834.1 | derlin 2 |
| CEP78 | ENST00000415759.2 | centrosomal protein 78kDa |
| PVRL3 | ENST00000319792.3 | poliovirus receptor-related 3 |
| AC137056.1 | ENST00000593357.1 | Uncharacterized protein; cDNA FLJ34659 fis, clone KIDNE2018863 |
| CMTM6 | ENST00000205636.3 | CKLF-like MARVEL transmembrane domain containing 6 |
| NDUFB2 | ENST00000482954.1 | NADH dehydrogenase (ubiquinone) 1 beta subcomplex, 2, 8kDa |
| RPL37 | ENST00000274242.5 | ribosomal protein L37 |
| PDZD4 | ENST00000164640.4 | PDZ domain containing 4 |
| PALM3 | ENST00000340790.4 | paralemmin 3 |
| MLLT10 | ENST00000377072.3 | myeloid/lymphoid or mixed-lineage leukemia (trithorax homolog, Drosophila); translocated to, 10 |
| CDHR3 | ENST00000343407.5 | cadherin-related family member 3 |
| FUT6 | ENST00000524754.1 | fucosyltransferase 6 (alpha (1,3) fucosyltransferase) |
| THSD7B | ENST00000409968.1 | thrombospondin, type I, domain containing 7B |
| EXT2 | ENST00000395673.3 | exostosin glycosyltransferase 2 |
| BIVM | ENST00000448849.2 | basic, immunoglobulin-like variable motif containing |
| TPST2 | ENST00000338754.4 | tyrosylprotein sulfotransferase 2 |
| AC117834.1 | ENST00000391681.1 |  |
| ZNF226 | ENST00000588883.1 | zinc finger protein 226 |
| HRH2 | ENST00000377291.2 | histamine receptor H2 |
| CFI | ENST00000394635.3 | complement factor I |
| SMIM10 | ENST00000330288.4 | small integral membrane protein 10 |
| AC022498.1 | ENST00000392468.2 | Uncharacterized protein |
| PGRMC2 | ENST00000296425.5 | progesterone receptor membrane component 2 |
| FAM120AOS | ENST00000423591.1 | family with sequence similarity 120A opposite strand |
| MGAM | ENST00000549489.2 | maltase-glucoamylase (alpha-glucosidase) |
| CCDC74B | ENST00000409943.3 | coiled-coil domain containing 74B |
| GOLGA8J | ENST00000341650.6 | golgin A8 family, member J |
| FAM3B | ENST00000357985.2 | family with sequence similarity 3, member B |
| PTPMT1 | ENST00000426530.2 | protein tyrosine phosphatase, mitochondrial 1 |
| TTC32 | ENST00000402414.1 | tetratricopeptide repeat domain 32 |
| ANP32E | ENST00000533654.1 | acidic (leucine-rich) nuclear phosphoprotein 32 family, member E |
| ADTRP | ENST00000379413.2 | androgen-dependent TFPI-regulating protein |
| GIPC3 | ENST00000322315.5 | GIPC PDZ domain containing family, member 3 |
| GOLGA8A | ENST00000360553.3 | golgin A8 family, member A |
| TMEM196 | ENST00000405844.1 | transmembrane protein 196 |
| PROKR2 | ENST00000546004.1 | prokineticin receptor 2 |
| GOLGA8B | ENST00000342314.5 | golgin A8 family, member B |
| FGF10 | ENST00000264664.4 | fibroblast growth factor 10 |
| METTL8 | ENST00000375258.4 | methyltransferase like 8 |
| CEP44 | ENST00000296519.4 | centrosomal protein 44kDa |
| DST | ENST00000312431.6 | dystonin |
| C1GALT1 | ENST00000223122.3 | core 1 synthase, glycoprotein-N-acetylgalactosamine 3-beta-galactosyltransferase, 1 |
| ZNF7 | ENST00000528372.1 | zinc finger protein 7 |
| VAMP3 | ENST00000054666.6 | vesicle-associated membrane protein 3 |
| TRAPPC2 | ENST00000453655.2 | trafficking protein particle complex 2 |
| ZFP36L1 | ENST00000555997.1 | ZFP36 ring finger protein-like 1 |
| ZNF300 | ENST00000427179.1 | zinc finger protein 300 |
| TMX1 | ENST00000457354.2 | thioredoxin-related transmembrane protein 1 |
| VPS13B | ENST00000395996.1 | vacuolar protein sorting 13 homolog B (yeast) |
| AC004899.1 | ENST00000596947.1 | Uncharacterized protein |
| AC079210.1 | ENST00000600820.1 | Uncharacterized protein; cDNA FLJ45097 fis, clone BRAWH3031054 |
| NME8 | ENST00000199447.4 | NME/NM23 family member 8 |
| MYCT1 | ENST00000367245.5 | myc target 1 |
| AACS | ENST00000261686.6 | acetoacetyl-CoA synthetase |
| MARVELD1 | ENST00000285605.6 | MARVEL domain containing 1 |
| SLITRK4 | ENST00000381779.4 | SLIT and NTRK-like family, member 4 |
| ESF1 | ENST00000202816.1 | ESF1, nucleolar pre-rRNA processing protein, homolog (S. cerevisiae) |
| CENPN | ENST00000305850.5 | centromere protein N |
| ACER3 | ENST00000532485.1 | alkaline ceramidase 3 |
| TTC26 | ENST00000430935.1 | tetratricopeptide repeat domain 26 |
| C15orf40 | ENST00000304177.5 | chromosome 15 open reading frame 40 |
| GOLGA8O | ENST00000509311.2 | golgin A8 family, member O |
| GOLGA8R | ENST00000327271.10 | golgin A8 family, member R |
| AC112715.2 | ENST00000445534.2 | Uncharacterized protein |
| CD96 | ENST00000352690.4 | CD96 molecule |
| MDM2 | ENST00000462284.1 | MDM2 oncogene, E3 ubiquitin protein ligase |
| IPP | ENST00000396478.3 | intracisternal A particle-promoted polypeptide |
| ABCD2 | ENST00000308666.3 | ATP-binding cassette, sub-family D (ALD), member 2 |
| SAMD12 | ENST00000409003.4 | sterile alpha motif domain containing 12 |
| SYN1 | ENST00000340666.4 | synapsin I |
| TBX21 | ENST00000177694.1 | T-box 21 |
| CKMT1A | ENST00000434505.1 | creatine kinase, mitochondrial 1A |
| ZNF14 | ENST00000344099.3 | zinc finger protein 14 |
| CLCN2 | ENST00000423355.2 | chloride channel, voltage-sensitive 2 |
| PPP3R2 | ENST00000374806.1 | protein phosphatase 3, regulatory subunit B, beta |
| HINT1 | ENST00000304043.5 | histidine triad nucleotide binding protein 1 |
| TIA1 | ENST00000433529.2 | TIA1 cytotoxic granule-associated RNA binding protein |
| ARMC2 | ENST00000392644.4 | armadillo repeat containing 2 |
| STAM | ENST00000377524.3 | signal transducing adaptor molecule (SH3 domain and ITAM motif) 1 |
| C8orf46 | ENST00000522977.1 | chromosome 8 open reading frame 46 |
| STAG3 | ENST00000317296.5 | stromal antigen 3 |
| IL18R1 | ENST00000409599.1 | interleukin 18 receptor 1 |
| C8B | ENST00000371237.4 | complement component 8, beta polypeptide |
| C9orf53 | ENST00000441769.2 | chromosome 9 open reading frame 53 |
| ACADSB | ENST00000358776.4 | acyl-CoA dehydrogenase, short/branched chain |
| GOLGA8H | ENST00000566740.1 | golgin A8 family, member H |
| MEOX2 | ENST00000262041.5 | mesenchyme homeobox 2 |
| HELLS | ENST00000394036.1 | helicase, lymphoid-specific |
| BLVRA | ENST00000402924.1 | biliverdin reductase A |
| ITM2A | ENST00000373298.2 | integral membrane protein 2A |
| OR10H5 | ENST00000308940.8 | olfactory receptor, family 10, subfamily H, member 5 |
| KIAA1107 | ENST00000370378.4 | KIAA1107 |
| TEFM | ENST00000580840.1 | transcription elongation factor, mitochondrial |
| THBS1 | ENST00000260356.5 | thrombospondin 1 |
| ADORA3 | ENST00000369716.4 | adenosine A3 receptor |
| ZDBF2 | ENST00000374423.3 | zinc finger, DBF-type containing 2 |
| THAP6 | ENST00000311638.3 | THAP domain containing 6 |
| MMP1 | ENST00000315274.6 | matrix metallopeptidase 1 (interstitial collagenase) |
| CKMT1B | ENST00000300283.6 | creatine kinase, mitochondrial 1B |
| GOLGA8M | ENST00000563027.1 | golgin A8 family, member M |
| MYLK | ENST00000360772.3 | myosin light chain kinase |
| UGT8 | ENST00000310836.6 | UDP glycosyltransferase 8 |
| CTXN3 | ENST00000379445.3 | cortexin 3 |
| CXCL11 | ENST00000306621.3 | chemokine (C-X-C motif) ligand 11 |
| SPARCL1 | ENST00000282470.6 | SPARC-like 1 (hevin) |
| MYBPC3 | ENST00000545968.1 | myosin binding protein C, cardiac |
| RBM12B-AS1 | ENST00000391680.1 | RBM12B antisense RNA 1 |
| OTOR | ENST00000246081.2 | otoraplin |
| GABRG2 | ENST00000356592.3 | gamma-aminobutyric acid (GABA) A receptor, gamma 2 |
| NR5A2 | ENST00000367362.3 | nuclear receptor subfamily 5, group A, member 2 |
| CDX2 | ENST00000381020.7 | caudal type homeobox 2 |
| AGMAT | ENST00000375826.3 | agmatine ureohydrolase (agmatinase) |
| APOLD1 | ENST00000356591.4 | apolipoprotein L domain containing 1 |
| EDARADD | ENST00000359362.5 | EDAR-associated death domain |
| MDP1 | ENST00000396833.2 | magnesium-dependent phosphatase 1 |
| ADAM28 | ENST00000265769.4 | ADAM metallopeptidase domain 28 |
| APOM | ENST00000375920.4 | apolipoprotein M |
| PAFAH1B1 | ENST00000397195.5 | platelet-activating factor acetylhydrolase 1b, regulatory subunit 1 (45kDa) |
| C9orf24 | ENST00000379133.3 | chromosome 9 open reading frame 24 |
| UBR2 | ENST00000372883.3 | ubiquitin protein ligase E3 component n-recognin 2 |
| CD82 | ENST00000227155.4 | CD82 molecule |
| CARD8 | ENST00000520753.1 | caspase recruitment domain family, member 8 |
| GOLGA8K | ENST00000512626.2 | golgin A8 family, member K |
| SLC35B1 | ENST00000240333.6 | solute carrier family 35, member B1 |
| ZNF365 | ENST00000395254.3 | zinc finger protein 365 |
| STK33 | ENST00000447869.1 | serine/threonine kinase 33 |
| ALMS1 | ENST00000264448.6 | Alstrom syndrome 1 |
| NF2 | ENST00000347330.5 | neurofibromin 2 (merlin) |
| SEPT2 | ENST00000391971.2 | septin 2 |
| GOLGA8I | ENST00000450802.3 | golgin A8 family, member I |
| TRPM3 | ENST00000377110.3 | transient receptor potential cation channel, subfamily M, member 3 |
| APOBEC1 | ENST00000229304.4 | apolipoprotein B mRNA editing enzyme, catalytic polypeptide 1 |
| CALB1 | ENST00000265431.3 | calbindin 1, 28kDa |
| MRPL1 | ENST00000315567.8 | mitochondrial ribosomal protein L1 |
| SPIN3 | ENST00000374919.3 | spindlin family, member 3 |
| TCHHL1 | ENST00000368806.1 | trichohyalin-like 1 |
| AC079354.1 | ENST00000295844.3 | uncharacterized protein KIAA2012 |
| C19orf73 | ENST00000408991.2 | chromosome 19 open reading frame 73 |
| GOLGA8N | ENST00000448387.2 | golgin A8 family, member N |
| RAB3B | ENST00000371655.3 | RAB3B, member RAS oncogene family |
| SLC19A3 | ENST00000258403.3 | solute carrier family 19 (thiamine transporter), member 3 |
| TTC31 | ENST00000410003.1 | tetratricopeptide repeat domain 31 |
| RIMBP3C | ENST00000331505.5 | RIMS binding protein 3C |
| RP11-1C1.5 | ENST00000506021.1 | Uncharacterized protein |
| NDUFV3 | ENST00000340344.4 | NADH dehydrogenase (ubiquinone) flavoprotein 3, 10kDa |
| ZG16 | ENST00000400752.4 | zymogen granule protein 16 |
| CCDC41 | ENST00000397809.5 | coiled-coil domain containing 41 |
| DCAF7 | ENST00000310827.4 | DDB1 and CUL4 associated factor 7 |
| GGACT | ENST00000376250.2 | gamma-glutamylamine cyclotransferase |
| CYP3A43 | ENST00000444905.1 | cytochrome P450, family 3, subfamily A, polypeptide 43 |
| ZNF286B | ENST00000285274.5 | zinc finger protein 286B |
| C11orf70 | ENST00000434758.2 | chromosome 11 open reading frame 70 |
| TXLNA | ENST00000373610.3 | taxilin alpha |
| VMAC | ENST00000339485.3 | vimentin-type intermediate filament associated coiled-coil protein |
| ZDHHC15 | ENST00000373367.3 | zinc finger, DHHC-type containing 15 |
| BACH1 | ENST00000286800.3 | BTB and CNC homology 1, basic leucine zipper transcription factor 1 |
| SLPI | ENST00000338380.2 | secretory leukocyte peptidase inhibitor |
| UBXN4 | ENST00000272638.9 | UBX domain protein 4 |
| CDC42SE2 | ENST00000505065.1 | CDC42 small effector 2 |
| SCRN3 | ENST00000272732.6 | secernin 3 |
| GUCY2F | ENST00000218006.2 | guanylate cyclase 2F, retinal |
| THAP1 | ENST00000345117.2 | THAP domain containing, apoptosis associated protein 1 |
| LYNX1 | ENST00000395192.2 | Ly6/neurotoxin 1 |
| GLS2 | ENST00000311966.4 | glutaminase 2 (liver, mitochondrial) |
| TTLL2 | ENST00000239587.5 | tubulin tyrosine ligase-like family, member 2 |
| LRRN1 | ENST00000319331.3 | leucine rich repeat neuronal 1 |
| C12orf36 | ENST00000318426.2 | chromosome 12 open reading frame 36 |
| NAMPT | ENST00000222553.3 | nicotinamide phosphoribosyltransferase |
| GLIPR2 | ENST00000396613.3 | GLI pathogenesis-related 2 |
| PDE6D | ENST00000409772.1 | phosphodiesterase 6D, cGMP-specific, rod, delta |
| LRRC55 | ENST00000497933.1 | leucine rich repeat containing 55 |
| ISL1 | ENST00000230658.7 | ISL LIM homeobox 1 |
| ONECUT1 | ENST00000560699.2 | one cut homeobox 1 |
| TMOD3 | ENST00000308580.7 | tropomodulin 3 (ubiquitous) |
| LHFPL3 | ENST00000535008.1 | lipoma HMGIC fusion partner-like 3 |
| TESPA1 | ENST00000524622.1 | thymocyte expressed, positive selection associated 1 |
| TMEM212 | ENST00000334567.5 | transmembrane protein 212 |
| RPRD1A | ENST00000399022.4 | regulation of nuclear pre-mRNA domain containing 1A |
| FAM211A | ENST00000409083.3 | family with sequence similarity 211, member A |
| REXO2 | ENST00000539275.1 | RNA exonuclease 2 |
| NLRP8 | ENST00000291971.3 | NLR family, pyrin domain containing 8 |
| OSTN | ENST00000445281.1 | osteocrin |
| EMC4 | ENST00000267750.4 | ER membrane protein complex subunit 4 |
| DIRC1 | ENST00000308100.4 | disrupted in renal carcinoma 1 |
| ZFP91 | ENST00000316059.6 | ZFP91 zinc finger protein |
| C17orf80 | ENST00000359042.2 | chromosome 17 open reading frame 80 |
| AIG1 | ENST00000357847.4 | androgen-induced 1 |
| ABHD10 | ENST00000494817.1 | abhydrolase domain containing 10 |
| MAGEB2 | ENST00000378988.4 | melanoma antigen family B, 2 |
| TAS2R3 | ENST00000247879.2 | taste receptor, type 2, member 3 |
| ST13 | ENST00000216218.3 | suppression of tumorigenicity 13 (colon carcinoma) (Hsp70 interacting protein) |
| RAB11FIP2 | ENST00000355624.3 | RAB11 family interacting protein 2 (class I) |
| SLC35F2 | ENST00000525071.1 | solute carrier family 35, member F2 |
| ACOX2 | ENST00000459701.2 | acyl-CoA oxidase 2, branched chain |
| PSMD10 | ENST00000372296.1 | proteasome (prosome, macropain) 26S subunit, non-ATPase, 10 |
| PCTP | ENST00000576183.1 | phosphatidylcholine transfer protein |
| FAM43B | ENST00000332947.4 | family with sequence similarity 43, member B |
| TMLHE | ENST00000334398.3 | trimethyllysine hydroxylase, epsilon |
| PCDH11X | ENST00000504220.2 | protocadherin 11 X-linked |
| WHSC1L1 | ENST00000317025.8 | Wolf-Hirschhorn syndrome candidate 1-like 1 |
| MTTP | ENST00000457717.1 | microsomal triglyceride transfer protein |
| ITPR2 | ENST00000381340.3 | inositol 1,4,5-trisphosphate receptor, type 2 |
| DDX3X | ENST00000399959.2 | DEAD (Asp-Glu-Ala-Asp) box helicase 3, X-linked |
| TRIP6 | ENST00000200457.4 | thyroid hormone receptor interactor 6 |
| DHFRL1 | ENST00000314636.2 | dihydrofolate reductase-like 1 |
| KLK8 | ENST00000320838.5 | kallikrein-related peptidase 8 |
| YARS2 | ENST00000324868.8 | tyrosyl-tRNA synthetase 2, mitochondrial |
| CCDC144A | ENST00000443444.2 | coiled-coil domain containing 144A |
| RAD54L | ENST00000442598.1 | RAD54-like (S. cerevisiae) |
| CDNF | ENST00000378442.1 | cerebral dopamine neurotrophic factor |
| C3orf67 | ENST00000491845.1 | chromosome 3 open reading frame 67 |
| TFEC | ENST00000265440.7 | transcription factor EC |
| HRSP12 | ENST00000254878.3 | heat-responsive protein 12 |
| SALL2 | ENST00000327430.3 | sal-like 2 (Drosophila) |
| C14orf37 | ENST00000267485.7 | chromosome 14 open reading frame 37 |
| ADSS | ENST00000366535.3 | adenylosuccinate synthase |
| MPZ | ENST00000533357.1 | myelin protein zero |
| STAR | ENST00000276449.4 | steroidogenic acute regulatory protein |
| AADACL4 | ENST00000376221.1 | arylacetamide deacetylase-like 4 |
| CYP2J2 | ENST00000371204.3 | cytochrome P450, family 2, subfamily J, polypeptide 2 |
| KRT80 | ENST00000313234.5 | keratin 80 |
| NEU3 | ENST00000532963.1 | sialidase 3 (membrane sialidase) |
| SAMD10 | ENST00000369886.3 | sterile alpha motif domain containing 10 |
| CCZ1B | ENST00000316731.8 | CCZ1 vacuolar protein trafficking and biogenesis associated homolog B (S. cerevisiae) |
| GLO1 | ENST00000373365.4 | glyoxalase I |
| SLC6A15 | ENST00000309283.7 | solute carrier family 6 (neutral amino acid transporter), member 15 |
| CCDC39 | ENST00000273654.4 | coiled-coil domain containing 39 |
| POSTN | ENST00000541179.1 | periostin, osteoblast specific factor |
| TDRKH | ENST00000368825.3 | tudor and KH domain containing |
| TMEM98 | ENST00000579849.1 | transmembrane protein 98 |
| GYG1 | ENST00000345003.4 | glycogenin 1 |
| IGFBP3 | ENST00000381086.5 | insulin-like growth factor binding protein 3 |
| RASD1 | ENST00000579152.1 | RAS, dexamethasone-induced 1 |
| HMGCLL1 | ENST00000274901.4 | 3-hydroxymethyl-3-methylglutaryl-CoA lyase-like 1 |
| KLHL23 | ENST00000392647.2 | kelch-like family member 23 |
| RNF24 | ENST00000336095.6 | ring finger protein 24 |
| JAM3 | ENST00000299106.4 | junctional adhesion molecule 3 |
| ZNF267 | ENST00000300870.10 | zinc finger protein 267 |
| PDE8B | ENST00000264917.5 | phosphodiesterase 8B |
| CEACAM8 | ENST00000244336.5 | carcinoembryonic antigen-related cell adhesion molecule 8 |
| FAM208A | ENST00000493960.2 | family with sequence similarity 208, member A |
| PCDH9 | ENST00000377861.3 | protocadherin 9 |
| MS4A1 | ENST00000534668.1 | membrane-spanning 4-domains, subfamily A, member 1 |
| FAM46A | ENST00000369754.3 | family with sequence similarity 46, member A |
| CADM2 | ENST00000383699.3 | cell adhesion molecule 2 |
| DSCR4 | ENST00000398948.1 | Down syndrome critical region gene 4 |
| ZNF605 | ENST00000360187.4 | zinc finger protein 605 |
| GLCCI1 | ENST00000223145.5 | glucocorticoid induced transcript 1 |
| BOD1 | ENST00000311086.4 | biorientation of chromosomes in cell division 1 |
| EXO5 | ENST00000296380.4 | exonuclease 5 |
| ATP6V1G1 | ENST00000374050.3 | ATPase, H+ transporting, lysosomal 13kDa, V1 subunit G1 |
| RPP14 | ENST00000445193.3 | ribonuclease P/MRP 14kDa subunit |
| NLRP11 | ENST00000589824.2 | NLR family, pyrin domain containing 11 |
| BRCA2 | ENST00000544455.1 | breast cancer 2, early onset |
| KIAA1841 | ENST00000402291.1 | KIAA1841 |
| ZNF195 | ENST00000438262.2 | zinc finger protein 195 |
| PPP2R3A | ENST00000264977.3 | protein phosphatase 2, regulatory subunit B'', alpha |
| DYNLT3 | ENST00000378581.3 | dynein, light chain, Tctex-type 3 |
| KRT24 | ENST00000264651.2 | keratin 24 |
| GOLT1A | ENST00000308302.3 | golgi transport 1A |
| ELP4 | ENST00000395934.2 | elongator acetyltransferase complex subunit 4 |
| DIMT1 | ENST00000199320.4 | DIM1 dimethyladenosine transferase 1 homolog (S. cerevisiae) |
| XAF1 | ENST00000361842.3 | XIAP associated factor 1 |
| PEX5L | ENST00000467460.1 | peroxisomal biogenesis factor 5-like |
| PRRG1 | ENST00000378628.4 | proline rich Gla (G-carboxyglutamic acid) 1 |
| SOX7 | ENST00000554914.1 | Transcription factor SOX-7; Uncharacterized protein; cDNA FLJ58508, highly similar to Transcription factor SOX-7 |
| NFE2L3 | ENST00000056233.3 | nuclear factor, erythroid 2-like 3 |
| FAM83F | ENST00000333407.6 | family with sequence similarity 83, member F |
| CLIP3 | ENST00000360535.4 | CAP-GLY domain containing linker protein 3 |
| MARCH1 | ENST00000274056.7 | membrane-associated ring finger (C3HC4) 1, E3 ubiquitin protein ligase |
| POU5F2 | ENST00000510627.4 | POU domain class 5, transcription factor 2 |
| SOGA3 | ENST00000556132.1 | SOGA family member 3 |
| C16orf95 | ENST00000253461.4 | chromosome 16 open reading frame 95 |
| MPV17L | ENST00000396385.3 | MPV17 mitochondrial membrane protein-like |
| AC119673.1 | ENST00000598338.1 |  |
| LECT2 | ENST00000274507.1 | leukocyte cell-derived chemotaxin 2 |
| UBE2W | ENST00000517608.1 | ubiquitin-conjugating enzyme E2W (putative) |
| OTC | ENST00000039007.4 | ornithine carbamoyltransferase |
| HLA-DQB1 | ENST00000399082.3 | major histocompatibility complex, class II, DQ beta 1 |
| DFFA | ENST00000377038.3 | DNA fragmentation factor, 45kDa, alpha polypeptide |
| C16orf87 | ENST00000394806.2 | chromosome 16 open reading frame 87 |
| AKR1C2 | ENST00000380753.4 | aldo-keto reductase family 1, member C2 |
| RP11-15E18.4 | ENST00000592917.1 | Uncharacterized protein |
| CRK | ENST00000398970.5 | v-crk avian sarcoma virus CT10 oncogene homolog |
| HIATL2 | ENST00000602917.1 | hippocampus abundant transcript-like 2 |
| MS4A14 | ENST00000531787.1 | membrane-spanning 4-domains, subfamily A, member 14 |
| FAM178A | ENST00000238961.4 | family with sequence similarity 178, member A |
| PAQR8 | ENST00000442253.2 | progestin and adipoQ receptor family member VIII |
| WDR82 | ENST00000296490.3 | WD repeat domain 82 |
| ZNF705B | ENST00000443676.1 | zinc finger protein 705B |
| SUN3 | ENST00000297325.4 | Sad1 and UNC84 domain containing 3 |
| IGF1 | ENST00000337514.6 | insulin-like growth factor 1 (somatomedin C) |
| PURB | ENST00000395699.2 | purine-rich element binding protein B |
| RAB1A | ENST00000409892.1 | RAB1A, member RAS oncogene family |
| C8orf4 | ENST00000315792.3 | chromosome 8 open reading frame 4 |
| C14orf159 | ENST00000256324.10 | chromosome 14 open reading frame 159 |
| SCIMP | ENST00000574081.1 | SLP adaptor and CSK interacting membrane protein |
| FAM179B | ENST00000382233.2 | family with sequence similarity 179, member B |
| RNF157 | ENST00000269391.6 | ring finger protein 157 |
| PPIL1 | ENST00000373699.5 | peptidylprolyl isomerase (cyclophilin)-like 1 |
| SRP14 | ENST00000267884.6 | signal recognition particle 14kDa (homologous Alu RNA binding protein) |
| SPAM1 | ENST00000340011.5 | sperm adhesion molecule 1 (PH-20 hyaluronidase, zona pellucida binding) |
| MFSD4 | ENST00000367147.4 | major facilitator superfamily domain containing 4 |
| OTUD7B | ENST00000369135.4 | OTU domain containing 7B |
| RSG1 | ENST00000375599.3 | REM2 and RAB-like small GTPase 1 |
| ZIK1 | ENST00000307468.4 | zinc finger protein interacting with K protein 1 |
| EREG | ENST00000244869.2 | epiregulin |
| GUCA2B | ENST00000372581.1 | guanylate cyclase activator 2B (uroguanylin) |
| C5orf64 | ENST00000505642.1 | chromosome 5 open reading frame 64 |
| UCMA | ENST00000378681.3 | upper zone of growth plate and cartilage matrix associated |
| PAPOLB | ENST00000404991.1 | poly(A) polymerase beta (testis specific) |
| SLCO4C1 | ENST00000310954.6 | solute carrier organic anion transporter family, member 4C1 |
| ZNF415 | ENST00000597503.1 | zinc finger protein 415 |
| CXorf28 | ENST00000457435.1 | chromosome X open reading frame 28 |
| RAB3D | ENST00000222120.3 | RAB3D, member RAS oncogene family |
| TAB2 | ENST00000367456.1 | TGF-beta activated kinase 1/MAP3K7 binding protein 2 |
| C10orf2 | ENST00000370228.1 | chromosome 10 open reading frame 2 |
| HSD11B2 | ENST00000326152.5 | hydroxysteroid (11-beta) dehydrogenase 2 |
| DRAM2 | ENST00000286692.4 | DNA-damage regulated autophagy modulator 2 |
| HFM1 | ENST00000294696.5 | HFM1, ATP-dependent DNA helicase homolog (S. cerevisiae) |
| C8orf22 | ENST00000303202.8 | chromosome 8 open reading frame 22 |
| CCDC74A | ENST00000467992.2 | coiled-coil domain containing 74A |
| AMACR | ENST00000335606.6 | alpha-methylacyl-CoA racemase |
| RP11-178L8.4 | ENST00000568879.1 |  |
| ZNF233 | ENST00000592581.1 | zinc finger protein 233 |
| ATP6V1G3 | ENST00000309309.7 | ATPase, H+ transporting, lysosomal 13kDa, V1 subunit G3 |
| SLC2A1 | ENST00000426263.3 | solute carrier family 2 (facilitated glucose transporter), member 1 |
| PLCXD1 | ENST00000381657.2 | phosphatidylinositol-specific phospholipase C, X domain containing 1 |
| ACSL6 | ENST00000379264.2 | acyl-CoA synthetase long-chain family member 6 |
| HEPACAM | ENST00000298251.4 | hepatic and glial cell adhesion molecule |
| C8orf31 | ENST00000395172.1 | chromosome 8 open reading frame 31 |
| ATE1 | ENST00000369043.3 | arginyltransferase 1 |
| TARBP2 | ENST00000552857.1 | TAR (HIV-1) RNA binding protein 2 |
| FOXA1 | ENST00000250448.2 | forkhead box A1 |
| FLJ20306 | ENST00000601673.1 | CDNA FLJ20306 fis, clone HEP06881; Putative uncharacterized protein FLJ20306; Uncharacterized protein |
| TREML2 | ENST00000483722.1 | triggering receptor expressed on myeloid cells-like 2 |
| CNTROB | ENST00000380255.3 | centrobin, centrosomal BRCA2 interacting protein |
| PRMT5 | ENST00000324366.8 | protein arginine methyltransferase 5 |
| KRT72 | ENST00000293745.2 | keratin 72 |
| SGOL2 | ENST00000357799.4 | shugoshin-like 2 (S. pombe) |
| ALPP | ENST00000392027.2 | alkaline phosphatase, placental |
| DAZ3 | ENST00000382365.2 | deleted in azoospermia 3 |
| TMEM154 | ENST00000304385.3 | transmembrane protein 154 |
| ZNF705D | ENST00000400085.3 | zinc finger protein 705D |
| TIMM8A | ENST00000372902.3 | translocase of inner mitochondrial membrane 8 homolog A (yeast) |
| L3MBTL3 | ENST00000529410.1 | l(3)mbt-like 3 (Drosophila) |
| APOBEC3G | ENST00000407997.3 | apolipoprotein B mRNA editing enzyme, catalytic polypeptide-like 3G |
| HHLA1 | ENST00000434736.2 | HERV-H LTR-associating 1 |
| MARVELD2 | ENST00000325631.5 | MARVEL domain containing 2 |
| PCP4 | ENST00000328619.5 | Purkinje cell protein 4 |
| CETN1 | ENST00000327228.3 | centrin, EF-hand protein, 1 |
| GLRX | ENST00000379979.4 | glutaredoxin (thioltransferase) |
| PPARG | ENST00000397000.1 | peroxisome proliferator-activated receptor gamma |
| FYB | ENST00000351578.6 | FYN binding protein |
| ZNF740 | ENST00000416904.3 | zinc finger protein 740 |
| NSUN2 | ENST00000264670.6 | NOP2/Sun RNA methyltransferase family, member 2 |
| KCNB1 | ENST00000371741.4 | potassium voltage-gated channel, Shab-related subfamily, member 1 |
| ZNF705A | ENST00000359286.4 | zinc finger protein 705A |
| C9orf66 | ENST00000382387.2 | chromosome 9 open reading frame 66 |
| FAS | ENST00000352159.4 | Fas cell surface death receptor |
| CDC73 | ENST00000367435.3 | cell division cycle 73 |
| USP36 | ENST00000542802.3 | ubiquitin specific peptidase 36 |
| ZNF460 | ENST00000360338.3 | zinc finger protein 460 |
| MPHOSPH6 | ENST00000258169.4 | M-phase phosphoprotein 6 |
| TMX4 | ENST00000246024.2 | thioredoxin-related transmembrane protein 4 |
| SCGB2A2 | ENST00000525380.1 | secretoglobin, family 2A, member 2 |
| RP11-382J12.1 | ENST00000499227.2 | Uncharacterized protein |
| PARP3 | ENST00000431474.1 | poly (ADP-ribose) polymerase family, member 3 |
| CLCA4 | ENST00000370563.3 | chloride channel accessory 4 |
| CXCR5 | ENST00000292174.4 | chemokine (C-X-C motif) receptor 5 |
| PICALM | ENST00000532317.1 | phosphatidylinositol binding clathrin assembly protein |
| U2SURP | ENST00000397933.2 | U2 snRNP-associated SURP domain containing |
| SELK | ENST00000541726.1 | Selenoprotein K |
| C16orf45 | ENST00000300006.4 | chromosome 16 open reading frame 45 |
| ZNF830 | ENST00000361952.3 | zinc finger protein 830 |
| THAP4 | ENST00000402136.1 | THAP domain containing 4 |
| CADM3 | ENST00000368125.4 | cell adhesion molecule 3 |
| CSF2RA | ENST00000381509.3 | colony stimulating factor 2 receptor, alpha, low-affinity (granulocyte-macrophage) |
| PFDN1 | ENST00000510217.1 | prefoldin subunit 1 |
| DAZ2 | ENST00000382449.1 | deleted in azoospermia 2 |
| RP11-724O16.1 | ENST00000513963.1 | Bardet-Biedl syndrome 5 protein; Uncharacterized protein |
| PCNXL4 | ENST00000535349.1 | pecanex-like 4 (Drosophila) |
| PCBP1 | ENST00000303577.5 | poly(rC) binding protein 1 |
| IRS2 | ENST00000375856.3 | insulin receptor substrate 2 |
| APPL1 | ENST00000288266.3 | adaptor protein, phosphotyrosine interaction, PH domain and leucine zipper containing 1 |
| CLCN4 | ENST00000380833.4 | chloride channel, voltage-sensitive 4 |
| NFATC3 | ENST00000329524.4 | nuclear factor of activated T-cells, cytoplasmic, calcineurin-dependent 3 |
| GOLGA1 | ENST00000373555.4 | golgin A1 |
| SLC25A31 | ENST00000281154.4 | solute carrier family 25 (mitochondrial carrier; adenine nucleotide translocator), member 31 |
| DAP | ENST00000230895.6 | death-associated protein |
| EID3 | ENST00000527879.1 | EP300 interacting inhibitor of differentiation 3 |
| SGPP2 | ENST00000321276.7 | sphingosine-1-phosphate phosphatase 2 |
| NAA20 | ENST00000310450.4 | N(alpha)-acetyltransferase 20, NatB catalytic subunit |
| LRAT | ENST00000336356.3 | lecithin retinol acyltransferase (phosphatidylcholine--retinol O-acyltransferase) |
| ARHGEF35 | ENST00000378115.2 | Rho guanine nucleotide exchange factor (GEF) 35 |
| COX17 | ENST00000261070.2 | COX17 cytochrome c oxidase copper chaperone |
| ZNF41 | ENST00000313116.7 | zinc finger protein 41 |
| LGALSL | ENST00000409537.2 | lectin, galactoside-binding-like |
| MRPL18 | ENST00000367034.4 | mitochondrial ribosomal protein L18 |
| TMEM60 | ENST00000257663.3 | transmembrane protein 60 |
| LDHC | ENST00000546146.1 | lactate dehydrogenase C |
| WDR49 | ENST00000479765.1 | WD repeat domain 49 |
| SMIM7 | ENST00000487416.2 | small integral membrane protein 7 |
| PCYT1B | ENST00000379145.1 | phosphate cytidylyltransferase 1, choline, beta |
| LHCGR | ENST00000294954.7 | luteinizing hormone/choriogonadotropin receptor |
| DDX1 | ENST00000381341.2 | DEAD (Asp-Glu-Ala-Asp) box helicase 1 |
| SYNDIG1 | ENST00000376862.3 | synapse differentiation inducing 1 |
| OCIAD1 | ENST00000396448.2 | OCIA domain containing 1 |
| CS | ENST00000548567.1 | citrate synthase |
| ZCCHC7 | ENST00000336755.5 | zinc finger, CCHC domain containing 7 |
| ARL8B | ENST00000419534.2 | ADP-ribosylation factor-like 8B |
| CTB-96E2.2 | ENST00000555059.2 | Uncharacterized protein |
| POLR1A | ENST00000263857.6 | polymerase (RNA) I polypeptide A, 194kDa |
| IDS | ENST00000422081.2 | Iduronate 2-sulfatase (Hunter syndrome), isoform CRA_e; Iduronate 2-sulfatase 14 kDa chain; cDNA FLJ42669 fis, clone BRAMY2022168, highly similar to IDURONATE 2-SULFATASE |
| RILPL2 | ENST00000280571.8 | Rab interacting lysosomal protein-like 2 |
| VIP | ENST00000367244.3 | vasoactive intestinal peptide |
| PAH | ENST00000553106.1 | phenylalanine hydroxylase |
| C1orf61 | ENST00000368243.1 | chromosome 1 open reading frame 61 |
| FUT9 | ENST00000302103.5 | fucosyltransferase 9 (alpha (1,3) fucosyltransferase) |
| LPPR1 | ENST00000374874.3 | Lipid phosphate phosphatase-related protein type 1 |
| IFI30 | ENST00000407280.3 | interferon, gamma-inducible protein 30 |
| WDR16 | ENST00000352665.5 | WD repeat domain 16 |
| PLEKHG7 | ENST00000344636.3 | pleckstrin homology domain containing, family G (with RhoGef domain) member 7 |
| RAB42 | ENST00000373826.3 | RAB42, member RAS oncogene family |
| ACADL | ENST00000233710.3 | acyl-CoA dehydrogenase, long chain |
| ZNF623 | ENST00000501748.2 | zinc finger protein 623 |
| TM6SF1 | ENST00000379390.6 | transmembrane 6 superfamily member 1 |
| SCG2 | ENST00000305409.2 | secretogranin II |
| SCRG1 | ENST00000296506.3 | stimulator of chondrogenesis 1 |
| NPM2 | ENST00000381530.5 | nucleophosmin/nucleoplasmin 2 |
| EPOR | ENST00000592375.2 | erythropoietin receptor |
| C20orf194 | ENST00000453730.2 | chromosome 20 open reading frame 194 |
| DPH1 | ENST00000263083.6 | diphthamide biosynthesis 1 |
| TLR4 | ENST00000355622.6 | toll-like receptor 4 |
| KCNG4 | ENST00000308251.4 | potassium voltage-gated channel, subfamily G, member 4 |
| TMPRSS4 | ENST00000534111.1 | transmembrane protease, serine 4 |
| INTS7 | ENST00000366994.3 | integrator complex subunit 7 |
| MFAP1 | ENST00000267812.3 | microfibrillar-associated protein 1 |
| CD99L2 | ENST00000370377.3 | CD99 molecule-like 2 |
| ENHO | ENST00000399775.2 | energy homeostasis associated |
| GPRC5C | ENST00000481232.1 | G protein-coupled receptor, family C, group 5, member C |
| GTF2IRD2B | ENST00000312575.7 | GTF2I repeat domain containing 2B |
| NLRC3 | ENST00000448023.2 | NLR family, CARD domain containing 3 |
| ABHD6 | ENST00000295962.4 | abhydrolase domain containing 6 |
| MCC | ENST00000302475.4 | mutated in colorectal cancers |
| SENP8 | ENST00000544411.1 | SUMO/sentrin specific peptidase family member 8 |
| DMTF1 | ENST00000413276.2 | cyclin D binding myb-like transcription factor 1 |
| ZC2HC1C | ENST00000238686.8 | zinc finger, C2HC-type containing 1C |
| GTF2IRD2 | ENST00000405086.2 | GTF2I repeat domain containing 2 |
| SMIM3 | ENST00000526627.1 | small integral membrane protein 3 |
| KPNA4 | ENST00000334256.4 | karyopherin alpha 4 (importin alpha 3) |
| FOXR2 | ENST00000339140.3 | forkhead box R2 |
| LEKR1 | ENST00000491763.1 | leucine, glutamate and lysine rich 1 |
| CHST9 | ENST00000580774.1 | carbohydrate (N-acetylgalactosamine 4-0) sulfotransferase 9 |
| HTATIP2 | ENST00000451739.2 | HIV-1 Tat interactive protein 2, 30kDa |
| PRKG1 | ENST00000373985.1 | protein kinase, cGMP-dependent, type I |
| OPHN1 | ENST00000355520.5 | oligophrenin 1 |
| UGT2B10 | ENST00000265403.7 | UDP glucuronosyltransferase 2 family, polypeptide B10 |
| C12orf56 | ENST00000543942.2 | chromosome 12 open reading frame 56 |
| ZFP28 | ENST00000301318.3 | ZFP28 zinc finger protein |
| SLC44A2 | ENST00000586078.1 | solute carrier family 44 (choline transporter), member 2 |
| TRIB2 | ENST00000155926.4 | tribbles pseudokinase 2 |
| GPR89B | ENST00000314163.7 | G protein-coupled receptor 89B |
| OCA2 | ENST00000353809.5 | oculocutaneous albinism II |
| HN1L | ENST00000248098.3 | hematological and neurological expressed 1-like |
| SIK2 | ENST00000304987.3 | salt-inducible kinase 2 |
| GTF3C6 | ENST00000329970.7 | general transcription factor IIIC, polypeptide 6, alpha 35kDa |
| C13orf35 | ENST00000356049.1 | chromosome 13 open reading frame 35 |
| ABCC9 | ENST00000261200.4 | ATP-binding cassette, sub-family C (CFTR/MRP), member 9 |
| DHRS9 | ENST00000602501.1 | dehydrogenase/reductase (SDR family) member 9 |
| ZNF564 | ENST00000339282.7 | zinc finger protein 564 |
| RCOR1 | ENST00000262241.6 | REST corepressor 1 |
| CDC5L | ENST00000371477.3 | cell division cycle 5-like |
| AOX1 | ENST00000374700.2 | aldehyde oxidase 1 |
| NSF | ENST00000225282.8 | N-ethylmaleimide-sensitive factor |
| TRMT61B | ENST00000306108.5 | tRNA methyltransferase 61 homolog B (S. cerevisiae) |
| CD5L | ENST00000368174.4 | CD5 molecule-like |
| DAZ1 | ENST00000405239.1 | deleted in azoospermia 1 |
| SLC22A4 | ENST00000200652.3 | solute carrier family 22 (organic cation/zwitterion transporter), member 4 |
| GMPPB | ENST00000480687.1 | GDP-mannose pyrophosphorylase B |
| COMMD8 | ENST00000381571.4 | COMM domain containing 8 |
| ZNF24 | ENST00000589881.1 | zinc finger protein 24 |
| FAM151B | ENST00000282226.4 | family with sequence similarity 151, member B |
| PPAT | ENST00000264220.2 | phosphoribosyl pyrophosphate amidotransferase |
| MAGEB1 | ENST00000378981.3 | melanoma antigen family B, 1 |
| GSG1L | ENST00000380898.2 | GSG1-like |
| OMG | ENST00000247271.4 | oligodendrocyte myelin glycoprotein |
| ZNF852 | ENST00000436261.1 | zinc finger protein 852 |
| CYP2C18 | ENST00000285979.6 | cytochrome P450, family 2, subfamily C, polypeptide 18 |
| FASN | ENST00000306749.2 | fatty acid synthase |
| SDCBP2 | ENST00000381808.3 | syndecan binding protein (syntenin) 2 |
| KLHL41 | ENST00000284669.1 | kelch-like family member 41 |
| CCDC47 | ENST00000225726.5 | coiled-coil domain containing 47 |
| BMPR1A | ENST00000372037.3 | bone morphogenetic protein receptor, type IA |
| PDRG1 | ENST00000202017.4 | p53 and DNA-damage regulated 1 |
| AP3B2 | ENST00000542200.1 | adaptor-related protein complex 3, beta 2 subunit |
| CDH11 | ENST00000394156.3 | cadherin 11, type 2, OB-cadherin (osteoblast) |
| AC069547.1 | ENST00000595931.1 | HCG1745369; PRO3073; Uncharacterized protein |
| SUMO2 | ENST00000420826.2 | small ubiquitin-like modifier 2 |
| GBAS | ENST00000322090.3 | glioblastoma amplified sequence |
| ZNF248 | ENST00000395867.3 | zinc finger protein 248 |
| AKAP1 | ENST00000337714.3 | A kinase (PRKA) anchor protein 1 |
| C10orf25 | ENST00000298298.1 | chromosome 10 open reading frame 25 |
| PKD2 | ENST00000237596.2 | polycystic kidney disease 2 (autosomal dominant) |
| NRBF2 | ENST00000277746.6 | nuclear receptor binding factor 2 |
| FGD4 | ENST00000427716.2 | FYVE, RhoGEF and PH domain containing 4 |
| POU2F1 | ENST00000367866.2 | POU class 2 homeobox 1 |
| CISD2 | ENST00000273986.4 | CDGSH iron sulfur domain 2 |
| CCKBR | ENST00000334619.2 | cholecystokinin B receptor |
| LDLRAD3 | ENST00000315571.5 | low density lipoprotein receptor class A domain containing 3 |
| NELFCD | ENST00000602795.1 | negative elongation factor complex member C/D |
| ARFIP2 | ENST00000254584.2 | ADP-ribosylation factor interacting protein 2 |
| SLC1A4 | ENST00000234256.3 | solute carrier family 1 (glutamate/neutral amino acid transporter), member 4 |
| BTC | ENST00000395743.3 | betacellulin |
| ZNF584 | ENST00000599238.1 | zinc finger protein 584 |
| NTN1 | ENST00000173229.2 | netrin 1 |
| CKS1B | ENST00000308987.5 | CDC28 protein kinase regulatory subunit 1B |
| CCNJ | ENST00000265992.5 | cyclin J |
| ULBP1 | ENST00000229708.3 | UL16 binding protein 1 |
| CYP3A7 | ENST00000336374.2 | cytochrome P450, family 3, subfamily A, polypeptide 7 |
| FGF5 | ENST00000456523.3 | fibroblast growth factor 5 |
| MSMO1 | ENST00000261507.6 | methylsterol monooxygenase 1 |
| HTR2A | ENST00000378688.4 | 5-hydroxytryptamine (serotonin) receptor 2A, G protein-coupled |
| CDX1 | ENST00000231656.8 | caudal type homeobox 1 |
| CCDC180 | ENST00000395220.1 | coiled-coil domain containing 180 |
| WNT8B | ENST00000343737.5 | wingless-type MMTV integration site family, member 8B |
| RBM3 | ENST00000354480.2 | RNA binding motif (RNP1, RRM) protein 3 |
| FAM126A | ENST00000409923.1 | family with sequence similarity 126, member A |
| NAP1L2 | ENST00000373517.3 | nucleosome assembly protein 1-like 2 |
| PTP4A2 | ENST00000602725.1 | protein tyrosine phosphatase type IVA, member 2 |
| ZSCAN21 | ENST00000292450.4 | zinc finger and SCAN domain containing 21 |
| FMR1NB | ENST00000370467.3 | fragile X mental retardation 1 neighbor |
| ZNF714 | ENST00000596053.1 | zinc finger protein 714 |
| RBM8A | ENST00000330165.8 | RNA binding motif protein 8A |
| TBL1X | ENST00000407597.2 | transducin (beta)-like 1X-linked |
| ACADVL | ENST00000356839.5 | acyl-CoA dehydrogenase, very long chain |
| PRPS2 | ENST00000380668.5 | phosphoribosyl pyrophosphate synthetase 2 |
| COPZ1 | ENST00000551779.1 | coatomer protein complex, subunit zeta 1 |
| FLJ30594 | ENST00000391666.2 | HCG1990367; Putative uncharacterized protein DKFZp761K2322; Putative uncharacterized protein FLJ30594; cDNA FLJ30594 fis, clone BRAWH2008903 |
| PRPH2 | ENST00000230381.5 | peripherin 2 (retinal degeneration, slow) |
| LHFPL4 | ENST00000287585.6 | lipoma HMGIC fusion partner-like 4 |
| PAFAH2 | ENST00000374282.3 | platelet-activating factor acetylhydrolase 2, 40kDa |
| CTNND1 | ENST00000524630.1 | catenin (cadherin-associated protein), delta 1 |
| UBE2D2 | ENST00000398733.3 | ubiquitin-conjugating enzyme E2D 2 |
| ZPBP2 | ENST00000377940.3 | zona pellucida binding protein 2 |
| ZSCAN32 | ENST00000574940.1 | zinc finger and SCAN domain containing 32 |
| GDPD1 | ENST00000284116.4 | glycerophosphodiester phosphodiesterase domain containing 1 |
| ATG14 | ENST00000247178.5 | autophagy related 14 |
| IL7R | ENST00000343305.4 | interleukin 7 receptor |
| COA6 | ENST00000366615.4 | cytochrome c oxidase assembly factor 6 homolog (S. cerevisiae) |
| C7orf76 | ENST00000356686.1 | chromosome 7 open reading frame 76 |
| HS6ST2 | ENST00000370836.2 | heparan sulfate 6-O-sulfotransferase 2 |
| MAP7D2 | ENST00000379651.3 | MAP7 domain containing 2 |
| VASH1 | ENST00000167106.4 | vasohibin 1 |
| KDM4A | ENST00000372396.3 | lysine (K)-specific demethylase 4A |
| CDKL1 | ENST00000395834.1 | cyclin-dependent kinase-like 1 (CDC2-related kinase) |
| DUSP19 | ENST00000354221.4 | dual specificity phosphatase 19 |
| BAIAP2L1 | ENST00000005260.8 | BAI1-associated protein 2-like 1 |
| PNPT1 | ENST00000447944.2 | polyribonucleotide nucleotidyltransferase 1 |
| FBXL4 | ENST00000369244.2 | F-box and leucine-rich repeat protein 4 |
| CYP3A4 | ENST00000354593.2 | cytochrome P450, family 3, subfamily A, polypeptide 4 |
| DPYD | ENST00000370192.3 | dihydropyrimidine dehydrogenase |
| AGO3 | ENST00000373191.4 | argonaute RISC catalytic component 3 |
| GPR75 | ENST00000394705.2 | G protein-coupled receptor 75 |
| ATP5D | ENST00000215375.2 | ATP synthase, H+ transporting, mitochondrial F1 complex, delta subunit |
| CCDC18 | ENST00000334652.5 | coiled-coil domain containing 18 |
| IL18 | ENST00000280357.7 | interleukin 18 (interferon-gamma-inducing factor) |
| TBATA | ENST00000299290.1 | thymus, brain and testes associated |
| IL15 | ENST00000296545.7 | interleukin 15 |
| SYTL5 | ENST00000357972.5 | synaptotagmin-like 5 |
| ALKBH8 | ENST00000428149.2 | alkB, alkylation repair homolog 8 (E. coli) |
| METTL10 | ENST00000368836.2 | methyltransferase like 10 |
| TMOD2 | ENST00000249700.4 | tropomodulin 2 (neuronal) |
| C14orf1 | ENST00000256319.6 | chromosome 14 open reading frame 1 |
| BTBD19 | ENST00000453418.1 | BTB (POZ) domain containing 19 |
| MNS1 | ENST00000260453.3 | meiosis-specific nuclear structural 1 |
| RANBP1 | ENST00000430524.1 | RAN binding protein 1 |
| ZMYND10 | ENST00000231749.3 | zinc finger, MYND-type containing 10 |
| LYSMD1 | ENST00000368908.5 | LysM, putative peptidoglycan-binding, domain containing 1 |
| UNC80 | ENST00000439458.1 | unc-80 homolog (C. elegans) |
| NPTX2 | ENST00000265634.3 | neuronal pentraxin II |
| LPL | ENST00000311322.8 | lipoprotein lipase |
| ERO1LB | ENST00000354619.5 | ERO1-like beta (S. cerevisiae) |
| CDH7 | ENST00000397968.2 | cadherin 7, type 2 |
| TNFSF15 | ENST00000374045.4 | tumor necrosis factor (ligand) superfamily, member 15 |
| C12orf77 | ENST00000549828.1 | chromosome 12 open reading frame 77 |
| TRAF1 | ENST00000373887.3 | TNF receptor-associated factor 1 |
| MGAT5 | ENST00000409645.1 | mannosyl (alpha-1,6-)-glycoprotein beta-1,6-N-acetyl-glucosaminyltransferase |
| ARPC3 | ENST00000228825.7 | actin related protein 2/3 complex, subunit 3, 21kDa |
| POU6F2 | ENST00000518318.2 | POU class 6 homeobox 2 |
| CTC-534A2.2 | ENST00000510585.2 | CDNA FLJ26957 fis, clone SLV00486; Uncharacterized protein |
| CXorf67 | ENST00000342995.2 | chromosome X open reading frame 67 |
| DLGAP5 | ENST00000395425.2 | discs, large (Drosophila) homolog-associated protein 5 |
| STX11 | ENST00000367568.4 | syntaxin 11 |
| C18orf54 | ENST00000300091.5 | chromosome 18 open reading frame 54 |
| CASP9 | ENST00000546424.1 | caspase 9, apoptosis-related cysteine peptidase |
| ST8SIA1 | ENST00000396037.4 | ST8 alpha-N-acetyl-neuraminide alpha-2,8-sialyltransferase 1 |
| CDH17 | ENST00000027335.3 | cadherin 17, LI cadherin (liver-intestine) |
| FAM154B | ENST00000339465.5 | family with sequence similarity 154, member B |
| FBXL13 | ENST00000393772.2 | F-box and leucine-rich repeat protein 13 |
| KANK2 | ENST00000586659.1 | KN motif and ankyrin repeat domains 2 |
| XG | ENST00000426774.1 | Xg blood group |
| GHR | ENST00000230882.4 | growth hormone receptor |
| GPR89A | ENST00000313835.9 | G protein-coupled receptor 89A |
| NOB1 | ENST00000268802.5 | NIN1/RPN12 binding protein 1 homolog (S. cerevisiae) |
| TNR | ENST00000367674.2 | tenascin R |
| HSD17B13 | ENST00000302219.6 | hydroxysteroid (17-beta) dehydrogenase 13 |
| ZNF439 | ENST00000304030.2 | zinc finger protein 439 |
| CCND2 | ENST00000261254.3 | cyclin D2 |
| TRDN | ENST00000398178.3 | triadin |
| RBM43 | ENST00000331426.5 | RNA binding motif protein 43 |
| SLC39A6 | ENST00000269187.5 | solute carrier family 39 (zinc transporter), member 6 |
| YWHAH | ENST00000397492.1 | tyrosine 3-monooxygenase/tryptophan 5-monooxygenase activation protein, eta polypeptide |
| TMEM97 | ENST00000226230.6 | transmembrane protein 97 |
| MEAF6 | ENST00000373075.2 | MYST/Esa1-associated factor 6 |
| GRAMD3 | ENST00000285689.3 | GRAM domain containing 3 |
| PCBD2 | ENST00000512783.1 | pterin-4 alpha-carbinolamine dehydratase/dimerization cofactor of hepatocyte nuclear factor 1 alpha (TCF1) 2 |
| BMP8B | ENST00000372827.3 | bone morphogenetic protein 8b |
| METTL9 | ENST00000358154.3 | methyltransferase like 9 |
| LGSN | ENST00000370658.5 | lengsin, lens protein with glutamine synthetase domain |
| TRIQK | ENST00000521988.1 | triple QxxK/R motif containing |
| BCAS1 | ENST00000371440.3 | breast carcinoma amplified sequence 1 |
| ZNF598 | ENST00000563630.1 | zinc finger protein 598 |
| PIK3CG | ENST00000359195.3 | phosphatidylinositol-4,5-bisphosphate 3-kinase, catalytic subunit gamma |
| SEPHS2 | ENST00000542752.1 | selenophosphate synthetase 2 |
| IQCJ | ENST00000482126.1 | IQ motif containing J |
| KCNMB1 | ENST00000274629.4 | potassium large conductance calcium-activated channel, subfamily M, beta member 1 |
| SRSF3 | ENST00000373715.6 | serine/arginine-rich splicing factor 3 |
| PDGFB | ENST00000331163.6 | platelet-derived growth factor beta polypeptide |
| PEX2 | ENST00000357039.4 | peroxisomal biogenesis factor 2 |
| GAS1 | ENST00000298743.7 | growth arrest-specific 1 |
| SERPINB4 | ENST00000341074.5 | serpin peptidase inhibitor, clade B (ovalbumin), member 4 |
| PRSS35 | ENST00000369700.3 | protease, serine, 35 |
| GOSR1 | ENST00000225724.5 | golgi SNAP receptor complex member 1 |
| SLC39A9 | ENST00000031146.4 | solute carrier family 39, member 9 |
| ACLY | ENST00000352035.2 | ATP citrate lyase |
| MORN4 | ENST00000478953.1 | MORN repeat containing 4 |
| GUCY1A3 | ENST00000296518.7 | guanylate cyclase 1, soluble, alpha 3 |
| KRT34 | ENST00000394001.1 | keratin 34 |
| FGF2 | ENST00000264498.3 | fibroblast growth factor 2 (basic) |
| MIS12 | ENST00000381165.3 | MIS12 kinetochore complex component |
| OCEL1 | ENST00000601529.1 | occludin/ELL domain containing 1 |
| PROK1 | ENST00000271331.3 | prokineticin 1 |
| SLC9A4 | ENST00000295269.4 | solute carrier family 9, subfamily A (NHE4, cation proton antiporter 4), member 4 |
| UBE2Q1 | ENST00000292211.4 | ubiquitin-conjugating enzyme E2Q family member 1 |
| CD160 | ENST00000369290.1 | CD160 molecule |
| GTF3C2 | ENST00000359541.2 | general transcription factor IIIC, polypeptide 2, beta 110kDa |
| PSG1 | ENST00000244296.2 | pregnancy specific beta-1-glycoprotein 1 |
| RP11-204N11.1 | ENST00000555187.1 | Uncharacterized protein |
| HTRA1 | ENST00000368984.3 | HtrA serine peptidase 1 |
| DOK6 | ENST00000382713.5 | docking protein 6 |
| HLA-G | ENST00000376828.2 | major histocompatibility complex, class I, G |
| PSTPIP2 | ENST00000409746.5 | proline-serine-threonine phosphatase interacting protein 2 |
| ATMIN | ENST00000299575.4 | ATM interactor |
| ZNF23 | ENST00000497160.1 | zinc finger protein 23 |
| RNF114 | ENST00000244061.2 | ring finger protein 114 |
| SCML2 | ENST00000398048.3 | sex comb on midleg-like 2 (Drosophila) |
| GSTA2 | ENST00000493422.1 | glutathione S-transferase alpha 2 |
| BPGM | ENST00000344924.3 | 2,3-bisphosphoglycerate mutase |
| FAM118B | ENST00000533050.1 | family with sequence similarity 118, member B |
| PKD2L2 | ENST00000508638.1 | polycystic kidney disease 2-like 2 |
| GPR89C | ENST00000447947.2 | G protein-coupled receptor 89C |
| ST6GAL1 | ENST00000169298.3 | ST6 beta-galactosamide alpha-2,6-sialyltranferase 1 |
| HMGCR | ENST00000287936.4 | 3-hydroxy-3-methylglutaryl-CoA reductase |
| NME6 | ENST00000450160.1 | NME/NM23 nucleoside diphosphate kinase 6 |
| RHOQ | ENST00000238738.4 | ras homolog family member Q |
| PAK1 | ENST00000278568.4 | p21 protein (Cdc42/Rac)-activated kinase 1 |
| GBP4 | ENST00000355754.6 | guanylate binding protein 4 |
| VANGL1 | ENST00000355485.2 | VANGL planar cell polarity protein 1 |
| TRAF6 | ENST00000526995.1 | TNF receptor-associated factor 6, E3 ubiquitin protein ligase |
| MECOM | ENST00000460814.1 | MDS1 and EVI1 complex locus |
| AC023632.1 | ENST00000391679.1 | HCG2009141; PRO2397; Uncharacterized protein |
| CTSS | ENST00000368985.3 | cathepsin S |
| FCAMR | ENST00000400962.3 | Fc receptor, IgA, IgM, high affinity |
| NIPAL4 | ENST00000311946.7 | NIPA-like domain containing 4 |
| MFAP3L | ENST00000393704.3 | microfibrillar-associated protein 3-like |
| PIGG | ENST00000296306.7 | phosphatidylinositol glycan anchor biosynthesis, class G |
| AP2B1 | ENST00000262325.7 | adaptor-related protein complex 2, beta 1 subunit |
| BCR | ENST00000305877.8 | breakpoint cluster region |
| TINAGL1 | ENST00000457433.2 | tubulointerstitial nephritis antigen-like 1 |
| KCNC2 | ENST00000393288.2 | potassium voltage-gated channel, Shaw-related subfamily, member 2 |
| PHLDA1 | ENST00000602540.1 | pleckstrin homology-like domain, family A, member 1 |
| C6orf10 | ENST00000447241.2 | chromosome 6 open reading frame 10 |
| FCGR3B | ENST00000367964.2 | Fc fragment of IgG, low affinity IIIb, receptor (CD16b) |
| KLK7 | ENST00000391807.1 | kallikrein-related peptidase 7 |
| B4GALT5 | ENST00000371711.4 | UDP-Gal:betaGlcNAc beta 1,4- galactosyltransferase, polypeptide 5 |
| CBLN1 | ENST00000219197.6 | cerebellin 1 precursor |
| ZBTB37 | ENST00000367701.5 | zinc finger and BTB domain containing 37 |
| CYB5D1 | ENST00000571846.1 | cytochrome b5 domain containing 1 |
| SOX1 | ENST00000330949.1 | SRY (sex determining region Y)-box 1 |
| AC010441.1 | ENST00000600109.1 |  |
| ST6GALNAC1 | ENST00000156626.7 | ST6 (alpha-N-acetyl-neuraminyl-2,3-beta-galactosyl-1,3)-N-acetylgalactosaminide alpha-2,6-sialyltransferase 1 |
| RORC | ENST00000356728.6 | RAR-related orphan receptor C |
| METTL2B | ENST00000480046.1 | methyltransferase like 2B |
| FAAH | ENST00000243167.8 | fatty acid amide hydrolase |
| FYCO1 | ENST00000296137.2 | FYVE and coiled-coil domain containing 1 |
| CEP68 | ENST00000377990.2 | centrosomal protein 68kDa |
| BRAT1 | ENST00000340611.4 | BRCA1-associated ATM activator 1 |
| NEUROD6 | ENST00000297142.3 | neuronal differentiation 6 |
| PIN4 | ENST00000218432.5 | protein (peptidylprolyl cis/trans isomerase) NIMA-interacting, 4 (parvulin) |
| FRMD5 | ENST00000484674.1 | FERM domain containing 5 |
| SERPINB3 | ENST00000283752.5 | serpin peptidase inhibitor, clade B (ovalbumin), member 3 |
| DGUOK | ENST00000264093.4 | deoxyguanosine kinase |
| SYNPR | ENST00000479198.1 | synaptoporin |
| DDX49 | ENST00000438170.2 | DEAD (Asp-Glu-Ala-Asp) box polypeptide 49 |
| SAMD7 | ENST00000428432.2 | sterile alpha motif domain containing 7 |
| UNC5C | ENST00000453304.1 | unc-5 homolog C (C. elegans) |
| INTS2 | ENST00000444766.3 | integrator complex subunit 2 |
| RASSF2 | ENST00000379400.3 | Ras association (RalGDS/AF-6) domain family member 2 |
| SLC12A2 | ENST00000262461.2 | solute carrier family 12 (sodium/potassium/chloride transporter), member 2 |
| JMJD1C | ENST00000399251.1 | jumonji domain containing 1C |
| DMRT1 | ENST00000382276.3 | doublesex and mab-3 related transcription factor 1 |
| TAF1B | ENST00000263663.5 | TATA box binding protein (TBP)-associated factor, RNA polymerase I, B, 63kDa |
| RBL2 | ENST00000262133.6 | retinoblastoma-like 2 (p130) |
| GCLM | ENST00000370238.3 | glutamate-cysteine ligase, modifier subunit |
| AHCYL2 | ENST00000325006.3 | adenosylhomocysteinase-like 2 |
| TRAPPC11 | ENST00000357207.4 | trafficking protein particle complex 11 |
| AKR1B1 | ENST00000285930.4 | aldo-keto reductase family 1, member B1 (aldose reductase) |
| EGF | ENST00000265171.5 | epidermal growth factor |
| RAB40A | ENST00000372633.1 | RAB40A, member RAS oncogene family |
| YWHAQ | ENST00000381844.4 | tyrosine 3-monooxygenase/tryptophan 5-monooxygenase activation protein, theta polypeptide |
| FSHB | ENST00000417547.1 | follicle stimulating hormone, beta polypeptide |
| CLNK | ENST00000226951.6 | cytokine-dependent hematopoietic cell linker |
| FANCI | ENST00000300027.8 | Fanconi anemia, complementation group I |
| PBRM1 | ENST00000356770.4 | polybromo 1 |
| C2orf74 | ENST00000426997.1 | chromosome 2 open reading frame 74 |
| ERLEC1 | ENST00000185150.4 | endoplasmic reticulum lectin 1 |
| ZHX1 | ENST00000395571.3 | zinc fingers and homeoboxes 1 |
| OGN | ENST00000262551.4 | osteoglycin |
| SMIM21 | ENST00000579022.1 | small integral membrane protein 21 |
| NRN1 | ENST00000244766.2 | neuritin 1 |
| DROSHA | ENST00000511367.2 | drosha, ribonuclease type III |
| GINS4 | ENST00000523277.2 | GINS complex subunit 4 (Sld5 homolog) |
| MAGEB10 | ENST00000356790.2 | melanoma antigen family B, 10 |
| MTMR9 | ENST00000221086.3 | myotubularin related protein 9 |
| SYPL2 | ENST00000369872.3 | synaptophysin-like 2 |
| FAM221B | ENST00000423537.2 | family with sequence similarity 221, member B |
| SLC30A8 | ENST00000427715.2 | solute carrier family 30 (zinc transporter), member 8 |
| UBTF | ENST00000343638.5 | upstream binding transcription factor, RNA polymerase I |
| TMEM138 | ENST00000278826.6 | transmembrane protein 138 |
| DPPA3 | ENST00000345088.2 | developmental pluripotency associated 3 |
| ARHGAP24 | ENST00000395184.1 | Rho GTPase activating protein 24 |
| ARHGAP12 | ENST00000311380.4 | Rho GTPase activating protein 12 |
| ST8SIA5 | ENST00000315087.7 | ST8 alpha-N-acetyl-neuraminide alpha-2,8-sialyltransferase 5 |
| PDK4 | ENST00000005178.5 | pyruvate dehydrogenase kinase, isozyme 4 |
| TMEM200A | ENST00000296978.3 | transmembrane protein 200A |
| MAGEB4 | ENST00000378982.2 | melanoma antigen family B, 4 |
| ZNF664 | ENST00000538932.2 | zinc finger protein 664 |
| AC104472.1 | ENST00000399242.2 | CDNA FLJ26134 fis, clone TMS03713; Uncharacterized protein |
| TMPRSS11F | ENST00000356291.2 | transmembrane protease, serine 11F |
| ERLIN2 | ENST00000276461.5 | ER lipid raft associated 2 |
| MOCOS | ENST00000261326.5 | molybdenum cofactor sulfurase |
| TWIST1 | ENST00000242261.5 | twist basic helix-loop-helix transcription factor 1 |
| OR12D3 | ENST00000396806.3 | olfactory receptor, family 12, subfamily D, member 3 |
| TMC5 | ENST00000381414.4 | transmembrane channel-like 5 |
| TBC1D31 | ENST00000378080.2 | TBC1 domain family, member 31 |
| SMIM5 | ENST00000375215.3 | small integral membrane protein 5 |
| B4GALT6 | ENST00000306851.5 | UDP-Gal:betaGlcNAc beta 1,4- galactosyltransferase, polypeptide 6 |
| RBM7 | ENST00000541475.1 | RNA binding motif protein 7 |
| ZNRF1 | ENST00000335325.4 | zinc and ring finger 1, E3 ubiquitin protein ligase |
| FAM213A | ENST00000372187.5 | family with sequence similarity 213, member A |
| TEX13B | ENST00000302917.1 | testis expressed 13B |
| ATP10B | ENST00000327245.5 | ATPase, class V, type 10B |
| DGCR6 | ENST00000608842.1 | DiGeorge syndrome critical region gene 6 |
| DPP4 | ENST00000360534.3 | dipeptidyl-peptidase 4 |
| CCR8 | ENST00000414803.1 | chemokine (C-C motif) receptor 8 |
| ACRV1 | ENST00000533904.1 | acrosomal vesicle protein 1 |
| UBP1 | ENST00000283629.3 | upstream binding protein 1 (LBP-1a) |
| ANKLE1 | ENST00000394458.3 | ankyrin repeat and LEM domain containing 1 |
| VPS4B | ENST00000238497.5 | vacuolar protein sorting 4 homolog B (S. cerevisiae) |
| AC012360.2 | ENST00000595531.1 | LOC644617 protein; Uncharacterized protein |
| COX15 | ENST00000016171.5 | cytochrome c oxidase assembly homolog 15 (yeast) |
| HSDL1 | ENST00000219439.4 | hydroxysteroid dehydrogenase like 1 |
| OIP5 | ENST00000220514.3 | Opa interacting protein 5 |
| ATG3 | ENST00000402314.2 | autophagy related 3 |
| CNDP2 | ENST00000579847.1 | CNDP dipeptidase 2 (metallopeptidase M20 family) |
| DAW1 | ENST00000373666.2 | dynein assembly factor with WDR repeat domains 1 |
| INPP5B | ENST00000373027.1 | inositol polyphosphate-5-phosphatase, 75kDa |
| FEZF1 | ENST00000442488.2 | FEZ family zinc finger 1 |
| PHACTR3 | ENST00000359926.3 | phosphatase and actin regulator 3 |
| SLC35F5 | ENST00000245680.2 | solute carrier family 35, member F5 |
| NKTR | ENST00000232978.8 | natural killer-tumor recognition sequence |
| DNER | ENST00000341772.4 | delta/notch-like EGF repeat containing |
| MRPL30 | ENST00000338148.3 | mitochondrial ribosomal protein L30 |
| C2orf15 | ENST00000512183.2 | chromosome 2 open reading frame 15 |
| DCUN1D1 | ENST00000292782.4 | DCN1, defective in cullin neddylation 1, domain containing 1 |
| SNTN | ENST00000343837.3 | sentan, cilia apical structure protein |
| NUDT18 | ENST00000521807.2 | nudix (nucleoside diphosphate linked moiety X)-type motif 18 |
| CALD1 | ENST00000361388.2 | caldesmon 1 |
| TRNT1 | ENST00000251607.6 | tRNA nucleotidyl transferase, CCA-adding, 1 |
| C1orf141 | ENST00000371007.2 | chromosome 1 open reading frame 141 |
| ANGPT2 | ENST00000325203.5 | angiopoietin 2 |
| EVI5 | ENST00000370331.1 | ecotropic viral integration site 5 |
| UFM1 | ENST00000239878.4 | ubiquitin-fold modifier 1 |
| GATC | ENST00000551765.1 | glutamyl-tRNA(Gln) amidotransferase, subunit C |
| LRRC10 | ENST00000361484.3 | leucine rich repeat containing 10 |
| CHRNB2 | ENST00000368476.3 | cholinergic receptor, nicotinic, beta 2 (neuronal) |
| ARX | ENST00000379044.4 | aristaless related homeobox |
| ZNF154 | ENST00000512439.2 | zinc finger protein 154 |
| VAMP4 | ENST00000236192.7 | vesicle-associated membrane protein 4 |
| ASAP1 | ENST00000357668.1 | ArfGAP with SH3 domain, ankyrin repeat and PH domain 1 |
| BAAT | ENST00000259407.2 | bile acid CoA: amino acid N-acyltransferase (glycine N-choloyltransferase) |
| PLCB1 | ENST00000378641.3 | phospholipase C, beta 1 (phosphoinositide-specific) |
| ZNF286A | ENST00000395894.2 | Zinc finger protein 286A |
| TMEM86A | ENST00000280734.2 | transmembrane protein 86A |
| TMEM213 | ENST00000442682.2 | transmembrane protein 213 |
| TMEM239 | ENST00000361033.1 | transmembrane protein 239 |
| RBL1 | ENST00000373664.3 | retinoblastoma-like 1 (p107) |
| NIN | ENST00000389868.3 | ninein (GSK3B interacting protein) |
| MUC4 | ENST00000349607.4 | mucin 4, cell surface associated |
| CD38 | ENST00000226279.3 | CD38 molecule |
| ALDH9A1 | ENST00000354775.4 | aldehyde dehydrogenase 9 family, member A1 |
| NDUFA5 | ENST00000471770.1 | NADH dehydrogenase (ubiquinone) 1 alpha subcomplex, 5 |
| CD1D | ENST00000368171.3 | CD1d molecule |
| SLC43A3 | ENST00000395123.2 | solute carrier family 43, member 3 |
| ZFR | ENST00000265069.8 | zinc finger RNA binding protein |
| EGLN1 | ENST00000366641.3 | egl-9 family hypoxia-inducible factor 1 |
| DLX6 | ENST00000518156.2 | distal-less homeobox 6 |
| LRRC8B | ENST00000330947.2 | leucine rich repeat containing 8 family, member B |
| LRP8 | ENST00000306052.6 | low density lipoprotein receptor-related protein 8, apolipoprotein e receptor |
| C1orf21 | ENST00000235307.6 | chromosome 1 open reading frame 21 |
| VASH2 | ENST00000366968.4 | vasohibin 2 |
| KLHL24 | ENST00000454652.2 | kelch-like family member 24 |
| RRS1 | ENST00000320270.2 | RRS1 ribosome biogenesis regulator homolog (S. cerevisiae) |
| TLR7 | ENST00000380659.3 | toll-like receptor 7 |
| ULBP3 | ENST00000367339.2 | UL16 binding protein 3 |
| GULP1 | ENST00000409843.1 | GULP, engulfment adaptor PTB domain containing 1 |
| IPCEF1 | ENST00000265198.4 | interaction protein for cytohesin exchange factors 1 |
| CHD3 | ENST00000380358.4 | chromodomain helicase DNA binding protein 3 |
| FZD6 | ENST00000358755.4 | frizzled family receptor 6 |
| AP000889.3 | ENST00000600612.1 | HCG2032453; Uncharacterized protein; cDNA FLJ25337 fis, clone TST00714 |
| CDCP2 | ENST00000371330.1 | CUB domain containing protein 2 |
| CHMP3 | ENST00000263856.4 | charged multivesicular body protein 3 |
| CASP1 | ENST00000533400.1 | caspase 1, apoptosis-related cysteine peptidase |
| CTAGE1 | ENST00000391403.2 | cutaneous T-cell lymphoma-associated antigen 1 |
| RNF103-CHMP3 | ENST00000604011.1 | RNF103-CHMP3 readthrough |
| METTL2A | ENST00000311506.5 | methyltransferase like 2A |
| PIP4K2C | ENST00000354947.5 | phosphatidylinositol-5-phosphate 4-kinase, type II, gamma |
| SLC25A17 | ENST00000435456.2 | solute carrier family 25 (mitochondrial carrier; peroxisomal membrane protein, 34kDa), member 17 |
| STARD4 | ENST00000512160.1 | StAR-related lipid transfer (START) domain containing 4 |
| ARL9 | ENST00000360096.2 | ADP-ribosylation factor-like 9 |
| SRPR | ENST00000332118.6 | signal recognition particle receptor (docking protein) |
| SCPEP1 | ENST00000262288.3 | serine carboxypeptidase 1 |
| MNX1 | ENST00000252971.6 | motor neuron and pancreas homeobox 1 |
| ETNK1 | ENST00000266517.4 | ethanolamine kinase 1 |
| CLUAP1 | ENST00000576634.1 | clusterin associated protein 1 |
| MAN1A1 | ENST00000368468.3 | mannosidase, alpha, class 1A, member 1 |
| ATP8A2 | ENST00000381655.2 | ATPase, aminophospholipid transporter, class I, type 8A, member 2 |
| MFGE8 | ENST00000539437.1 | milk fat globule-EGF factor 8 protein |
| ARL10 | ENST00000310389.5 | ADP-ribosylation factor-like 10 |
| TACR1 | ENST00000305249.5 | tachykinin receptor 1 |
| FAM111B | ENST00000411426.1 | family with sequence similarity 111, member B |
| RNF145 | ENST00000274542.2 | ring finger protein 145 |
| LCMT2 | ENST00000567039.1 | leucine carboxyl methyltransferase 2 |
| C1orf173 | ENST00000326665.5 | chromosome 1 open reading frame 173 |
| SCML1 | ENST00000380045.3 | sex comb on midleg-like 1 (Drosophila) |
| EPB41L1 | ENST00000441639.1 | erythrocyte membrane protein band 4.1-like 1 |
| DSCR3 | ENST00000309117.6 | Down syndrome critical region gene 3 |
| ITM2B | ENST00000378565.5 | integral membrane protein 2B |
| DCAKD | ENST00000452796.2 | dephospho-CoA kinase domain containing |
| TLCD2 | ENST00000330676.6 | TLC domain containing 2 |
| SH3RF2 | ENST00000511217.1 | SH3 domain containing ring finger 2 |
| KRBOX4 | ENST00000360017.5 | KRAB box domain containing 4 |
| CLOCK | ENST00000309964.4 | clock circadian regulator |
| NAIP | ENST00000517649.1 | NLR family, apoptosis inhibitory protein |
| WNT2B | ENST00000369686.5 | wingless-type MMTV integration site family, member 2B |
| BANP | ENST00000393208.2 | BTG3 associated nuclear protein |
| SLC6A20 | ENST00000353278.4 | solute carrier family 6 (proline IMINO transporter), member 20 |
| THBD | ENST00000377103.2 | thrombomodulin |
| HERC6 | ENST00000264346.7 | HECT and RLD domain containing E3 ubiquitin protein ligase family member 6 |
| SEC14L3 | ENST00000215812.4 | SEC14-like 3 (S. cerevisiae) |
| RNF125 | ENST00000217740.3 | ring finger protein 125, E3 ubiquitin protein ligase |
| LIN28B | ENST00000345080.4 | lin-28 homolog B (C. elegans) |
| SNX22 | ENST00000325881.4 | sorting nexin 22 |
| IFNLR1 | ENST00000327575.2 | interferon, lambda receptor 1 |
| PCDHB7 | ENST00000231137.3 | protocadherin beta 7 |
| HINFP | ENST00000350777.2 | histone H4 transcription factor |
| ZNF441 | ENST00000357901.4 | zinc finger protein 441 |
| MRS2 | ENST00000274747.7 | MRS2 magnesium transporter |
| ZNF84 | ENST00000392319.2 | zinc finger protein 84 |
| SLC25A44 | ENST00000359511.4 | solute carrier family 25, member 44 |
| AL021546.6 | ENST00000551806.1 | Glutamyl-tRNA(Gln) amidotransferase subunit C, mitochondrial |
| ZNF487 | ENST00000437590.2 | zinc finger protein 487 |
| GPR110 | ENST00000371253.2 | G protein-coupled receptor 110 |
| ANKRD29 | ENST00000592179.1 | ankyrin repeat domain 29 |
| ICAM1 | ENST00000264832.3 | intercellular adhesion molecule 1 |
| SHC4 | ENST00000332408.4 | SHC (Src homology 2 domain containing) family, member 4 |
| PRLR | ENST00000342362.5 | prolactin receptor |
| ANO1 | ENST00000355303.5 | anoctamin 1, calcium activated chloride channel |
| C1orf131 | ENST00000318906.2 | chromosome 1 open reading frame 131 |
| SNX20 | ENST00000330943.4 | sorting nexin 20 |
| ALDH2 | ENST00000261733.2 | aldehyde dehydrogenase 2 family (mitochondrial) |
| TSLP | ENST00000379706.4 | thymic stromal lymphopoietin |
| WRAP73 | ENST00000378322.3 | WD repeat containing, antisense to TP73 |
| TGM4 | ENST00000296125.4 | transglutaminase 4 |
| SLC28A3 | ENST00000376238.4 | solute carrier family 28 (concentrative nucleoside transporter), member 3 |
| STRIP2 | ENST00000249344.2 | striatin interacting protein 2 |
| TAF1A | ENST00000543857.1 | TATA box binding protein (TBP)-associated factor, RNA polymerase I, A, 48kDa |
| WARS2 | ENST00000369426.5 | tryptophanyl tRNA synthetase 2, mitochondrial |
| CNTF | ENST00000361987.4 | ciliary neurotrophic factor |
| CYP39A1 | ENST00000275016.2 | cytochrome P450, family 39, subfamily A, polypeptide 1 |
| DAZ4 | ENST00000382290.3 | deleted in azoospermia 4 |
| PRKCH | ENST00000332981.5 | protein kinase C, eta |
| LOH12CR2 | ENST00000381800.2 | loss of heterozygosity, 12, chromosomal region 2 (non-protein coding) |
| ODC1 | ENST00000234111.4 | ornithine decarboxylase 1 |
| ZNF841 | ENST00000594295.1 | zinc finger protein 841 |
| SLITRK6 | ENST00000400286.2 | SLIT and NTRK-like family, member 6 |
| PCDHB12 | ENST00000239450.2 | protocadherin beta 12 |
| QKI | ENST00000392127.2 | QKI, KH domain containing, RNA binding |
| DKFZP434H0512 | ENST00000600140.1 | Protein LOC100506667; Putative uncharacterized protein DKFZp434H0512 |
| ZNF280A | ENST00000302097.3 | zinc finger protein 280A |
| PARP16 | ENST00000261888.6 | poly (ADP-ribose) polymerase family, member 16 |
| PCDH11Y | ENST00000215473.6 | protocadherin 11 Y-linked |
| CYP20A1 | ENST00000356079.4 | cytochrome P450, family 20, subfamily A, polypeptide 1 |
| TNIK | ENST00000436636.2 | TRAF2 and NCK interacting kinase |
| PRDM14 | ENST00000276594.2 | PR domain containing 14 |
| RP6-24A23.6 | ENST00000563887.1 | Uncharacterized protein |
| ERCC6L2 | ENST00000407474.3 | excision repair cross-complementing rodent repair deficiency, complementation group 6-like 2 |
| FOXJ2 | ENST00000162391.3 | forkhead box J2 |
| TTPA | ENST00000260116.4 | tocopherol (alpha) transfer protein |
| NLGN1 | ENST00000457714.1 | neuroligin 1 |
| KIAA0947 | ENST00000296564.7 | KIAA0947 |
| PLOD2 | ENST00000461497.1 | procollagen-lysine, 2-oxoglutarate 5-dioxygenase 2 |
| AAK1 | ENST00000409085.4 | AP2 associated kinase 1 |
| MTHFD1 | ENST00000216605.8 | methylenetetrahydrofolate dehydrogenase (NADP+ dependent) 1, methenyltetrahydrofolate cyclohydrolase, formyltetrahydrofolate synthetase |
| RCAN1 | ENST00000482533.1 | regulator of calcineurin 1 |
| RABGEF1 | ENST00000284957.5 | RAB guanine nucleotide exchange factor (GEF) 1 |
| HECTD2 | ENST00000371667.1 | HECT domain containing E3 ubiquitin protein ligase 2 |
| POU5F1B | ENST00000465342.2 | POU class 5 homeobox 1B |
| PROK2 | ENST00000353065.3 | prokineticin 2 |
| WASF3 | ENST00000335327.5 | WAS protein family, member 3 |
| CYP4B1 | ENST00000271153.4 | cytochrome P450, family 4, subfamily B, polypeptide 1 |
| KRT38 | ENST00000246646.3 | keratin 38 |
| RSPH10B2 | ENST00000359718.3 | radial spoke head 10 homolog B2 (Chlamydomonas) |
| RSPH4A | ENST00000368581.4 | radial spoke head 4 homolog A (Chlamydomonas) |
| CEPT1 | ENST00000545121.1 | choline/ethanolamine phosphotransferase 1 |
| RAB33B | ENST00000305626.5 | RAB33B, member RAS oncogene family |
| ICA1L | ENST00000392237.2 | islet cell autoantigen 1,69kDa-like |
| SLC30A4 | ENST00000261867.4 | solute carrier family 30 (zinc transporter), member 4 |
| MSRB3 | ENST00000308259.5 | methionine sulfoxide reductase B3 |
| ACOT8 | ENST00000217455.4 | acyl-CoA thioesterase 8 |
| LIFR | ENST00000263409.4 | leukemia inhibitory factor receptor alpha |
| CLDN23 | ENST00000519106.1 | claudin 23 |
| FLG | ENST00000368799.1 | filaggrin |
| CNGB3 | ENST00000320005.5 | cyclic nucleotide gated channel beta 3 |
| RPRD1B | ENST00000373433.4 | regulation of nuclear pre-mRNA domain containing 1B |
| ZNF572 | ENST00000319286.5 | zinc finger protein 572 |
| PSG9 | ENST00000244293.7 | pregnancy specific beta-1-glycoprotein 9 |
| EMR2 | ENST00000315576.3 | egf-like module containing, mucin-like, hormone receptor-like 2 |
| HMGB3 | ENST00000325307.7 | high mobility group box 3 |
| TARDBP | ENST00000240185.3 | TAR DNA binding protein |
| ZNF486 | ENST00000335117.8 | zinc finger protein 486 |
| MCM8 | ENST00000378896.3 | minichromosome maintenance complex component 8 |
| TGFBR2 | ENST00000359013.4 | transforming growth factor, beta receptor II (70/80kDa) |
| HBS1L | ENST00000367837.5 | HBS1-like (S. cerevisiae) |
| CANT1 | ENST00000392446.5 | calcium activated nucleotidase 1 |
| CPT1A | ENST00000265641.5 | carnitine palmitoyltransferase 1A (liver) |
| RSAD2 | ENST00000382040.3 | radical S-adenosyl methionine domain containing 2 |
| ZNF587B | ENST00000442832.4 | zinc finger protein 587B |
| KCNAB1 | ENST00000302490.8 | potassium voltage-gated channel, shaker-related subfamily, beta member 1 |
| GPR50 | ENST00000218316.3 | G protein-coupled receptor 50 |
| TRIM66 | ENST00000299550.6 | tripartite motif containing 66 |
| SEMA6A | ENST00000343348.6 | sema domain, transmembrane domain (TM), and cytoplasmic domain, (semaphorin) 6A |
| MAPK1 | ENST00000215832.6 | mitogen-activated protein kinase 1 |
| YIPF4 | ENST00000238831.4 | Yip1 domain family, member 4 |
| FAR1 | ENST00000354817.3 | fatty acyl CoA reductase 1 |
| LEPROTL1 | ENST00000321250.8 | leptin receptor overlapping transcript-like 1 |
| GATA6 | ENST00000269216.3 | GATA binding protein 6 |
| METAP1 | ENST00000296411.6 | methionyl aminopeptidase 1 |
| SPI1 | ENST00000533030.1 | spleen focus forming virus (SFFV) proviral integration oncogene |
| AL009178.1 | ENST00000597278.1 | Uncharacterized protein; cDNA FLJ43200 fis, clone FEBRA2007793 |
| SLC25A21 | ENST00000331299.5 | solute carrier family 25 (mitochondrial oxoadipate carrier), member 21 |
| RNF11 | ENST00000242719.3 | ring finger protein 11 |
| ICOS | ENST00000435193.1 | inducible T-cell co-stimulator |
| RBFA | ENST00000262197.7 | ribosome binding factor A (putative) |
| PSME3 | ENST00000541124.1 | proteasome (prosome, macropain) activator subunit 3 (PA28 gamma; Ki) |
| UBE2V1 | ENST00000371657.5 | ubiquitin-conjugating enzyme E2 variant 1 |
| MS4A8 | ENST00000300226.2 | membrane-spanning 4-domains, subfamily A, member 8 |
| ZNF37A | ENST00000361085.5 | zinc finger protein 37A |
| POR | ENST00000394893.1 | P450 (cytochrome) oxidoreductase |
| TMEM200B | ENST00000521452.1 | transmembrane protein 200B |
| C5AR1 | ENST00000355085.3 | complement component 5a receptor 1 |
| KCNS1 | ENST00000306117.1 | potassium voltage-gated channel, delayed-rectifier, subfamily S, member 1 |
| RBPMS | ENST00000320203.4 | RNA binding protein with multiple splicing |
| IMPG1 | ENST00000369963.3 | interphotoreceptor matrix proteoglycan 1 |
| ZRANB2 | ENST00000254821.6 | zinc finger, RAN-binding domain containing 2 |
| FAM129A | ENST00000367511.3 | family with sequence similarity 129, member A |
| RALGPS1 | ENST00000259351.5 | Ral GEF with PH domain and SH3 binding motif 1 |
| RHOU | ENST00000366691.3 | ras homolog family member U |
| AKAP6 | ENST00000557272.1 | A kinase (PRKA) anchor protein 6 |
| IDI1 | ENST00000381344.3 | isopentenyl-diphosphate delta isomerase 1 |
| DNAJB11 | ENST00000439351.1 | DnaJ (Hsp40) homolog, subfamily B, member 11 |
| GOLGA6A | ENST00000290438.3 | golgin A6 family, member A |
| ACSF3 | ENST00000317447.4 | acyl-CoA synthetase family member 3 |
| FRMD3 | ENST00000304195.3 | FERM domain containing 3 |
| RGSL1 | ENST00000294854.8 | regulator of G-protein signaling like 1 |
| TPK1 | ENST00000549981.1 | thiamin pyrophosphokinase 1 |
| PMP2 | ENST00000256103.2 | peripheral myelin protein 2 |
| METTL13 | ENST00000362019.3 | methyltransferase like 13 |
| PYGO1 | ENST00000302000.6 | pygopus homolog 1 (Drosophila) |
| EIF4EBP2 | ENST00000373218.4 | eukaryotic translation initiation factor 4E binding protein 2 |
| ZNF292 | ENST00000369577.3 | zinc finger protein 292 |
| WDR43 | ENST00000407426.3 | WD repeat domain 43 |
| C10orf71 | ENST00000323868.4 | chromosome 10 open reading frame 71 |
| ARMC1 | ENST00000276569.3 | armadillo repeat containing 1 |
| C10orf137 | ENST00000337623.3 | chromosome 10 open reading frame 137 |
| SLC22A2 | ENST00000366953.3 | solute carrier family 22 (organic cation transporter), member 2 |
| POMGNT1 | ENST00000396420.3 | protein O-linked mannose N-acetylglucosaminyltransferase 1 (beta 1,2-) |
| ETV3 | ENST00000368192.4 | ets variant 3 |
| AKAP11 | ENST00000025301.2 | A kinase (PRKA) anchor protein 11 |
| SLC9C1 | ENST00000305815.5 | solute carrier family 9, subfamily C (Na+-transporting carboxylic acid decarboxylase), member 1 |
| SLC17A7 | ENST00000221485.3 | solute carrier family 17 (vesicular glutamate transporter), member 7 |
| LARP1B | ENST00000441387.1 | La ribonucleoprotein domain family, member 1B |
| FRMD6 | ENST00000395718.2 | FERM domain containing 6 |
| ATPAF1 | ENST00000576409.1 | ATP synthase mitochondrial F1 complex assembly factor 1 |
| MBNL3 | ENST00000370839.3 | muscleblind-like splicing regulator 3 |
| GPR56 | ENST00000388812.4 | G protein-coupled receptor 56 |
| RAB27B | ENST00000262094.5 | RAB27B, member RAS oncogene family |
| ELOVL6 | ENST00000394607.3 | ELOVL fatty acid elongase 6 |
| TULP4 | ENST00000367094.2 | tubby like protein 4 |
| TIGIT | ENST00000486257.1 | T cell immunoreceptor with Ig and ITIM domains |
| SMAD3 | ENST00000327367.4 | SMAD family member 3 |
| EDEM3 | ENST00000318130.8 | ER degradation enhancer, mannosidase alpha-like 3 |
| RAB35 | ENST00000534951.1 | RAB35, member RAS oncogene family |
| KCNG1 | ENST00000396017.3 | potassium voltage-gated channel, subfamily G, member 1 |
| C1QL3 | ENST00000298943.3 | complement component 1, q subcomponent-like 3 |
| FSTL1 | ENST00000295633.3 | follistatin-like 1 |
| TNFRSF10A | ENST00000221132.3 | tumor necrosis factor receptor superfamily, member 10a |
| DMRT2 | ENST00000259622.6 | doublesex and mab-3 related transcription factor 2 |
| FRS2 | ENST00000550389.1 | fibroblast growth factor receptor substrate 2 |
| CCDC149 | ENST00000428116.2 | coiled-coil domain containing 149 |
| FAM115A | ENST00000355951.2 | family with sequence similarity 115, member A |
| NHEJ1 | ENST00000356853.5 | nonhomologous end-joining factor 1 |
| ARHGAP35 | ENST00000404338.3 | Rho GTPase activating protein 35 |
| PLXDC2 | ENST00000377252.4 | plexin domain containing 2 |
| MIS18A | ENST00000290130.3 | MIS18 kinetochore protein homolog A (S. pombe) |
| TAF8 | ENST00000372977.3 | TAF8 RNA polymerase II, TATA box binding protein (TBP)-associated factor, 43kDa |
| ENOX1 | ENST00000261488.6 | ecto-NOX disulfide-thiol exchanger 1 |
| CAMK2N1 | ENST00000375078.3 | calcium/calmodulin-dependent protein kinase II inhibitor 1 |
| MKX | ENST00000375790.5 | mohawk homeobox |
| TOR2A | ENST00000458505.3 | torsin family 2, member A |
| PPM1M | ENST00000296487.4 | protein phosphatase, Mg2+/Mn2+ dependent, 1M |
| OXR1 | ENST00000312046.6 | oxidation resistance 1 |
| MTHFSD | ENST00000360900.6 | methenyltetrahydrofolate synthetase domain containing |
| WBP2NL | ENST00000328823.9 | WBP2 N-terminal like |
| ZNF235 | ENST00000291182.4 | zinc finger protein 235 |
| FYTTD1 | ENST00000241502.4 | forty-two-three domain containing 1 |
| RFC3 | ENST00000380071.3 | replication factor C (activator 1) 3, 38kDa |
| FAM198A | ENST00000430121.2 | family with sequence similarity 198, member A |
| SLC25A27 | ENST00000371347.5 | solute carrier family 25, member 27 |
| PTPLB | ENST00000383657.5 | protein tyrosine phosphatase-like (proline instead of catalytic arginine), member b |
| DNAJC16 | ENST00000375847.3 | DnaJ (Hsp40) homolog, subfamily C, member 16 |
| GPR64 | ENST00000379873.2 | G protein-coupled receptor 64 |
| GPX8 | ENST00000296734.6 | glutathione peroxidase 8 (putative) |
| P2RY2 | ENST00000311131.2 | purinergic receptor P2Y, G-protein coupled, 2 |
| PDAP1 | ENST00000350498.3 | PDGFA associated protein 1 |
| PWWP2A | ENST00000456329.3 | PWWP domain containing 2A |
| FHDC1 | ENST00000260008.3 | FH2 domain containing 1 |
| ENTPD5 | ENST00000334696.6 | ectonucleoside triphosphate diphosphohydrolase 5 |
| FAM198B | ENST00000585682.1 | family with sequence similarity 198, member B |
| CRIM1 | ENST00000280527.2 | cysteine rich transmembrane BMP regulator 1 (chordin-like) |
| AQP5 | ENST00000293599.6 | aquaporin 5 |
| URI1 | ENST00000392271.1 | URI1, prefoldin-like chaperone |
| STC1 | ENST00000290271.2 | stanniocalcin 1 |
| MED28 | ENST00000237380.7 | mediator complex subunit 28 |
| ZNF451 | ENST00000370708.4 | zinc finger protein 451 |
| LCOR | ENST00000371103.3 | ligand dependent nuclear receptor corepressor |
| C15orf54 | ENST00000318578.3 | chromosome 15 open reading frame 54 |
| KNTC1 | ENST00000537348.1 | kinetochore associated 1 |
| ELF2 | ENST00000394235.2 | E74-like factor 2 (ets domain transcription factor) |
| STYXL1 | ENST00000360591.3 | serine/threonine/tyrosine interacting-like 1 |
| CCNG1 | ENST00000340828.2 | cyclin G1 |
| FRMPD4 | ENST00000380682.1 | FERM and PDZ domain containing 4 |
| AC007405.2 | ENST00000409786.1 | Uncharacterized protein |
| EXOSC10 | ENST00000544779.1 | exosome component 10 |
| DHRSX | ENST00000334651.5 | dehydrogenase/reductase (SDR family) X-linked |
| RCN1 | ENST00000054950.3 | reticulocalbin 1, EF-hand calcium binding domain |
| PRELID2 | ENST00000334744.4 | PRELI domain containing 2 |
| PNRC1 | ENST00000336032.3 | proline-rich nuclear receptor coactivator 1 |
| CC2D1B | ENST00000371586.2 | coiled-coil and C2 domain containing 1B |
| PRKAA2 | ENST00000371244.4 | protein kinase, AMP-activated, alpha 2 catalytic subunit |
| RBBP5 | ENST00000264515.6 | retinoblastoma binding protein 5 |
| RNF122 | ENST00000256257.1 | ring finger protein 122 |
| GLP1R | ENST00000373256.4 | glucagon-like peptide 1 receptor |
| ZNF436 | ENST00000314011.4 | zinc finger protein 436 |
| METTL15 | ENST00000342303.5 | methyltransferase like 15 |
| NSRP1 | ENST00000247026.5 | nuclear speckle splicing regulatory protein 1 |
| PRICKLE2 | ENST00000295902.6 | prickle homolog 2 (Drosophila) |
| POTEM | ENST00000551509.1 | POTE ankyrin domain family, member M |
| PSEN1 | ENST00000344094.3 | presenilin 1 |
| F2RL2 | ENST00000296641.4 | coagulation factor II (thrombin) receptor-like 2 |
| POTEG | ENST00000409832.3 | POTE ankyrin domain family, member G |
| NUP98 | ENST00000359171.4 | nucleoporin 98kDa |
| FAM83B | ENST00000306858.7 | family with sequence similarity 83, member B |
| ERG | ENST00000398905.1 | v-ets avian erythroblastosis virus E26 oncogene homolog |
| FAM181B | ENST00000329203.3 | family with sequence similarity 181, member B |
| UNC119B | ENST00000344651.4 | unc-119 homolog B (C. elegans) |
| CLCN6 | ENST00000312413.6 | chloride channel, voltage-sensitive 6 |
| ZNF680 | ENST00000447137.2 | zinc finger protein 680 |
| GRK5 | ENST00000392870.2 | G protein-coupled receptor kinase 5 |
| NHSL1 | ENST00000427025.2 | NHS-like 1 |
| MKRN2 | ENST00000170447.7 | makorin ring finger protein 2 |
| SVOP | ENST00000299134.5 | SV2 related protein homolog (rat) |
| SEC16B | ENST00000308284.6 | SEC16 homolog B (S. cerevisiae) |
| LCP2 | ENST00000046794.5 | lymphocyte cytosolic protein 2 (SH2 domain containing leukocyte protein of 76kDa) |
| KLHL6 | ENST00000341319.3 | kelch-like family member 6 |
| KLHL4 | ENST00000373119.4 | kelch-like family member 4 |
| FAM174B | ENST00000327355.5 | family with sequence similarity 174, member B |
| VWC2L | ENST00000427124.1 | von Willebrand factor C domain containing protein 2-like |
| FAHD1 | ENST00000427358.2 | fumarylacetoacetate hydrolase domain containing 1 |
| RAB22A | ENST00000244040.3 | RAB22A, member RAS oncogene family |
| RICTOR | ENST00000357387.3 | RPTOR independent companion of MTOR, complex 2 |
| MAPK10 | ENST00000395169.3 | mitogen-activated protein kinase 10 |
| CRTC3 | ENST00000420329.2 | CREB regulated transcription coactivator 3 |
| PRR9 | ENST00000368744.3 | proline rich 9 |
| TMPRSS11BNL | ENST00000432593.3 | TMPRSS11B N-terminal like |
| SP9 | ENST00000394967.2 | Sp9 transcription factor |
| LTB4R | ENST00000396789.4 | leukotriene B4 receptor |
| DIEXF | ENST00000491415.2 | digestive organ expansion factor homolog (zebrafish) |
| PDE4D | ENST00000340635.6 | phosphodiesterase 4D, cAMP-specific |
| DNAJC24 | ENST00000465995.1 | DnaJ (Hsp40) homolog, subfamily C, member 24 |
| TGFBR1 | ENST00000374994.4 | transforming growth factor, beta receptor 1 |
| CNTD2 | ENST00000430325.2 | cyclin N-terminal domain containing 2 |
| RASL12 | ENST00000220062.4 | RAS-like, family 12 |
| ATG4A | ENST00000372232.3 | autophagy related 4A, cysteine peptidase |
| ZNF804B | ENST00000333190.4 | zinc finger protein 804B |
| PAPD7 | ENST00000230859.6 | PAP associated domain containing 7 |
| MTFP1 | ENST00000266263.5 | mitochondrial fission process 1 |
| SNX27 | ENST00000368843.3 | sorting nexin family member 27 |
| CXorf22 | ENST00000297866.5 | chromosome X open reading frame 22 |
| RSPH10B | ENST00000539903.1 | radial spoke head 10 homolog B (Chlamydomonas) |
| ZNF599 | ENST00000587354.2 | zinc finger protein 599 |
| PRKDC | ENST00000314191.2 | protein kinase, DNA-activated, catalytic polypeptide |
| HDAC8 | ENST00000439122.2 | histone deacetylase 8 |
| MSR1 | ENST00000350896.3 | macrophage scavenger receptor 1 |
| NOTCH2NL | ENST00000369340.3 | notch 2 N-terminal like |
| PPP1R14C | ENST00000361131.4 | protein phosphatase 1, regulatory (inhibitor) subunit 14C |
| PARS2 | ENST00000371279.3 | prolyl-tRNA synthetase 2, mitochondrial (putative) |
| ABHD17B | ENST00000333421.6 | abhydrolase domain containing 17B |
| TMEM33 | ENST00000504986.1 | transmembrane protein 33 |
| CBFB | ENST00000290858.6 | core-binding factor, beta subunit |
| HCN3 | ENST00000368358.3 | hyperpolarization activated cyclic nucleotide-gated potassium channel 3 |
| PPARGC1A | ENST00000264867.2 | peroxisome proliferator-activated receptor gamma, coactivator 1 alpha |
| STX3 | ENST00000535361.1 | syntaxin 3 |
| LGALS3BP | ENST00000591778.1 | lectin, galactoside-binding, soluble, 3 binding protein |
| RIN2 | ENST00000255006.6 | Ras and Rab interactor 2 |
| ZNF654 | ENST00000309495.5 | zinc finger protein 654 |
| STK17B | ENST00000263955.4 | serine/threonine kinase 17b |
| TNFAIP8L3 | ENST00000327536.5 | tumor necrosis factor, alpha-induced protein 8-like 3 |
| FRMD7 | ENST00000370879.1 | FERM domain containing 7 |
| MTHFD2 | ENST00000394053.2 | methylenetetrahydrofolate dehydrogenase (NADP+ dependent) 2, methenyltetrahydrofolate cyclohydrolase |
| LAD1 | ENST00000391967.2 | ladinin 1 |
| WNT2 | ENST00000265441.3 | wingless-type MMTV integration site family member 2 |
| TMEM260 | ENST00000261556.6 | transmembrane protein 260 |
| CC2D2B | ENST00000371198.2 | coiled-coil and C2 domain containing 2B |
| SREK1IP1 | ENST00000513458.4 | SREK1-interacting protein 1 |
| TAP2 | ENST00000374897.2 | transporter 2, ATP-binding cassette, sub-family B (MDR/TAP) |
| AP1S3 | ENST00000396654.2 | adaptor-related protein complex 1, sigma 3 subunit |
| WDR26 | ENST00000414423.2 | WD repeat domain 26 |
| FAM53C | ENST00000513056.1 | family with sequence similarity 53, member C |
| GHITM | ENST00000372134.3 | growth hormone inducible transmembrane protein |
| TMEM242 | ENST00000400788.4 | transmembrane protein 242 |
| ZRANB3 | ENST00000401392.1 | zinc finger, RAN-binding domain containing 3 |
| CA10 | ENST00000570565.1 | carbonic anhydrase X |
| KIAA1199 | ENST00000220244.3 | KIAA1199 |
| RXFP1 | ENST00000307765.5 | relaxin/insulin-like family peptide receptor 1 |
| ZNF558 | ENST00000601372.1 | zinc finger protein 558 |
| C1orf200 | ENST00000377320.3 | chromosome 1 open reading frame 200 |
| RP11-770J1.4 | ENST00000532619.1 |  |
| BCL7A | ENST00000538010.1 | B-cell CLL/lymphoma 7A |
| MPZL2 | ENST00000278937.2 | myelin protein zero-like 2 |
| MAN1A2 | ENST00000356554.3 | mannosidase, alpha, class 1A, member 2 |
| IPO7 | ENST00000379719.3 | importin 7 |
| MYLIP | ENST00000349606.4 | myosin regulatory light chain interacting protein |
| PDZD8 | ENST00000334464.5 | PDZ domain containing 8 |
| MRPS25 | ENST00000253686.2 | mitochondrial ribosomal protein S25 |
| OLR1 | ENST00000543993.1 | oxidized low density lipoprotein (lectin-like) receptor 1 |
| FAM156B | ENST00000430150.2 | family with sequence similarity 156, member B |
| TMEM192 | ENST00000306480.6 | transmembrane protein 192 |
| KLHL7 | ENST00000339077.5 | kelch-like family member 7 |
| DDAH1 | ENST00000535924.2 | dimethylarginine dimethylaminohydrolase 1 |
| ASCL1 | ENST00000266744.3 | achaete-scute complex homolog 1 (Drosophila) |
| F10 | ENST00000375551.3 | coagulation factor X |
| AC092850.1 | ENST00000598023.1 | Protein LOC100996701 |
| FAM156A | ENST00000356333.4 | family with sequence similarity 156, member A |
| HIST1H2BJ | ENST00000541790.1 | histone cluster 1, H2bj |
| KCTD7 | ENST00000275532.3 | potassium channel tetramerization domain containing 7 |
| OGT | ENST00000373719.3 | O-linked N-acetylglucosamine (GlcNAc) transferase |
| CECR6 | ENST00000399875.1 | cat eye syndrome chromosome region, candidate 6 |
| CTD-2368P22.1 | ENST00000550135.1 | HCG1811579; Uncharacterized protein |
| ACSBG1 | ENST00000258873.4 | acyl-CoA synthetase bubblegum family member 1 |
| C9orf84 | ENST00000394779.3 | chromosome 9 open reading frame 84 |
| KCNN3 | ENST00000271915.4 | potassium intermediate/small conductance calcium-activated channel, subfamily N, member 3 |
| NELL2 | ENST00000395487.2 | NEL-like 2 (chicken) |
| PMM2 | ENST00000268261.4 | phosphomannomutase 2 |
| FBXL20 | ENST00000394294.3 | F-box and leucine-rich repeat protein 20 |
| DIP2C | ENST00000280886.6 | DIP2 disco-interacting protein 2 homolog C (Drosophila) |
| BMPR2 | ENST00000374574.2 | bone morphogenetic protein receptor, type II (serine/threonine kinase) |
| IKZF5 | ENST00000368886.5 | IKAROS family zinc finger 5 (Pegasus) |
| ANKRD6 | ENST00000369408.5 | ankyrin repeat domain 6 |
| DTNA | ENST00000283365.9 | dystrobrevin, alpha |
| LINC00923 | ENST00000503874.3 | long intergenic non-protein coding RNA 923 |
| PRDM12 | ENST00000253008.2 | PR domain containing 12 |
| FMNL2 | ENST00000475377.2 | formin-like 2 |
| PRDM9 | ENST00000296682.3 | PR domain containing 9 |
| XBP1 | ENST00000344347.5 | X-box binding protein 1 |
| CLN6 | ENST00000249806.5 | ceroid-lipofuscinosis, neuronal 6, late infantile, variant |
| CALU | ENST00000535011.2 | calumenin |
| GAS2 | ENST00000454584.2 | growth arrest-specific 2 |
| MAGI2 | ENST00000419488.1 | membrane associated guanylate kinase, WW and PDZ domain containing 2 |
| SMO | ENST00000249373.3 | smoothened, frizzled family receptor |
| ESPNL | ENST00000343063.3 | espin-like |
| IKBKE | ENST00000367120.3 | inhibitor of kappa light polypeptide gene enhancer in B-cells, kinase epsilon |
| GCH1 | ENST00000491895.2 | GTP cyclohydrolase 1 |
| ENPP5 | ENST00000371383.2 | ectonucleotide pyrophosphatase/phosphodiesterase 5 (putative) |
| MUT | ENST00000274813.3 | methylmalonyl CoA mutase |
| VAPB | ENST00000395802.3 | VAMP (vesicle-associated membrane protein)-associated protein B and C |
| APH1A | ENST00000360244.4 | APH1A gamma secretase subunit |
| ERI1 | ENST00000250263.7 | exoribonuclease 1 |
| KRBA2 | ENST00000396267.1 | KRAB-A domain containing 2 |
| KRI1 | ENST00000312962.6 | KRI1 homolog (S. cerevisiae) |
| RBMS3 | ENST00000396583.3 | RNA binding motif, single stranded interacting protein 3 |
| FKBP15 | ENST00000238256.3 | FK506 binding protein 15, 133kDa |
| CD300A | ENST00000360141.3 | CD300a molecule |
| ARHGAP27 | ENST00000532038.1 | Rho GTPase activating protein 27 |
| C1orf204 | ENST00000368102.1 | chromosome 1 open reading frame 204 |
| AP1M1 | ENST00000291439.3 | adaptor-related protein complex 1, mu 1 subunit |
| MYD88 | ENST00000495303.1 | myeloid differentiation primary response 88 |
| ITK | ENST00000422843.3 | IL2-inducible T-cell kinase |
| CRYBG3 | ENST00000389622.2 | beta-gamma crystallin domain containing 3 |
| ADAMTS8 | ENST00000257359.6 | ADAM metallopeptidase with thrombospondin type 1 motif, 8 |
| LRRTM3 | ENST00000361320.4 | leucine rich repeat transmembrane neuronal 3 |
| ST8SIA4 | ENST00000231461.5 | ST8 alpha-N-acetyl-neuraminide alpha-2,8-sialyltransferase 4 |
| SAMD9 | ENST00000379958.2 | sterile alpha motif domain containing 9 |
| TTL | ENST00000233336.6 | tubulin tyrosine ligase |
| CDC25B | ENST00000245960.5 | cell division cycle 25B |
| EFCAB6 | ENST00000356087.4 | EF-hand calcium binding domain 6 |
| ZBTB7C | ENST00000535628.2 | zinc finger and BTB domain containing 7C |
| GPC6 | ENST00000377047.4 | glypican 6 |
| CPEB2 | ENST00000538197.1 | cytoplasmic polyadenylation element binding protein 2 |
| TMA16 | ENST00000358572.5 | translation machinery associated 16 homolog (S. cerevisiae) |
| WDR77 | ENST00000235090.5 | WD repeat domain 77 |
| GJC2 | ENST00000366714.2 | gap junction protein, gamma 2, 47kDa |
| PGGT1B | ENST00000419445.1 | protein geranylgeranyltransferase type I, beta subunit |
| TEDDM1 | ENST00000367565.1 | transmembrane epididymal protein 1 |
| SLC26A1 | ENST00000361661.2 | solute carrier family 26 (anion exchanger), member 1 |
| SFRP4 | ENST00000436072.2 | secreted frizzled-related protein 4 |
| MTMR6 | ENST00000381801.5 | myotubularin related protein 6 |
| CLIC4 | ENST00000374379.4 | chloride intracellular channel 4 |
| TNFSF4 | ENST00000367718.1 | tumor necrosis factor (ligand) superfamily, member 4 |
| CAMSAP2 | ENST00000358823.2 | calmodulin regulated spectrin-associated protein family, member 2 |
| FNIP2 | ENST00000264433.6 | folliculin interacting protein 2 |
| CCDC50 | ENST00000392455.3 | coiled-coil domain containing 50 |
| FAM69A | ENST00000370310.4 | family with sequence similarity 69, member A |
| SMAD1 | ENST00000302085.4 | SMAD family member 1 |
| FAM26E | ENST00000368599.3 | family with sequence similarity 26, member E |
| B3GAT2 | ENST00000230053.6 | beta-1,3-glucuronyltransferase 2 (glucuronosyltransferase S) |
| NECAB1 | ENST00000417640.2 | N-terminal EF-hand calcium binding protein 1 |
| RAB27A | ENST00000396307.2 | RAB27A, member RAS oncogene family |
| SMARCA1 | ENST00000371121.3 | SWI/SNF related, matrix associated, actin dependent regulator of chromatin, subfamily a, member 1 |
| UNC13C | ENST00000545554.1 | unc-13 homolog C (C. elegans) |
| ZNF3 | ENST00000424697.1 | zinc finger protein 3 |
| PCSK2 | ENST00000377899.1 | proprotein convertase subtilisin/kexin type 2 |
| ZPLD1 | ENST00000306176.1 | zona pellucida-like domain containing 1 |
| PTPN18 | ENST00000175756.5 | protein tyrosine phosphatase, non-receptor type 18 (brain-derived) |
| TSPYL1 | ENST00000368608.3 | TSPY-like 1 |
| FOXC2 | ENST00000320354.4 | forkhead box C2 (MFH-1, mesenchyme forkhead 1) |
| CHMP1B | ENST00000526991.2 | charged multivesicular body protein 1B |
| ZNF573 | ENST00000590414.2 | zinc finger protein 573 |
| MON2 | ENST00000546600.1 | MON2 homolog (S. cerevisiae) |
| PTTG1IP | ENST00000397887.3 | pituitary tumor-transforming 1 interacting protein |
| ANO5 | ENST00000324559.8 | anoctamin 5 |
| C17orf50 | ENST00000285023.4 | chromosome 17 open reading frame 50 |
| KLHL1 | ENST00000377844.4 | kelch-like family member 1 |
| ITGB6 | ENST00000283249.2 | integrin, beta 6 |
| C17orf105 | ENST00000449302.3 | chromosome 17 open reading frame 105 |
| NARG2 | ENST00000261520.4 | NMDA receptor regulated 2 |
| GUCD1 | ENST00000447813.2 | guanylyl cyclase domain containing 1 |
| UEVLD | ENST00000541984.1 | UEV and lactate/malate dehyrogenase domains |
| TRIB3 | ENST00000217233.3 | tribbles pseudokinase 3 |
| DSEL | ENST00000310045.7 | dermatan sulfate epimerase-like |
| NEK10 | ENST00000295720.6 | NIMA-related kinase 10 |
| ZNF677 | ENST00000599012.1 | zinc finger protein 677 |
| NETO2 | ENST00000562435.1 | neuropilin (NRP) and tolloid (TLL)-like 2 |
| MID2 | ENST00000262843.6 | midline 2 |
| C5orf24 | ENST00000394976.3 | chromosome 5 open reading frame 24 |
| CORO2A | ENST00000343933.5 | coronin, actin binding protein, 2A |
| EID2 | ENST00000390658.2 | EP300 interacting inhibitor of differentiation 2 |
| MED16 | ENST00000269814.4 | mediator complex subunit 16 |
| NUMB | ENST00000554546.1 | numb homolog (Drosophila) |
| RASSF6 | ENST00000307439.5 | Ras association (RalGDS/AF-6) domain family member 6 |
| LSMEM1 | ENST00000312849.4 | leucine-rich single-pass membrane protein 1 |
| ST6GAL2 | ENST00000361686.4 | ST6 beta-galactosamide alpha-2,6-sialyltranferase 2 |
| THOC1 | ENST00000261600.6 | THO complex 1 |
| MCM3 | ENST00000229854.7 | minichromosome maintenance complex component 3 |
| DSC1 | ENST00000257197.3 | desmocollin 1 |
| CBFA2T3 | ENST00000327483.5 | core-binding factor, runt domain, alpha subunit 2; translocated to, 3 |
| TRMT1L | ENST00000367504.3 | tRNA methyltransferase 1 homolog (S. cerevisiae)-like |
| C6orf211 | ENST00000367294.3 | chromosome 6 open reading frame 211 |
| OXSR1 | ENST00000311806.3 | oxidative stress responsive 1 |
| ASB4 | ENST00000325885.5 | ankyrin repeat and SOCS box containing 4 |
| CLEC1A | ENST00000315330.4 | C-type lectin domain family 1, member A |
| GABPA | ENST00000354828.3 | GA binding protein transcription factor, alpha subunit 60kDa |
| CFHR5 | ENST00000367414.5 | complement factor H-related 5 |
| RELN | ENST00000428762.1 | reelin |
| THEMIS2 | ENST00000373925.1 | thymocyte selection associated family member 2 |
| TGFA | ENST00000295400.6 | transforming growth factor, alpha |
| RIC3 | ENST00000396677.2 | RIC3 acetylcholine receptor chaperone |
| PRRX1 | ENST00000367760.3 | paired related homeobox 1 |
| KLK13 | ENST00000595793.1 | kallikrein-related peptidase 13 |
| SLC16A2 | ENST00000587091.1 | solute carrier family 16, member 2 (thyroid hormone transporter) |
| GALNT6 | ENST00000543196.2 | UDP-N-acetyl-alpha-D-galactosamine:polypeptide N-acetylgalactosaminyltransferase 6 (GalNAc-T6) |
| LRRC32 | ENST00000260061.5 | leucine rich repeat containing 32 |
| ROR2 | ENST00000375708.3 | receptor tyrosine kinase-like orphan receptor 2 |
| KLHL15 | ENST00000328046.8 | kelch-like family member 15 |
| SLC26A10 | ENST00000379218.2 | solute carrier family 26, member 10 |
| QSER1 | ENST00000399302.2 | glutamine and serine rich 1 |
| CDK19 | ENST00000368911.3 | cyclin-dependent kinase 19 |
| DCAF12L1 | ENST00000371126.1 | DDB1 and CUL4 associated factor 12-like 1 |
| FAM92A1 | ENST00000518322.1 | family with sequence similarity 92, member A1 |
| NR2F1 | ENST00000327111.3 | nuclear receptor subfamily 2, group F, member 1 |
| TRNP1 | ENST00000522111.2 | TMF1-regulated nuclear protein 1 |
| TNS4 | ENST00000254051.6 | tensin 4 |
| AVL9 | ENST00000318709.4 | AVL9 homolog (S. cerevisiase) |
| POLK | ENST00000241436.4 | polymerase (DNA directed) kappa |
| NUSAP1 | ENST00000260359.6 | nucleolar and spindle associated protein 1 |
| NPHP1 | ENST00000316534.4 | nephronophthisis 1 (juvenile) |
| EIF3J | ENST00000261868.5 | eukaryotic translation initiation factor 3, subunit J |
| GRIK2 | ENST00000413795.1 | glutamate receptor, ionotropic, kainate 2 |
| NUDT4 | ENST00000337179.5 | nudix (nucleoside diphosphate linked moiety X)-type motif 4 |
| RASGEF1B | ENST00000509081.1 | RasGEF domain family, member 1B |
| SPTLC3 | ENST00000399002.2 | serine palmitoyltransferase, long chain base subunit 3 |
| VTA1 | ENST00000367630.4 | vesicle (multivesicular body) trafficking 1 |
| SYNRG | ENST00000339208.6 | synergin, gamma |
| RMND1 | ENST00000336451.3 | required for meiotic nuclear division 1 homolog (S. cerevisiae) |
| UBXN2A | ENST00000309033.4 | UBX domain protein 2A |
| FICD | ENST00000361549.2 | FIC domain containing |
| ADAMDEC1 | ENST00000256412.4 | ADAM-like, decysin 1 |
| SASH1 | ENST00000367467.3 | SAM and SH3 domain containing 1 |
| SLC12A8 | ENST00000430155.2 | solute carrier family 12, member 8 |
| SRP72 | ENST00000342756.5 | signal recognition particle 72kDa |
| SGPL1 | ENST00000373202.3 | sphingosine-1-phosphate lyase 1 |
| RP4-539M6.19 | ENST00000439838.1 | Uncharacterized protein |
| SCAMP1 | ENST00000538629.1 | secretory carrier membrane protein 1 |
| NCOA3 | ENST00000341724.6 | nuclear receptor coactivator 3 |
| ERLIN1 | ENST00000421367.2 | ER lipid raft associated 1 |
| USP45 | ENST00000392738.2 | ubiquitin specific peptidase 45 |
| KLRC3 | ENST00000381903.2 | killer cell lectin-like receptor subfamily C, member 3 |
| IFIT5 | ENST00000371795.4 | interferon-induced protein with tetratricopeptide repeats 5 |
| ZNF285 | ENST00000330997.4 | zinc finger protein 285 |
| UBE2Z | ENST00000360943.5 | ubiquitin-conjugating enzyme E2Z |
| PTPN11 | ENST00000351677.2 | protein tyrosine phosphatase, non-receptor type 11 |
| EPHA5 | ENST00000273854.3 | EPH receptor A5 |
| ENPEP | ENST00000265162.5 | glutamyl aminopeptidase (aminopeptidase A) |
| AMER2 | ENST00000357816.2 | APC membrane recruitment protein 2 |
| MIS18BP1 | ENST00000310806.4 | MIS18 binding protein 1 |
| RHOJ | ENST00000316754.3 | ras homolog family member J |
| KPNA5 | ENST00000368564.1 | karyopherin alpha 5 (importin alpha 6) |
| CLSPN | ENST00000318121.3 | claspin |
| IL17RD | ENST00000296318.7 | interleukin 17 receptor D |
| MOCS2 | ENST00000450852.3 | molybdenum cofactor synthesis 2 |
| ARHGAP26 | ENST00000378004.3 | Rho GTPase activating protein 26 |
| ZBTB41 | ENST00000367405.4 | zinc finger and BTB domain containing 41 |
| GPR45 | ENST00000258456.1 | G protein-coupled receptor 45 |
| SLAMF8 | ENST00000289707.5 | SLAM family member 8 |
| S1PR2 | ENST00000590320.1 | sphingosine-1-phosphate receptor 2 |
| GPR85 | ENST00000297146.3 | G protein-coupled receptor 85 |
| FAM19A2 | ENST00000416284.3 | family with sequence similarity 19 (chemokine (C-C motif)-like), member A2 |
| C3orf80 | ENST00000326474.3 | chromosome 3 open reading frame 80 |
| EMP2 | ENST00000359543.3 | epithelial membrane protein 2 |
| HIST1H2AK | ENST00000330180.2 | histone cluster 1, H2ak |
| ADAMTS5 | ENST00000284987.5 | ADAM metallopeptidase with thrombospondin type 1 motif, 5 |
| PRKCA | ENST00000413366.3 | protein kinase C, alpha |
| ACAA2 | ENST00000285093.10 | acetyl-CoA acyltransferase 2 |
| CAB39 | ENST00000258418.5 | calcium binding protein 39 |
| GABRA4 | ENST00000264318.3 | gamma-aminobutyric acid (GABA) A receptor, alpha 4 |
| CNOT7 | ENST00000361272.4 | CCR4-NOT transcription complex, subunit 7 |
| RASA2 | ENST00000286364.3 | RAS p21 protein activator 2 |
| ESRRG | ENST00000361525.3 | estrogen-related receptor gamma |
| TRIM46 | ENST00000392451.2 | tripartite motif containing 46 |
| SRPK1 | ENST00000373825.2 | SRSF protein kinase 1 |
| VTI1A | ENST00000393077.2 | vesicle transport through interaction with t-SNAREs 1A |
| UBR1 | ENST00000382177.2 | ubiquitin protein ligase E3 component n-recognin 1 |
| CLDN10 | ENST00000376873.3 | claudin 10 |
| MLLT3 | ENST00000380338.4 | myeloid/lymphoid or mixed-lineage leukemia (trithorax homolog, Drosophila); translocated to, 3 |
| SRFBP1 | ENST00000339397.4 | serum response factor binding protein 1 |
| IDE | ENST00000265986.6 | insulin-degrading enzyme |
| FZD3 | ENST00000240093.3 | frizzled family receptor 3 |
| AKIRIN1 | ENST00000432648.3 | akirin 1 |
| SNTB1 | ENST00000395601.3 | syntrophin, beta 1 (dystrophin-associated protein A1, 59kDa, basic component 1) |
| C15orf52 | ENST00000397536.2 | chromosome 15 open reading frame 52 |
| EXTL2 | ENST00000370113.3 | exostosin-like glycosyltransferase 2 |
| RPP25 | ENST00000322177.5 | ribonuclease P/MRP 25kDa subunit |
| SLC6A3 | ENST00000270349.9 | solute carrier family 6 (neurotransmitter transporter), member 3 |
| GXYLT1 | ENST00000398675.3 | glucoside xylosyltransferase 1 |
| C1orf186 | ENST00000331555.5 | chromosome 1 open reading frame 186 |
| PEG10 | ENST00000482108.1 | paternally expressed 10 |
| PDXDC1 | ENST00000396410.4 | pyridoxal-dependent decarboxylase domain containing 1 |
| C3orf14 | ENST00000494481.1 | chromosome 3 open reading frame 14 |
| POC1B | ENST00000378528.2 | POC1 centriolar protein B |
| IRAK1BP1 | ENST00000369940.2 | interleukin-1 receptor-associated kinase 1 binding protein 1 |
| RBM27 | ENST00000265271.5 | RNA binding motif protein 27 |
| DENND4C | ENST00000380432.2 | DENN/MADD domain containing 4C |
| FAM83C | ENST00000374408.3 | family with sequence similarity 83, member C |
| EPS8L3 | ENST00000361852.4 | EPS8-like 3 |
| BX255923.1 | ENST00000354995.3 | HDCMB45P; Uncharacterized protein |
| TMEM189 | ENST00000557021.1 | transmembrane protein 189 |
| EPM2AIP1 | ENST00000322716.5 | EPM2A (laforin) interacting protein 1 |
| PPP3R1 | ENST00000234310.3 | protein phosphatase 3, regulatory subunit B, alpha |
| LMF2 | ENST00000380796.3 | lipase maturation factor 2 |
| VSTM5 | ENST00000409977.1 | V-set and transmembrane domain containing 5 |
| CYTH4 | ENST00000248901.6 | cytohesin 4 |
| MAP3K7 | ENST00000369325.3 | mitogen-activated protein kinase kinase kinase 7 |
| KBTBD13 | ENST00000432196.2 | kelch repeat and BTB (POZ) domain containing 13 |
| TMEM189-UBE2V1 | ENST00000341698.2 | TMEM189-UBE2V1 readthrough |
| BRCA1 | ENST00000468300.1 | breast cancer 1, early onset |
| THRB | ENST00000396671.2 | thyroid hormone receptor, beta |
| BEND4 | ENST00000504360.1 | BEN domain containing 4 |
| EIF3F | ENST00000533626.1 | eukaryotic translation initiation factor 3, subunit F |
| MKRN3 | ENST00000314520.3 | makorin ring finger protein 3 |
| ZFHX4 | ENST00000521891.2 | zinc finger homeobox 4 |
| CCM2L | ENST00000262659.8 | cerebral cavernous malformation 2-like |
| ZNF91 | ENST00000300619.7 | zinc finger protein 91 |
| PLA2G4F | ENST00000397272.3 | phospholipase A2, group IVF |
| ZNF641 | ENST00000301042.3 | zinc finger protein 641 |
| TMEM109 | ENST00000227525.3 | transmembrane protein 109 |
| FOXP1 | ENST00000318789.4 | forkhead box P1 |
| PTCH2 | ENST00000447098.2 | patched 2 |
| LILRB4 | ENST00000391736.1 | leukocyte immunoglobulin-like receptor, subfamily B (with TM and ITIM domains), member 4 |
| MAN2A1 | ENST00000261483.4 | mannosidase, alpha, class 2A, member 1 |
| FKBP5 | ENST00000536438.1 | FK506 binding protein 5 |
| CCDC38 | ENST00000546386.1 | coiled-coil domain containing 38 |
| CAPZA2 | ENST00000361183.3 | capping protein (actin filament) muscle Z-line, alpha 2 |
| ADRA1D | ENST00000379453.4 | adrenoceptor alpha 1D |
| PPP2CA | ENST00000481195.1 | protein phosphatase 2, catalytic subunit, alpha isozyme |
| TMPRSS11B | ENST00000332644.5 | transmembrane protease, serine 11B |
| TLR3 | ENST00000296795.3 | toll-like receptor 3 |
| MFSD2B | ENST00000338315.4 | major facilitator superfamily domain containing 2B |
| WDR96 | ENST00000369720.1 | WD repeat domain 96 |
| CAPZA1 | ENST00000263168.3 | capping protein (actin filament) muscle Z-line, alpha 1 |
| AC007040.11 | ENST00000606025.1 |  |
| TNPO2 | ENST00000425528.1 | transportin 2 |
| SERP1 | ENST00000239944.2 | stress-associated endoplasmic reticulum protein 1 |
| KIAA1456 | ENST00000524591.2 | KIAA1456 |
| GZF1 | ENST00000338121.5 | GDNF-inducible zinc finger protein 1 |
| BET1L | ENST00000325147.9 | Bet1 golgi vesicular membrane trafficking protein-like |
| SPRTN | ENST00000391858.4 | SprT-like N-terminal domain |
| ZBTB21 | ENST00000398505.3 | zinc finger and BTB domain containing 21 |
| NEGR1 | ENST00000357731.5 | neuronal growth regulator 1 |
| CLEC2L | ENST00000422142.2 | C-type lectin domain family 2, member L |
| B3GALT5 | ENST00000380620.4 | UDP-Gal:betaGlcNAc beta 1,3-galactosyltransferase, polypeptide 5 |
| WWC3 | ENST00000380861.4 | WWC family member 3 |
| MED23 | ENST00000368068.3 | mediator complex subunit 23 |
| KCNH8 | ENST00000328405.2 | potassium voltage-gated channel, subfamily H (eag-related), member 8 |
| FOXF2 | ENST00000259806.1 | forkhead box F2 |
| SLC46A3 | ENST00000266943.6 | solute carrier family 46, member 3 |
| BCL2 | ENST00000398117.1 | B-cell CLL/lymphoma 2 |
| AFF2 | ENST00000370460.2 | AF4/FMR2 family, member 2 |
| RAB3IL1 | ENST00000394836.2 | RAB3A interacting protein (rabin3)-like 1 |
| GAL3ST4 | ENST00000423751.1 | galactose-3-O-sulfotransferase 4 |
| CSGALNACT1 | ENST00000454498.2 | chondroitin sulfate N-acetylgalactosaminyltransferase 1 |
| TBCK | ENST00000273980.5 | TBC1 domain containing kinase |
| LRRC8A | ENST00000372600.4 | leucine rich repeat containing 8 family, member A |
| CDPF1 | ENST00000404744.1 | cysteine-rich, DPF motif domain containing 1 |
| SNAP91 | ENST00000521485.1 | synaptosomal-associated protein, 91kDa |
| RP11-108K14.8 | ENST00000468317.2 | Mitochondrial GTPase 1 |
| ESD | ENST00000378720.3 | esterase D |
| RAB3IP | ENST00000483530.2 | RAB3A interacting protein |
| LMAN1 | ENST00000251047.5 | lectin, mannose-binding, 1 |
| GPR107 | ENST00000372410.3 | G protein-coupled receptor 107 |
| SEMA5A | ENST00000382496.5 | sema domain, seven thrombospondin repeats (type 1 and type 1-like), transmembrane domain (TM) and short cytoplasmic domain, (semaphorin) 5A |
| ACER2 | ENST00000340967.2 | alkaline ceramidase 2 |
| TRPV3 | ENST00000301365.4 | transient receptor potential cation channel, subfamily V, member 3 |
| ONECUT2 | ENST00000491143.2 | one cut homeobox 2 |
| BMS1 | ENST00000374518.5 | BMS1 ribosome biogenesis factor |
| TSPAN11 | ENST00000261177.9 | tetraspanin 11 |
| ROBO4 | ENST00000306534.3 | roundabout, axon guidance receptor, homolog 4 (Drosophila) |
| TMPRSS11A | ENST00000508048.1 | transmembrane protease, serine 11A |
| SEZ6 | ENST00000317338.12 | seizure related 6 homolog (mouse) |
| ZNF687 | ENST00000368879.2 | zinc finger protein 687 |
| KLF10 | ENST00000285407.6 | Kruppel-like factor 10 |
| AC002451.1 | ENST00000601424.1 | Protein LOC100996577 |
| RIMKLA | ENST00000431473.3 | ribosomal modification protein rimK-like family member A |
| ARAP2 | ENST00000303965.4 | ArfGAP with RhoGAP domain, ankyrin repeat and PH domain 2 |
| CNNM3 | ENST00000377060.3 | cyclin M3 |
| CHST3 | ENST00000373115.4 | carbohydrate (chondroitin 6) sulfotransferase 3 |
| AIF1L | ENST00000372300.1 | allograft inflammatory factor 1-like |
| C6orf141 | ENST00000529246.2 | chromosome 6 open reading frame 141 |
| MTG1 | ENST00000317502.6 | mitochondrial ribosome-associated GTPase 1 |
| LPIN1 | ENST00000256720.2 | lipin 1 |
| EHD3 | ENST00000322054.5 | EH-domain containing 3 |
| LCA5 | ENST00000369846.4 | Leber congenital amaurosis 5 |
| TBX20 | ENST00000408931.3 | T-box 20 |
| NBPF3 | ENST00000318220.6 | neuroblastoma breakpoint family, member 3 |
| TAF5L | ENST00000366676.1 | TAF5-like RNA polymerase II, p300/CBP-associated factor (PCAF)-associated factor, 65kDa |
| AMHR2 | ENST00000550311.1 | anti-Mullerian hormone receptor, type II |
| AASDHPPT | ENST00000278618.4 | aminoadipate-semialdehyde dehydrogenase-phosphopantetheinyl transferase |
| FER | ENST00000281092.4 | fer (fps/fes related) tyrosine kinase |
| TTC39B | ENST00000512701.2 | tetratricopeptide repeat domain 39B |
| L2HGDH | ENST00000267436.4 | L-2-hydroxyglutarate dehydrogenase |
| SIRPB1 | ENST00000279477.7 | signal-regulatory protein beta 1 |
| TRAPPC13 | ENST00000399438.3 | trafficking protein particle complex 13 |
| RIMKLB | ENST00000357529.3 | ribosomal modification protein rimK-like family member B |
| STK4 | ENST00000372801.1 | serine/threonine kinase 4 |
| ALKBH5 | ENST00000399138.4 | alkB, alkylation repair homolog 5 (E. coli) |
| HEATR1 | ENST00000366582.3 | HEAT repeat containing 1 |
| GOLGA6D | ENST00000434739.3 | golgin A6 family, member D |
| GJB3 | ENST00000373362.3 | gap junction protein, beta 3, 31kDa |
| MLANA | ENST00000381477.3 | melan-A |
| RBM48 | ENST00000481551.1 | RNA binding motif protein 48 |
| ARL4C | ENST00000390645.2 | ADP-ribosylation factor-like 4C |
| MTCH2 | ENST00000302503.3 | mitochondrial carrier 2 |
| JAK2 | ENST00000381652.3 | Janus kinase 2 |
| MICU3 | ENST00000318063.5 | mitochondrial calcium uptake family, member 3 |
| GSE1 | ENST00000253458.7 | Gse1 coiled-coil protein |
| CXXC4 | ENST00000394767.2 | CXXC finger protein 4 |
| ZFAND5 | ENST00000237937.3 | zinc finger, AN1-type domain 5 |
| ENPP4 | ENST00000321037.4 | ectonucleotide pyrophosphatase/phosphodiesterase 4 (putative) |
| GRIA1 | ENST00000285900.5 | glutamate receptor, ionotropic, AMPA 1 |
| PPP2R5E | ENST00000337537.3 | protein phosphatase 2, regulatory subunit B', epsilon isoform |
| RASA4 | ENST00000262940.7 | RAS p21 protein activator 4 |
| KLF14 | ENST00000310992.4 | Kruppel-like factor 14 |
| ASPA | ENST00000263080.2 | aspartoacylase |
| EPB41L5 | ENST00000443902.2 | erythrocyte membrane protein band 4.1 like 5 |
| PPM1K | ENST00000608933.1 | protein phosphatase, Mg2+/Mn2+ dependent, 1K |
| MOCS1 | ENST00000373186.4 | molybdenum cofactor synthesis 1 |
| C11orf87 | ENST00000327419.6 | chromosome 11 open reading frame 87 |
| SPNS2 | ENST00000329078.3 | spinster homolog 2 (Drosophila) |
| ZNF207 | ENST00000394670.4 | zinc finger protein 207 |
| PTCD1 | ENST00000292478.4 | pentatricopeptide repeat domain 1 |
| LRRC14 | ENST00000292524.1 | leucine rich repeat containing 14 |
| SCD | ENST00000370355.2 | stearoyl-CoA desaturase (delta-9-desaturase) |
| BDP1 | ENST00000380675.2 | B double prime 1, subunit of RNA polymerase III transcription initiation factor IIIB |
| STYK1 | ENST00000075503.3 | serine/threonine/tyrosine kinase 1 |
| LOX | ENST00000231004.4 | lysyl oxidase |
| CBX5 | ENST00000209875.4 | chromobox homolog 5 |
| AMOTL1 | ENST00000317837.9 | angiomotin like 1 |
| STX17 | ENST00000259400.6 | syntaxin 17 |
| RABEP1 | ENST00000262477.6 | rabaptin, RAB GTPase binding effector protein 1 |
| ZC3H13 | ENST00000242848.4 | zinc finger CCCH-type containing 13 |
| RGS7BP | ENST00000334025.2 | regulator of G-protein signaling 7 binding protein |
| RNF186 | ENST00000375121.2 | ring finger protein 186 |
| FGF7 | ENST00000267843.4 | fibroblast growth factor 7 |
| PLSCR4 | ENST00000354952.2 | phospholipid scramblase 4 |
| KIAA0141 | ENST00000194118.4 | KIAA0141 |
| FAM13B | ENST00000033079.3 | family with sequence similarity 13, member B |
| ZNF432 | ENST00000594154.1 | zinc finger protein 432 |
| KIAA0226 | ENST00000273582.5 | KIAA0226 |
| CHODL | ENST00000400128.1 | chondrolectin |
| ITGA4 | ENST00000397033.2 | integrin, alpha 4 (antigen CD49D, alpha 4 subunit of VLA-4 receptor) |
| PRDM5 | ENST00000515109.1 | PR domain containing 5 |
| KIAA0087 | ENST00000242109.3 | KIAA0087 |
| IHH | ENST00000295731.6 | indian hedgehog |
| KIAA1147 | ENST00000536163.1 | KIAA1147 |
| HGF | ENST00000222390.5 | hepatocyte growth factor (hepapoietin A; scatter factor) |
| PPAP2B | ENST00000371250.3 | phosphatidic acid phosphatase type 2B |
| THRAP3 | ENST00000354618.5 | thyroid hormone receptor associated protein 3 |
| DCX | ENST00000356915.2 | doublecortin |
| SLC52A3 | ENST00000381944.3 | solute carrier family 52 (riboflavin transporter), member 3 |
| CLASP2 | ENST00000539981.1 | cytoplasmic linker associated protein 2 |
| TCTA | ENST00000273590.3 | T-cell leukemia translocation altered |
| PPP1R3B | ENST00000310455.3 | protein phosphatase 1, regulatory subunit 3B |
| TMF1 | ENST00000398559.2 | TATA element modulatory factor 1 |
| RP11-383H13.1 | ENST00000521467.1 | Protein LOC100132891; cDNA FLJ53548 |
| GOLGA6C | ENST00000300576.5 | golgin A6 family, member C |
| SLC16A12 | ENST00000341233.4 | solute carrier family 16, member 12 |
| IMPG2 | ENST00000193391.7 | interphotoreceptor matrix proteoglycan 2 |
| POC1B-GALNT4 | ENST00000547474.1 | POC1B-GALNT4 readthrough |
| CPXM2 | ENST00000241305.3 | carboxypeptidase X (M14 family), member 2 |
| SLC22A3 | ENST00000392145.1 | solute carrier family 22 (organic cation transporter), member 3 |
| PRUNE | ENST00000271620.3 | prune exopolyphosphatase |
| CYP7B1 | ENST00000310193.3 | cytochrome P450, family 7, subfamily B, polypeptide 1 |
| PPP4R1L | ENST00000334187.8 | protein phosphatase 4, regulatory subunit 1-like |
| FRYL | ENST00000503238.1 | FRY-like |
| STXBP6 | ENST00000396700.1 | syntaxin binding protein 6 (amisyn) |
| CXorf56 | ENST00000320339.4 | chromosome X open reading frame 56 |
| PAQR5 | ENST00000395407.2 | progestin and adipoQ receptor family member V |
| HIST3H2BB | ENST00000369160.2 | histone cluster 3, H2bb |
| MCFD2 | ENST00000444761.2 | multiple coagulation factor deficiency 2 |
| GPR26 | ENST00000284674.1 | G protein-coupled receptor 26 |
| GJB2 | ENST00000382848.4 | gap junction protein, beta 2, 26kDa |
| ZDHHC17 | ENST00000426126.2 | zinc finger, DHHC-type containing 17 |
| MEIS2 | ENST00000397624.3 | Meis homeobox 2 |
| LDB2 | ENST00000502640.1 | LIM domain binding 2 |
| CD3EAP | ENST00000309424.3 | CD3e molecule, epsilon associated protein |
| CERS6 | ENST00000305747.6 | ceramide synthase 6 |
| AC135983.2 | ENST00000593303.1 | Protein LOC100996413 |
| MTPN | ENST00000393085.3 | myotrophin |
| PPP4R2 | ENST00000356692.5 | protein phosphatase 4, regulatory subunit 2 |
| PIGK | ENST00000370812.3 | phosphatidylinositol glycan anchor biosynthesis, class K |
| CAV1 | ENST00000405348.1 | caveolin 1, caveolae protein, 22kDa |
| PANK3 | ENST00000239231.6 | pantothenate kinase 3 |
| FCHSD2 | ENST00000311172.7 | FCH and double SH3 domains 2 |
| DHDDS | ENST00000360009.2 | dehydrodolichyl diphosphate synthase |
| SEC14L5 | ENST00000251170.7 | SEC14-like 5 (S. cerevisiae) |
| ASCC3 | ENST00000369162.2 | activating signal cointegrator 1 complex subunit 3 |
| SLC13A1 | ENST00000194130.2 | solute carrier family 13 (sodium/sulfate symporter), member 1 |
| PRRG4 | ENST00000257836.3 | proline rich Gla (G-carboxyglutamic acid) 4 (transmembrane) |
| MYO1A | ENST00000300119.3 | myosin IA |
| CD59 | ENST00000395850.3 | CD59 molecule, complement regulatory protein |
| LOH12CR1 | ENST00000314565.4 | loss of heterozygosity, 12, chromosomal region 1 |
| KIAA0586 | ENST00000423743.3 | KIAA0586 |
| KIF27 | ENST00000297814.2 | kinesin family member 27 |
| CYP19A1 | ENST00000396402.1 | cytochrome P450, family 19, subfamily A, polypeptide 1 |
| BOD1L2 | ENST00000585477.1 | biorientation of chromosomes in cell division 1-like 2 |
| NOX5 | ENST00000260364.5 | NADPH oxidase, EF-hand calcium binding domain 5 |
| ZNF470 | ENST00000330619.8 | zinc finger protein 470 |
| GPR137C | ENST00000321662.6 | G protein-coupled receptor 137C |
| OSBPL6 | ENST00000190611.4 | oxysterol binding protein-like 6 |
| NUP62 | ENST00000422090.2 | nucleoporin 62kDa |
| SYT6 | ENST00000607941.1 | synaptotagmin VI |
| SOCS4 | ENST00000395472.2 | suppressor of cytokine signaling 4 |
| SPATA13 | ENST00000382108.3 | spermatogenesis associated 13 |
| TACC1 | ENST00000330691.6 | transforming, acidic coiled-coil containing protein 1 |
| MAPK13 | ENST00000373766.5 | mitogen-activated protein kinase 13 |
| TMEM255A | ENST00000309720.5 | transmembrane protein 255A |
| ERMAP | ENST00000372514.3 | erythroblast membrane-associated protein (Scianna blood group) |
| HLF | ENST00000226067.5 | hepatic leukemia factor |
| NUP50 | ENST00000347635.4 | nucleoporin 50kDa |
| FAM135B | ENST00000395297.1 | family with sequence similarity 135, member B |
| C14orf105 | ENST00000216445.3 | chromosome 14 open reading frame 105 |
| MGRN1 | ENST00000415496.1 | mahogunin ring finger 1, E3 ubiquitin protein ligase |
| HS6ST1 | ENST00000259241.6 | heparan sulfate 6-O-sulfotransferase 1 |
| MOSPD2 | ENST00000380492.3 | motile sperm domain containing 2 |
| NOVA1 | ENST00000465357.2 | neuro-oncological ventral antigen 1 |
| NUP85 | ENST00000447371.2 | nucleoporin 85kDa |
| XK | ENST00000378616.3 | X-linked Kx blood group (McLeod syndrome) |
| LPP | ENST00000312675.4 | LIM domain containing preferred translocation partner in lipoma |
| BBS7 | ENST00000264499.4 | Bardet-Biedl syndrome 7 |
| CACNG4 | ENST00000262138.3 | calcium channel, voltage-dependent, gamma subunit 4 |
| TANC2 | ENST00000424789.2 | tetratricopeptide repeat, ankyrin repeat and coiled-coil containing 2 |
| RP11-766F14.2 | ENST00000511828.1 | Protein LOC285556 |
| USP7 | ENST00000344836.4 | ubiquitin specific peptidase 7 (herpes virus-associated) |
| PGBD5 | ENST00000391860.1 | piggyBac transposable element derived 5 |
| FAM204A | ENST00000369183.4 | family with sequence similarity 204, member A |
| CREBRF | ENST00000540014.1 | CREB3 regulatory factor |
| ZBTB10 | ENST00000430430.1 | zinc finger and BTB domain containing 10 |
| CREBZF | ENST00000398294.2 | CREB/ATF bZIP transcription factor |
| DUSP28 | ENST00000405954.1 | dual specificity phosphatase 28 |
| NPR3 | ENST00000265074.8 | natriuretic peptide receptor C/guanylate cyclase C (atrionatriuretic peptide receptor C) |
| PCNX | ENST00000304743.2 | pecanex homolog (Drosophila) |
| FAIM2 | ENST00000320634.3 | Fas apoptotic inhibitory molecule 2 |
| PDE5A | ENST00000354960.3 | phosphodiesterase 5A, cGMP-specific |
| DDX3Y | ENST00000336079.3 | DEAD (Asp-Glu-Ala-Asp) box helicase 3, Y-linked |
| XCR1 | ENST00000309285.3 | chemokine (C motif) receptor 1 |
| CCR5 | ENST00000292303.4 | chemokine (C-C motif) receptor 5 (gene/pseudogene) |
| AP003068.23 | ENST00000526623.1 | Uncharacterized protein |
| ICMT | ENST00000343813.5 | isoprenylcysteine carboxyl methyltransferase |
| PHACTR4 | ENST00000373839.3 | phosphatase and actin regulator 4 |
| GAB1 | ENST00000262995.4 | GRB2-associated binding protein 1 |
| CHDH | ENST00000315251.6 | choline dehydrogenase |
| ACSL1 | ENST00000454703.2 | acyl-CoA synthetase long-chain family member 1 |
| DLC1 | ENST00000276297.4 | deleted in liver cancer 1 |
| DAB1 | ENST00000371236.2 | Dab, reelin signal transducer, homolog 1 (Drosophila) |
| DPY19L4 | ENST00000414645.2 | dpy-19-like 4 (C. elegans) |
| XIAP | ENST00000371199.3 | X-linked inhibitor of apoptosis |
| SMEK1 | ENST00000554684.1 | SMEK homolog 1, suppressor of mek1 (Dictyostelium) |
| ZNF514 | ENST00000295208.2 | zinc finger protein 514 |
| SUDS3 | ENST00000397564.2 | suppressor of defective silencing 3 homolog (S. cerevisiae) |
| GCFC2 | ENST00000541687.1 | GC-rich sequence DNA-binding factor 2 |
| RPGR | ENST00000338898.3 | retinitis pigmentosa GTPase regulator |
| TMPPE | ENST00000416695.2 | transmembrane protein with metallophosphoesterase domain |
| RABIF | ENST00000367262.3 | RAB interacting factor |
| FBXO21 | ENST00000427718.2 | F-box protein 21 |
| BICD1 | ENST00000548411.1 | bicaudal D homolog 1 (Drosophila) |
| AGGF1 | ENST00000312916.7 | angiogenic factor with G patch and FHA domains 1 |
| UBASH3B | ENST00000284273.5 | ubiquitin associated and SH3 domain containing B |
| DOCK7 | ENST00000251157.5 | dedicator of cytokinesis 7 |
| SLIT2 | ENST00000504154.1 | slit homolog 2 (Drosophila) |
| LARP4 | ENST00000398473.2 | La ribonucleoprotein domain family, member 4 |
| GPR158 | ENST00000376351.3 | G protein-coupled receptor 158 |
| GPHN | ENST00000478722.1 | gephyrin |
| SLC41A2 | ENST00000258538.3 | solute carrier family 41 (magnesium transporter), member 2 |
| CYP27C1 | ENST00000335247.7 | cytochrome P450, family 27, subfamily C, polypeptide 1 |
| SLC37A1 | ENST00000398341.3 | solute carrier family 37 (glucose-6-phosphate transporter), member 1 |
| GSTM5 | ENST00000369813.1 | glutathione S-transferase mu 5 |
| SUSD5 | ENST00000309558.3 | sushi domain containing 5 |
| SLC23A2 | ENST00000338244.1 | solute carrier family 23 (ascorbic acid transporter), member 2 |
| TSHZ2 | ENST00000371497.5 | teashirt zinc finger homeobox 2 |
| RNF7 | ENST00000273480.3 | ring finger protein 7 |
| UHMK1 | ENST00000538489.1 | U2AF homology motif (UHM) kinase 1 |
| DTHD1 | ENST00000456874.2 | death domain containing 1 |
| ASAH1 | ENST00000262097.6 | N-acylsphingosine amidohydrolase (acid ceramidase) 1 |
| VWC2 | ENST00000340652.4 | von Willebrand factor C domain containing 2 |
| FSTL4 | ENST00000265342.7 | follistatin-like 4 |
| MXD1 | ENST00000264444.2 | MAX dimerization protein 1 |
| ASB14 | ENST00000487349.1 | ankyrin repeat and SOCS box containing 14 |
| PCSK1 | ENST00000311106.3 | proprotein convertase subtilisin/kexin type 1 |
| TMEM43 | ENST00000306077.4 | transmembrane protein 43 |
| PRPF38A | ENST00000257181.9 | pre-mRNA processing factor 38A |
| TCF7 | ENST00000518915.1 | transcription factor 7 (T-cell specific, HMG-box) |
| AMT | ENST00000458307.2 | aminomethyltransferase |
| ZNF705G | ENST00000400156.4 | zinc finger protein 705G |
| USP12 | ENST00000282344.6 | ubiquitin specific peptidase 12 |
| SOCS2 | ENST00000548537.1 | suppressor of cytokine signaling 2 |
| RUNX1T1 | ENST00000523629.1 | runt-related transcription factor 1; translocated to, 1 (cyclin D-related) |
| OPRK1 | ENST00000265572.3 | opioid receptor, kappa 1 |
| CAMK2A | ENST00000348628.6 | calcium/calmodulin-dependent protein kinase II alpha |
| ABCC11 | ENST00000353782.5 | ATP-binding cassette, sub-family C (CFTR/MRP), member 11 |
| TJAP1 | ENST00000372444.2 | tight junction associated protein 1 (peripheral) |
| FLRT3 | ENST00000378053.3 | fibronectin leucine rich transmembrane protein 3 |
| RORA | ENST00000335670.6 | RAR-related orphan receptor A |
| CR769776.1 | ENST00000414341.2 | LOC441426 protein; Uncharacterized protein |
| HNMT | ENST00000280097.3 | histamine N-methyltransferase |
| RAP2A | ENST00000245304.4 | RAP2A, member of RAS oncogene family |
| CCDC90B | ENST00000529689.1 | coiled-coil domain containing 90B |
| PPP1R9A | ENST00000289495.5 | protein phosphatase 1, regulatory subunit 9A |
| CYCS | ENST00000305786.2 | cytochrome c, somatic |
| IL6ST | ENST00000381287.4 | interleukin 6 signal transducer (gp130, oncostatin M receptor) |
| TCF19 | ENST00000376257.3 | transcription factor 19 |
| SERINC4 | ENST00000299969.6 | serine incorporator 4 |
| TMEM55A | ENST00000285419.3 | transmembrane protein 55A |
| ZNF19 | ENST00000288177.5 | zinc finger protein 19 |
| MGEA5 | ENST00000439817.1 | meningioma expressed antigen 5 (hyaluronidase) |
| RNMT | ENST00000383314.2 | RNA (guanine-7-) methyltransferase |
| AC027763.2 | ENST00000399540.2 | Uncharacterized protein |
| SEH1L | ENST00000262124.11 | SEH1-like (S. cerevisiae) |
| PDE10A | ENST00000366882.1 | phosphodiesterase 10A |
| OGDHL | ENST00000374103.4 | oxoglutarate dehydrogenase-like |
| DPP10 | ENST00000410059.1 | dipeptidyl-peptidase 10 (non-functional) |
| TMEM40 | ENST00000314124.7 | transmembrane protein 40 |
| KCNQ4 | ENST00000347132.5 | potassium voltage-gated channel, KQT-like subfamily, member 4 |
| RNF128 | ENST00000324342.3 | ring finger protein 128, E3 ubiquitin protein ligase |
| SIRPG | ENST00000381580.1 | signal-regulatory protein gamma |
| ZNF546 | ENST00000347077.4 | zinc finger protein 546 |
| KIAA1549 | ENST00000440172.1 | KIAA1549 |
| TAF1D | ENST00000448108.2 | TATA box binding protein (TBP)-associated factor, RNA polymerase I, D, 41kDa |
| MYO1B | ENST00000339514.4 | myosin IB |
| RNF217 | ENST00000521654.2 | ring finger protein 217 |
| SLC4A8 | ENST00000453097.2 | solute carrier family 4, sodium bicarbonate cotransporter, member 8 |
| ZFP14 | ENST00000270001.7 | ZFP14 zinc finger protein |
| ADRB1 | ENST00000369295.2 | adrenoceptor beta 1 |
| KDR | ENST00000263923.4 | kinase insert domain receptor (a type III receptor tyrosine kinase) |
| ENPP6 | ENST00000296741.2 | ectonucleotide pyrophosphatase/phosphodiesterase 6 |
| RHOBTB3 | ENST00000379982.3 | Rho-related BTB domain containing 3 |
| WNK1 | ENST00000315939.6 | WNK lysine deficient protein kinase 1 |
| NUGGC | ENST00000413272.2 | nuclear GTPase, germinal center associated |
| KCNA6 | ENST00000433855.1 | potassium voltage-gated channel, shaker-related subfamily, member 6 |
| HHIP | ENST00000296575.3 | hedgehog interacting protein |
| OPA1 | ENST00000392438.3 | optic atrophy 1 (autosomal dominant) |
| KIAA1958 | ENST00000337530.6 | KIAA1958 |
| PPP1R3E | ENST00000452015.4 | protein phosphatase 1, regulatory subunit 3E |
| ZMAT3 | ENST00000311417.2 | zinc finger, matrin-type 3 |
| AP5M1 | ENST00000261558.3 | adaptor-related protein complex 5, mu 1 subunit |
| ROR1 | ENST00000371079.1 | receptor tyrosine kinase-like orphan receptor 1 |
| RAG1 | ENST00000299440.5 | recombination activating gene 1 |
| GPR111 | ENST00000398742.2 | G protein-coupled receptor 111 |
| ADAMTS3 | ENST00000286657.4 | ADAM metallopeptidase with thrombospondin type 1 motif, 3 |
| PLEKHA8 | ENST00000449726.1 | pleckstrin homology domain containing, family A (phosphoinositide binding specific) member 8 |
| SPTSSA | ENST00000298130.4 | serine palmitoyltransferase, small subunit A |
| ATL2 | ENST00000406122.1 | atlastin GTPase 2 |
| SLC9B2 | ENST00000503103.1 | solute carrier family 9, subfamily B (NHA2, cation proton antiporter 2), member 2 |
| LIN7C | ENST00000278193.2 | lin-7 homolog C (C. elegans) |
| CAMK4 | ENST00000282356.4 | calcium/calmodulin-dependent protein kinase IV |
| ANGPTL2 | ENST00000373425.3 | angiopoietin-like 2 |
| TSPYL5 | ENST00000322128.3 | TSPY-like 5 |
| EPM2A | ENST00000367519.3 | epilepsy, progressive myoclonus type 2A, Lafora disease (laforin) |
| LIN7A | ENST00000552864.1 | lin-7 homolog A (C. elegans) |
| GPR83 | ENST00000243673.2 | G protein-coupled receptor 83 |
| AIMP1 | ENST00000394701.4 | aminoacyl tRNA synthetase complex-interacting multifunctional protein 1 |
| P2RY1 | ENST00000305097.3 | purinergic receptor P2Y, G-protein coupled, 1 |
| LMO4 | ENST00000370544.5 | LIM domain only 4 |
| GREM1 | ENST00000560677.1 | gremlin 1, DAN family BMP antagonist |
| SZRD1 | ENST00000401089.3 | SUZ RNA binding domain containing 1 |
| EHBP1 | ENST00000431489.1 | EH domain binding protein 1 |
| KIF16B | ENST00000354981.2 | kinesin family member 16B |
| ZNF532 | ENST00000336078.4 | zinc finger protein 532 |
| SP1 | ENST00000426431.2 | Sp1 transcription factor |
| MTMR1 | ENST00000445323.2 | myotubularin related protein 1 |
| APOL4 | ENST00000404685.3 | apolipoprotein L, 4 |
| GLTSCR1L | ENST00000314073.5 | GLTSCR1-like |
| ALDH3B2 | ENST00000530069.1 | aldehyde dehydrogenase 3 family, member B2 |
| CUL4A | ENST00000326335.4 | cullin 4A |
| NUP210 | ENST00000254508.5 | nucleoporin 210kDa |
| ATXN3 | ENST00000545170.1 | ataxin 3 |
| IMPAD1 | ENST00000262644.4 | inositol monophosphatase domain containing 1 |
| INPP4A | ENST00000409016.4 | inositol polyphosphate-4-phosphatase, type I, 107kDa |
| NT5C2 | ENST00000343289.5 | 5'-nucleotidase, cytosolic II |
| NR2C2 | ENST00000425241.1 | nuclear receptor subfamily 2, group C, member 2 |
| SEC23IP | ENST00000369075.3 | SEC23 interacting protein |
| PCGF3 | ENST00000362003.5 | polycomb group ring finger 3 |
| SCAI | ENST00000336505.6 | suppressor of cancer cell invasion |
| TXNRD2 | ENST00000535882.1 | thioredoxin reductase 2 |
| KIAA0895 | ENST00000297063.6 | KIAA0895 |
| IL2RA | ENST00000379959.3 | interleukin 2 receptor, alpha |
| OLFM3 | ENST00000370103.4 | olfactomedin 3 |
| BNC1 | ENST00000345382.2 | basonuclin 1 |
| MYOZ3 | ENST00000297130.4 | myozenin 3 |
| SFMBT2 | ENST00000361972.4 | Scm-like with four mbt domains 2 |
| ACAP2 | ENST00000326793.6 | ArfGAP with coiled-coil, ankyrin repeat and PH domains 2 |
| CEP72 | ENST00000444221.1 | centrosomal protein 72kDa |
| HIPK2 | ENST00000406875.3 | homeodomain interacting protein kinase 2 |
| GBP2 | ENST00000370466.3 | guanylate binding protein 2, interferon-inducible |
| AS3MT | ENST00000369880.3 | arsenic (+3 oxidation state) methyltransferase |
| TBC1D8B | ENST00000357242.5 | TBC1 domain family, member 8B (with GRAM domain) |
| IGFN1 | ENST00000295591.8 | immunoglobulin-like and fibronectin type III domain containing 1 |
| MUC22 | ENST00000561890.1 | mucin 22 |
| ZFYVE9 | ENST00000371591.1 | zinc finger, FYVE domain containing 9 |
| NTRK2 | ENST00000395882.1 | neurotrophic tyrosine kinase, receptor, type 2 |
| RASAL2 | ENST00000448150.3 | RAS protein activator like 2 |
| PRDM10 | ENST00000358825.5 | PR domain containing 10 |
| APOL6 | ENST00000409652.4 | apolipoprotein L, 6 |
| CREBL2 | ENST00000228865.2 | cAMP responsive element binding protein-like 2 |
| PLEKHG3 | ENST00000247226.7 | pleckstrin homology domain containing, family G (with RhoGef domain) member 3 |
| CD300LB | ENST00000392621.1 | CD300 molecule-like family member b |
| AKAP2 | ENST00000374525.1 | A kinase (PRKA) anchor protein 2 |
| FAM57A | ENST00000308278.8 | family with sequence similarity 57, member A |
| ATP5J2-PTCD1 | ENST00000413834.1 | ATP5J2-PTCD1 readthrough |
| PDK3 | ENST00000441463.2 | pyruvate dehydrogenase kinase, isozyme 3 |
| HP1BP3 | ENST00000312239.5 | heterochromatin protein 1, binding protein 3 |
| ELAVL4 | ENST00000371824.1 | ELAV like neuron-specific RNA binding protein 4 |
| SYT12 | ENST00000393946.2 | synaptotagmin XII |
| CNTNAP5 | ENST00000431078.1 | contactin associated protein-like 5 |
| MYOCD | ENST00000425538.1 | myocardin |
| ZNF398 | ENST00000420008.2 | zinc finger protein 398 |
| GTF3C4 | ENST00000372146.4 | general transcription factor IIIC, polypeptide 4, 90kDa |
| KIAA1324 | ENST00000369939.3 | KIAA1324 |
| RPSAP58 | ENST00000496398.1 | ribosomal protein SA pseudogene 58 |
| SCG5 | ENST00000300175.4 | secretogranin V (7B2 protein) |
| ROBO1 | ENST00000436010.2 | roundabout, axon guidance receptor, homolog 1 (Drosophila) |
| BRINP2 | ENST00000361539.4 | bone morphogenetic protein/retinoic acid inducible neural-specific 2 |
| PNN | ENST00000216832.4 | pinin, desmosome associated protein |
| ZDHHC7 | ENST00000313732.4 | zinc finger, DHHC-type containing 7 |
| ARL11 | ENST00000282026.1 | ADP-ribosylation factor-like 11 |
| TMEM178B | ENST00000565468.1 | transmembrane protein 178B |
| ZNF85 | ENST00000328178.8 | zinc finger protein 85 |
| CPM | ENST00000551568.1 | carboxypeptidase M |
| WDR1 | ENST00000499869.2 | WD repeat domain 1 |
| STK39 | ENST00000355999.4 | serine threonine kinase 39 |
| VN1R1 | ENST00000321039.3 | vomeronasal 1 receptor 1 |
| ZNF2 | ENST00000398107.2 | zinc finger protein 2 |
| LPCAT2 | ENST00000262134.5 | lysophosphatidylcholine acyltransferase 2 |
| ZNF224 | ENST00000336976.6 | zinc finger protein 224 |
| PRPF40A | ENST00000410080.1 | PRP40 pre-mRNA processing factor 40 homolog A (S. cerevisiae) |
| GMNC | ENST00000442080.1 | geminin coiled-coil domain containing |
| LITAF | ENST00000413364.2 | lipopolysaccharide-induced TNF factor |
| PROX2 | ENST00000556084.2 | prospero homeobox 2 |
| CORO2B | ENST00000261861.5 | coronin, actin binding protein, 2B |
| FRMD8 | ENST00000317568.5 | FERM domain containing 8 |
| FCGR2A | ENST00000271450.6 | Fc fragment of IgG, low affinity IIa, receptor (CD32) |
| FAM53B | ENST00000337318.3 | family with sequence similarity 53, member B |
| ZFAND3 | ENST00000287218.4 | zinc finger, AN1-type domain 3 |
| APBB2 | ENST00000295974.8 | amyloid beta (A4) precursor protein-binding, family B, member 2 |
| HIPK3 | ENST00000303296.4 | homeodomain interacting protein kinase 3 |
| MET | ENST00000397752.3 | met proto-oncogene |
| HNF4G | ENST00000396423.2 | hepatocyte nuclear factor 4, gamma |
| ZMYM5 | ENST00000382907.4 | zinc finger, MYM-type 5 |
| SNX19 | ENST00000265909.4 | sorting nexin 19 |
| ZNF827 | ENST00000379448.4 | zinc finger protein 827 |
| TMTC4 | ENST00000376234.3 | transmembrane and tetratricopeptide repeat containing 4 |
| RP11-122A3.2 | ENST00000517562.2 | uncharacterized protein LOC100127983 |
| RAP1GDS1 | ENST00000408927.3 | RAP1, GTP-GDP dissociation stimulator 1 |
| USP13 | ENST00000263966.3 | ubiquitin specific peptidase 13 (isopeptidase T-3) |
| COPS8 | ENST00000354371.2 | COP9 signalosome subunit 8 |
| ZFYVE16 | ENST00000338008.5 | zinc finger, FYVE domain containing 16 |
| SREK1 | ENST00000334121.6 | splicing regulatory glutamine/lysine-rich protein 1 |
| RORB | ENST00000376896.3 | RAR-related orphan receptor B |
| SHROOM4 | ENST00000376020.2 | shroom family member 4 |
| CHRDL1 | ENST00000372045.1 | chordin-like 1 |
| SOX4 | ENST00000244745.1 | SRY (sex determining region Y)-box 4 |
| RAB30 | ENST00000533486.1 | RAB30, member RAS oncogene family |
| CACNA2D2 | ENST00000423994.2 | calcium channel, voltage-dependent, alpha 2/delta subunit 2 |
| LGR5 | ENST00000266674.5 | leucine-rich repeat containing G protein-coupled receptor 5 |
| GOLGA3 | ENST00000204726.3 | golgin A3 |
| SMC3 | ENST00000361804.4 | structural maintenance of chromosomes 3 |
| BCL2L11 | ENST00000393256.3 | BCL2-like 11 (apoptosis facilitator) |
| PARD6G | ENST00000353265.3 | par-6 family cell polarity regulator gamma |
| KSR2 | ENST00000425217.1 | kinase suppressor of ras 2 |
| TBX18 | ENST00000369663.5 | T-box 18 |
| CCND1 | ENST00000227507.2 | cyclin D1 |
| POGZ | ENST00000392723.1 | pogo transposable element with ZNF domain |
| RFX2 | ENST00000303657.5 | regulatory factor X, 2 (influences HLA class II expression) |
| PLD5 | ENST00000536534.2 | phospholipase D family, member 5 |
| PNPLA1 | ENST00000388715.3 | patatin-like phospholipase domain containing 1 |
| DCT | ENST00000377028.5 | dopachrome tautomerase |
| SLX4IP | ENST00000334534.5 | SLX4 interacting protein |
| KIAA1211 | ENST00000504228.1 | KIAA1211 |
| SPIN4 | ENST00000374884.2 | spindlin family, member 4 |
| VAPA | ENST00000400000.2 | VAMP (vesicle-associated membrane protein)-associated protein A, 33kDa |
| CTTNBP2NL | ENST00000271277.6 | CTTNBP2 N-terminal like |
| PEX6 | ENST00000244546.4 | peroxisomal biogenesis factor 6 |
| DIDO1 | ENST00000266070.4 | death inducer-obliterator 1 |
| YPEL1 | ENST00000339468.3 | yippee-like 1 (Drosophila) |
| ADCY7 | ENST00000394697.2 | adenylate cyclase 7 |
| FAM219B | ENST00000357635.5 | family with sequence similarity 219, member B |
| KIAA0922 | ENST00000409663.3 | KIAA0922 |
| ATP13A3 | ENST00000439040.1 | ATPase type 13A3 |
| FPGT | ENST00000370894.5 | fucose-1-phosphate guanylyltransferase |
| PALM2-AKAP2 | ENST00000374530.3 | PALM2-AKAP2 readthrough |
| TBRG1 | ENST00000441174.3 | transforming growth factor beta regulator 1 |
| PTPRC | ENST00000367376.2 | protein tyrosine phosphatase, receptor type, C |
| UBE3B | ENST00000434735.2 | ubiquitin protein ligase E3B |
| CCDC141 | ENST00000420890.2 | coiled-coil domain containing 141 |
| PRRG3 | ENST00000370353.3 | proline rich Gla (G-carboxyglutamic acid) 3 (transmembrane) |
| PODXL | ENST00000541194.1 | podocalyxin-like |
| CEP350 | ENST00000367607.3 | centrosomal protein 350kDa |
| TRIM33 | ENST00000358465.2 | tripartite motif containing 33 |
| RNF115 | ENST00000369291.5 | ring finger protein 115 |
| PDE12 | ENST00000311180.8 | phosphodiesterase 12 |
| TRIM25 | ENST00000316881.4 | tripartite motif containing 25 |
| RCOR3 | ENST00000367006.4 | REST corepressor 3 |
| ALX1 | ENST00000316824.3 | ALX homeobox 1 |
| KLHL14 | ENST00000359358.4 | kelch-like family member 14 |
| BHLHE22 | ENST00000321870.1 | basic helix-loop-helix family, member e22 |
| LINC00346 | ENST00000538077.1 | long intergenic non-protein coding RNA 346 |
| ABCA1 | ENST00000374736.3 | ATP-binding cassette, sub-family A (ABC1), member 1 |
| ADIPOQ | ENST00000412955.2 | adiponectin, C1Q and collagen domain containing |
| CHL1 | ENST00000256509.2 | cell adhesion molecule L1-like |
| SH2B3 | ENST00000341259.2 | SH2B adaptor protein 3 |
| ZIC4 | ENST00000383075.3 | Zic family member 4 |
| PGM3 | ENST00000513973.1 | phosphoglucomutase 3 |
| ANGPT1 | ENST00000520734.1 | angiopoietin 1 |
| BCAT1 | ENST00000261192.7 | branched chain amino-acid transaminase 1, cytosolic |
| CECR2 | ENST00000400573.5 | cat eye syndrome chromosome region, candidate 2 |
| RABGAP1L | ENST00000489615.1 | RAB GTPase activating protein 1-like |
| KIAA0232 | ENST00000425103.1 | KIAA0232 |
| ATP5E | ENST00000243997.3 | ATP synthase, H+ transporting, mitochondrial F1 complex, epsilon subunit |
| RPS6KA6 | ENST00000262752.2 | ribosomal protein S6 kinase, 90kDa, polypeptide 6 |
| FMR1 | ENST00000370471.3 | fragile X mental retardation 1 |
| PLCXD3 | ENST00000377801.3 | phosphatidylinositol-specific phospholipase C, X domain containing 3 |
| CCDC80 | ENST00000206423.3 | coiled-coil domain containing 80 |
| SYNM | ENST00000560674.1 | synemin, intermediate filament protein |
| CSTF1 | ENST00000217109.4 | cleavage stimulation factor, 3' pre-RNA, subunit 1, 50kDa |
| SLC36A4 | ENST00000326402.4 | solute carrier family 36 (proton/amino acid symporter), member 4 |
| DRAXIN | ENST00000294485.5 | dorsal inhibitory axon guidance protein |
| ZSCAN12 | ENST00000361028.1 | zinc finger and SCAN domain containing 12 |
| CHRNB1 | ENST00000306071.2 | cholinergic receptor, nicotinic, beta 1 (muscle) |
| LRRC20 | ENST00000355790.4 | leucine rich repeat containing 20 |
| PAPLN | ENST00000381166.3 | papilin, proteoglycan-like sulfated glycoprotein |
| PHACTR1 | ENST00000379350.1 | phosphatase and actin regulator 1 |
| HPSE2 | ENST00000370546.1 | heparanase 2 |
| EIF5AL1 | ENST00000520547.2 | eukaryotic translation initiation factor 5A-like 1 |
| UBE2N | ENST00000550657.1 | ubiquitin-conjugating enzyme E2N |
| SERBP1 | ENST00000370994.4 | SERPINE1 mRNA binding protein 1 |
| PTPLAD1 | ENST00000261875.5 | protein tyrosine phosphatase-like A domain containing 1 |
| RAPH1 | ENST00000319170.5 | Ras association (RalGDS/AF-6) and pleckstrin homology domains 1 |
| TSGA10 | ENST00000393483.3 | testis specific, 10 |
| RNF152 | ENST00000312828.3 | ring finger protein 152 |
| EML6 | ENST00000356458.6 | echinoderm microtubule associated protein like 6 |
| AP3M1 | ENST00000355264.4 | adaptor-related protein complex 3, mu 1 subunit |
| CLIP1 | ENST00000361654.4 | CAP-GLY domain containing linker protein 1 |
| PPP1R12B | ENST00000608999.1 | protein phosphatase 1, regulatory subunit 12B |
| MBP | ENST00000397863.1 | myelin basic protein |
| ACACA | ENST00000353139.5 | acetyl-CoA carboxylase alpha |
| PIGL | ENST00000225609.5 | phosphatidylinositol glycan anchor biosynthesis, class L |
| RAB23 | ENST00000317483.3 | RAB23, member RAS oncogene family |
| CDH6 | ENST00000265071.2 | cadherin 6, type 2, K-cadherin (fetal kidney) |
| AIRE | ENST00000329347.4 | autoimmune regulator |
| ABCC12 | ENST00000416054.1 | ATP-binding cassette, sub-family C (CFTR/MRP), member 12 |
| AMIGO3 | ENST00000320431.7 | adhesion molecule with Ig-like domain 3 |
| LARGE | ENST00000354992.2 | like-glycosyltransferase |
| RNF2 | ENST00000367510.3 | ring finger protein 2 |
| KANK4 | ENST00000317477.4 | KN motif and ankyrin repeat domains 4 |
| ZIC1 | ENST00000282928.4 | Zic family member 1 |
| USP24 | ENST00000294383.6 | ubiquitin specific peptidase 24 |
| CHSY1 | ENST00000254190.3 | chondroitin sulfate synthase 1 |
| MAP1B | ENST00000296755.7 | microtubule-associated protein 1B |
| SERPINB1 | ENST00000380739.5 | serpin peptidase inhibitor, clade B (ovalbumin), member 1 |
| ZNF557 | ENST00000414706.1 | zinc finger protein 557 |
| MAP2 | ENST00000360351.4 | microtubule-associated protein 2 |
| UHRF1BP1 | ENST00000192788.5 | UHRF1 binding protein 1 |
| MATR3 | ENST00000510056.1 | matrin 3 |
| SMAD9 | ENST00000399275.2 | SMAD family member 9 |
| KIAA1161 | ENST00000297625.7 | KIAA1161 |
| PRKCQ | ENST00000263125.5 | protein kinase C, theta |
| HIF3A | ENST00000377670.4 | hypoxia inducible factor 3, alpha subunit |
| ZNF844 | ENST00000439326.3 | zinc finger protein 844 |
| SEPT3 | ENST00000396425.3 | septin 3 |
| SHF | ENST00000560540.1 | Src homology 2 domain containing F |
| DNAJC3 | ENST00000602402.1 | DnaJ (Hsp40) homolog, subfamily C, member 3 |
| OLIG1 | ENST00000382348.1 | oligodendrocyte transcription factor 1 |
| RPS6KC1 | ENST00000366960.3 | ribosomal protein S6 kinase, 52kDa, polypeptide 1 |
| ATP7A | ENST00000343533.5 | ATPase, Cu++ transporting, alpha polypeptide |
| EEF1A1 | ENST00000316292.9 | eukaryotic translation elongation factor 1 alpha 1 |
| CTBP2 | ENST00000337195.5 | C-terminal binding protein 2 |
| DUSP3 | ENST00000226004.3 | dual specificity phosphatase 3 |
| WNT5A | ENST00000474267.1 | wingless-type MMTV integration site family, member 5A |
| C7orf73 | ENST00000507606.1 | chromosome 7 open reading frame 73 |
| RRM2B | ENST00000251810.3 | ribonucleotide reductase M2 B (TP53 inducible) |
| TXNDC5 | ENST00000539054.1 | thioredoxin domain containing 5 (endoplasmic reticulum) |
| APPBP2 | ENST00000083182.3 | amyloid beta precursor protein (cytoplasmic tail) binding protein 2 |
| BYSL | ENST00000230340.4 | bystin-like |
| AK4 | ENST00000545314.1 | adenylate kinase 4 |
| PDGFRB | ENST00000261799.4 | platelet-derived growth factor receptor, beta polypeptide |
| BRPF3 | ENST00000534400.1 | bromodomain and PHD finger containing, 3 |
| PCDH12 | ENST00000231484.3 | protocadherin 12 |
| PHF20L1 | ENST00000395386.2 | PHD finger protein 20-like 1 |
| NT5E | ENST00000369651.3 | 5'-nucleotidase, ecto (CD73) |
| BTBD6 | ENST00000392554.3 | BTB (POZ) domain containing 6 |
| GRIK4 | ENST00000527524.2 | glutamate receptor, ionotropic, kainate 4 |
| SENP5 | ENST00000323460.5 | SUMO1/sentrin specific peptidase 5 |
| PLEKHB2 | ENST00000438882.2 | pleckstrin homology domain containing, family B (evectins) member 2 |
| CBX2 | ENST00000310942.4 | chromobox homolog 2 |
| CTD-2054N24.2 | ENST00000559714.1 | Uncharacterized protein |
| SIRT1 | ENST00000212015.6 | sirtuin 1 |
| PDK1 | ENST00000282077.3 | pyruvate dehydrogenase kinase, isozyme 1 |
| OSMR | ENST00000274276.3 | oncostatin M receptor |
| HIST1H2BN | ENST00000606613.1 | histone cluster 1, H2bn |
| SLC9A7 | ENST00000328306.4 | solute carrier family 9, subfamily A (NHE7, cation proton antiporter 7), member 7 |
| AEBP2 | ENST00000266508.9 | AE binding protein 2 |
| RAB31 | ENST00000578921.1 | RAB31, member RAS oncogene family |
| GNA13 | ENST00000439174.2 | guanine nucleotide binding protein (G protein), alpha 13 |
| SERPIND1 | ENST00000215727.5 | serpin peptidase inhibitor, clade D (heparin cofactor), member 1 |
| DTX4 | ENST00000227451.3 | deltex homolog 4 (Drosophila) |
| E2F7 | ENST00000416496.2 | E2F transcription factor 7 |
| NYAP2 | ENST00000272907.6 | neuronal tyrosine-phosphorylated phosphoinositide-3-kinase adaptor 2 |
| KCNN1 | ENST00000222249.9 | potassium intermediate/small conductance calcium-activated channel, subfamily N, member 1 |
| L3MBTL2 | ENST00000216237.5 | l(3)mbt-like 2 (Drosophila) |
| MICALCL | ENST00000256186.2 | MICAL C-terminal like |
| KLHL8 | ENST00000273963.5 | kelch-like family member 8 |
| MGA | ENST00000219905.7 | MGA, MAX dimerization protein |
| PRKD1 | ENST00000331968.5 | protein kinase D1 |
| SSH1 | ENST00000360239.3 | slingshot protein phosphatase 1 |
| PRSS12 | ENST00000296498.3 | protease, serine, 12 (neurotrypsin, motopsin) |
| C12orf4 | ENST00000261250.3 | chromosome 12 open reading frame 4 |
| LRRC1 | ENST00000370888.1 | leucine rich repeat containing 1 |
| AC007461.1 | ENST00000602013.1 | Uncharacterized protein |
| UPF2 | ENST00000356352.2 | UPF2 regulator of nonsense transcripts homolog (yeast) |
| KIAA2018 | ENST00000316407.4 | KIAA2018 |
| RASD2 | ENST00000216127.4 | RASD family, member 2 |
| RRP12 | ENST00000370992.4 | ribosomal RNA processing 12 homolog (S. cerevisiae) |
| MTMR7 | ENST00000180173.5 | myotubularin related protein 7 |
| TP53I11 | ENST00000395648.3 | tumor protein p53 inducible protein 11 |
| RAI1 | ENST00000353383.1 | retinoic acid induced 1 |
| FMO2 | ENST00000441535.1 | flavin containing monooxygenase 2 (non-functional) |
| FLRT2 | ENST00000330753.4 | fibronectin leucine rich transmembrane protein 2 |
| SRSF10 | ENST00000343255.5 | serine/arginine-rich splicing factor 10 |
| SMIM13 | ENST00000416247.2 | small integral membrane protein 13 |
| WDR12 | ENST00000261015.4 | WD repeat domain 12 |
| TFAP2B | ENST00000393655.3 | transcription factor AP-2 beta (activating enhancer binding protein 2 beta) |
| NCKAP1 | ENST00000361354.4 | NCK-associated protein 1 |
| HMGB1 | ENST00000399489.1 | high mobility group box 1 |
| ZNF793 | ENST00000445217.1 | zinc finger protein 793 |
| TMEM132B | ENST00000299308.3 | transmembrane protein 132B |
| OLFML1 | ENST00000329293.3 | olfactomedin-like 1 |
| VNN1 | ENST00000367928.4 | vanin 1 |
| KIAA1429 | ENST00000437199.1 | KIAA1429 |
| UBQLNL | ENST00000380184.1 | ubiquilin-like |
| SLC6A14 | ENST00000371900.4 | solute carrier family 6 (amino acid transporter), member 14 |
| TMEM38A | ENST00000187762.2 | transmembrane protein 38A |
| ZNF81 | ENST00000338637.7 | zinc finger protein 81 |
| PIK3IP1 | ENST00000441972.1 | phosphoinositide-3-kinase interacting protein 1 |
| KLLN | ENST00000445946.3 | killin, p53-regulated DNA replication inhibitor |
| EMCN | ENST00000296420.4 | endomucin |
| XRCC2 | ENST00000359321.1 | X-ray repair complementing defective repair in Chinese hamster cells 2 |
| C6orf89 | ENST00000480824.2 | chromosome 6 open reading frame 89 |
| FAM172A | ENST00000395965.3 | family with sequence similarity 172, member A |
| MAF | ENST00000393350.1 | v-maf avian musculoaponeurotic fibrosarcoma oncogene homolog |
| POGK | ENST00000367875.1 | pogo transposable element with KRAB domain |
| GABRA2 | ENST00000507460.1 | gamma-aminobutyric acid (GABA) A receptor, alpha 2 |
| LRRC58 | ENST00000295628.3 | leucine rich repeat containing 58 |
| RWDD2A | ENST00000369724.4 | RWD domain containing 2A |
| PDGFD | ENST00000393158.2 | platelet derived growth factor D |
| CARM1 | ENST00000327064.4 | coactivator-associated arginine methyltransferase 1 |
| SLIT3 | ENST00000519560.1 | slit homolog 3 (Drosophila) |
| ZDHHC2 | ENST00000262096.8 | zinc finger, DHHC-type containing 2 |
| MRPL50 | ENST00000374865.4 | mitochondrial ribosomal protein L50 |
| PROX1 | ENST00000366958.4 | prospero homeobox 1 |
| SPATA17 | ENST00000366933.4 | spermatogenesis associated 17 |
| ZC3H10 | ENST00000257940.2 | zinc finger CCCH-type containing 10 |
| TMEM26 | ENST00000399298.3 | transmembrane protein 26 |
| MTIF2 | ENST00000403721.1 | mitochondrial translational initiation factor 2 |
| EAF1 | ENST00000396842.2 | ELL associated factor 1 |
| RDX | ENST00000343115.4 | radixin |
| TUFT1 | ENST00000368849.3 | tuftelin 1 |
| SLC7A14 | ENST00000231706.5 | solute carrier family 7, member 14 |
| SLC38A11 | ENST00000303735.4 | solute carrier family 38, member 11 |
| KIAA1244 | ENST00000251691.4 | KIAA1244 |
| ZNF510 | ENST00000375231.1 | zinc finger protein 510 |
| ABHD5 | ENST00000458276.2 | abhydrolase domain containing 5 |
| TNRC6C | ENST00000335749.4 | trinucleotide repeat containing 6C |
| AFAP1L1 | ENST00000296721.4 | actin filament associated protein 1-like 1 |
| MPP3 | ENST00000398393.1 | membrane protein, palmitoylated 3 (MAGUK p55 subfamily member 3) |
| CDC27 | ENST00000066544.3 | cell division cycle 27 |
| MGAT4A | ENST00000264968.3 | mannosyl (alpha-1,3-)-glycoprotein beta-1,4-N-acetylglucosaminyltransferase, isozyme A |
| CALM3 | ENST00000291295.9 | calmodulin 3 (phosphorylase kinase, delta) |
| FAXC | ENST00000389677.5 | failed axon connections homolog (Drosophila) |
| RIF1 | ENST00000243326.5 | RAP1 interacting factor homolog (yeast) |
| PCDH10 | ENST00000264360.5 | protocadherin 10 |
| ZFHX3 | ENST00000268489.5 | zinc finger homeobox 3 |
| CD300LD | ENST00000375352.1 | CD300 molecule-like family member d |
| ABCA12 | ENST00000272895.7 | ATP-binding cassette, sub-family A (ABC1), member 12 |
| ZNF99 | ENST00000596209.1 | zinc finger protein 99 |
| ASAP2 | ENST00000315273.4 | ArfGAP with SH3 domain, ankyrin repeat and PH domain 2 |
| NUDCD2 | ENST00000302764.4 | NudC domain containing 2 |
| C1orf116 | ENST00000359470.5 | chromosome 1 open reading frame 116 |
| SOSTDC1 | ENST00000307068.4 | sclerostin domain containing 1 |
| CALCRL | ENST00000392370.3 | calcitonin receptor-like |
| PSPC1 | ENST00000338910.4 | paraspeckle component 1 |
| STEAP2 | ENST00000287908.3 | STEAP family member 2, metalloreductase |
| ZDHHC3 | ENST00000296127.3 | zinc finger, DHHC-type containing 3 |
| ANKRD13C | ENST00000370944.4 | ankyrin repeat domain 13C |
| ZNF704 | ENST00000327835.3 | zinc finger protein 704 |
| KCNJ13 | ENST00000409779.1 | potassium inwardly-rectifying channel, subfamily J, member 13 |
| DCTN5 | ENST00000300087.2 | dynactin 5 (p25) |
| STK17A | ENST00000319357.5 | serine/threonine kinase 17a |
| ZNF821 | ENST00000564134.1 | zinc finger protein 821 |
| LRP2BP | ENST00000362004.3 | LRP2 binding protein |
| ARHGAP32 | ENST00000524655.1 | Rho GTPase activating protein 32 |
| ZNF865 | ENST00000568956.1 | zinc finger protein 865 |
| GALNT4 | ENST00000529983.2 | UDP-N-acetyl-alpha-D-galactosamine:polypeptide N-acetylgalactosaminyltransferase 4 (GalNAc-T4) |
| SELE | ENST00000367781.4 | selectin E |
| SETD3 | ENST00000331768.5 | SET domain containing 3 |
| HSPA4L | ENST00000296464.4 | heat shock 70kDa protein 4-like |
| NKAP | ENST00000371410.3 | NFKB activating protein |
| ABLIM1 | ENST00000392952.3 | actin binding LIM protein 1 |
| ZNF585A | ENST00000356958.4 | zinc finger protein 585A |
| C9orf9 | ENST00000372136.3 | chromosome 9 open reading frame 9 |
| UCHL5 | ENST00000367455.4 | ubiquitin carboxyl-terminal hydrolase L5 |
| RAB7L1 | ENST00000367139.3 | RAB7, member RAS oncogene family-like 1 |
| MTR | ENST00000366577.5 | 5-methyltetrahydrofolate-homocysteine methyltransferase |
| SLC9A2 | ENST00000233969.2 | solute carrier family 9, subfamily A (NHE2, cation proton antiporter 2), member 2 |
| KCNJ2 | ENST00000243457.3 | potassium inwardly-rectifying channel, subfamily J, member 2 |
| IL6R | ENST00000344086.4 | interleukin 6 receptor |
| ZNF106 | ENST00000263805.4 | zinc finger protein 106 |
| PCP4L1 | ENST00000504449.1 | Purkinje cell protein 4 like 1 |
| PPP4R4 | ENST00000328839.3 | protein phosphatase 4, regulatory subunit 4 |
| LIPT2 | ENST00000310109.4 | lipoyl(octanoyl) transferase 2 (putative) |
| CAPN5 | ENST00000531028.1 | calpain 5 |
| CHST6 | ENST00000332272.4 | carbohydrate (N-acetylglucosamine 6-O) sulfotransferase 6 |
| PTRF | ENST00000357037.5 | polymerase I and transcript release factor |
| CLDN11 | ENST00000064724.3 | claudin 11 |
| ZNF43 | ENST00000594012.1 | zinc finger protein 43 |
| SPTLC1 | ENST00000262554.2 | serine palmitoyltransferase, long chain base subunit 1 |
| C11orf82 | ENST00000528759.1 | chromosome 11 open reading frame 82 |
| UBXN2B | ENST00000399598.2 | UBX domain protein 2B |
| MCM9 | ENST00000316316.6 | minichromosome maintenance complex component 9 |
| JAZF1 | ENST00000283928.5 | JAZF zinc finger 1 |
| FAM9C | ENST00000542843.1 | family with sequence similarity 9, member C |
| EPPIN | ENST00000555685.1 | epididymal peptidase inhibitor |
| EPPIN-WFDC6 | ENST00000504988.1 | EPPIN-WFDC6 readthrough |
| CABLES2 | ENST00000279101.5 | Cdk5 and Abl enzyme substrate 2 |
| KIF1C | ENST00000320785.5 | kinesin family member 1C |
| SSPN | ENST00000242729.2 | sarcospan |
| ARSE | ENST00000540563.1 | arylsulfatase E (chondrodysplasia punctata 1) |
| TUBB1 | ENST00000217133.1 | tubulin, beta 1 class VI |
| KIAA1143 | ENST00000296121.4 | KIAA1143 |
| CELF5 | ENST00000541430.2 | CUGBP, Elav-like family member 5 |
| SLC1A2 | ENST00000278379.3 | solute carrier family 1 (glial high affinity glutamate transporter), member 2 |
| C7orf65 | ENST00000408988.2 | chromosome 7 open reading frame 65 |
| CSRNP3 | ENST00000314499.7 | cysteine-serine-rich nuclear protein 3 |
| PTPN6 | ENST00000456013.1 | protein tyrosine phosphatase, non-receptor type 6 |
| CBLN2 | ENST00000585159.1 | cerebellin 2 precursor |
| PTPRK | ENST00000368226.4 | protein tyrosine phosphatase, receptor type, K |
| DPY19L3 | ENST00000586987.1 | dpy-19-like 3 (C. elegans) |
| ZDHHC9 | ENST00000357166.6 | zinc finger, DHHC-type containing 9 |
| KMT2D | ENST00000301067.7 | lysine (K)-specific methyltransferase 2D |
| ERMP1 | ENST00000381506.3 | endoplasmic reticulum metallopeptidase 1 |
| IQGAP1 | ENST00000268182.5 | IQ motif containing GTPase activating protein 1 |
| PDE3A | ENST00000359062.3 | phosphodiesterase 3A, cGMP-inhibited |
| SLC47A1 | ENST00000270570.4 | solute carrier family 47 (multidrug and toxin extrusion), member 1 |
| RNASEH1 | ENST00000315212.3 | ribonuclease H1 |
| SOX6 | ENST00000316399.6 | SRY (sex determining region Y)-box 6 |
| KDM6A | ENST00000377967.4 | lysine (K)-specific demethylase 6A |
| MOB1A | ENST00000396049.4 | MOB kinase activator 1A |
| PKIA | ENST00000396418.2 | protein kinase (cAMP-dependent, catalytic) inhibitor alpha |
| PARP8 | ENST00000503750.2 | poly (ADP-ribose) polymerase family, member 8 |
| LEPREL4 | ENST00000355468.3 | leprecan-like 4 |
| KCNQ5 | ENST00000370398.1 | potassium voltage-gated channel, KQT-like subfamily, member 5 |
| FBLIM1 | ENST00000375771.1 | filamin binding LIM protein 1 |
| VPS13C | ENST00000249837.3 | vacuolar protein sorting 13 homolog C (S. cerevisiae) |
| ZNF525 | ENST00000467003.1 | zinc finger protein 525 |
| FGF14 | ENST00000376131.4 | fibroblast growth factor 14 |
| NRK | ENST00000243300.9 | Nik related kinase |
| USP6 | ENST00000332776.4 | ubiquitin specific peptidase 6 (Tre-2 oncogene) |
| ZNF148 | ENST00000360647.4 | zinc finger protein 148 |
| GPRIN3 | ENST00000609438.1 | GPRIN family member 3 |
| ZKSCAN1 | ENST00000324306.6 | zinc finger with KRAB and SCAN domains 1 |
| TMEM56 | ENST00000370203.4 | transmembrane protein 56 |
| FAM120C | ENST00000477084.1 | family with sequence similarity 120C |
| GINM1 | ENST00000367419.5 | glycoprotein integral membrane 1 |
| ZNF317 | ENST00000247956.6 | zinc finger protein 317 |
| FAM110C | ENST00000327669.4 | family with sequence similarity 110, member C |
| BIRC3 | ENST00000532808.1 | baculoviral IAP repeat containing 3 |
| XYLT2 | ENST00000017003.2 | xylosyltransferase II |
| ASTN1 | ENST00000361833.2 | astrotactin 1 |
| FASTK | ENST00000540185.1 | Fas-activated serine/threonine kinase |
| SYAP1 | ENST00000380155.3 | synapse associated protein 1 |
| ZNF202 | ENST00000336139.4 | zinc finger protein 202 |
| KRT86 | ENST00000293525.5 | keratin 86 |
| TLDC1 | ENST00000343629.6 | TBC/LysM-associated domain containing 1 |
| KLF3 | ENST00000261438.5 | Kruppel-like factor 3 (basic) |
| GNB4 | ENST00000232564.3 | guanine nucleotide binding protein (G protein), beta polypeptide 4 |
| RRP7A | ENST00000323013.6 | ribosomal RNA processing 7 homolog A (S. cerevisiae) |
| SGSM1 | ENST00000400358.4 | small G protein signaling modulator 1 |
| TRIP13 | ENST00000166345.3 | thyroid hormone receptor interactor 13 |
| HCCS | ENST00000321143.4 | holocytochrome c synthase |
| ARPP19 | ENST00000566423.1 | cAMP-regulated phosphoprotein, 19kDa |
| ADH1B | ENST00000305046.8 | alcohol dehydrogenase 1B (class I), beta polypeptide |
| SLC30A2 | ENST00000374278.3 | solute carrier family 30 (zinc transporter), member 2 |
| PTPRT | ENST00000373187.1 | protein tyrosine phosphatase, receptor type, T |
| ZBTB26 | ENST00000373656.3 | zinc finger and BTB domain containing 26 |
| RNF150 | ENST00000515673.2 | ring finger protein 150 |
| CP | ENST00000264613.6 | ceruloplasmin (ferroxidase) |
| NPHP3 | ENST00000326682.8 | nephronophthisis 3 (adolescent) |
| DYNLL2 | ENST00000579991.2 | dynein, light chain, LC8-type 2 |
| XPO1 | ENST00000401558.2 | exportin 1 (CRM1 homolog, yeast) |
| FAM20B | ENST00000263733.4 | family with sequence similarity 20, member B |
| TP53RK | ENST00000372102.3 | TP53 regulating kinase |
| DPH3 | ENST00000488423.1 | diphthamide biosynthesis 3 |
| KLHL42 | ENST00000381271.2 | kelch-like family member 42 |
| DCP2 | ENST00000389063.2 | decapping mRNA 2 |
| TNPO3 | ENST00000393245.1 | transportin 3 |
| FAM210B | ENST00000371384.3 | family with sequence similarity 210, member B |
| TAS2R4 | ENST00000247881.2 | taste receptor, type 2, member 4 |
| XPO5 | ENST00000265351.7 | exportin 5 |
| CLVS2 | ENST00000275162.5 | clavesin 2 |
| KITLG | ENST00000228280.5 | KIT ligand |
| AASS | ENST00000393376.1 | aminoadipate-semialdehyde synthase |
| KLF11 | ENST00000305883.1 | Kruppel-like factor 11 |
| MYO9B | ENST00000595618.1 | myosin IXB |
| GPLD1 | ENST00000230036.1 | glycosylphosphatidylinositol specific phospholipase D1 |
| SERPINH1 | ENST00000533603.1 | serpin peptidase inhibitor, clade H (heat shock protein 47), member 1, (collagen binding protein 1) |
| SH2D4B | ENST00000339284.2 | SH2 domain containing 4B |
| CDC40 | ENST00000368932.1 | cell division cycle 40 |
| CXorf36 | ENST00000398000.2 | chromosome X open reading frame 36 |
| PER2 | ENST00000254658.3 | period circadian clock 2 |
| HS3ST1 | ENST00000002596.5 | heparan sulfate (glucosamine) 3-O-sulfotransferase 1 |
| C21orf91 | ENST00000400558.3 | chromosome 21 open reading frame 91 |
| C6orf132 | ENST00000341865.4 | chromosome 6 open reading frame 132 |
| RSF1 | ENST00000308488.6 | remodeling and spacing factor 1 |
| TRAPPC2P1 | ENST00000543226.1 | trafficking protein particle complex 2 pseudogene 1 |
| PGBD4 | ENST00000397766.2 | piggyBac transposable element derived 4 |
| CSNK1G3 | ENST00000360683.2 | casein kinase 1, gamma 3 |
| ARHGEF38 | ENST00000420470.2 | Rho guanine nucleotide exchange factor (GEF) 38 |
| ZNF445 | ENST00000425708.2 | zinc finger protein 445 |
| PCNXL2 | ENST00000258229.9 | pecanex-like 2 (Drosophila) |
| ESR1 | ENST00000440973.1 | estrogen receptor 1 |
| C5orf15 | ENST00000231512.3 | chromosome 5 open reading frame 15 |
| KIAA0319 | ENST00000537886.1 | KIAA0319 |
| GID8 | ENST00000266069.3 | GID complex subunit 8 |
| SLC5A3 | ENST00000608209.1 | sodium/myo-inositol cotransporter |
| HGFAC | ENST00000382774.3 | HGF activator |
| SUV39H2 | ENST00000313519.5 | suppressor of variegation 3-9 homolog 2 (Drosophila) |
| CA12 | ENST00000178638.3 | carbonic anhydrase XII |
| DSC3 | ENST00000434452.1 | desmocollin 3 |
| SEL1L | ENST00000336735.4 | sel-1 suppressor of lin-12-like (C. elegans) |
| RBM12B | ENST00000399300.2 | RNA binding motif protein 12B |
| TEX22 | ENST00000451127.2 | testis expressed 22 |
| ATOH8 | ENST00000306279.3 | atonal homolog 8 (Drosophila) |
| TMED8 | ENST00000216468.7 | transmembrane emp24 protein transport domain containing 8 |
| PURA | ENST00000331327.3 | purine-rich element binding protein A |
| GRM5 | ENST00000418177.2 | glutamate receptor, metabotropic 5 |
| MEF2A | ENST00000354410.5 | myocyte enhancer factor 2A |
| GOLGA6L10 | ENST00000439287.4 | Putative golgin subfamily A member 6-like protein 10 |
| MMP16 | ENST00000286614.6 | matrix metallopeptidase 16 (membrane-inserted) |
| CD28 | ENST00000324106.8 | CD28 molecule |
| ZNF766 | ENST00000439461.1 | zinc finger protein 766 |
| PLA2R1 | ENST00000283243.7 | phospholipase A2 receptor 1, 180kDa |
| VPS35 | ENST00000299138.7 | vacuolar protein sorting 35 homolog (S. cerevisiae) |
| TBC1D9B | ENST00000519746.1 | TBC1 domain family, member 9B (with GRAM domain) |
| SMCR8 | ENST00000406438.3 | Smith-Magenis syndrome chromosome region, candidate 8 |
| SLC9A8 | ENST00000361573.2 | solute carrier family 9, subfamily A (NHE8, cation proton antiporter 8), member 8 |
| B3GALTL | ENST00000343307.4 | beta 1,3-galactosyltransferase-like |
| FRRS1 | ENST00000287474.5 | ferric-chelate reductase 1 |
| MAPK1IP1L | ENST00000395468.4 | mitogen-activated protein kinase 1 interacting protein 1-like |
| PTGER2 | ENST00000245457.5 | prostaglandin E receptor 2 (subtype EP2), 53kDa |
| COG6 | ENST00000455146.3 | component of oligomeric golgi complex 6 |
| CCDC134 | ENST00000255784.5 | coiled-coil domain containing 134 |
| PTBP3 | ENST00000374257.1 | polypyrimidine tract binding protein 3 |
| TMEM11 | ENST00000317635.5 | transmembrane protein 11 |
| CACNG8 | ENST00000270458.2 | calcium channel, voltage-dependent, gamma subunit 8 |
| IRAK2 | ENST00000256458.4 | interleukin-1 receptor-associated kinase 2 |
| SORD | ENST00000267814.9 | sorbitol dehydrogenase |
| CMKLR1 | ENST00000312143.7 | chemokine-like receptor 1 |
| ETV1 | ENST00000430479.1 | ets variant 1 |
| SPG20 | ENST00000438666.2 | spastic paraplegia 20 (Troyer syndrome) |
| CCNG2 | ENST00000316355.5 | cyclin G2 |
| EIF2S3 | ENST00000253039.4 | eukaryotic translation initiation factor 2, subunit 3 gamma, 52kDa |
| LRRC8C | ENST00000370454.4 | leucine rich repeat containing 8 family, member C |
| CBLB | ENST00000264122.4 | Cbl proto-oncogene B, E3 ubiquitin protein ligase |
| DIO2 | ENST00000438257.4 | deiodinase, iodothyronine, type II |
| TMEM252 | ENST00000377311.3 | transmembrane protein 252 |
| MTF1 | ENST00000373036.4 | metal-regulatory transcription factor 1 |
| TFDP2 | ENST00000499676.2 | transcription factor Dp-2 (E2F dimerization partner 2) |
| RNF180 | ENST00000389100.4 | ring finger protein 180 |
| AL031666.2 | ENST00000599904.1 | HCG2018772; Uncharacterized protein; cDNA FLJ31609 fis, clone NT2RI2002852 |
| EXOC6B | ENST00000272427.6 | exocyst complex component 6B |
| TRIM2 | ENST00000338700.5 | tripartite motif containing 2 |
| SKA2 | ENST00000580541.1 | spindle and kinetochore associated complex subunit 2 |
| GRIN3B | ENST00000234389.3 | glutamate receptor, ionotropic, N-methyl-D-aspartate 3B |
| UBFD1 | ENST00000395878.3 | ubiquitin family domain containing 1 |
| ZNF175 | ENST00000262259.2 | zinc finger protein 175 |
| KDM2B | ENST00000536437.1 | lysine (K)-specific demethylase 2B |
| VGLL3 | ENST00000398399.2 | vestigial like 3 (Drosophila) |
| EGFR | ENST00000275493.2 | epidermal growth factor receptor |
| ZC3H8 | ENST00000409573.2 | zinc finger CCCH-type containing 8 |
| MESDC1 | ENST00000267984.2 | mesoderm development candidate 1 |
| EMX1 | ENST00000258106.6 | empty spiracles homeobox 1 |
| SLC4A4 | ENST00000340595.3 | solute carrier family 4 (sodium bicarbonate cotransporter), member 4 |
| CALML4 | ENST00000395465.3 | calmodulin-like 4 |
| SLC7A2 | ENST00000494857.1 | solute carrier family 7 (cationic amino acid transporter, y+ system), member 2 |
| IFRD1 | ENST00000403825.3 | interferon-related developmental regulator 1 |
| USP38 | ENST00000307017.4 | ubiquitin specific peptidase 38 |
| PADI1 | ENST00000375471.4 | peptidyl arginine deiminase, type I |
| DLGAP2 | ENST00000421627.2 | discs, large (Drosophila) homolog-associated protein 2 |
| CLDN12 | ENST00000287916.4 | claudin 12 |
| TINF2 | ENST00000558566.1 | TERF1 (TRF1)-interacting nuclear factor 2 |
| GTDC1 | ENST00000392869.2 | glycosyltransferase-like domain containing 1 |
| ESCO2 | ENST00000305188.8 | establishment of sister chromatid cohesion N-acetyltransferase 2 |
| TMEM194A | ENST00000379391.3 | transmembrane protein 194A |
| LSM14A | ENST00000433627.5 | LSM14A, SCD6 homolog A (S. cerevisiae) |
| PARVA | ENST00000334956.8 | parvin, alpha |
| SPPL2A | ENST00000261854.5 | signal peptide peptidase like 2A |
| STON1 | ENST00000309835.3 | stonin 1 |
| PCDHA4 | ENST00000530339.1 | protocadherin alpha 4 |
| PCDHA5 | ENST00000529859.1 | protocadherin alpha 5 |
| PCDHA7 | ENST00000525929.1 | protocadherin alpha 7 |
| TRPS1 | ENST00000395715.3 | trichorhinophalangeal syndrome I |
| KCNE4 | ENST00000281830.3 | potassium voltage-gated channel, Isk-related family, member 4 |
| RP13-996F3.5 | ENST00000559949.1 | Uncharacterized protein |
| RC3H1 | ENST00000367696.2 | ring finger and CCCH-type domains 1 |
| ANKS1A | ENST00000360359.3 | ankyrin repeat and sterile alpha motif domain containing 1A |
| CBL | ENST00000264033.4 | Cbl proto-oncogene, E3 ubiquitin protein ligase |
| PCDHA12 | ENST00000398631.2 | protocadherin alpha 12 |
| PCDHA10 | ENST00000307360.5 | protocadherin alpha 10 |
| CCSER2 | ENST00000224756.8 | coiled-coil serine-rich protein 2 |
| PCDHA2 | ENST00000526136.1 | protocadherin alpha 2 |
| SLC8A1 | ENST00000406785.2 | solute carrier family 8 (sodium/calcium exchanger), member 1 |
| BARD1 | ENST00000260947.4 | BRCA1 associated RING domain 1 |
| NUDT12 | ENST00000230792.2 | nudix (nucleoside diphosphate linked moiety X)-type motif 12 |
| RGS12 | ENST00000538395.1 | regulator of G-protein signaling 12 |
| SGCD | ENST00000435422.3 | sarcoglycan, delta (35kDa dystrophin-associated glycoprotein) |
| NCAM2 | ENST00000400546.1 | neural cell adhesion molecule 2 |
| MOB3B | ENST00000262244.5 | MOB kinase activator 3B |
| PCDHA1 | ENST00000504120.2 | protocadherin alpha 1 |
| PCDHA13 | ENST00000289272.2 | protocadherin alpha 13 |
| PCDHA11 | ENST00000398640.2 | protocadherin alpha 11 |
| AL161915.1 | ENST00000391366.1 | Uncharacterized protein |
| STRN3 | ENST00000355683.5 | striatin, calmodulin binding protein 3 |
| PCDHA3 | ENST00000522353.2 | protocadherin alpha 3 |
| GOLGA6L4 | ENST00000510439.2 | golgin A6 family-like 4 |
| GPA33 | ENST00000367868.3 | glycoprotein A33 (transmembrane) |
| PKM | ENST00000319622.6 | pyruvate kinase, muscle |
| ZNF770 | ENST00000356321.4 | zinc finger protein 770 |
| PCDHA9 | ENST00000532602.1 | protocadherin alpha 9 |
| PCDHA6 | ENST00000529310.1 | protocadherin alpha 6 |
| SCN3A | ENST00000360093.3 | sodium channel, voltage-gated, type III, alpha subunit |
| PCDHAC1 | ENST00000253807.2 | protocadherin alpha subfamily C, 1 |
| PCDHA8 | ENST00000531613.1 | protocadherin alpha 8 |
| YWHAZ | ENST00000395957.2 | tyrosine 3-monooxygenase/tryptophan 5-monooxygenase activation protein, zeta polypeptide |
| ZNF208 | ENST00000397126.4 | zinc finger protein 208 |
| PTPRG | ENST00000474889.1 | protein tyrosine phosphatase, receptor type, G |
| LANCL3 | ENST00000378621.3 | LanC lantibiotic synthetase component C-like 3 (bacterial) |
| GALNT10 | ENST00000297107.6 | UDP-N-acetyl-alpha-D-galactosamine:polypeptide N-acetylgalactosaminyltransferase 10 (GalNAc-T10) |
| RABL3 | ENST00000273375.3 | RAB, member of RAS oncogene family-like 3 |
| NUDT16 | ENST00000359850.3 | nudix (nucleoside diphosphate linked moiety X)-type motif 16 |
| RHBDD1 | ENST00000341329.3 | rhomboid domain containing 1 |
| WIZ | ENST00000389282.4 | widely interspaced zinc finger motifs |
| TIMP4 | ENST00000287814.4 | TIMP metallopeptidase inhibitor 4 |
| C4orf46 | ENST00000508457.1 | chromosome 4 open reading frame 46 |
| SEC16A | ENST00000313050.7 | SEC16 homolog A (S. cerevisiae) |
| INPP4B | ENST00000513000.1 | inositol polyphosphate-4-phosphatase, type II, 105kDa |
| FCRL5 | ENST00000361835.3 | Fc receptor-like 5 |
| NSG1 | ENST00000421177.2 | Neuron-specific protein family member 1 |
| FNBP1L | ENST00000370253.2 | formin binding protein 1-like |
| RGAG4 | ENST00000545866.1 | retrotransposon gag domain containing 4 |
| ILDR1 | ENST00000273691.3 | immunoglobulin-like domain containing receptor 1 |
| PPARA | ENST00000396000.2 | peroxisome proliferator-activated receptor alpha |
| ANKRD34C | ENST00000421388.2 | ankyrin repeat domain 34C |
| RPL10A | ENST00000322203.6 | ribosomal protein L10a |
| GFPT1 | ENST00000357308.4 | glutamine--fructose-6-phosphate transaminase 1 |
| NFIB | ENST00000397575.3 | nuclear factor I/B |
| NAT10 | ENST00000257829.3 | N-acetyltransferase 10 (GCN5-related) |
| NCAPD2 | ENST00000315579.5 | non-SMC condensin I complex, subunit D2 |
| MLEC | ENST00000228506.3 | malectin |
| ELK3 | ENST00000228741.3 | ELK3, ETS-domain protein (SRF accessory protein 2) |
| METTL20 | ENST00000357721.3 | methyltransferase like 20 |
| ADAL | ENST00000428046.3 | adenosine deaminase-like |
| SLC30A7 | ENST00000370112.4 | solute carrier family 30 (zinc transporter), member 7 |
| SCEL | ENST00000377246.3 | sciellin |
| SLFN12 | ENST00000394562.1 | schlafen family member 12 |
| LAX1 | ENST00000442561.2 | lymphocyte transmembrane adaptor 1 |
| CLDN19 | ENST00000296387.1 | claudin 19 |
| GSTK1 | ENST00000358406.5 | glutathione S-transferase kappa 1 |
| PAX6 | ENST00000419022.1 | paired box 6 |
| KIF11 | ENST00000260731.3 | kinesin family member 11 |
| PDP1 | ENST00000396200.3 | pyruvate dehyrogenase phosphatase catalytic subunit 1 |
| PCDHB11 | ENST00000354757.3 | protocadherin beta 11 |
| SCN2B | ENST00000278947.5 | sodium channel, voltage-gated, type II, beta subunit |
| PCDHAC2 | ENST00000289269.5 | protocadherin alpha subfamily C, 2 |
| ALPK3 | ENST00000258888.5 | alpha-kinase 3 |
| XYLT1 | ENST00000261381.6 | xylosyltransferase I |
| GAREM | ENST00000399218.4 | GRB2 associated, regulator of MAPK1 |
| ABAT | ENST00000569156.1 | 4-aminobutyrate aminotransferase |
| MKLN1 | ENST00000352689.6 | muskelin 1, intracellular mediator containing kelch motifs |
| SMAGP | ENST00000603798.1 | small cell adhesion glycoprotein |
| RGS5 | ENST00000313961.5 | regulator of G-protein signaling 5 |
| DCTN4 | ENST00000447998.2 | dynactin 4 (p62) |
| NUDT3 | ENST00000607016.1 | nudix (nucleoside diphosphate linked moiety X)-type motif 3 |
| FNBP1 | ENST00000355681.3 | formin binding protein 1 |
| EFCAB2 | ENST00000366523.1 | EF-hand calcium binding domain 2 |
| GDF11 | ENST00000257868.5 | growth differentiation factor 11 |
| TMOD1 | ENST00000259365.4 | tropomodulin 1 |
| PAN3 | ENST00000282391.5 | PAN3 poly(A) specific ribonuclease subunit homolog (S. cerevisiae) |
| GDAP1L1 | ENST00000342560.5 | ganglioside induced differentiation associated protein 1-like 1 |
| ADAM11 | ENST00000200557.6 | ADAM metallopeptidase domain 11 |
| FAM63B | ENST00000559228.1 | family with sequence similarity 63, member B |
| IKZF3 | ENST00000346872.3 | IKAROS family zinc finger 3 (Aiolos) |
| NRCAM | ENST00000379028.3 | neuronal cell adhesion molecule |
| ITPRIP | ENST00000337478.1 | inositol 1,4,5-trisphosphate receptor interacting protein |
| CNGA3 | ENST00000272602.2 | cyclic nucleotide gated channel alpha 3 |
| SEPT6 | ENST00000394610.1 | septin 6 |
| GABBR2 | ENST00000259455.2 | gamma-aminobutyric acid (GABA) B receptor, 2 |
| PTPRE | ENST00000254667.3 | protein tyrosine phosphatase, receptor type, E |
| DCAF12 | ENST00000361264.4 | DDB1 and CUL4 associated factor 12 |
| SSH2 | ENST00000582084.1 | slingshot protein phosphatase 2 |
| CARHSP1 | ENST00000396593.2 | calcium regulated heat stable protein 1, 24kDa |
| VPS13A | ENST00000360280.3 | vacuolar protein sorting 13 homolog A (S. cerevisiae) |
| ZNF799 | ENST00000419318.1 | zinc finger protein 799 |
| C16orf52 | ENST00000542527.2 | chromosome 16 open reading frame 52 |
| MTMR2 | ENST00000346299.5 | myotubularin related protein 2 |
| ADAM12 | ENST00000368679.4 | ADAM metallopeptidase domain 12 |
| LONRF3 | ENST00000304778.7 | LON peptidase N-terminal domain and ring finger 3 |
| METTL12 | ENST00000532971.1 | methyltransferase like 12 |
| PREX2 | ENST00000288368.4 | phosphatidylinositol-3,4,5-trisphosphate-dependent Rac exchange factor 2 |
| TTC9 | ENST00000256367.2 | tetratricopeptide repeat domain 9 |
| ABL1 | ENST00000318560.5 | c-abl oncogene 1, non-receptor tyrosine kinase |
| BMP2 | ENST00000378827.4 | bone morphogenetic protein 2 |
| NUDT15 | ENST00000258662.2 | nudix (nucleoside diphosphate linked moiety X)-type motif 15 |
| POU2F2 | ENST00000342301.4 | POU class 2 homeobox 2 |
| EOMES | ENST00000295743.4 | eomesodermin |
| LSAMP | ENST00000490035.2 | limbic system-associated membrane protein |
| RILPL1 | ENST00000376874.4 | Rab interacting lysosomal protein-like 1 |
| IVNS1ABP | ENST00000367498.3 | influenza virus NS1A binding protein |
| CRBN | ENST00000231948.4 | cereblon |
| TNFRSF19 | ENST00000382263.3 | tumor necrosis factor receptor superfamily, member 19 |
| SEPHS1 | ENST00000545675.1 | selenophosphate synthetase 1 |
| CREG2 | ENST00000324768.5 | cellular repressor of E1A-stimulated genes 2 |
| ID4 | ENST00000378700.3 | inhibitor of DNA binding 4, dominant negative helix-loop-helix protein |
| WIPF1 | ENST00000392547.2 | WAS/WASL interacting protein family, member 1 |
| GMPS | ENST00000496455.2 | guanine monphosphate synthase |
| LGALS8 | ENST00000526589.1 | lectin, galactoside-binding, soluble, 8 |
| ZNF268 | ENST00000542986.2 | zinc finger protein 268 |
| NEURL1B | ENST00000369800.5 | neuralized homolog 1B (Drosophila) |
| ZNF76 | ENST00000373953.3 | zinc finger protein 76 |
| ADPRH | ENST00000478399.1 | ADP-ribosylarginine hydrolase |
| AIM1 | ENST00000369066.3 | absent in melanoma 1 |
| MTMR3 | ENST00000333027.3 | myotubularin related protein 3 |
| C17orf107 | ENST00000381365.3 | chromosome 17 open reading frame 107 |
| HIP1 | ENST00000336926.6 | huntingtin interacting protein 1 |
| LIMD1 | ENST00000273317.4 | LIM domains containing 1 |
| WDR70 | ENST00000265107.4 | WD repeat domain 70 |
| OXTR | ENST00000316793.3 | oxytocin receptor |
| ARL5B | ENST00000377275.3 | ADP-ribosylation factor-like 5B |
| SAP30L | ENST00000297109.6 | SAP30-like |
| PTPN14 | ENST00000366956.5 | protein tyrosine phosphatase, non-receptor type 14 |
| KPNA6 | ENST00000373625.3 | karyopherin alpha 6 (importin alpha 7) |
| MFSD9 | ENST00000258436.5 | major facilitator superfamily domain containing 9 |
| ZNF211 | ENST00000347302.3 | zinc finger protein 211 |
| TBC1D1 | ENST00000261439.4 | TBC1 (tre-2/USP6, BUB2, cdc16) domain family, member 1 |
| ORAI2 | ENST00000356387.2 | ORAI calcium release-activated calcium modulator 2 |
| USP22 | ENST00000261497.4 | ubiquitin specific peptidase 22 |
| TMEM106B | ENST00000396667.3 | transmembrane protein 106B |
| STRN | ENST00000263918.4 | striatin, calmodulin binding protein |
| PAPPA | ENST00000328252.3 | pregnancy-associated plasma protein A, pappalysin 1 |
| PGM2L1 | ENST00000298198.4 | phosphoglucomutase 2-like 1 |
| VKORC1L1 | ENST00000360768.3 | vitamin K epoxide reductase complex, subunit 1-like 1 |
| JAKMIP2 | ENST00000507386.1 | janus kinase and microtubule interacting protein 2 |
| GPR4 | ENST00000323040.4 | G protein-coupled receptor 4 |
| MANEAL | ENST00000397631.3 | mannosidase, endo-alpha-like |
| HDAC9 | ENST00000405010.3 | histone deacetylase 9 |
| GPR180 | ENST00000376958.4 | G protein-coupled receptor 180 |
| LUZP1 | ENST00000418342.1 | leucine zipper protein 1 |
| FGF11 | ENST00000293829.4 | fibroblast growth factor 11 |
| NMT2 | ENST00000378165.4 | N-myristoyltransferase 2 |
| MMS22L | ENST00000275053.4 | MMS22-like, DNA repair protein |
| GABRQ | ENST00000370306.2 | gamma-aminobutyric acid (GABA) A receptor, theta |
| ZNF320 | ENST00000391781.2 | zinc finger protein 320 |
| CDH12 | ENST00000504376.2 | cadherin 12, type 2 (N-cadherin 2) |
| KCNMB4 | ENST00000258111.4 | potassium large conductance calcium-activated channel, subfamily M, beta member 4 |
| C2CD3 | ENST00000334126.7 | C2 calcium-dependent domain containing 3 |
| BEND2 | ENST00000380033.4 | BEN domain containing 2 |
| ZBTB24 | ENST00000230122.3 | zinc finger and BTB domain containing 24 |
| SORT1 | ENST00000256637.6 | sortilin 1 |
| DGKI | ENST00000453654.2 | diacylglycerol kinase, iota |
| C11orf48 | ENST00000354588.3 | chromosome 11 open reading frame 48 |
| SLMAP | ENST00000295951.3 | sarcolemma associated protein |
| NT5DC1 | ENST00000319550.4 | 5'-nucleotidase domain containing 1 |
| ELFN2 | ENST00000402918.2 | extracellular leucine-rich repeat and fibronectin type III domain containing 2 |
| SRPX2 | ENST00000373004.3 | sushi-repeat containing protein, X-linked 2 |
| MYPN | ENST00000358913.5 | myopalladin |
| PEAK1 | ENST00000312493.4 | pseudopodium-enriched atypical kinase 1 |
| FLI1 | ENST00000527786.2 | Fli-1 proto-oncogene, ETS transcription factor |
| SMURF2 | ENST00000262435.9 | SMAD specific E3 ubiquitin protein ligase 2 |
| TOP2B | ENST00000542520.1 | topoisomerase (DNA) II beta 180kDa |
| TRPM2 | ENST00000300482.5 | transient receptor potential cation channel, subfamily M, member 2 |
| SEPN1 | ENST00000361547.2 | selenoprotein N, 1 |
| C21orf59 | ENST00000382549.4 | chromosome 21 open reading frame 59 |
| BACE2 | ENST00000328735.6 | beta-site APP-cleaving enzyme 2 |
| CRB1 | ENST00000367400.3 | crumbs homolog 1 (Drosophila) |
| PROSER2 | ENST00000277570.5 | proline and serine-rich protein 2 |
| PPP1R11 | ENST00000376773.1 | protein phosphatase 1, regulatory (inhibitor) subunit 11 |
| ARHGAP31 | ENST00000264245.4 | Rho GTPase activating protein 31 |
| SORL1 | ENST00000260197.7 | sortilin-related receptor, L(DLR class) A repeats containing |
| NCR3LG1 | ENST00000338965.4 | natural killer cell cytotoxicity receptor 3 ligand 1 |
| RP11-315D16.2 | ENST00000562767.1 | Uncharacterized protein |
| DCPS | ENST00000263579.4 | decapping enzyme, scavenger |
| CLSTN1 | ENST00000377298.4 | calsyntenin 1 |
| LACE1 | ENST00000368977.4 | lactation elevated 1 |
| RASAL1 | ENST00000546530.1 | RAS protein activator like 1 (GAP1 like) |
| TMEM65 | ENST00000297632.6 | transmembrane protein 65 |
| B4GALT1 | ENST00000379731.4 | UDP-Gal:betaGlcNAc beta 1,4- galactosyltransferase, polypeptide 1 |
| PIGM | ENST00000368090.2 | phosphatidylinositol glycan anchor biosynthesis, class M |
| MED21 | ENST00000282892.3 | mediator complex subunit 21 |
| NPR1 | ENST00000368680.3 | natriuretic peptide receptor A/guanylate cyclase A (atrionatriuretic peptide receptor A) |
| SUB1 | ENST00000265073.4 | SUB1 homolog (S. cerevisiae) |
| TIMM22 | ENST00000327158.4 | translocase of inner mitochondrial membrane 22 homolog (yeast) |
| AVPR1A | ENST00000299178.2 | arginine vasopressin receptor 1A |
| RANBP10 | ENST00000317506.3 | RAN binding protein 10 |
| RFX3 | ENST00000382004.3 | regulatory factor X, 3 (influences HLA class II expression) |
| MEI4 | ENST00000602452.2 | meiosis-specific 4 homolog (S. cerevisiae) |
| C17orf51 | ENST00000391411.5 | chromosome 17 open reading frame 51 |
| XKR4 | ENST00000327381.6 | XK, Kell blood group complex subunit-related family, member 4 |
| AGFG1 | ENST00000310078.8 | ArfGAP with FG repeats 1 |
| PPARD | ENST00000448077.2 | peroxisome proliferator-activated receptor delta |
| TTLL4 | ENST00000457313.1 | tubulin tyrosine ligase-like family, member 4 |
| PNMA5 | ENST00000361887.5 | paraneoplastic Ma antigen family member 5 |
| MAPK8IP2 | ENST00000399908.2 | mitogen-activated protein kinase 8 interacting protein 2 |
| ZMYM1 | ENST00000373330.1 | zinc finger, MYM-type 1 |
| RAB11FIP4 | ENST00000325874.8 | RAB11 family interacting protein 4 (class II) |
| DDHD1 | ENST00000323669.5 | DDHD domain containing 1 |
| STAT5B | ENST00000293328.3 | signal transducer and activator of transcription 5B |
| RBM28 | ENST00000223073.2 | RNA binding motif protein 28 |
| MYSM1 | ENST00000472487.1 | Myb-like, SWIRM and MPN domains 1 |
| HSPA6 | ENST00000309758.4 | heat shock 70kDa protein 6 (HSP70B') |
| C12orf23 | ENST00000548125.1 | chromosome 12 open reading frame 23 |
| ZNF501 | ENST00000396048.2 | zinc finger protein 501 |
| SFT2D2 | ENST00000271375.4 | SFT2 domain containing 2 |
| SPTY2D1 | ENST00000336349.5 | SPT2, Suppressor of Ty, domain containing 1 (S. cerevisiae) |
| EEF2K | ENST00000263026.5 | eukaryotic elongation factor-2 kinase |
| ADAM23 | ENST00000264377.3 | ADAM metallopeptidase domain 23 |
| ZNF431 | ENST00000311048.7 | zinc finger protein 431 |
| FAT3 | ENST00000298047.6 | FAT atypical cadherin 3 |
| ILDR2 | ENST00000469934.2 | immunoglobulin-like domain containing receptor 2 |
| HSPA12A | ENST00000369209.3 | heat shock 70kDa protein 12A |
| PLSCR1 | ENST00000342435.4 | phospholipid scramblase 1 |
| CNTNAP2 | ENST00000361727.3 | contactin associated protein-like 2 |
| MEF2C | ENST00000340208.5 | myocyte enhancer factor 2C |
| ZNF160 | ENST00000599056.1 | zinc finger protein 160 |
| LYRM2 | ENST00000520318.1 | LYR motif containing 2 |
| RPS6KA5 | ENST00000261991.3 | ribosomal protein S6 kinase, 90kDa, polypeptide 5 |
| SLC35D1 | ENST00000235345.5 | solute carrier family 35 (UDP-GlcA/UDP-GalNAc transporter), member D1 |
| MTMR10 | ENST00000435680.1 | myotubularin related protein 10 |
| TMTC1 | ENST00000256062.5 | transmembrane and tetratricopeptide repeat containing 1 |
| STX6 | ENST00000258301.5 | syntaxin 6 |
| SC5D | ENST00000264027.4 | sterol-C5-desaturase |
| PIK3C3 | ENST00000262039.4 | phosphatidylinositol 3-kinase, catalytic subunit type 3 |
| ATG9B | ENST00000605938.1 | autophagy related 9B |
| C6orf222 | ENST00000437635.2 | chromosome 6 open reading frame 222 |
| MRPS10 | ENST00000053468.3 | mitochondrial ribosomal protein S10 |
| BTD | ENST00000303498.5 | biotinidase |
| BAIAP3 | ENST00000397488.2 | BAI1-associated protein 3 |
| TPPP | ENST00000360578.5 | tubulin polymerization promoting protein |
| RAPGEF1 | ENST00000372189.3 | Rap guanine nucleotide exchange factor (GEF) 1 |
| TBPL1 | ENST00000237264.4 | TBP-like 1 |
| HECA | ENST00000367658.2 | headcase homolog (Drosophila) |
| RPS6KA3 | ENST00000379565.3 | ribosomal protein S6 kinase, 90kDa, polypeptide 3 |
| NTNG2 | ENST00000393229.3 | netrin G2 |
| THG1L | ENST00000231198.7 | tRNA-histidine guanylyltransferase 1-like (S. cerevisiae) |
| KIAA1462 | ENST00000375377.1 | KIAA1462 |
| SHROOM2 | ENST00000380913.3 | shroom family member 2 |
| EBF3 | ENST00000368648.3 | early B-cell factor 3 |
| STARD5 | ENST00000302824.6 | StAR-related lipid transfer (START) domain containing 5 |
| GIMAP1 | ENST00000307194.5 | GTPase, IMAP family member 1 |
| NFASC | ENST00000401399.1 | neurofascin |
| MADD | ENST00000406482.1 | MAP-kinase activating death domain |
| C4orf50 | ENST00000531445.1 | chromosome 4 open reading frame 50 |
| NFATC2 | ENST00000371564.3 | nuclear factor of activated T-cells, cytoplasmic, calcineurin-dependent 2 |
| C22orf46 | ENST00000402966.1 | chromosome 22 open reading frame 46 |
| SLC24A2 | ENST00000341998.2 | solute carrier family 24 (sodium/potassium/calcium exchanger), member 2 |
| SV2A | ENST00000369146.3 | synaptic vesicle glycoprotein 2A |
| VWDE | ENST00000275358.3 | von Willebrand factor D and EGF domains |
| INO80D | ENST00000403263.1 | INO80 complex subunit D |
| JPH2 | ENST00000372980.3 | junctophilin 2 |
| SOWAHB | ENST00000334306.2 | sosondowah ankyrin repeat domain family member B |
| AR | ENST00000374690.3 | androgen receptor |
| AJAP1 | ENST00000378191.4 | adherens junctions associated protein 1 |
| KCND3 | ENST00000369697.1 | potassium voltage-gated channel, Shal-related subfamily, member 3 |
| LRRC15 | ENST00000347624.3 | leucine rich repeat containing 15 |
| BAZ1B | ENST00000339594.4 | bromodomain adjacent to zinc finger domain, 1B |
| IL16 | ENST00000394660.2 | interleukin 16 |
| RFX1 | ENST00000254325.4 | regulatory factor X, 1 (influences HLA class II expression) |
| IL1A | ENST00000263339.3 | interleukin 1, alpha |
| CACNA1D | ENST00000288139.4 | calcium channel, voltage-dependent, L type, alpha 1D subunit |
| ANO3 | ENST00000256737.3 | anoctamin 3 |
| PI15 | ENST00000260113.2 | peptidase inhibitor 15 |
| OAS2 | ENST00000392583.2 | 2'-5'-oligoadenylate synthetase 2, 69/71kDa |
| TET3 | ENST00000409262.3 | tet methylcytosine dioxygenase 3 |
| CTD-2140B24.4 | ENST00000540096.2 | Zinc finger protein 268 |
| GAPVD1 | ENST00000470056.1 | GTPase activating protein and VPS9 domains 1 |
| GLRX3 | ENST00000368644.1 | glutaredoxin 3 |
| RRAS2 | ENST00000537760.1 | related RAS viral (r-ras) oncogene homolog 2 |
| C9orf69 | ENST00000561457.1 | chromosome 9 open reading frame 69 |
| DYRK3 | ENST00000367108.3 | dual-specificity tyrosine-(Y)-phosphorylation regulated kinase 3 |
| PTPN3 | ENST00000412145.1 | protein tyrosine phosphatase, non-receptor type 3 |
| EML1 | ENST00000262233.6 | echinoderm microtubule associated protein like 1 |
| ZNF284 | ENST00000421176.3 | zinc finger protein 284 |
| FAM126B | ENST00000418596.3 | family with sequence similarity 126, member B |
| RPF2 | ENST00000441448.2 | ribosome production factor 2 homolog (S. cerevisiae) |
| B3GALT1 | ENST00000392690.3 | UDP-Gal:betaGlcNAc beta 1,3-galactosyltransferase, polypeptide 1 |
| FBXW11 | ENST00000296933.6 | F-box and WD repeat domain containing 11 |
| HNRNPUL2 | ENST00000301785.5 | heterogeneous nuclear ribonucleoprotein U-like 2 |
| ANK2 | ENST00000357077.4 | ankyrin 2, neuronal |
| NFIA | ENST00000403491.3 | nuclear factor I/A |
| WNK3 | ENST00000375169.3 | WNK lysine deficient protein kinase 3 |
| PLEKHA2 | ENST00000420274.1 | pleckstrin homology domain containing, family A (phosphoinositide binding specific) member 2 |
| SLC5A8 | ENST00000536262.2 | solute carrier family 5 (sodium/monocarboxylate cotransporter), member 8 |
| MYO18B | ENST00000335473.7 | myosin XVIIIB |
| PARP15 | ENST00000483793.1 | poly (ADP-ribose) polymerase family, member 15 |
| MMAA | ENST00000281317.5 | methylmalonic aciduria (cobalamin deficiency) cblA type |
| PTPLAD2 | ENST00000495827.2 | protein tyrosine phosphatase-like A domain containing 2 |
| ABL2 | ENST00000502732.1 | c-abl oncogene 2, non-receptor tyrosine kinase |
| ACOX1 | ENST00000293217.5 | acyl-CoA oxidase 1, palmitoyl |
| KAZN | ENST00000422387.2 | kazrin, periplakin interacting protein |
| ZNF274 | ENST00000326804.4 | zinc finger protein 274 |
| SFMBT1 | ENST00000394752.3 | Scm-like with four mbt domains 1 |
| RBM17 | ENST00000446108.1 | RNA binding motif protein 17 |
| LRRC3DN | ENST00000596691.1 | LRRC3 downstream neighbor (non-protein coding) |
| ATP10A | ENST00000356865.6 | ATPase, class V, type 10A |
| CXADR | ENST00000356275.6 | coxsackie virus and adenovirus receptor |
| HNRNPA2B1 | ENST00000354667.4 | heterogeneous nuclear ribonucleoprotein A2/B1 |
| STXBP1 | ENST00000373302.3 | syntaxin binding protein 1 |
| FUT2 | ENST00000425340.2 | fucosyltransferase 2 (secretor status included) |
| CDH3 | ENST00000429102.2 | cadherin 3, type 1, P-cadherin (placental) |
| SESTD1 | ENST00000428443.3 | SEC14 and spectrin domains 1 |
| NOP9 | ENST00000396802.3 | NOP9 nucleolar protein |
| OPRL1 | ENST00000336866.2 | opiate receptor-like 1 |
| MRPS16 | ENST00000372945.3 | mitochondrial ribosomal protein S16 |
| FBXO32 | ENST00000517956.1 | F-box protein 32 |
| ZEB2 | ENST00000558170.2 | zinc finger E-box binding homeobox 2 |
| MIB1 | ENST00000261537.6 | mindbomb E3 ubiquitin protein ligase 1 |
| ACOT13 | ENST00000537591.1 | acyl-CoA thioesterase 13 |
| PIK3AP1 | ENST00000339364.5 | phosphoinositide-3-kinase adaptor protein 1 |
| SS18L2 | ENST00000447630.1 | synovial sarcoma translocation gene on chromosome 18-like 2 |
| STYX | ENST00000354586.4 | serine/threonine/tyrosine interacting protein |
| OPA3 | ENST00000263275.4 | optic atrophy 3 (autosomal recessive, with chorea and spastic paraplegia) |
| CBFA2T2 | ENST00000375279.2 | core-binding factor, runt domain, alpha subunit 2; translocated to, 2 |
| ARHGEF39 | ENST00000343259.3 | Rho guanine nucleotide exchange factor (GEF) 39 |
| ADCY2 | ENST00000338316.4 | adenylate cyclase 2 (brain) |
| SPRED1 | ENST00000299084.4 | sprouty-related, EVH1 domain containing 1 |
| PGAP1 | ENST00000354764.4 | post-GPI attachment to proteins 1 |
| POPDC3 | ENST00000254765.3 | popeye domain containing 3 |
| CRX | ENST00000221996.7 | cone-rod homeobox |
| OPCML | ENST00000331898.7 | opioid binding protein/cell adhesion molecule-like |
| ZFYVE1 | ENST00000318876.5 | zinc finger, FYVE domain containing 1 |
| VTI1B | ENST00000554659.1 | vesicle transport through interaction with t-SNAREs 1B |
| ATL3 | ENST00000398868.3 | atlastin GTPase 3 |
| IL5RA | ENST00000438560.1 | interleukin 5 receptor, alpha |
| LDLRAD4 | ENST00000399848.3 | low density lipoprotein receptor class A domain containing 4 |
| ZNF845 | ENST00000458035.1 | zinc finger protein 845 |
| SMAD4 | ENST00000398417.2 | SMAD family member 4 |
| MPRIP | ENST00000341712.4 | myosin phosphatase Rho interacting protein |
| POLR1B | ENST00000409894.3 | polymerase (RNA) I polypeptide B, 128kDa |
| ARSB | ENST00000264914.4 | arylsulfatase B |
| ZNF264 | ENST00000263095.6 | zinc finger protein 264 |
| CLIC2 | ENST00000369449.2 | chloride intracellular channel 2 |
| FGF12 | ENST00000445105.2 | fibroblast growth factor 12 |
| HS2ST1 | ENST00000370550.5 | heparan sulfate 2-O-sulfotransferase 1 |
| LZTS1 | ENST00000381569.1 | leucine zipper, putative tumor suppressor 1 |
| DESI1 | ENST00000263256.6 | desumoylating isopeptidase 1 |
| BTF3L4 | ENST00000489308.2 | basic transcription factor 3-like 4 |
| ARHGEF12 | ENST00000397843.2 | Rho guanine nucleotide exchange factor (GEF) 12 |
| GOPC | ENST00000368498.2 | golgi-associated PDZ and coiled-coil motif containing |
| ATP6V1A | ENST00000273398.3 | ATPase, H+ transporting, lysosomal 70kDa, V1 subunit A |
| TMEM41B | ENST00000528080.1 | transmembrane protein 41B |
| SMC4 | ENST00000357388.3 | structural maintenance of chromosomes 4 |
| ADAT1 | ENST00000307921.3 | adenosine deaminase, tRNA-specific 1 |
| ATP5G1 | ENST00000355938.5 | ATP synthase, H+ transporting, mitochondrial Fo complex, subunit C1 (subunit 9) |
| GEN1 | ENST00000381254.2 | GEN1 Holliday junction 5' flap endonuclease |
| EXOC8 | ENST00000360394.2 | exocyst complex component 8 |
| RAD1 | ENST00000382038.2 | RAD1 homolog (S. pombe) |
| RRAGD | ENST00000369415.4 | Ras-related GTP binding D |
| BVES | ENST00000314641.5 | blood vessel epicardial substance |
| NSD1 | ENST00000439151.2 | nuclear receptor binding SET domain protein 1 |
| GJC1 | ENST00000426548.1 | gap junction protein, gamma 1, 45kDa |
| SLC12A5 | ENST00000243964.3 | solute carrier family 12 (potassium/chloride transporter), member 5 |
| AP4E1 | ENST00000261842.5 | adaptor-related protein complex 4, epsilon 1 subunit |
| ZFP30 | ENST00000392144.1 | ZFP30 zinc finger protein |
| NANOS1 | ENST00000425699.1 | nanos homolog 1 (Drosophila) |
| FAM105B | ENST00000284274.4 | family with sequence similarity 105, member B |
| CRTC1 | ENST00000338797.6 | CREB regulated transcription coactivator 1 |
| PPP2R1B | ENST00000527614.1 | protein phosphatase 2, regulatory subunit A, beta |
| NDUFS1 | ENST00000233190.6 | NADH dehydrogenase (ubiquinone) Fe-S protein 1, 75kDa (NADH-coenzyme Q reductase) |
| ENAH | ENST00000366844.3 | enabled homolog (Drosophila) |
| CNTLN | ENST00000380641.4 | centlein, centrosomal protein |
| SLC16A10 | ENST00000368850.3 | solute carrier family 16 (aromatic amino acid transporter), member 10 |
| TMED10 | ENST00000303575.4 | transmembrane emp24-like trafficking protein 10 (yeast) |
| AMIGO1 | ENST00000369864.4 | adhesion molecule with Ig-like domain 1 |
| MICAL2 | ENST00000379612.3 | microtubule associated monooxygenase, calponin and LIM domain containing 2 |
| NCK1 | ENST00000469404.1 | NCK adaptor protein 1 |
| PAIP2B | ENST00000244221.8 | poly(A) binding protein interacting protein 2B |
| FREM2 | ENST00000280481.7 | FRAS1 related extracellular matrix protein 2 |
| CPOX | ENST00000264193.2 | coproporphyrinogen oxidase |
| DNAAF2 | ENST00000298292.8 | dynein, axonemal, assembly factor 2 |
| MARCH3 | ENST00000308660.5 | membrane-associated ring finger (C3HC4) 3, E3 ubiquitin protein ligase |
| ANKEF1 | ENST00000378380.3 | ankyrin repeat and EF-hand domain containing 1 |
| ABLIM3 | ENST00000326685.7 | actin binding LIM protein family, member 3 |
| USP49 | ENST00000394253.3 | ubiquitin specific peptidase 49 |
| UBXN7 | ENST00000296328.4 | UBX domain protein 7 |
| ENY2 | ENST00000520147.1 | enhancer of yellow 2 homolog (Drosophila) |
| CCR6 | ENST00000341935.5 | chemokine (C-C motif) receptor 6 |
| PCDH7 | ENST00000543491.1 | protocadherin 7 |
| C5orf63 | ENST00000535381.1 | chromosome 5 open reading frame 63 |
| RAP1A | ENST00000369709.3 | RAP1A, member of RAS oncogene family |
| ERBB3 | ENST00000267101.3 | v-erb-b2 avian erythroblastic leukemia viral oncogene homolog 3 |
| TPH1 | ENST00000250018.2 | tryptophan hydroxylase 1 |
| TGFBR3 | ENST00000212355.4 | transforming growth factor, beta receptor III |
| E2F3 | ENST00000346618.3 | E2F transcription factor 3 |
| LRRK1 | ENST00000388948.3 | leucine-rich repeat kinase 1 |
| ASXL2 | ENST00000435504.4 | additional sex combs like 2 (Drosophila) |
| RWDD1 | ENST00000466444.2 | RWD domain containing 1 |
| FKBP14 | ENST00000222803.5 | FK506 binding protein 14, 22 kDa |
| UGGT1 | ENST00000259253.6 | UDP-glucose glycoprotein glucosyltransferase 1 |
| UBE4A | ENST00000252108.3 | ubiquitination factor E4A |
| ZNF350 | ENST00000243644.4 | zinc finger protein 350 |
| DDI2 | ENST00000480945.1 | DNA-damage inducible 1 homolog 2 (S. cerevisiae) |
| KDM5A | ENST00000399788.2 | lysine (K)-specific demethylase 5A |
| DLEU1 | ENST00000378180.4 | deleted in lymphocytic leukemia 1 (non-protein coding) |
| ANAPC16 | ENST00000299381.4 | anaphase promoting complex subunit 16 |
| NHLRC2 | ENST00000369301.3 | NHL repeat containing 2 |
| MAVS | ENST00000428216.2 | mitochondrial antiviral signaling protein |
| EEPD1 | ENST00000242108.4 | endonuclease/exonuclease/phosphatase family domain containing 1 |
| PABPC1 | ENST00000318607.5 | poly(A) binding protein, cytoplasmic 1 |
| RNF169 | ENST00000299563.4 | ring finger protein 169 |
| HELZ | ENST00000358691.5 | helicase with zinc finger |
| ZNF468 | ENST00000595646.1 | zinc finger protein 468 |
| CEP41 | ENST00000223208.5 | centrosomal protein 41kDa |
| DR1 | ENST00000370272.4 | down-regulator of transcription 1, TBP-binding (negative cofactor 2) |
| TPRA1 | ENST00000296210.7 | transmembrane protein, adipocyte asscociated 1 |
| KIAA1549L | ENST00000321505.4 | KIAA1549-like |
| POFUT1 | ENST00000375749.3 | protein O-fucosyltransferase 1 |
| DNAJC21 | ENST00000382021.2 | DnaJ (Hsp40) homolog, subfamily C, member 21 |
| PTBP2 | ENST00000609116.1 | polypyrimidine tract binding protein 2 |
| TXLNB | ENST00000358430.3 | taxilin beta |
| SP110 | ENST00000258381.6 | SP110 nuclear body protein |
| RFK | ENST00000376736.1 | riboflavin kinase |
| GABRB2 | ENST00000393959.1 | gamma-aminobutyric acid (GABA) A receptor, beta 2 |
| HECW2 | ENST00000260983.3 | HECT, C2 and WW domain containing E3 ubiquitin protein ligase 2 |
| NAA35 | ENST00000361671.5 | N(alpha)-acetyltransferase 35, NatC auxiliary subunit |
| PIK3R3 | ENST00000262741.5 | phosphoinositide-3-kinase, regulatory subunit 3 (gamma) |
| SMC6 | ENST00000448223.2 | structural maintenance of chromosomes 6 |
| CEACAM1 | ENST00000403444.3 | carcinoembryonic antigen-related cell adhesion molecule 1 (biliary glycoprotein) |
| PYGO2 | ENST00000368457.2 | pygopus homolog 2 (Drosophila) |
| SLC45A4 | ENST00000519067.1 | solute carrier family 45, member 4 |
| LYSMD3 | ENST00000509384.1 | LysM, putative peptidoglycan-binding, domain containing 3 |
| SLC43A2 | ENST00000301335.5 | solute carrier family 43 (amino acid system L transporter), member 2 |
| SEMA4G | ENST00000210633.3 | sema domain, immunoglobulin domain (Ig), transmembrane domain (TM) and short cytoplasmic domain, (semaphorin) 4G |
| IFNE | ENST00000448696.3 | interferon, epsilon |
| EPS8 | ENST00000543523.1 | epidermal growth factor receptor pathway substrate 8 |
| IPMK | ENST00000373935.3 | inositol polyphosphate multikinase |
| SIPA1L2 | ENST00000366630.1 | signal-induced proliferation-associated 1 like 2 |
| RNF185 | ENST00000326132.6 | ring finger protein 185 |
| DAAM2 | ENST00000538976.1 | dishevelled associated activator of morphogenesis 2 |
| SGIP1 | ENST00000371036.3 | SH3-domain GRB2-like (endophilin) interacting protein 1 |
| COL17A1 | ENST00000353479.5 | collagen, type XVII, alpha 1 |
| NUCB1 | ENST00000405315.4 | nucleobindin 1 |
| ITGB8 | ENST00000222573.4 | integrin, beta 8 |
| FAM149B1 | ENST00000242505.6 | family with sequence similarity 149, member B1 |
| NR1D2 | ENST00000312521.4 | nuclear receptor subfamily 1, group D, member 2 |
| NQO1 | ENST00000379047.3 | NAD(P)H dehydrogenase, quinone 1 |
| ABCC5 | ENST00000334444.6 | ATP-binding cassette, sub-family C (CFTR/MRP), member 5 |
| EPT1 | ENST00000260585.7 | ethanolaminephosphotransferase 1 (CDP-ethanolamine-specific) |
| CTD-2162K18.4 | ENST00000590750.1 | Uncharacterized protein |
| TEX9 | ENST00000558083.2 | testis expressed 9 |
| NDFIP2 | ENST00000218652.7 | Nedd4 family interacting protein 2 |
| SLC15A2 | ENST00000489711.1 | solute carrier family 15 (oligopeptide transporter), member 2 |
| SLC16A7 | ENST00000261187.4 | solute carrier family 16 (monocarboxylate transporter), member 7 |
| ADCYAP1R1 | ENST00000304166.4 | adenylate cyclase activating polypeptide 1 (pituitary) receptor type I |
| SPN | ENST00000360121.3 | sialophorin |
| MEGF11 | ENST00000360698.4 | multiple EGF-like-domains 11 |
| STX18 | ENST00000306200.2 | syntaxin 18 |
| PAX5 | ENST00000358127.4 | paired box 5 |
| LRCH3 | ENST00000438796.2 | leucine-rich repeats and calponin homology (CH) domain containing 3 |
| TANGO6 | ENST00000261778.1 | transport and golgi organization 6 homolog (Drosophila) |
| SLAMF1 | ENST00000302035.6 | signaling lymphocytic activation molecule family member 1 |
| STARD9 | ENST00000290607.7 | StAR-related lipid transfer (START) domain containing 9 |
| SYN3 | ENST00000358763.2 | synapsin III |
| DPYSL4 | ENST00000338492.4 | dihydropyrimidinase-like 4 |
| WSCD2 | ENST00000332082.4 | WSC domain containing 2 |
| S1PR3 | ENST00000358157.2 | sphingosine-1-phosphate receptor 3 |
| TMC3 | ENST00000558726.1 | transmembrane channel-like 3 |
| KREMEN1 | ENST00000400335.4 | kringle containing transmembrane protein 1 |
| DDR1 | ENST00000446312.1 | discoidin domain receptor tyrosine kinase 1 |
| ARHGAP36 | ENST00000370922.1 | Rho GTPase activating protein 36 |
| DNAH10 | ENST00000409039.3 | dynein, axonemal, heavy chain 10 |
| KIF3B | ENST00000375712.3 | kinesin family member 3B |
| POM121C | ENST00000257665.5 | POM121 transmembrane nucleoporin C |
| SLC4A5 | ENST00000423644.1 | solute carrier family 4 (sodium bicarbonate cotransporter), member 5 |
| PIFO | ENST00000369738.4 | primary cilia formation |
| SLC25A43 | ENST00000217909.7 | solute carrier family 25, member 43 |
| GPR12 | ENST00000405846.3 | G protein-coupled receptor 12 |
| EPN3 | ENST00000268933.3 | epsin 3 |
| SSH3 | ENST00000376757.5 | slingshot protein phosphatase 3 |
| KLHL25 | ENST00000337975.5 | kelch-like family member 25 |
| GRK7 | ENST00000264952.2 | G protein-coupled receptor kinase 7 |
| C15orf39 | ENST00000567617.1 | chromosome 15 open reading frame 39 |
| PLCE1 | ENST00000371380.3 | phospholipase C, epsilon 1 |
| BAK1 | ENST00000442998.2 | BCL2-antagonist/killer 1 |
| MAP2K4 | ENST00000415385.3 | mitogen-activated protein kinase kinase 4 |
| KLHDC7A | ENST00000400664.1 | kelch domain containing 7A |
| ACACB | ENST00000543201.1 | acetyl-CoA carboxylase beta |
| PDE1C | ENST00000396193.1 | phosphodiesterase 1C, calmodulin-dependent 70kDa |
| ARRB1 | ENST00000420843.2 | arrestin, beta 1 |
| FGF23 | ENST00000237837.1 | fibroblast growth factor 23 |
| SAMD14 | ENST00000330175.4 | sterile alpha motif domain containing 14 |
| NTSR1 | ENST00000370501.3 | neurotensin receptor 1 (high affinity) |
| RUNX2 | ENST00000371432.3 | runt-related transcription factor 2 |
| CNTNAP1 | ENST00000264638.4 | contactin associated protein 1 |
| CSNK1G1 | ENST00000303052.7 | casein kinase 1, gamma 1 |
| MMP2 | ENST00000219070.4 | matrix metallopeptidase 2 (gelatinase A, 72kDa gelatinase, 72kDa type IV collagenase) |
| DRP2 | ENST00000402866.1 | dystrophin related protein 2 |
| PRR14L | ENST00000434485.1 | proline rich 14-like |
| F2RL3 | ENST00000248076.3 | coagulation factor II (thrombin) receptor-like 3 |
| NDST1 | ENST00000261797.6 | N-deacetylase/N-sulfotransferase (heparan glucosaminyl) 1 |
| F2R | ENST00000319211.4 | coagulation factor II (thrombin) receptor |
| CACNA1E | ENST00000526775.1 | calcium channel, voltage-dependent, R type, alpha 1E subunit |
| CTNNA3 | ENST00000433211.2 | catenin (cadherin-associated protein), alpha 3 |
| C5orf45 | ENST00000518219.1 | chromosome 5 open reading frame 45 |
| ELMSAN1 | ENST00000394071.2 | ELM2 and Myb/SANT-like domain containing 1 |
| PKHD1 | ENST00000371117.3 | polycystic kidney and hepatic disease 1 (autosomal recessive) |
| RAB36 | ENST00000263116.2 | RAB36, member RAS oncogene family |
| KLHL21 | ENST00000377663.3 | kelch-like family member 21 |
| KIAA0907 | ENST00000368320.3 | KIAA0907 |
| ARHGEF4 | ENST00000392953.3 | Rho guanine nucleotide exchange factor (GEF) 4 |
| CNOT6L | ENST00000504123.1 | CCR4-NOT transcription complex, subunit 6-like |
| RUFY2 | ENST00000388768.2 | RUN and FYVE domain containing 2 |
| SP6 | ENST00000342234.2 | Sp6 transcription factor |
| PLCB3 | ENST00000540288.1 | phospholipase C, beta 3 (phosphatidylinositol-specific) |
| FAM131B | ENST00000443739.2 | family with sequence similarity 131, member B |
| NAT9 | ENST00000357814.3 | N-acetyltransferase 9 (GCN5-related, putative) |
| ISY1-RAB43 | ENST00000418265.1 | ISY1-RAB43 readthrough |
| ATRNL1 | ENST00000355044.3 | attractin-like 1 |
| ARNT2 | ENST00000303329.4 | aryl-hydrocarbon receptor nuclear translocator 2 |
| KHNYN | ENST00000251343.5 | KH and NYN domain containing |
| TNFRSF1A | ENST00000162749.2 | tumor necrosis factor receptor superfamily, member 1A |
| KCNC1 | ENST00000379472.3 | potassium voltage-gated channel, Shaw-related subfamily, member 1 |
| PHF20 | ENST00000439301.1 | PHD finger protein 20 |
| MAU2 | ENST00000262815.8 | MAU2 sister chromatid cohesion factor |
| GPAM | ENST00000348367.4 | glycerol-3-phosphate acyltransferase, mitochondrial |
| CNGB1 | ENST00000564448.1 | cyclic nucleotide gated channel beta 1 |
| SH3RF3 | ENST00000309415.6 | SH3 domain containing ring finger 3 |
| KIF14 | ENST00000367350.4 | kinesin family member 14 |
| KCNQ3 | ENST00000388996.4 | potassium voltage-gated channel, KQT-like subfamily, member 3 |
| GDNF | ENST00000326524.2 | glial cell derived neurotrophic factor |
| LMAN2L | ENST00000264963.4 | lectin, mannose-binding 2-like |
| ELMOD3 | ENST00000315658.7 | ELMO/CED-12 domain containing 3 |
| LGI2 | ENST00000382114.4 | leucine-rich repeat LGI family, member 2 |
| CIITA | ENST00000324288.8 | class II, major histocompatibility complex, transactivator |
| ZNF609 | ENST00000326648.3 | zinc finger protein 609 |
| ZNF629 | ENST00000262525.4 | zinc finger protein 629 |
| GCK | ENST00000403799.3 | glucokinase (hexokinase 4) |
| ANKRD63 | ENST00000434396.1 | ankyrin repeat domain 63 |
| PCDH19 | ENST00000420881.2 | protocadherin 19 |
| NLRC5 | ENST00000436936.1 | NLR family, CARD domain containing 5 |
| FAM212B | ENST00000357260.5 | family with sequence similarity 212, member B |
| EP400 | ENST00000333577.4 | E1A binding protein p400 |
| PTPRF | ENST00000372414.3 | protein tyrosine phosphatase, receptor type, F |
| ANGEL1 | ENST00000251089.2 | angel homolog 1 (Drosophila) |
| RREB1 | ENST00000379938.2 | ras responsive element binding protein 1 |
| FAM168A | ENST00000064778.4 | family with sequence similarity 168, member A |
| PDLIM5 | ENST00000437932.1 | PDZ and LIM domain 5 |
| PI4K2A | ENST00000370649.3 | Phosphatidylinositol 4-kinase type 2-alpha; Uncharacterized protein |
| LCLAT1 | ENST00000309052.4 | lysocardiolipin acyltransferase 1 |
| HEG1 | ENST00000311127.4 | heart development protein with EGF-like domains 1 |
| MR1 | ENST00000367580.5 | major histocompatibility complex, class I-related |
| NUFIP2 | ENST00000225388.4 | nuclear fragile X mental retardation protein interacting protein 2 |
| ERN1 | ENST00000433197.3 | endoplasmic reticulum to nucleus signaling 1 |
| LAMP2 | ENST00000371335.4 | lysosomal-associated membrane protein 2 |
| SEMA3F | ENST00000002829.3 | sema domain, immunoglobulin domain (Ig), short basic domain, secreted, (semaphorin) 3F |
| COL6A1 | ENST00000361866.3 | collagen, type VI, alpha 1 |
| ZNF585B | ENST00000532828.2 | zinc finger protein 585B |
| CSRNP1 | ENST00000273153.5 | cysteine-serine-rich nuclear protein 1 |
| ZNF132 | ENST00000254166.3 | zinc finger protein 132 |
| SPSB1 | ENST00000328089.6 | splA/ryanodine receptor domain and SOCS box containing 1 |
| STT3A | ENST00000392708.4 | STT3A, subunit of the oligosaccharyltransferase complex (catalytic) |
| SIM1 | ENST00000369208.3 | single-minded homolog 1 (Drosophila) |
| GPR37 | ENST00000303921.2 | G protein-coupled receptor 37 (endothelin receptor type B-like) |
| BMPER | ENST00000297161.2 | BMP binding endothelial regulator |
| CRYZ | ENST00000370872.3 | crystallin, zeta (quinone reductase) |
| SLC5A12 | ENST00000396005.3 | solute carrier family 5 (sodium/monocarboxylate cotransporter), member 12 |
| TBCEL | ENST00000422003.2 | tubulin folding cofactor E-like |
| POLD3 | ENST00000263681.2 | polymerase (DNA-directed), delta 3, accessory subunit |
| RPS6KB1 | ENST00000225577.4 | ribosomal protein S6 kinase, 70kDa, polypeptide 1 |
| WDR59 | ENST00000262144.6 | WD repeat domain 59 |
| CYP2U1 | ENST00000332884.6 | cytochrome P450, family 2, subfamily U, polypeptide 1 |
| TTPAL | ENST00000372906.2 | tocopherol (alpha) transfer protein-like |
| INSR | ENST00000341500.5 | insulin receptor |
| STEAP3 | ENST00000409811.1 | STEAP family member 3, metalloreductase |
| COQ7 | ENST00000321998.5 | coenzyme Q7 homolog, ubiquinone (yeast) |
| SYNPO2 | ENST00000429713.2 | synaptopodin 2 |
| OGFRL1 | ENST00000370435.4 | opioid growth factor receptor-like 1 |
| MSI2 | ENST00000284073.2 | musashi RNA-binding protein 2 |
| HIF1AN | ENST00000299163.6 | hypoxia inducible factor 1, alpha subunit inhibitor |
| HS3ST3A1 | ENST00000284110.1 | heparan sulfate (glucosamine) 3-O-sulfotransferase 3A1 |
| GNL3L | ENST00000336470.4 | guanine nucleotide binding protein-like 3 (nucleolar)-like |
| F8 | ENST00000330287.6 | coagulation factor VIII, procoagulant component |
| ST3GAL6 | ENST00000265261.6 | ST3 beta-galactoside alpha-2,3-sialyltransferase 6 |
| TTBK2 | ENST00000267890.6 | tau tubulin kinase 2 |
| MCTP1 | ENST00000515393.1 | multiple C2 domains, transmembrane 1 |
| ADAM22 | ENST00000398204.4 | ADAM metallopeptidase domain 22 |
| BBX | ENST00000415149.2 | bobby sox homolog (Drosophila) |
| MEIS1 | ENST00000488550.1 | Meis homeobox 1 |
| TOM1L2 | ENST00000581396.1 | target of myb1-like 2 (chicken) |
| GPCPD1 | ENST00000379019.4 | glycerophosphocholine phosphodiesterase GDE1 homolog (S. cerevisiae) |
| DDX21 | ENST00000354185.4 | DEAD (Asp-Glu-Ala-Asp) box helicase 21 |
| LPPR5 | ENST00000370188.3 | Lipid phosphate phosphatase-related protein type 5 |
| HEBP2 | ENST00000607197.1 | heme binding protein 2 |
| ARHGAP42 | ENST00000524892.2 | Rho GTPase activating protein 42 |
| ARRDC2 | ENST00000379656.3 | arrestin domain containing 2 |
| EEA1 | ENST00000322349.8 | early endosome antigen 1 |
| MFF | ENST00000304593.9 | mitochondrial fission factor |
| EGFLAM | ENST00000322350.5 | EGF-like, fibronectin type III and laminin G domains |
| LONP2 | ENST00000285737.4 | lon peptidase 2, peroxisomal |
| HEMK1 | ENST00000232854.4 | HemK methyltransferase family member 1 |
| NEDD9 | ENST00000379446.5 | neural precursor cell expressed, developmentally down-regulated 9 |
| HECW1 | ENST00000395891.2 | HECT, C2 and WW domain containing E3 ubiquitin protein ligase 1 |
| UBR5 | ENST00000520539.1 | ubiquitin protein ligase E3 component n-recognin 5 |
| CCDC88A | ENST00000336838.6 | coiled-coil domain containing 88A |
| GDI2 | ENST00000380191.4 | GDP dissociation inhibitor 2 |
| DUSP8 | ENST00000397374.3 | dual specificity phosphatase 8 |
| NBEAL1 | ENST00000449802.1 | neurobeachin-like 1 |
| CD109 | ENST00000437994.2 | CD109 molecule |
| MED7 | ENST00000286317.5 | mediator complex subunit 7 |
| SPRED3 | ENST00000587013.1 | sprouty-related, EVH1 domain containing 3 |
| FUOM | ENST00000278025.4 | fucose mutarotase |
| GEMIN6 | ENST00000409011.1 | gem (nuclear organelle) associated protein 6 |
| FHL1 | ENST00000394155.2 | four and a half LIM domains 1 |
| WSCD1 | ENST00000574946.1 | WSC domain containing 1 |
| FMNL3 | ENST00000335154.5 | formin-like 3 |
| CTPS2 | ENST00000443824.1 | CTP synthase 2 |
| RPS16 | ENST00000599539.1 | ribosomal protein S16 |
| MFAP5 | ENST00000359478.2 | microfibrillar associated protein 5 |
| ENDOD1 | ENST00000278505.4 | endonuclease domain containing 1 |
| ZNF397 | ENST00000330501.7 | zinc finger protein 397 |
| ISPD | ENST00000407010.2 | isoprenoid synthase domain containing |
| GTPBP10 | ENST00000222511.6 | GTP-binding protein 10 (putative) |
| MTO1 | ENST00000498286.1 | mitochondrial tRNA translation optimization 1 |
| R3HCC1 | ENST00000265806.6 | R3H domain and coiled-coil containing 1 |
| ERCC6 | ENST00000355832.5 | excision repair cross-complementing rodent repair deficiency, complementation group 6 |
| MAP3K3 | ENST00000361357.3 | mitogen-activated protein kinase kinase kinase 3 |
| SLAIN2 | ENST00000264313.6 | SLAIN motif family, member 2 |
| RAB3GAP1 | ENST00000264158.8 | RAB3 GTPase activating protein subunit 1 (catalytic) |
| ABCG2 | ENST00000515655.1 | ATP-binding cassette, sub-family G (WHITE), member 2 |
| CLCN3 | ENST00000513761.1 | chloride channel, voltage-sensitive 3 |
| BSN | ENST00000296452.4 | bassoon presynaptic cytomatrix protein |
| CALCOCO1 | ENST00000548263.1 | calcium binding and coiled-coil domain 1 |
| SH3PXD2A | ENST00000369774.4 | SH3 and PX domains 2A |
| MAP3K9 | ENST00000554752.2 | mitogen-activated protein kinase kinase kinase 9 |
| LCORL | ENST00000326877.4 | ligand dependent nuclear receptor corepressor-like |
| MECP2 | ENST00000303391.6 | methyl CpG binding protein 2 (Rett syndrome) |
| ZBTB8A | ENST00000316459.4 | zinc finger and BTB domain containing 8A |
| TTC30A | ENST00000355689.5 | tetratricopeptide repeat domain 30A |
| IQCE | ENST00000402050.2 | IQ motif containing E |
| KRAS | ENST00000256078.4 | Kirsten rat sarcoma viral oncogene homolog |
| DUT | ENST00000559540.1 | deoxyuridine triphosphatase |
| BRI3 | ENST00000297290.3 | brain protein I3 |
| HAS2 | ENST00000303924.4 | hyaluronan synthase 2 |
| REEP5 | ENST00000545426.1 | receptor accessory protein 5 |
| CENPO | ENST00000380834.2 | centromere protein O |
| RBM33 | ENST00000341148.3 | RNA binding motif protein 33 |
| HECTD3 | ENST00000372168.3 | HECT domain containing E3 ubiquitin protein ligase 3 |
| DBT | ENST00000370132.4 | dihydrolipoamide branched chain transacylase E2 |
| SLC25A26 | ENST00000354883.6 | solute carrier family 25 (S-adenosylmethionine carrier), member 26 |
| ANKRD12 | ENST00000262126.4 | ankyrin repeat domain 12 |
| GAS7 | ENST00000437099.2 | growth arrest-specific 7 |
| PPARGC1B | ENST00000309241.5 | peroxisome proliferator-activated receptor gamma, coactivator 1 beta |
| STS | ENST00000217961.4 | steroid sulfatase (microsomal), isozyme S |
| APLF | ENST00000303795.4 | aprataxin and PNKP like factor |
| FAM134B | ENST00000399793.2 | family with sequence similarity 134, member B |
| ZNF678 | ENST00000343776.5 | zinc finger protein 678 |
| SEC63 | ENST00000369002.4 | SEC63 homolog (S. cerevisiae) |
| STX16 | ENST00000355957.5 | syntaxin 16 |
| GPR160 | ENST00000355897.5 | G protein-coupled receptor 160 |
| RBP2 | ENST00000232217.2 | retinol binding protein 2, cellular |
| TNRC6B | ENST00000335727.9 | trinucleotide repeat containing 6B |
| ZNF200 | ENST00000396868.3 | zinc finger protein 200 |
| SDK1 | ENST00000404826.2 | sidekick cell adhesion molecule 1 |
| GPN1 | ENST00000264718.3 | GPN-loop GTPase 1 |
| ORC4 | ENST00000392857.5 | origin recognition complex, subunit 4 |
| PIGV | ENST00000078527.4 | phosphatidylinositol glycan anchor biosynthesis, class V |
| TGFBRAP1 | ENST00000393359.2 | transforming growth factor, beta receptor associated protein 1 |
| STRBP | ENST00000447404.2 | spermatid perinuclear RNA binding protein |
| TRIM10 | ENST00000449742.2 | tripartite motif containing 10 |
| MBD5 | ENST00000407073.1 | methyl-CpG binding domain protein 5 |
| SBF2 | ENST00000256190.8 | SET binding factor 2 |
| ACSL3 | ENST00000357430.3 | acyl-CoA synthetase long-chain family member 3 |
| PPP3CB | ENST00000360663.5 | protein phosphatase 3, catalytic subunit, beta isozyme |
| TMEM67 | ENST00000453321.3 | transmembrane protein 67 |
| ZDHHC24 | ENST00000310442.3 | zinc finger, DHHC-type containing 24 |
| RAB10 | ENST00000264710.4 | RAB10, member RAS oncogene family |
| CENPC | ENST00000273853.6 | centromere protein C |
| MORC2 | ENST00000397641.3 | MORC family CW-type zinc finger 2 |
| ZNF324 | ENST00000536459.2 | zinc finger protein 324 |
| ASB1 | ENST00000264607.4 | ankyrin repeat and SOCS box containing 1 |
| CTBS | ENST00000370630.5 | chitobiase, di-N-acetyl- |
| SERINC3 | ENST00000342374.4 | serine incorporator 3 |
| SZT2 | ENST00000562955.1 | seizure threshold 2 homolog (mouse) |
| VCL | ENST00000372755.3 | vinculin |
| PSMG1 | ENST00000331573.3 | proteasome (prosome, macropain) assembly chaperone 1 |
| DDX51 | ENST00000397333.3 | DEAD (Asp-Glu-Ala-Asp) box polypeptide 51 |
| ERBB4 | ENST00000342788.4 | v-erb-b2 avian erythroblastic leukemia viral oncogene homolog 4 |
| RAB21 | ENST00000261263.3 | RAB21, member RAS oncogene family |
| STK32A | ENST00000397936.3 | serine/threonine kinase 32A |
| DTL | ENST00000366991.4 | denticleless E3 ubiquitin protein ligase homolog (Drosophila) |
| NEDD4L | ENST00000256832.7 | neural precursor cell expressed, developmentally down-regulated 4-like, E3 ubiquitin protein ligase |
| C1orf170 | ENST00000433179.2 | chromosome 1 open reading frame 170 |
| ZNF383 | ENST00000352998.3 | zinc finger protein 383 |
| SMG1 | ENST00000446231.2 | SMG1 phosphatidylinositol 3-kinase-related kinase |
| ZNF354B | ENST00000322434.3 | zinc finger protein 354B |
| ELK4 | ENST00000357992.4 | ELK4, ETS-domain protein (SRF accessory protein 1) |
| ADD2 | ENST00000264436.4 | adducin 2 (beta) |
| ZNF496 | ENST00000294753.4 | zinc finger protein 496 |
| ZNF639 | ENST00000326361.3 | zinc finger protein 639 |
| MPP2 | ENST00000377184.3 | membrane protein, palmitoylated 2 (MAGUK p55 subfamily member 2) |
| ZNF638 | ENST00000355812.3 | zinc finger protein 638 |
| ZNF708 | ENST00000356929.3 | zinc finger protein 708 |
| RAD50 | ENST00000378823.3 | RAD50 homolog (S. cerevisiae) |
| CYP4V2 | ENST00000378802.4 | cytochrome P450, family 4, subfamily V, polypeptide 2 |
| SLC30A10 | ENST00000366926.3 | solute carrier family 30, member 10 |
| TPRG1 | ENST00000345063.3 | tumor protein p63 regulated 1 |
| ZNF519 | ENST00000590202.1 | zinc finger protein 519 |
| SLC2A5 | ENST00000377424.4 | solute carrier family 2 (facilitated glucose/fructose transporter), member 5 |
| ZNF560 | ENST00000301480.4 | zinc finger protein 560 |
| ATP6V1C2 | ENST00000381661.3 | ATPase, H+ transporting, lysosomal 42kDa, V1 subunit C2 |
| KIAA1377 | ENST00000263468.8 | KIAA1377 |
| ONECUT3 | ENST00000382349.4 | one cut homeobox 3 |
| FAM120A | ENST00000333936.5 | family with sequence similarity 120A |
| ETS2 | ENST00000360214.3 | v-ets avian erythroblastosis virus E26 oncogene homolog 2 |
| ABCB8 | ENST00000356058.4 | ATP-binding cassette, sub-family B (MDR/TAP), member 8 |
| TRIM59 | ENST00000309784.4 | tripartite motif containing 59 |
| RBM12 | ENST00000374114.3 | RNA binding motif protein 12 |
| LARP4B | ENST00000316157.3 | La ribonucleoprotein domain family, member 4B |
| KIF6 | ENST00000287152.7 | kinesin family member 6 |
| ALG10B | ENST00000308742.4 | ALG10B, alpha-1,2-glucosyltransferase |
| OSGEPL1 | ENST00000519810.1 | O-sialoglycoprotein endopeptidase-like 1 |
| RP11-302B13.5 | ENST00000398092.4 | ADP-ribosylation factor 3 |
| DOCK5 | ENST00000276440.7 | dedicator of cytokinesis 5 |
| ZKSCAN2 | ENST00000328086.7 | zinc finger with KRAB and SCAN domains 2 |
| UBR3 | ENST00000272793.5 | ubiquitin protein ligase E3 component n-recognin 3 (putative) |
| CYB5B | ENST00000512062.1 | cytochrome b5 type B (outer mitochondrial membrane) |
| RHOH | ENST00000381799.5 | ras homolog family member H |
| RCAN3 | ENST00000374395.4 | RCAN family member 3 |
| RPAP2 | ENST00000610020.1 | RNA polymerase II associated protein 2 |
| TMEM120B | ENST00000449592.2 | transmembrane protein 120B |
| ZNF652 | ENST00000362063.2 | zinc finger protein 652 |
| RPP40 | ENST00000380051.2 | ribonuclease P/MRP 40kDa subunit |
| RALGPS2 | ENST00000367635.3 | Ral GEF with PH domain and SH3 binding motif 2 |
| KCTD20 | ENST00000373731.2 | potassium channel tetramerization domain containing 20 |
| POT1 | ENST00000357628.3 | protection of telomeres 1 |
| HPCAL4 | ENST00000372844.3 | hippocalcin like 4 |
| PARG | ENST00000402038.3 | poly (ADP-ribose) glycohydrolase |
| FOXK1 | ENST00000328914.4 | forkhead box K1 |
| NPAT | ENST00000278612.8 | nuclear protein, ataxia-telangiectasia locus |
| TECPR2 | ENST00000359520.7 | tectonin beta-propeller repeat containing 2 |
| ACVRL1 | ENST00000550683.1 | activin A receptor type II-like 1 |
| AGPAT4 | ENST00000366911.5 | 1-acylglycerol-3-phosphate O-acyltransferase 4 |
| WDR36 | ENST00000506538.2 | WD repeat domain 36 |
| AGA | ENST00000264595.2 | aspartylglucosaminidase |
| SERAC1 | ENST00000367102.2 | serine active site containing 1 |
| TRPC4 | ENST00000379705.3 | transient receptor potential cation channel, subfamily C, member 4 |
| PACS1 | ENST00000320580.4 | phosphofurin acidic cluster sorting protein 1 |
| XPO4 | ENST00000400602.2 | exportin 4 |
| ZNF334 | ENST00000457685.2 | zinc finger protein 334 |
| GRK4 | ENST00000503518.2 | G protein-coupled receptor kinase 4 |
| DHRS7B | ENST00000395511.3 | dehydrogenase/reductase (SDR family) member 7B |
| ARL5A | ENST00000295087.8 | ADP-ribosylation factor-like 5A |
| SAMD8 | ENST00000372687.4 | sterile alpha motif domain containing 8 |
| MED12L | ENST00000474524.1 | mediator complex subunit 12-like |
| FAM49B | ENST00000519824.2 | family with sequence similarity 49, member B |
| ACVR2A | ENST00000241416.7 | activin A receptor, type IIA |
| KRR1 | ENST00000229214.4 | KRR1, small subunit (SSU) processome component, homolog (yeast) |
| RIOK2 | ENST00000283109.3 | RIO kinase 2 |
| SMIM12 | ENST00000521580.2 | small integral membrane protein 12 |
| GOLGA6L9 | ENST00000300515.8 | golgin A6 family-like 9 |
| RLIM | ENST00000332687.6 | ring finger protein, LIM domain interacting |
| C5orf30 | ENST00000319933.2 | chromosome 5 open reading frame 30 |
| FAM210A | ENST00000322247.3 | family with sequence similarity 210, member A |
| ACTN2 | ENST00000366578.4 | actinin, alpha 2 |
| SMCHD1 | ENST00000320876.6 | structural maintenance of chromosomes flexible hinge domain containing 1 |
| ATP1B3 | ENST00000539728.1 | ATPase, Na+/K+ transporting, beta 3 polypeptide |
| ZSCAN2 | ENST00000541040.1 | zinc finger and SCAN domain containing 2 |
| INPP5K | ENST00000421807.2 | inositol polyphosphate-5-phosphatase K |
| EBF2 | ENST00000535548.1 | early B-cell factor 2 |
| DNAJC11 | ENST00000377577.5 | DnaJ (Hsp40) homolog, subfamily C, member 11 |
| MYCBP | ENST00000397572.2 | MYC binding protein |
| UMPS | ENST00000232607.2 | uridine monophosphate synthetase |
| CBS | ENST00000398158.1 | cystathionine-beta-synthase |
| TYW5 | ENST00000354611.4 | tRNA-yW synthesizing protein 5 |
| REEP3 | ENST00000373758.4 | receptor accessory protein 3 |
| BNIP2 | ENST00000267859.3 | BCL2/adenovirus E1B 19kDa interacting protein 2 |
| ATP6V1B2 | ENST00000276390.2 | ATPase, H+ transporting, lysosomal 56/58kDa, V1 subunit B2 |
| NDUFC1 | ENST00000394228.1 | NADH dehydrogenase (ubiquinone) 1, subcomplex unknown, 1, 6kDa |
| KDM1B | ENST00000388870.2 | lysine (K)-specific demethylase 1B |
| CDC42EP2 | ENST00000279249.2 | CDC42 effector protein (Rho GTPase binding) 2 |
| PXK | ENST00000463280.1 | PX domain containing serine/threonine kinase |
| TRIM45 | ENST00000256649.4 | tripartite motif containing 45 |
| TXNDC17 | ENST00000250101.5 | thioredoxin domain containing 17 |
| BRWD1 | ENST00000342449.3 | bromodomain and WD repeat domain containing 1 |
| TFCP2L1 | ENST00000263707.5 | transcription factor CP2-like 1 |
| XIRP1 | ENST00000396251.1 | xin actin-binding repeat containing 1 |
| CYBB | ENST00000378588.4 | cytochrome b-245, beta polypeptide |
| ERCC4 | ENST00000311895.7 | excision repair cross-complementing rodent repair deficiency, complementation group 4 |
| TRANK1 | ENST00000428977.2 | tetratricopeptide repeat and ankyrin repeat containing 1 |
| HIVEP3 | ENST00000372583.1 | human immunodeficiency virus type I enhancer binding protein 3 |
| EPHA7 | ENST00000369303.4 | EPH receptor A7 |
| MLXIPL | ENST00000434326.1 | MLX interacting protein-like |
| GOLGA8F | ENST00000526619.2 | golgin A8 family, member F |
| SHISA6 | ENST00000441885.3 | shisa family member 6 |
| PRIMA1 | ENST00000393140.1 | proline rich membrane anchor 1 |
| KCNJ6 | ENST00000609713.1 | potassium inwardly-rectifying channel, subfamily J, member 6 |
| IKBKG | ENST00000393549.2 | inhibitor of kappa light polypeptide gene enhancer in B-cells, kinase gamma |
| ATP2B4 | ENST00000367218.3 | ATPase, Ca++ transporting, plasma membrane 4 |
| SORCS2 | ENST00000507866.2 | sortilin-related VPS10 domain containing receptor 2 |
| GOLGA8G | ENST00000329523.6 | golgin A8 family, member G |
| HDAC7 | ENST00000380610.4 | histone deacetylase 7 |
| FBXO30 | ENST00000237281.4 | F-box protein 30 |
| ST8SIA3 | ENST00000324000.3 | ST8 alpha-N-acetyl-neuraminide alpha-2,8-sialyltransferase 3 |
| STXBP5L | ENST00000273666.6 | syntaxin binding protein 5-like |
| GAB3 | ENST00000369575.3 | GRB2-associated binding protein 3 |
| NWD1 | ENST00000524140.2 | NACHT and WD repeat domain containing 1 |
| ATRN | ENST00000262919.5 | attractin |
| KIRREL | ENST00000368172.1 | kin of IRRE like (Drosophila) |
| COL6A6 | ENST00000358511.6 | collagen, type VI, alpha 6 |
| ZYG11B | ENST00000294353.6 | zyg-11 family member B, cell cycle regulator |
| ZNF860 | ENST00000360311.4 | zinc finger protein 860 |
| TAOK1 | ENST00000261716.3 | TAO kinase 1 |
| MDGA1 | ENST00000297153.7 | MAM domain containing glycosylphosphatidylinositol anchor 1 |
| HUNK | ENST00000270112.2 | hormonally up-regulated Neu-associated kinase |
| XPR1 | ENST00000367590.4 | xenotropic and polytropic retrovirus receptor 1 |
| HHIPL1 | ENST00000330710.5 | HHIP-like 1 |
| ZNF813 | ENST00000396403.4 | zinc finger protein 813 |
| PPP1CB | ENST00000395366.2 | protein phosphatase 1, catalytic subunit, beta isozyme |
| MTUS1 | ENST00000381869.3 | microtubule associated tumor suppressor 1 |
| ERC1 | ENST00000355446.5 | ELKS/RAB6-interacting/CAST family member 1 |
| OTUD7A | ENST00000307050.4 | OTU domain containing 7A |
| PPP6C | ENST00000373547.4 | protein phosphatase 6, catalytic subunit |
| NPTX1 | ENST00000306773.4 | neuronal pentraxin I |
| SEPT11 | ENST00000264893.6 | septin 11 |
| MFN1 | ENST00000471841.1 | mitofusin 1 |
| AP1G1 | ENST00000299980.4 | adaptor-related protein complex 1, gamma 1 subunit |
| NKD1 | ENST00000268459.3 | naked cuticle homolog 1 (Drosophila) |
| SIM2 | ENST00000290399.6 | single-minded homolog 2 (Drosophila) |
| SIX4 | ENST00000216513.4 | SIX homeobox 4 |
| LRP10 | ENST00000359591.4 | low density lipoprotein receptor-related protein 10 |
| DDX31 | ENST00000372159.3 | DEAD (Asp-Glu-Ala-Asp) box polypeptide 31 |
| SYNJ1 | ENST00000357345.3 | synaptojanin 1 |
| MAP7D3 | ENST00000316077.9 | MAP7 domain containing 3 |
| EOGT | ENST00000383701.3 | EGF domain-specific O-linked N-acetylglucosamine (GlcNAc) transferase |
| LONRF2 | ENST00000393437.3 | LON peptidase N-terminal domain and ring finger 2 |
| DSTYK | ENST00000367160.4 | dual serine/threonine and tyrosine protein kinase |
| TIMM10 | ENST00000257245.4 | translocase of inner mitochondrial membrane 10 homolog (yeast) |
| TEP1 | ENST00000262715.5 | telomerase-associated protein 1 |
| PTGS1 | ENST00000362012.2 | prostaglandin-endoperoxide synthase 1 (prostaglandin G/H synthase and cyclooxygenase) |
| ZBTB40 | ENST00000404138.1 | zinc finger and BTB domain containing 40 |
| IFNAR2 | ENST00000404220.3 | interferon (alpha, beta and omega) receptor 2 |
| CLCN5 | ENST00000376088.3 | chloride channel, voltage-sensitive 5 |
| TTC39A | ENST00000530004.1 | tetratricopeptide repeat domain 39A |
| SMC2 | ENST00000374793.3 | structural maintenance of chromosomes 2 |
| BCAS2 | ENST00000369541.3 | breast carcinoma amplified sequence 2 |
| IMP4 | ENST00000409935.1 | IMP4, U3 small nucleolar ribonucleoprotein, homolog (yeast) |
| HOXD12 | ENST00000404162.2 | homeobox D12 |
| RPL24 | ENST00000495401.1 | ribosomal protein L24 |
| BCORL1 | ENST00000540052.1 | BCL6 corepressor-like 1 |
| VCPIP1 | ENST00000310421.4 | valosin containing protein (p97)/p47 complex interacting protein 1 |
| C14orf28 | ENST00000325192.3 | chromosome 14 open reading frame 28 |
| MAN1C1 | ENST00000374332.4 | mannosidase, alpha, class 1C, member 1 |
| EIF5A2 | ENST00000474096.1 | eukaryotic translation initiation factor 5A2 |
| DCTN6 | ENST00000221114.3 | dynactin 6 |
| LZIC | ENST00000377223.1 | leucine zipper and CTNNBIP1 domain containing |
| STON2 | ENST00000267540.2 | stonin 2 |
| MARCH6 | ENST00000274140.5 | membrane-associated ring finger (C3HC4) 6, E3 ubiquitin protein ligase |
| RAD54L2 | ENST00000409535.2 | RAD54-like 2 (S. cerevisiae) |
| ADH5 | ENST00000296412.8 | alcohol dehydrogenase 5 (class III), chi polypeptide |
| JDP2 | ENST00000435893.2 | Jun dimerization protein 2 |
| SIKE1 | ENST00000369528.5 | suppressor of IKBKE 1 |
| ANKRD33B | ENST00000296657.5 | ankyrin repeat domain 33B |
| LPHN3 | ENST00000512091.2 | latrophilin 3 |
| FAM84A | ENST00000295092.2 | family with sequence similarity 84, member A |
| AHSA2 | ENST00000394457.3 | AHA1, activator of heat shock 90kDa protein ATPase homolog 2 (yeast) |
| DUSP4 | ENST00000240100.2 | dual specificity phosphatase 4 |
| PML | ENST00000565898.1 | promyelocytic leukemia |
| INTS6 | ENST00000311234.4 | integrator complex subunit 6 |
| CHCHD7 | ENST00000518801.1 | coiled-coil-helix-coiled-coil-helix domain containing 7 |
| MAD2L1 | ENST00000296509.6 | MAD2 mitotic arrest deficient-like 1 (yeast) |
| CEP76 | ENST00000262127.2 | centrosomal protein 76kDa |
| NXPH3 | ENST00000328741.5 | neurexophilin 3 |
| IRAK3 | ENST00000261233.4 | interleukin-1 receptor-associated kinase 3 |
| MLXIP | ENST00000319080.7 | MLX interacting protein |
| FBXO9 | ENST00000244426.6 | F-box protein 9 |
| BNIP3L | ENST00000380629.2 | BCL2/adenovirus E1B 19kDa interacting protein 3-like |
| HFE | ENST00000357618.5 | hemochromatosis |
| ADRBK2 | ENST00000324198.6 | adrenergic, beta, receptor kinase 2 |
| AKAP8 | ENST00000269701.2 | A kinase (PRKA) anchor protein 8 |
| FPGS | ENST00000373245.1 | folylpolyglutamate synthase |
| TOX | ENST00000361421.1 | thymocyte selection-associated high mobility group box |
| UBD | ENST00000377050.4 | ubiquitin D |
| SAR1B | ENST00000402673.2 | SAR1 homolog B (S. cerevisiae) |
| FREM1 | ENST00000380881.4 | FRAS1 related extracellular matrix 1 |
| TCF21 | ENST00000367882.4 | transcription factor 21 |
| WDR41 | ENST00000296679.4 | WD repeat domain 41 |
| HMX2 | ENST00000339992.3 | H6 family homeobox 2 |
| F2RL1 | ENST00000296677.4 | coagulation factor II (thrombin) receptor-like 1 |
| MRPL17 | ENST00000288937.6 | mitochondrial ribosomal protein L17 |
| GDF5OS | ENST00000374375.1 | growth differentiation factor 5 opposite strand |
| SOD2 | ENST00000538183.2 | superoxide dismutase 2, mitochondrial |
| CA5B | ENST00000454127.2 | carbonic anhydrase VB, mitochondrial |
| FAM134A | ENST00000430297.2 | family with sequence similarity 134, member A |
| SMU1 | ENST00000397149.3 | smu-1 suppressor of mec-8 and unc-52 homolog (C. elegans) |
| KIAA1715 | ENST00000272748.4 | KIAA1715 |
| FRMD4B | ENST00000398540.3 | FERM domain containing 4B |
| ATP5S | ENST00000245448.6 | ATP synthase, H+ transporting, mitochondrial Fo complex, subunit s (factor B) |
| PRPF4B | ENST00000337659.6 | pre-mRNA processing factor 4B |
| KCNJ8 | ENST00000240662.2 | potassium inwardly-rectifying channel, subfamily J, member 8 |
| FAM179A | ENST00000379558.4 | family with sequence similarity 179, member A |
| TERF2 | ENST00000254942.3 | telomeric repeat binding factor 2 |
| RASSF9 | ENST00000361228.3 | Ras association (RalGDS/AF-6) domain family (N-terminal) member 9 |
| CNIH1 | ENST00000395573.4 | cornichon family AMPA receptor auxiliary protein 1 |
| GABPB2 | ENST00000368918.3 | GA binding protein transcription factor, beta subunit 2 |
| PELO | ENST00000274311.2 | pelota homolog (Drosophila) |
| AGPAT5 | ENST00000285518.6 | 1-acylglycerol-3-phosphate O-acyltransferase 5 |
| UBE2F | ENST00000272930.4 | ubiquitin-conjugating enzyme E2F (putative) |
| SH3PXD2B | ENST00000311601.5 | SH3 and PX domains 2B |
| IGF1R | ENST00000268035.6 | insulin-like growth factor 1 receptor |
| ELF3 | ENST00000367284.5 | E74-like factor 3 (ets domain transcription factor, epithelial-specific ) |
| A1CF | ENST00000374001.2 | APOBEC1 complementation factor |
| WDR33 | ENST00000322313.4 | WD repeat domain 33 |
| XPNPEP3 | ENST00000357137.4 | X-prolyl aminopeptidase (aminopeptidase P) 3, putative |
| KIAA0408 | ENST00000483725.3 | KIAA0408 |
| CYLD | ENST00000540145.1 | cylindromatosis (turban tumor syndrome) |
| ANG | ENST00000336811.6 | angiogenin, ribonuclease, RNase A family, 5 |
| HARBI1 | ENST00000326737.3 | harbinger transposase derived 1 |
| HSPA9 | ENST00000297185.3 | heat shock 70kDa protein 9 (mortalin) |
| EDC3 | ENST00000315127.4 | enhancer of mRNA decapping 3 |
| LRRC27 | ENST00000392638.2 | leucine rich repeat containing 27 |
| G3BP1 | ENST00000394123.3 | GTPase activating protein (SH3 domain) binding protein 1 |
| FAM96A | ENST00000557835.1 | family with sequence similarity 96, member A |
| MPDZ | ENST00000319217.7 | multiple PDZ domain protein |
| NAA50 | ENST00000240922.3 | N(alpha)-acetyltransferase 50, NatE catalytic subunit |
| ZNF805 | ENST00000535550.1 | zinc finger protein 805 |
| RAP2B | ENST00000323534.2 | RAP2B, member of RAS oncogene family |
| SH3GLB1 | ENST00000370558.4 | SH3-domain GRB2-like endophilin B1 |
| KPNB1 | ENST00000290158.4 | karyopherin (importin) beta 1 |
| KNSTRN | ENST00000608100.1 | kinetochore-localized astrin/SPAG5 binding protein |
| EBAG9 | ENST00000337573.5 | estrogen receptor binding site associated, antigen, 9 |
| CDS2 | ENST00000460006.1 | CDP-diacylglycerol synthase (phosphatidate cytidylyltransferase) 2 |
| CELF2 | ENST00000379261.4 | CUGBP, Elav-like family member 2 |
| SLC6A19 | ENST00000304460.10 | solute carrier family 6 (neutral amino acid transporter), member 19 |
| CPEB3 | ENST00000412050.4 | cytoplasmic polyadenylation element binding protein 3 |
| FDX1 | ENST00000260270.2 | ferredoxin 1 |
| ZSCAN16 | ENST00000340487.4 | zinc finger and SCAN domain containing 16 |
| MOCS3 | ENST00000244051.1 | molybdenum cofactor synthesis 3 |
| RABL5 | ENST00000517481.1 | RAB, member RAS oncogene family-like 5 |
| ZBTB6 | ENST00000373659.3 | zinc finger and BTB domain containing 6 |
| SULF2 | ENST00000359930.4 | sulfatase 2 |
| EFHC1 | ENST00000371068.5 | EF-hand domain (C-terminal) containing 1 |
| SPEM1 | ENST00000323675.3 | spermatid maturation 1 |
| DICER1 | ENST00000541352.1 | dicer 1, ribonuclease type III |
| AC007375.1 | ENST00000600936.1 | Uncharacterized protein; cDNA FLJ43210 fis, clone FEBRA2020582 |
| CPPED1 | ENST00000381774.4 | calcineurin-like phosphoesterase domain containing 1 |
| SNX24 | ENST00000513881.1 | sorting nexin 24 |
| TMEM44 | ENST00000381975.3 | transmembrane protein 44 |
| KNOP1 | ENST00000219837.7 | lysine-rich nucleolar protein 1 |
| GGA2 | ENST00000309859.4 | golgi-associated, gamma adaptin ear containing, ARF binding protein 2 |
| KIAA1551 | ENST00000312561.4 | KIAA1551 |
| PIK3CA | ENST00000263967.3 | phosphatidylinositol-4,5-bisphosphate 3-kinase, catalytic subunit alpha |
| RCC1 | ENST00000373833.6 | regulator of chromosome condensation 1 |
| COG8 | ENST00000306875.4 | component of oligomeric golgi complex 8 |
| WISP2 | ENST00000372868.2 | WNT1 inducible signaling pathway protein 2 |
| SHFM1 | ENST00000248566.2 | split hand/foot malformation (ectrodactyly) type 1 |
| VCAM1 | ENST00000347652.2 | vascular cell adhesion molecule 1 |
| GAN | ENST00000568107.2 | gigaxonin |
| CCNL1 | ENST00000295926.3 | cyclin L1 |
| RMND5A | ENST00000283632.4 | required for meiotic nuclear division 5 homolog A (S. cerevisiae) |
| STAMBP | ENST00000394070.2 | STAM binding protein |
| ZFAND4 | ENST00000344646.5 | zinc finger, AN1-type domain 4 |
| GID4 | ENST00000268719.4 | GID complex subunit 4 |
| RP11-343C2.12 | ENST00000562949.1 | Conserved oligomeric Golgi complex subunit 8 |
| DPY19L2 | ENST00000324472.4 | dpy-19-like 2 (C. elegans) |
| SPHAR | ENST00000366688.3 | S-phase response (cyclin related) |
| ABCF3 | ENST00000429586.2 | ATP-binding cassette, sub-family F (GCN20), member 3 |
| PDF | ENST00000288022.1 | peptide deformylase (mitochondrial) |
| FGFR1OP | ENST00000366847.4 | FGFR1 oncogene partner |
| RAD51 | ENST00000423169.2 | RAD51 recombinase |
| MAGIX | ENST00000376338.3 | MAGI family member, X-linked |
| GTF2F2 | ENST00000340473.6 | general transcription factor IIF, polypeptide 2, 30kDa |
| ALDH5A1 | ENST00000357578.3 | aldehyde dehydrogenase 5 family, member A1 |
| ITIH4 | ENST00000266041.4 | inter-alpha-trypsin inhibitor heavy chain family, member 4 |
| XRRA1 | ENST00000527087.1 | X-ray radiation resistance associated 1 |
| GFRA1 | ENST00000439649.3 | GDNF family receptor alpha 1 |
| GJA9 | ENST00000454994.2 | gap junction protein, alpha 9, 59kDa |
| GLUL | ENST00000331872.6 | glutamate-ammonia ligase |
| VSIG10 | ENST00000359236.5 | V-set and immunoglobulin domain containing 10 |
| MMADHC | ENST00000303319.5 | methylmalonic aciduria (cobalamin deficiency) cblD type, with homocystinuria |
| FAF1 | ENST00000396153.2 | Fas (TNFRSF6) associated factor 1 |
| PER1 | ENST00000317276.4 | period circadian clock 1 |
| ZNF555 | ENST00000334241.4 | zinc finger protein 555 |
| UPP1 | ENST00000331803.4 | uridine phosphorylase 1 |
| HNRNPK | ENST00000376281.4 | heterogeneous nuclear ribonucleoprotein K |
| UTP6 | ENST00000261708.4 | UTP6, small subunit (SSU) processome component, homolog (yeast) |
| NUP37 | ENST00000552283.1 | nucleoporin 37kDa |
| EMP1 | ENST00000256951.5 | epithelial membrane protein 1 |
| TSR1 | ENST00000301364.5 | TSR1, 20S rRNA accumulation, homolog (S. cerevisiae) |
| SRGAP3 | ENST00000383836.3 | SLIT-ROBO Rho GTPase activating protein 3 |
| PPIP5K2 | ENST00000321521.9 | diphosphoinositol pentakisphosphate kinase 2 |
| KCTD16 | ENST00000507359.3 | potassium channel tetramerization domain containing 16 |
| POU4F1 | ENST00000377208.5 | POU class 4 homeobox 1 |
| N4BP2L2 | ENST00000267068.3 | NEDD4 binding protein 2-like 2 |
| NCOA1 | ENST00000405141.1 | nuclear receptor coactivator 1 |
| C7orf55-LUC7L2 | ENST00000354926.4 | C7orf55-LUC7L2 readthrough |
| SLFN5 | ENST00000299977.4 | schlafen family member 5 |
| COX7C | ENST00000509578.1 | cytochrome c oxidase subunit VIIc |
| ADAMTS4 | ENST00000367996.5 | ADAM metallopeptidase with thrombospondin type 1 motif, 4 |
| FBXW8 | ENST00000455858.2 | F-box and WD repeat domain containing 8 |
| C15orf38 | ENST00000357484.5 | chromosome 15 open reading frame 38 |
| CHORDC1 | ENST00000320585.6 | cysteine and histidine-rich domain (CHORD) containing 1 |
| ABCB7 | ENST00000253577.3 | ATP-binding cassette, sub-family B (MDR/TAP), member 7 |
| ZNF507 | ENST00000311921.4 | zinc finger protein 507 |
| PPM1L | ENST00000498165.1 | protein phosphatase, Mg2+/Mn2+ dependent, 1L |
| LUC7L2 | ENST00000541515.3 | LUC7-like 2 (S. cerevisiae) |
| ZNF273 | ENST00000545510.1 | zinc finger protein 273 |
| FANCM | ENST00000267430.5 | Fanconi anemia, complementation group M |
| DGCR6L | ENST00000248879.3 | DiGeorge syndrome critical region gene 6-like |
| IST1 | ENST00000329908.8 | increased sodium tolerance 1 homolog (yeast) |
| OSGIN2 | ENST00000451899.2 | oxidative stress induced growth inhibitor family member 2 |
| TNFRSF11A | ENST00000586569.1 | tumor necrosis factor receptor superfamily, member 11a, NFKB activator |
| PEX26 | ENST00000329627.7 | peroxisomal biogenesis factor 26 |
| APTX | ENST00000436040.2 | aprataxin |
| DDR2 | ENST00000367922.3 | discoidin domain receptor tyrosine kinase 2 |
| C1orf50 | ENST00000372525.5 | chromosome 1 open reading frame 50 |
| PSMC4 | ENST00000157812.2 | proteasome (prosome, macropain) 26S subunit, ATPase, 4 |
| GLRX2 | ENST00000367439.3 | glutaredoxin 2 |
| FZD5 | ENST00000295417.3 | frizzled family receptor 5 |
| RAB3C | ENST00000282878.4 | RAB3C, member RAS oncogene family |
| CCDC115 | ENST00000437688.2 | coiled-coil domain containing 115 |
| HERPUD2 | ENST00000396081.1 | HERPUD family member 2 |
| ARPC2 | ENST00000295685.10 | actin related protein 2/3 complex, subunit 2, 34kDa |
| SUSD1 | ENST00000374263.3 | sushi domain containing 1 |
| RHCG | ENST00000544600.1 | Rh family, C glycoprotein |
| SHROOM1 | ENST00000378679.3 | shroom family member 1 |
| C18orf21 | ENST00000592875.1 | chromosome 18 open reading frame 21 |
| FKBP9 | ENST00000242209.4 | FK506 binding protein 9, 63 kDa |
| HNRNPR | ENST00000478691.1 | heterogeneous nuclear ribonucleoprotein R |
| IARS2 | ENST00000366922.1 | isoleucyl-tRNA synthetase 2, mitochondrial |
| SCIN | ENST00000297029.5 | scinderin |
| C1orf27 | ENST00000287859.6 | chromosome 1 open reading frame 27 |
| ESRRB | ENST00000261532.7 | estrogen-related receptor beta |
| PNP | ENST00000361505.5 | purine nucleoside phosphorylase |
| PITPNA | ENST00000313486.7 | phosphatidylinositol transfer protein, alpha |
| MROH1 | ENST00000423230.2 | maestro heat-like repeat family member 1 |
| FCF1 | ENST00000341162.4 | FCF1 rRNA-processing protein |
| POU3F3 | ENST00000361360.2 | POU class 3 homeobox 3 |
| IFRG15 | ENST00000553856.1 | Homo sapiens torsin A interacting protein 2 (TOR1AIP2), transcript variant 1, mRNA. |
| ERCC6L | ENST00000373657.1 | excision repair cross-complementing rodent repair deficiency, complementation group 6-like |
| MPC2 | ENST00000367846.4 | mitochondrial pyruvate carrier 2 |
| FAM122A | ENST00000394264.3 | family with sequence similarity 122A |
| LRIG3 | ENST00000379141.4 | leucine-rich repeats and immunoglobulin-like domains 3 |
| ZNF259 | ENST00000227322.3 | zinc finger protein 259 |
| METTL14 | ENST00000388822.5 | methyltransferase like 14 |
| MAGI1 | ENST00000330909.8 | membrane associated guanylate kinase, WW and PDZ domain containing 1 |
| ZNF347 | ENST00000334197.7 | zinc finger protein 347 |
| C6orf62 | ENST00000378119.4 | chromosome 6 open reading frame 62 |
| NAV1 | ENST00000295624.6 | neuron navigator 1 |
| ATP6V1C1 | ENST00000395862.3 | ATPase, H+ transporting, lysosomal 42kDa, V1 subunit C1 |
| SLC1A5 | ENST00000542575.2 | solute carrier family 1 (neutral amino acid transporter), member 5 |
| SNRNP35 | ENST00000526639.2 | small nuclear ribonucleoprotein 35kDa (U11/U12) |
| TBL1XR1 | ENST00000430069.1 | transducin (beta)-like 1 X-linked receptor 1 |
| CHML | ENST00000366553.1 | choroideremia-like (Rab escort protein 2) |
| MRPL3 | ENST00000264995.3 | mitochondrial ribosomal protein L3 |
| AGK | ENST00000355413.4 | acylglycerol kinase |
| LIMCH1 | ENST00000313860.7 | LIM and calponin homology domains 1 |
| GUCY1A2 | ENST00000526355.2 | guanylate cyclase 1, soluble, alpha 2 |
| TMEM200C | ENST00000581347.2 | transmembrane protein 200C |
| HEXIM2 | ENST00000307275.3 | hexamethylene bis-acetamide inducible 2 |
| PTCHD1 | ENST00000379361.4 | patched domain containing 1 |
| DMD | ENST00000378677.2 | dystrophin |
| TRIM24 | ENST00000343526.4 | tripartite motif containing 24 |
| CERS4 | ENST00000559336.1 | ceramide synthase 4 |
| STK24 | ENST00000397517.2 | serine/threonine kinase 24 |
| ZNF177 | ENST00000541595.2 | Zinc finger protein 177 |
| TPGS2 | ENST00000334295.4 | tubulin polyglutamylase complex subunit 2 |
| ARHGEF40 | ENST00000298694.4 | Rho guanine nucleotide exchange factor (GEF) 40 |
| PRRC2B | ENST00000372249.1 | proline-rich coiled-coil 2B |
| SIN3A | ENST00000394947.3 | SIN3 transcription regulator family member A |
| SFRP1 | ENST00000220772.3 | secreted frizzled-related protein 1 |
| EIF5B | ENST00000289371.6 | eukaryotic translation initiation factor 5B |
| CITED2 | ENST00000367651.2 | Cbp/p300-interacting transactivator, with Glu/Asp-rich carboxy-terminal domain, 2 |
| XPOT | ENST00000332707.5 | exportin, tRNA |
| TK2 | ENST00000299697.7 | thymidine kinase 2, mitochondrial |
| C3orf62 | ENST00000343010.3 | chromosome 3 open reading frame 62 |
| PAK1IP1 | ENST00000379568.3 | PAK1 interacting protein 1 |
| LMTK2 | ENST00000297293.5 | lemur tyrosine kinase 2 |
| TMEM167A | ENST00000502346.1 | transmembrane protein 167A |
| ZBTB25 | ENST00000608382.1 | zinc finger and BTB domain containing 25 |
| DNASE1L2 | ENST00000564065.1 | deoxyribonuclease I-like 2 |
| AZI2 | ENST00000479665.1 | 5-azacytidine induced 2 |
| RPH3AL | ENST00000331302.7 | rabphilin 3A-like (without C2 domains) |
| IBA57 | ENST00000366711.3 | IBA57, iron-sulfur cluster assembly homolog (S. cerevisiae) |
| FBXW2 | ENST00000608872.1 | F-box and WD repeat domain containing 2 |
| ZNF480 | ENST00000490272.1 | zinc finger protein 480 |
| CLPB | ENST00000294053.3 | ClpB caseinolytic peptidase B homolog (E. coli) |
| HEPH | ENST00000519389.1 | hephaestin |
| ZNF670 | ENST00000366503.2 | zinc finger protein 670 |
| POLI | ENST00000579534.1 | polymerase (DNA directed) iota |
| ACTR1A | ENST00000487599.1 | ARP1 actin-related protein 1 homolog A, centractin alpha (yeast) |
| CETN3 | ENST00000283122.3 | centrin, EF-hand protein, 3 |
| COL4A3BP | ENST00000380494.5 | collagen, type IV, alpha 3 (Goodpasture antigen) binding protein |
| ABCE1 | ENST00000296577.4 | ATP-binding cassette, sub-family E (OABP), member 1 |
| HDAC2 | ENST00000519065.1 | histone deacetylase 2 |
| PDIA3 | ENST00000300289.5 | protein disulfide isomerase family A, member 3 |
| DMXL1 | ENST00000311085.8 | Dmx-like 1 |
| PERP | ENST00000421351.3 | PERP, TP53 apoptosis effector |
| YTHDC1 | ENST00000344157.4 | YTH domain containing 1 |
| GFM1 | ENST00000486715.1 | G elongation factor, mitochondrial 1 |
| SLC29A3 | ENST00000373189.5 | solute carrier family 29 (equilibrative nucleoside transporter), member 3 |
| MIA3 | ENST00000344922.5 | melanoma inhibitory activity family, member 3 |
| ATP5B | ENST00000262030.3 | ATP synthase, H+ transporting, mitochondrial F1 complex, beta polypeptide |
| TPT1 | ENST00000379056.1 | tumor protein, translationally-controlled 1 |
| TSKU | ENST00000333090.4 | tsukushi, small leucine rich proteoglycan |
| TROVE2 | ENST00000367444.3 | TROVE domain family, member 2 |
| DNAJC15 | ENST00000379221.2 | DnaJ (Hsp40) homolog, subfamily C, member 15 |
| DCAF5 | ENST00000341516.5 | DDB1 and CUL4 associated factor 5 |
| NAGS | ENST00000293404.3 | N-acetylglutamate synthase |
| LSM3 | ENST00000306024.3 | LSM3 homolog, U6 small nuclear RNA associated (S. cerevisiae) |
| RIMS3 | ENST00000372684.3 | regulating synaptic membrane exocytosis 3 |
| TMEM136 | ENST00000529187.1 | transmembrane protein 136 |
| SPOCK2 | ENST00000373109.2 | sparc/osteonectin, cwcv and kazal-like domains proteoglycan (testican) 2 |
| SERPINA4 | ENST00000555095.1 | serpin peptidase inhibitor, clade A (alpha-1 antiproteinase, antitrypsin), member 4 |
| TPM3 | ENST00000368531.2 | tropomyosin 3 |
| SKP1 | ENST00000353411.6 | S-phase kinase-associated protein 1 |
| CYB5R4 | ENST00000369681.5 | cytochrome b5 reductase 4 |
| NABP1 | ENST00000410026.2 | nucleic acid binding protein 1 |
| VSNL1 | ENST00000406397.1 | visinin-like 1 |
| ZNF737 | ENST00000427401.4 | zinc finger protein 737 |
| FIBIN | ENST00000318627.2 | fin bud initiation factor homolog (zebrafish) |
| ZKSCAN3 | ENST00000377255.3 | zinc finger with KRAB and SCAN domains 3 |
| EHD4 | ENST00000220325.4 | EH-domain containing 4 |
| FAM71F2 | ENST00000480462.1 | family with sequence similarity 71, member F2 |
| ASTN2 | ENST00000341734.4 | astrotactin 2 |
| KIAA1045 | ENST00000242315.3 | KIAA1045 |
| MCOLN3 | ENST00000370589.2 | mucolipin 3 |
| CNKSR3 | ENST00000607772.1 | CNKSR family member 3 |
| GTPBP4 | ENST00000360803.4 | GTP binding protein 4 |
| ODF2L | ENST00000370566.3 | outer dense fiber of sperm tails 2-like |
| GPSM2 | ENST00000406462.2 | G-protein signaling modulator 2 |
| POLR1E | ENST00000377798.4 | polymerase (RNA) I polypeptide E, 53kDa |
| PLCG2 | ENST00000359376.3 | phospholipase C, gamma 2 (phosphatidylinositol-specific) |
| C2orf68 | ENST00000306336.5 | chromosome 2 open reading frame 68 |
| CBX8 | ENST00000269385.4 | chromobox homolog 8 |
| HSD17B12 | ENST00000278353.4 | hydroxysteroid (17-beta) dehydrogenase 12 |
| PAQR3 | ENST00000512733.1 | progestin and adipoQ receptor family member III |
| IWS1 | ENST00000295321.4 | IWS1 homolog (S. cerevisiae) |
| BNC2 | ENST00000380672.4 | basonuclin 2 |
| CCDC59 | ENST00000256151.7 | coiled-coil domain containing 59 |
| GEMIN4 | ENST00000319004.5 | gem (nuclear organelle) associated protein 4 |
| GRAMD1B | ENST00000529750.1 | GRAM domain containing 1B |
| WDFY2 | ENST00000298125.5 | WD repeat and FYVE domain containing 2 |
| C3orf17 | ENST00000314400.5 | chromosome 3 open reading frame 17 |
| DSN1 | ENST00000426836.1 | DSN1, MIS12 kinetochore complex component |
| ZNF223 | ENST00000434772.3 | zinc finger protein 223 |
| ALG12 | ENST00000330817.6 | ALG12, alpha-1,6-mannosyltransferase |
| VPS16 | ENST00000380445.3 | vacuolar protein sorting 16 homolog (S. cerevisiae) |
| FAM229B | ENST00000368656.2 | family with sequence similarity 229, member B |
| FLYWCH2 | ENST00000396958.3 | FLYWCH family member 2 |
| KIAA0101 | ENST00000558008.1 | KIAA0101 |
| PTGR2 | ENST00000555661.1 | prostaglandin reductase 2 |
| SLC25A16 | ENST00000609923.1 | solute carrier family 25 (mitochondrial carrier; Graves disease autoantigen), member 16 |
| STMN1 | ENST00000455785.2 | stathmin 1 |
| VPS37A | ENST00000324849.4 | vacuolar protein sorting 37 homolog A (S. cerevisiae) |
| MRE11A | ENST00000323929.3 | MRE11 meiotic recombination 11 homolog A (S. cerevisiae) |
| BAMBI | ENST00000375533.3 | BMP and activin membrane-bound inhibitor |
| OXA1L | ENST00000285848.5 | oxidase (cytochrome c) assembly 1-like |
| C19orf52 | ENST00000270502.6 | chromosome 19 open reading frame 52 |
| ZNF780B | ENST00000434248.1 | zinc finger protein 780B |
| UBE2B | ENST00000265339.2 | ubiquitin-conjugating enzyme E2B |
| CSTF2 | ENST00000415585.2 | cleavage stimulation factor, 3' pre-RNA, subunit 2, 64kDa |
| ZNF449 | ENST00000339249.4 | zinc finger protein 449 |
| SNAP47 | ENST00000366760.1 | synaptosomal-associated protein, 47kDa |
| ACO1 | ENST00000309951.6 | aconitase 1, soluble |
| NDUFB6 | ENST00000379847.3 | NADH dehydrogenase (ubiquinone) 1 beta subcomplex, 6, 17kDa |
| WDR92 | ENST00000295121.6 | WD repeat domain 92 |
| ZNF71 | ENST00000328070.6 | zinc finger protein 71 |
| MARCKS | ENST00000368635.4 | myristoylated alanine-rich protein kinase C substrate |
| DEPDC1 | ENST00000456315.2 | DEP domain containing 1 |
| POLDIP3 | ENST00000348657.2 | polymerase (DNA-directed), delta interacting protein 3 |
| FAM173B | ENST00000280330.8 | family with sequence similarity 173, member B |
| NEBL | ENST00000377122.4 | nebulette |
| CENPP | ENST00000375587.3 | centromere protein P |
| TIFA | ENST00000361717.3 | TRAF-interacting protein with forkhead-associated domain |
| GPRC5A | ENST00000014914.5 | G protein-coupled receptor, family C, group 5, member A |
| RRP8 | ENST00000254605.6 | ribosomal RNA processing 8, methyltransferase, homolog (yeast) |
| GDPGP1 | ENST00000558017.1 | GDP-D-glucose phosphorylase 1 |
| ASNSD1 | ENST00000260952.4 | asparagine synthetase domain containing 1 |
| SEC31B | ENST00000370345.3 | SEC31 homolog B (S. cerevisiae) |
| HPX | ENST00000265983.3 | hemopexin |
| AGPAT6 | ENST00000396987.3 | 1-acylglycerol-3-phosphate O-acyltransferase 6 |
| ZNF526 | ENST00000301215.3 | zinc finger protein 526 |
| TSPAN31 | ENST00000547992.1 | tetraspanin 31 |
| MDH2 | ENST00000315758.5 | malate dehydrogenase 2, NAD (mitochondrial) |
| ABCA5 | ENST00000392676.3 | ATP-binding cassette, sub-family A (ABC1), member 5 |
| SSX2IP | ENST00000342203.3 | synovial sarcoma, X breakpoint 2 interacting protein |
| TRDMT1 | ENST00000377799.3 | tRNA aspartic acid methyltransferase 1 |
| GABRB3 | ENST00000311550.5 | gamma-aminobutyric acid (GABA) A receptor, beta 3 |
| RAB4A | ENST00000366690.4 | RAB4A, member RAS oncogene family |
| AKT3 | ENST00000366539.1 | v-akt murine thymoma viral oncogene homolog 3 |
| CTSV | ENST00000259470.5 | cathepsin V |
| RTN3 | ENST00000339997.4 | reticulon 3 |
| ZWINT | ENST00000373944.3 | ZW10 interacting kinetochore protein |
| ATP5G3 | ENST00000284727.4 | ATP synthase, H+ transporting, mitochondrial Fo complex, subunit C3 (subunit 9) |
| SH3GLB2 | ENST00000372564.3 | SH3-domain GRB2-like endophilin B2 |
| FZD1 | ENST00000287934.2 | frizzled family receptor 1 |
| DSE | ENST00000452085.3 | dermatan sulfate epimerase |
| BDNF | ENST00000439476.2 | brain-derived neurotrophic factor |
| FMN1 | ENST00000334528.9 | formin 1 |
| MYO1E | ENST00000288235.4 | myosin IE |
| NAA38 | ENST00000249299.2 | N(alpha)-acetyltransferase 38, NatC auxiliary subunit |
| NSL1 | ENST00000422588.2 | NSL1, MIS12 kinetochore complex component |
| UROS | ENST00000368797.4 | uroporphyrinogen III synthase |
| RPL14 | ENST00000416518.1 | ribosomal protein L14 |
| SLC35C2 | ENST00000372227.1 | solute carrier family 35 (GDP-fucose transporter), member C2 |
| TNFRSF13C | ENST00000291232.3 | tumor necrosis factor receptor superfamily, member 13C |
| WDR72 | ENST00000396328.1 | WD repeat domain 72 |
| NSUN3 | ENST00000314622.4 | NOP2/Sun domain family, member 3 |
| MTDH | ENST00000336273.3 | metadherin |
| KB-1507C5.2 | ENST00000524007.1 | HCG15011, isoform CRA_a; Protein LOC100996457 |
| ACAD8 | ENST00000281182.4 | acyl-CoA dehydrogenase family, member 8 |
| NUF2 | ENST00000367900.3 | NUF2, NDC80 kinetochore complex component |
| PDGFC | ENST00000502773.1 | platelet derived growth factor C |
| GNL1 | ENST00000376621.3 | guanine nucleotide binding protein-like 1 |
| PAPD5 | ENST00000357464.3 | PAP associated domain containing 5 |
| TACO1 | ENST00000258975.6 | translational activator of mitochondrially encoded cytochrome c oxidase I |
| IGSF10 | ENST00000282466.3 | immunoglobulin superfamily, member 10 |
| WDR73 | ENST00000434634.2 | WD repeat domain 73 |
| LNX1 | ENST00000306888.2 | ligand of numb-protein X 1, E3 ubiquitin protein ligase |
| CLMN | ENST00000298912.4 | calmin (calponin-like, transmembrane) |
| PAPD4 | ENST00000453514.1 | PAP associated domain containing 4 |
| RSBN1L | ENST00000334955.8 | round spermatid basic protein 1-like |
| PCF11 | ENST00000298281.4 | PCF11 cleavage and polyadenylation factor subunit |
| C9orf156 | ENST00000375119.3 | chromosome 9 open reading frame 156 |
| SRSF6 | ENST00000244020.3 | serine/arginine-rich splicing factor 6 |
| ATP5F1 | ENST00000369722.3 | ATP synthase, H+ transporting, mitochondrial Fo complex, subunit B1 |
| NDNF | ENST00000379692.4 | neuron-derived neurotrophic factor |
| TBC1D4 | ENST00000377636.3 | TBC1 domain family, member 4 |
| IFT74 | ENST00000380062.5 | intraflagellar transport 74 homolog (Chlamydomonas) |
| LPHN2 | ENST00000370715.1 | latrophilin 2 |
| KCNK1 | ENST00000366621.3 | potassium channel, subfamily K, member 1 |
| PPIL2 | ENST00000406385.1 | peptidylprolyl isomerase (cyclophilin)-like 2 |
| TF | ENST00000402696.3 | transferrin |
| ZBTB14 | ENST00000357006.4 | zinc finger and BTB domain containing 14 |
| RNF126 | ENST00000292363.5 | ring finger protein 126 |
| EIF2B1 | ENST00000424014.2 | eukaryotic translation initiation factor 2B, subunit 1 alpha, 26kDa |
| SP3 | ENST00000310015.6 | Sp3 transcription factor |
| IL17RA | ENST00000319363.6 | interleukin 17 receptor A |
| ERP44 | ENST00000262455.6 | endoplasmic reticulum protein 44 |
| SEC14L2 | ENST00000312932.9 | SEC14-like 2 (S. cerevisiae) |
| FANCB | ENST00000324138.3 | Fanconi anemia, complementation group B |
| FAM114A2 | ENST00000351797.4 | family with sequence similarity 114, member A2 |
| PCBP2 | ENST00000455667.3 | poly(rC) binding protein 2 |
| SLC38A7 | ENST00000570101.1 | solute carrier family 38, member 7 |
| PSD4 | ENST00000441564.3 | pleckstrin and Sec7 domain containing 4 |
| ZSCAN29 | ENST00000562072.1 | zinc finger and SCAN domain containing 29 |
| TBC1D16 | ENST00000310924.2 | TBC1 domain family, member 16 |
| NDUFA6 | ENST00000602404.1 | NADH dehydrogenase (ubiquinone) 1 alpha subcomplex, 6, 14kDa |
| SLC30A5 | ENST00000396591.3 | solute carrier family 30 (zinc transporter), member 5 |
| FTO | ENST00000471389.1 | fat mass and obesity associated |
| GTPBP8 | ENST00000383677.3 | GTP-binding protein 8 (putative) |
| NOL6 | ENST00000455041.2 | nucleolar protein 6 (RNA-associated) |
| SCN9A | ENST00000409672.1 | sodium channel, voltage-gated, type IX, alpha subunit |
| CNNM2 | ENST00000369878.4 | cyclin M2 |
| SETD9 | ENST00000285947.2 | SET domain containing 9 |
| USP31 | ENST00000219689.7 | ubiquitin specific peptidase 31 |
| COBLL1 | ENST00000375458.2 | cordon-bleu WH2 repeat protein-like 1 |
| WEE1 | ENST00000299613.6 | WEE1 homolog (S. pombe) |
| PHF8 | ENST00000338946.6 | PHD finger protein 8 |
| INTS8 | ENST00000523731.1 | integrator complex subunit 8 |
| ESCO1 | ENST00000269214.5 | establishment of sister chromatid cohesion N-acetyltransferase 1 |
| AFF3 | ENST00000409236.2 | AF4/FMR2 family, member 3 |
| OCIAD2 | ENST00000273860.4 | OCIA domain containing 2 |
| DIP2A | ENST00000318711.7 | DIP2 disco-interacting protein 2 homolog A (Drosophila) |
| DDRGK1 | ENST00000354488.3 | DDRGK domain containing 1 |
| TIGD2 | ENST00000317005.2 | tigger transposable element derived 2 |
| EXOC3 | ENST00000512944.1 | exocyst complex component 3 |
| TTC38 | ENST00000381031.3 | tetratricopeptide repeat domain 38 |
| SUPT3H | ENST00000371460.1 | suppressor of Ty 3 homolog (S. cerevisiae) |
| MAPK14 | ENST00000229795.3 | mitogen-activated protein kinase 14 |
| C18orf42 | ENST00000580650.1 | chromosome 18 open reading frame 42 |
| GLG1 | ENST00000422840.2 | golgi glycoprotein 1 |
| SLC16A1 | ENST00000369626.3 | solute carrier family 16 (monocarboxylate transporter), member 1 |
| EIF2AK1 | ENST00000199389.6 | eukaryotic translation initiation factor 2-alpha kinase 1 |
| ZNF613 | ENST00000293471.6 | zinc finger protein 613 |
| TSPAN14 | ENST00000429989.3 | tetraspanin 14 |
| TTF2 | ENST00000369466.4 | transcription termination factor, RNA polymerase II |
| ULK2 | ENST00000395544.4 | unc-51 like autophagy activating kinase 2 |
| MSRB2 | ENST00000376510.3 | methionine sulfoxide reductase B2 |
| VPS18 | ENST00000220509.5 | vacuolar protein sorting 18 homolog (S. cerevisiae) |
| RIC8B | ENST00000392837.4 | RIC8 guanine nucleotide exchange factor B |
| NCAPH2 | ENST00000420993.2 | non-SMC condensin II complex, subunit H2 |
| DNAJC10 | ENST00000264065.7 | DnaJ (Hsp40) homolog, subfamily C, member 10 |
| WDR37 | ENST00000358220.1 | WD repeat domain 37 |
| PET112 | ENST00000263985.6 | PET112 homolog (yeast) |
| STX7 | ENST00000367941.2 | syntaxin 7 |
| CKAP2 | ENST00000378037.5 | cytoskeleton associated protein 2 |
| ZC3H15 | ENST00000337859.6 | zinc finger CCCH-type containing 15 |
| FEM1C | ENST00000274457.3 | fem-1 homolog c (C. elegans) |
| CHIC2 | ENST00000263921.3 | cysteine-rich hydrophobic domain 2 |
| SPATS2 | ENST00000553127.1 | spermatogenesis associated, serine-rich 2 |
| DLG1 | ENST00000346964.2 | discs, large homolog 1 (Drosophila) |
| NGDN | ENST00000397154.3 | neuroguidin, EIF4E binding protein |
| CTD-2228K2.5 | ENST00000342584.3 | Uncharacterized protein |
| C12orf5 | ENST00000179259.4 | chromosome 12 open reading frame 5 |
| PLEKHO1 | ENST00000369124.4 | pleckstrin homology domain containing, family O member 1 |
| C15orf61 | ENST00000342683.4 | chromosome 15 open reading frame 61 |
| SCO1 | ENST00000255390.5 | SCO1 cytochrome c oxidase assembly protein |
| TPRG1L | ENST00000378344.2 | tumor protein p63 regulated 1-like |
| ACSS2 | ENST00000336325.4 | acyl-CoA synthetase short-chain family member 2 |
| SNX2 | ENST00000379516.2 | sorting nexin 2 |
| ATP6V1E1 | ENST00000253413.5 | ATPase, H+ transporting, lysosomal 31kDa, V1 subunit E1 |
| ZNF556 | ENST00000586426.1 | zinc finger protein 556 |
| PGLS | ENST00000252603.2 | 6-phosphogluconolactonase |
| STK25 | ENST00000316586.4 | serine/threonine kinase 25 |
| IGSF3 | ENST00000369486.3 | immunoglobulin superfamily, member 3 |
| PRSS23 | ENST00000280258.5 | protease, serine, 23 |
| GDAP2 | ENST00000369443.5 | ganglioside induced differentiation associated protein 2 |
| ST5 | ENST00000526757.1 | suppression of tumorigenicity 5 |
| G6PC | ENST00000253801.2 | glucose-6-phosphatase, catalytic subunit |
| NOLC1 | ENST00000405356.1 | nucleolar and coiled-body phosphoprotein 1 |
| SLC22A23 | ENST00000436008.2 | solute carrier family 22, member 23 |
| HTT | ENST00000355072.5 | huntingtin |
| ECHDC3 | ENST00000379215.4 | enoyl CoA hydratase domain containing 3 |
| CLEC4C | ENST00000542353.1 | C-type lectin domain family 4, member C |
| ASCC1 | ENST00000342444.4 | activating signal cointegrator 1 complex subunit 1 |
| ASH2L | ENST00000343823.6 | ash2 (absent, small, or homeotic)-like (Drosophila) |
| MKKS | ENST00000347364.3 | McKusick-Kaufman syndrome |
| TMEM233 | ENST00000426426.1 | transmembrane protein 233 |
| ALG14 | ENST00000370205.5 | ALG14, UDP-N-acetylglucosaminyltransferase subunit |
| PELP1 | ENST00000574876.1 | proline, glutamate and leucine rich protein 1 |
| CYP11B1 | ENST00000292427.4 | cytochrome P450, family 11, subfamily B, polypeptide 1 |
| EXT1 | ENST00000378204.2 | exostosin glycosyltransferase 1 |
| AQR | ENST00000156471.5 | aquarius intron-binding spliceosomal factor |
| SHOC2 | ENST00000369452.4 | soc-2 suppressor of clear homolog (C. elegans) |
| ARGLU1 | ENST00000400198.3 | arginine and glutamate rich 1 |
| SLC2A12 | ENST00000275230.5 | solute carrier family 2 (facilitated glucose transporter), member 12 |
| ZNF749 | ENST00000334181.4 | zinc finger protein 749 |
| ZNF607 | ENST00000355202.4 | zinc finger protein 607 |
| GALNT2 | ENST00000366672.4 | UDP-N-acetyl-alpha-D-galactosamine:polypeptide N-acetylgalactosaminyltransferase 2 (GalNAc-T2) |
| DCUN1D5 | ENST00000260247.5 | DCN1, defective in cullin neddylation 1, domain containing 5 |
| TRPM7 | ENST00000560955.1 | transient receptor potential cation channel, subfamily M, member 7 |
| ETV5 | ENST00000306376.5 | ets variant 5 |
| PIGP | ENST00000360525.4 | phosphatidylinositol glycan anchor biosynthesis, class P |
| AGPS | ENST00000264167.4 | alkylglycerone phosphate synthase |
| GSKIP | ENST00000555181.1 | GSK3B interacting protein |
| STK16 | ENST00000409638.3 | serine/threonine kinase 16 |
| GPR78 | ENST00000382487.4 | G protein-coupled receptor 78 |
| RFFL | ENST00000315249.7 | ring finger and FYVE-like domain containing E3 ubiquitin protein ligase |
| CDR1 | ENST00000370532.2 | cerebellar degeneration-related protein 1, 34kDa |
| POLDIP2 | ENST00000540200.1 | polymerase (DNA-directed), delta interacting protein 2 |
| CCDC6 | ENST00000263102.6 | coiled-coil domain containing 6 |
| ISL2 | ENST00000290759.4 | ISL LIM homeobox 2 |
| TUBD1 | ENST00000346141.6 | tubulin, delta 1 |
| TSC22D2 | ENST00000361875.3 | TSC22 domain family, member 2 |
| OR2A4 | ENST00000315453.2 | olfactory receptor, family 2, subfamily A, member 4 |
| ZNF574 | ENST00000600245.1 | zinc finger protein 574 |
| FBLN1 | ENST00000327858.6 | fibulin 1 |
| BCL2L15 | ENST00000393316.3 | BCL2-like 15 |
| FOS | ENST00000303562.4 | FBJ murine osteosarcoma viral oncogene homolog |
| IFFO1 | ENST00000436152.2 | intermediate filament family orphan 1 |
| EMILIN2 | ENST00000254528.3 | elastin microfibril interfacer 2 |
| CCT5 | ENST00000280326.4 | chaperonin containing TCP1, subunit 5 (epsilon) |
| CKAP5 | ENST00000529230.1 | cytoskeleton associated protein 5 |
| MRTO4 | ENST00000330263.4 | mRNA turnover 4 homolog (S. cerevisiae) |
| GRSF1 | ENST00000254799.6 | G-rich RNA sequence binding factor 1 |
| AC002472.13 | ENST00000543388.1 | Leucine-rich repeat-containing protein LOC400891 |
| BID | ENST00000317361.7 | BH3 interacting domain death agonist |
| ZNF90 | ENST00000418063.2 | zinc finger protein 90 |
| BRWD3 | ENST00000373275.4 | bromodomain and WD repeat domain containing 3 |
| MCTS1 | ENST00000371317.5 | malignant T cell amplified sequence 1 |
| CORO7 | ENST00000251166.4 | coronin 7 |
| POC1A | ENST00000296484.2 | POC1 centriolar protein A |
| MCF2L2 | ENST00000328913.3 | MCF.2 cell line derived transforming sequence-like 2 |
| NUPL2 | ENST00000258742.5 | nucleoporin like 2 |
| MAL | ENST00000309988.4 | mal, T-cell differentiation protein |
| GPC4 | ENST00000370828.3 | glypican 4 |
| SSBP2 | ENST00000320672.4 | single-stranded DNA binding protein 2 |
| FOXA2 | ENST00000419308.2 | forkhead box A2 |
| PHB2 | ENST00000546111.1 | prohibitin 2 |
| VGLL2 | ENST00000352536.3 | vestigial like 2 (Drosophila) |
| ALG13 | ENST00000371979.3 | ALG13, UDP-N-acetylglucosaminyltransferase subunit |
| LRRN4 | ENST00000378858.4 | leucine rich repeat neuronal 4 |
| ANKH | ENST00000284268.6 | ANKH inorganic pyrophosphate transport regulator |
| DEFB105B | ENST00000335510.6 | defensin, beta 105B |
| RAD51L3-RFFL | ENST00000593039.1 | Uncharacterized protein |
| IL17RB | ENST00000288167.3 | interleukin 17 receptor B |
| LSM10 | ENST00000315732.2 | LSM10, U7 small nuclear RNA associated |
| DEFB105A | ENST00000334773.6 | defensin, beta 105A |
| ACP6 | ENST00000369238.6 | acid phosphatase 6, lysophosphatidic |
| GTF2H5 | ENST00000607778.1 | general transcription factor IIH, polypeptide 5 |
| TMEM86B | ENST00000327042.4 | transmembrane protein 86B |
| ATXN7L2 | ENST00000369870.3 | ataxin 7-like 2 |
| DDHD2 | ENST00000397166.2 | DDHD domain containing 2 |
| GSG1 | ENST00000396302.3 | germ cell associated 1 |
| LMOD2 | ENST00000458573.2 | leiomodin 2 (cardiac) |
| MBD2 | ENST00000256429.3 | methyl-CpG binding domain protein 2 |
| TNFSF8 | ENST00000223795.2 | tumor necrosis factor (ligand) superfamily, member 8 |
| CLCC1 | ENST00000356970.2 | chloride channel CLIC-like 1 |
| BTN3A2 | ENST00000396948.1 | butyrophilin, subfamily 3, member A2 |
| TAB1 | ENST00000216160.6 | TGF-beta activated kinase 1/MAP3K7 binding protein 1 |
| SYNCRIP | ENST00000355238.6 | synaptotagmin binding, cytoplasmic RNA interacting protein |
| RETSAT | ENST00000295802.4 | retinol saturase (all-trans-retinol 13,14-reductase) |
| SENP1 | ENST00000004980.5 | SUMO1/sentrin specific peptidase 1 |
| C2orf72 | ENST00000373640.4 | chromosome 2 open reading frame 72 |
| SPANXN1 | ENST00000370493.3 | SPANX family, member N1 |
| PSIP1 | ENST00000380738.4 | PC4 and SFRS1 interacting protein 1 |
| NUCKS1 | ENST00000367142.4 | nuclear casein kinase and cyclin-dependent kinase substrate 1 |
| TRMU | ENST00000381019.3 | tRNA 5-methylaminomethyl-2-thiouridylate methyltransferase |
| ORC6 | ENST00000568364.2 | origin recognition complex, subunit 6 |
| C8A | ENST00000361249.3 | complement component 8, alpha polypeptide |
| RASSF4 | ENST00000374417.2 | Ras association (RalGDS/AF-6) domain family member 4 |
| MS4A2 | ENST00000278888.3 | membrane-spanning 4-domains, subfamily A, member 2 |
| VWA2 | ENST00000392982.3 | von Willebrand factor A domain containing 2 |
| AKR1D1 | ENST00000432161.1 | aldo-keto reductase family 1, member D1 |
| WNT1 | ENST00000293549.3 | wingless-type MMTV integration site family, member 1 |
| SNTG1 | ENST00000522124.1 | syntrophin, gamma 1 |
| UGT2B4 | ENST00000305107.6 | UDP glucuronosyltransferase 2 family, polypeptide B4 |
| EHD2 | ENST00000263277.3 | EH-domain containing 2 |
| GRIN2A | ENST00000562109.1 | glutamate receptor, ionotropic, N-methyl D-aspartate 2A |
| GPR137B | ENST00000366592.3 | G protein-coupled receptor 137B |
| HOXB2 | ENST00000330070.4 | homeobox B2 |
| GRWD1 | ENST00000253237.5 | glutamate-rich WD repeat containing 1 |
| SLFN12L | ENST00000260908.7 | schlafen family member 12-like |
| C6orf25 | ENST00000375810.4 | chromosome 6 open reading frame 25 |
| ABI2 | ENST00000295851.5 | abl-interactor 2 |
| SLC35F6 | ENST00000344420.5 | solute carrier family 35, member F6 |
| PFKM | ENST00000312352.7 | phosphofructokinase, muscle |
| METTL24 | ENST00000338882.4 | methyltransferase like 24 |
| SERPING1 | ENST00000278407.4 | serpin peptidase inhibitor, clade G (C1 inhibitor), member 1 |
| COLEC10 | ENST00000332843.2 | collectin sub-family member 10 (C-type lectin) |
| CLU | ENST00000316403.10 | clusterin |
| FCN2 | ENST00000350339.2 | ficolin (collagen/fibrinogen domain containing lectin) 2 |
| AKR7A2 | ENST00000235835.3 | aldo-keto reductase family 7, member A2 (aflatoxin aldehyde reductase) |
| ABCB11 | ENST00000263817.6 | ATP-binding cassette, sub-family B (MDR/TAP), member 11 |
| AQP6 | ENST00000315520.5 | aquaporin 6, kidney specific |
| SF3B14 | ENST00000233468.4 | Pre-mRNA branch site protein p14 |
| WTIP | ENST00000590071.2 | Wilms tumor 1 interacting protein |
| ORMDL2 | ENST00000243045.5 | ORM1-like 2 (S. cerevisiae) |
| FHL2 | ENST00000409177.1 | four and a half LIM domains 2 |
| MGAT2 | ENST00000305386.2 | mannosyl (alpha-1,6-)-glycoprotein beta-1,2-N-acetylglucosaminyltransferase |
| KLF8 | ENST00000468660.1 | Kruppel-like factor 8 |
| C17orf72 | ENST00000580752.1 | chromosome 17 open reading frame 72 |
| GPX5 | ENST00000469384.1 | glutathione peroxidase 5 (epididymal androgen-related protein) |
| AKR1C4 | ENST00000380448.1 | aldo-keto reductase family 1, member C4 |
| DIRC2 | ENST00000261038.5 | disrupted in renal carcinoma 2 |
| CAPZB | ENST00000375142.1 | capping protein (actin filament) muscle Z-line, beta |
| LRRC6 | ENST00000519595.1 | leucine rich repeat containing 6 |
| BEST3 | ENST00000488961.1 | bestrophin 3 |
| LRRC37A3 | ENST00000339474.5 | leucine rich repeat containing 37, member A3 |
| CDH18 | ENST00000382275.1 | cadherin 18, type 2 |
| EFTUD2 | ENST00000426333.2 | elongation factor Tu GTP binding domain containing 2 |
| XPNPEP2 | ENST00000371106.3 | X-prolyl aminopeptidase (aminopeptidase P) 2, membrane-bound |
| ADAD1 | ENST00000388724.2 | adenosine deaminase domain containing 1 (testis-specific) |
| IGSF6 | ENST00000268389.4 | immunoglobulin superfamily, member 6 |
| FPR1 | ENST00000595042.1 | formyl peptide receptor 1 |
| GOLGA6B | ENST00000421285.3 | golgin A6 family, member B |
| NDUFA10 | ENST00000252711.2 | NADH dehydrogenase (ubiquinone) 1 alpha subcomplex, 10, 42kDa |
| SHMT1 | ENST00000316694.3 | serine hydroxymethyltransferase 1 (soluble) |
| SLC25A12 | ENST00000422440.2 | solute carrier family 25 (aspartate/glutamate carrier), member 12 |
| CACNG2 | ENST00000300105.6 | calcium channel, voltage-dependent, gamma subunit 2 |
| COX6B1 | ENST00000246554.3 | cytochrome c oxidase subunit VIb polypeptide 1 (ubiquitous) |
| TRAT1 | ENST00000295756.6 | T cell receptor associated transmembrane adaptor 1 |
| WDR7 | ENST00000254442.3 | WD repeat domain 7 |
| RBCK1 | ENST00000356286.5 | RanBP-type and C3HC4-type zinc finger containing 1 |
| ZNF703 | ENST00000331569.4 | zinc finger protein 703 |
| IL1RAPL1 | ENST00000378993.1 | interleukin 1 receptor accessory protein-like 1 |
| PTGDR | ENST00000553372.1 | prostaglandin D2 receptor (DP) |
| TLR8 | ENST00000218032.6 | toll-like receptor 8 |
| SMARCA5 | ENST00000283131.3 | SWI/SNF related, matrix associated, actin dependent regulator of chromatin, subfamily a, member 5 |
| RAF1 | ENST00000251849.4 | v-raf-1 murine leukemia viral oncogene homolog 1 |
| P2RY10 | ENST00000171757.2 | purinergic receptor P2Y, G-protein coupled, 10 |
| HEPHL1 | ENST00000315765.9 | hephaestin-like 1 |
| TRIOBP | ENST00000406386.3 | TRIO and F-actin binding protein |
| CRISP1 | ENST00000335847.4 | cysteine-rich secretory protein 1 |
| F7 | ENST00000375581.3 | coagulation factor VII (serum prothrombin conversion accelerator) |
| DKK3 | ENST00000396505.2 | dickkopf WNT signaling pathway inhibitor 3 |
| GALNT8 | ENST00000252318.2 | UDP-N-acetyl-alpha-D-galactosamine:polypeptide N-acetylgalactosaminyltransferase 8 (GalNAc-T8) |
| FUT10 | ENST00000327671.5 | fucosyltransferase 10 (alpha (1,3) fucosyltransferase) |
| CFHR2 | ENST00000367421.3 | complement factor H-related 2 |
| FMO3 | ENST00000367755.4 | flavin containing monooxygenase 3 |
| SMC1A | ENST00000322213.4 | structural maintenance of chromosomes 1A |
| CPEB4 | ENST00000265085.5 | cytoplasmic polyadenylation element binding protein 4 |
| CD36 | ENST00000447544.2 | CD36 molecule (thrombospondin receptor) |
| CD3D | ENST00000300692.4 | CD3d molecule, delta (CD3-TCR complex) |
| SOX12 | ENST00000342665.2 | SRY (sex determining region Y)-box 12 |
| PI4K2B | ENST00000264864.6 | phosphatidylinositol 4-kinase type 2 beta |
| ARHGAP29 | ENST00000260526.6 | Rho GTPase activating protein 29 |
| CDK12 | ENST00000447079.4 | cyclin-dependent kinase 12 |
| KANSL1L | ENST00000281772.9 | KAT8 regulatory NSL complex subunit 1-like |
| BAZ2B | ENST00000392782.1 | bromodomain adjacent to zinc finger domain, 2B |
| ATP1B4 | ENST00000218008.3 | ATPase, Na+/K+ transporting, beta 4 polypeptide |
| TLR2 | ENST00000260010.6 | toll-like receptor 2 |
| TNPO1 | ENST00000337273.5 | transportin 1 |
| TIMD4 | ENST00000274532.2 | T-cell immunoglobulin and mucin domain containing 4 |
| TCF3 | ENST00000262965.5 | transcription factor 3 |
| LUM | ENST00000266718.4 | lumican |
| KLHL36 | ENST00000564996.1 | kelch-like family member 36 |
| MRPL12 | ENST00000333676.3 | mitochondrial ribosomal protein L12 |
| LRPAP1 | ENST00000500728.2 | low density lipoprotein receptor-related protein associated protein 1 |
| CNTD1 | ENST00000588408.1 | cyclin N-terminal domain containing 1 |
| FDFT1 | ENST00000220584.4 | farnesyl-diphosphate farnesyltransferase 1 |
| CCNI2 | ENST00000378731.1 | cyclin I family, member 2 |
| FZD8 | ENST00000374694.1 | frizzled family receptor 8 |
| FIGNL1 | ENST00000356889.4 | fidgetin-like 1 |
| CCDC127 | ENST00000296824.3 | coiled-coil domain containing 127 |
| NPEPPS | ENST00000322157.4 | aminopeptidase puromycin sensitive |
| AGMO | ENST00000342526.3 | alkylglycerol monooxygenase |
| ZNF699 | ENST00000591998.1 | zinc finger protein 699 |
| AGBL5 | ENST00000323064.8 | ATP/GTP binding protein-like 5 |
| SLC16A3 | ENST00000581287.1 | solute carrier family 16 (monocarboxylate transporter), member 3 |
| CSTF2T | ENST00000331173.4 | cleavage stimulation factor, 3' pre-RNA, subunit 2, 64kDa, tau variant |
| HCN1 | ENST00000303230.4 | hyperpolarization activated cyclic nucleotide-gated potassium channel 1 |
| MMP15 | ENST00000219271.3 | matrix metallopeptidase 15 (membrane-inserted) |
| IL12RB2 | ENST00000262345.1 | interleukin 12 receptor, beta 2 |
| TRIM11 | ENST00000493030.2 | tripartite motif containing 11 |
| SRPK2 | ENST00000393651.3 | SRSF protein kinase 2 |
| AGAP9 | ENST00000453919.1 | ArfGAP with GTPase domain, ankyrin repeat and PH domain 9 |
| POTED | ENST00000299443.5 | POTE ankyrin domain family, member D |
| F5 | ENST00000367797.3 | coagulation factor V (proaccelerin, labile factor) |
| GLYAT | ENST00000344743.3 | glycine-N-acyltransferase |
| PPP2R5B | ENST00000164133.2 | protein phosphatase 2, regulatory subunit B', beta |
| ATG2A | ENST00000421419.2 | autophagy related 2A |
| GLIPR1 | ENST00000266659.3 | GLI pathogenesis-related 1 |
| AVPI1 | ENST00000370626.3 | arginine vasopressin-induced 1 |
| GPR22 | ENST00000304402.4 | G protein-coupled receptor 22 |
| HIST1H4C | ENST00000377803.2 | histone cluster 1, H4c |
| NSMCE1 | ENST00000361439.4 | non-SMC element 1 homolog (S. cerevisiae) |
| MAN2B2 | ENST00000285599.3 | mannosidase, alpha, class 2B, member 2 |
| RP13-996F3.4 | ENST00000557886.1 | Uncharacterized protein |
| ABCA6 | ENST00000284425.2 | ATP-binding cassette, sub-family A (ABC1), member 6 |
| INSIG1 | ENST00000342407.5 | insulin induced gene 1 |
| OSBPL9 | ENST00000371710.3 | oxysterol binding protein-like 9 |
| G3BP2 | ENST00000395719.3 | GTPase activating protein (SH3 domain) binding protein 2 |
| AC106017.1 | ENST00000436914.1 | Uncharacterized protein |
| NLN | ENST00000380985.5 | neurolysin (metallopeptidase M3 family) |
| TXK | ENST00000264316.4 | TXK tyrosine kinase |
| GABRB1 | ENST00000295454.3 | gamma-aminobutyric acid (GABA) A receptor, beta 1 |
| MAT1A | ENST00000372213.3 | methionine adenosyltransferase I, alpha |
| NUP88 | ENST00000573584.1 | nucleoporin 88kDa |
| SP140L | ENST00000415673.2 | SP140 nuclear body protein-like |
| SH3TC2 | ENST00000502274.1 | SH3 domain and tetratricopeptide repeats 2 |
| MYBPC1 | ENST00000392934.3 | myosin binding protein C, slow type |
| TSHR | ENST00000541158.2 | thyroid stimulating hormone receptor |
| RANBP3L | ENST00000296604.3 | RAN binding protein 3-like |
| SND1 | ENST00000354725.3 | staphylococcal nuclease and tudor domain containing 1 |
| ATIC | ENST00000236959.9 | 5-aminoimidazole-4-carboxamide ribonucleotide formyltransferase/IMP cyclohydrolase |
| APOL3 | ENST00000397293.2 | apolipoprotein L, 3 |
| CADM1 | ENST00000452722.3 | cell adhesion molecule 1 |
| PTGER3 | ENST00000370924.4 | prostaglandin E receptor 3 (subtype EP3) |
| LGR4 | ENST00000379214.4 | leucine-rich repeat containing G protein-coupled receptor 4 |
| RP11-210M15.2 | ENST00000559008.1 | Uncharacterized protein |
| TOR1AIP1 | ENST00000606911.2 | torsin A interacting protein 1 |
| CHRNA5 | ENST00000299565.5 | cholinergic receptor, nicotinic, alpha 5 (neuronal) |
| SULT1C2 | ENST00000251481.6 | sulfotransferase family, cytosolic, 1C, member 2 |
| TMEM70 | ENST00000517439.1 | transmembrane protein 70 |
| ZBTB20 | ENST00000462705.1 | zinc finger and BTB domain containing 20 |
| GPR155 | ENST00000392552.2 | G protein-coupled receptor 155 |
| RBM25 | ENST00000261973.7 | RNA binding motif protein 25 |
| KLHL30 | ENST00000409223.1 | kelch-like family member 30 |
| CFC1 | ENST00000259216.4 | cripto, FRL-1, cryptic family 1 |
| KLHDC9 | ENST00000392192.2 | kelch domain containing 9 |
| CPD | ENST00000225719.4 | carboxypeptidase D |
| OMD | ENST00000375550.4 | osteomodulin |
| NLRP9 | ENST00000332836.2 | NLR family, pyrin domain containing 9 |
| FAM216B | ENST00000537894.1 | family with sequence similarity 216, member B |
| C5orf55 | ENST00000408966.2 | chromosome 5 open reading frame 55 |
| CIRH1A | ENST00000314423.7 | cirrhosis, autosomal recessive 1A (cirhin) |
| PLCL1 | ENST00000428675.1 | phospholipase C-like 1 |
| SERPINC1 | ENST00000367698.3 | serpin peptidase inhibitor, clade C (antithrombin), member 1 |
| ACSM2A | ENST00000573854.1 | acyl-CoA synthetase medium-chain family member 2A |
| PRKACB | ENST00000370689.2 | protein kinase, cAMP-dependent, catalytic, beta |
| ELF4 | ENST00000335997.7 | E74-like factor 4 (ets domain transcription factor) |
| SOBP | ENST00000317357.5 | sine oculis binding protein homolog (Drosophila) |
| GOLM1 | ENST00000388712.3 | golgi membrane protein 1 |
| MYC | ENST00000377970.2 | v-myc avian myelocytomatosis viral oncogene homolog |
| PARP2 | ENST00000527915.1 | poly (ADP-ribose) polymerase 2 |
| PCDHB5 | ENST00000231134.5 | protocadherin beta 5 |
| UBE2D3 | ENST00000453744.2 | ubiquitin-conjugating enzyme E2D 3 |
| TRIM44 | ENST00000299413.5 | tripartite motif containing 44 |
| PCDHB15 | ENST00000231173.3 | protocadherin beta 15 |
| SMAP1 | ENST00000370452.3 | small ArfGAP 1 |
| ABCF2 | ENST00000287844.2 | ATP-binding cassette, sub-family F (GCN20), member 2 |
| CYP2C19 | ENST00000371321.3 | cytochrome P450, family 2, subfamily C, polypeptide 19 |
| SLC24A4 | ENST00000393265.2 | solute carrier family 24 (sodium/potassium/calcium exchanger), member 4 |
| EPHX2 | ENST00000521400.1 | epoxide hydrolase 2, cytoplasmic |
| ALDOA | ENST00000569798.1 | aldolase A, fructose-bisphosphate |
| NETO1 | ENST00000327305.6 | neuropilin (NRP) and tolloid (TLL)-like 1 |
| BST1 | ENST00000382346.3 | bone marrow stromal cell antigen 1 |
| ACSM2B | ENST00000329697.6 | acyl-CoA synthetase medium-chain family member 2B |
| ZNF141 | ENST00000240499.7 | zinc finger protein 141 |
| SLC22A15 | ENST00000369503.4 | solute carrier family 22, member 15 |
| RNF187 | ENST00000305943.7 | ring finger protein 187 |
| PDCL | ENST00000259467.4 | phosducin-like |
| CCNO | ENST00000282572.4 | cyclin O |
| LUC7L3 | ENST00000505658.1 | LUC7-like 3 (S. cerevisiae) |
| RSPO3 | ENST00000356698.4 | R-spondin 3 |
| RAB43 | ENST00000476465.1 | RAB43, member RAS oncogene family |
| DNAJB4 | ENST00000370763.5 | DnaJ (Hsp40) homolog, subfamily B, member 4 |
| SEMA4F | ENST00000357877.2 | sema domain, immunoglobulin domain (Ig), transmembrane domain (TM) and short cytoplasmic domain, (semaphorin) 4F |
| LRRC34 | ENST00000446859.1 | leucine rich repeat containing 34 |
| ING1 | ENST00000333219.7 | inhibitor of growth family, member 1 |
| ZNF426 | ENST00000253115.2 | zinc finger protein 426 |
| ZNF562 | ENST00000293648.4 | zinc finger protein 562 |
| NOTCH2 | ENST00000256646.2 | notch 2 |
| TMEM59 | ENST00000371348.1 | transmembrane protein 59 |
| LRRFIP1 | ENST00000308482.9 | leucine rich repeat (in FLII) interacting protein 1 |
| PKHD1L1 | ENST00000378402.5 | polycystic kidney and hepatic disease 1 (autosomal recessive)-like 1 |
| PRMT6 | ENST00000370078.1 | protein arginine methyltransferase 6 |
| BET1 | ENST00000222547.3 | Bet1 golgi vesicular membrane trafficking protein |
| DLGAP3 | ENST00000373347.1 | discs, large (Drosophila) homolog-associated protein 3 |
| SYNC | ENST00000409190.3 | syncoilin, intermediate filament protein |
| EMC1 | ENST00000477853.1 | ER membrane protein complex subunit 1 |
| RPS24 | ENST00000435275.1 | ribosomal protein S24 |
| GPR35 | ENST00000319838.5 | G protein-coupled receptor 35 |
| SAMD15 | ENST00000216471.4 | sterile alpha motif domain containing 15 |
| ANKFY1 | ENST00000341657.4 | ankyrin repeat and FYVE domain containing 1 |
| TMEM106C | ENST00000429772.2 | transmembrane protein 106C |
| POLD4 | ENST00000312419.3 | polymerase (DNA-directed), delta 4, accessory subunit |
| PBX1 | ENST00000367897.1 | pre-B-cell leukemia homeobox 1 |
| PTCHD3 | ENST00000438700.3 | patched domain containing 3 |
| SPRY1 | ENST00000394339.2 | sprouty homolog 1, antagonist of FGF signaling (Drosophila) |
| N6AMT1 | ENST00000303775.5 | N-6 adenine-specific DNA methyltransferase 1 (putative) |
| MDFIC | ENST00000393486.1 | MyoD family inhibitor domain containing |
| MORC1 | ENST00000232603.5 | MORC family CW-type zinc finger 1 |
| TTI2 | ENST00000360742.5 | TELO2 interacting protein 2 |
| IFIT1 | ENST00000371804.3 | interferon-induced protein with tetratricopeptide repeats 1 |
| SOX17 | ENST00000297316.4 | SRY (sex determining region Y)-box 17 |
| WT1 | ENST00000379079.2 | Wilms tumor 1 |
| FECH | ENST00000262093.5 | ferrochelatase |
| MARC1 | ENST00000366910.5 | mitochondrial amidoxime reducing component 1 |
| HSD11B1 | ENST00000367028.2 | hydroxysteroid (11-beta) dehydrogenase 1 |
| CELF3 | ENST00000290585.4 | CUGBP, Elav-like family member 3 |
| HEYL | ENST00000372852.3 | hairy/enhancer-of-split related with YRPW motif-like |
| ZNF140 | ENST00000355557.2 | zinc finger protein 140 |
| CFL2 | ENST00000341223.3 | cofilin 2 (muscle) |
| BDH2 | ENST00000296424.4 | 3-hydroxybutyrate dehydrogenase, type 2 |
| IGF2R | ENST00000356956.1 | insulin-like growth factor 2 receptor |
| SRMS | ENST00000217188.1 | src-related kinase lacking C-terminal regulatory tyrosine and N-terminal myristylation sites |
| RGS4 | ENST00000531057.1 | regulator of G-protein signaling 4 |
| MCTP2 | ENST00000357742.4 | multiple C2 domains, transmembrane 2 |
| ABHD15 | ENST00000307201.4 | abhydrolase domain containing 15 |
| MED14 | ENST00000324817.1 | mediator complex subunit 14 |
| PLXDC1 | ENST00000315392.4 | plexin domain containing 1 |
| C4orf19 | ENST00000381980.4 | chromosome 4 open reading frame 19 |
| CFHR4 | ENST00000608469.1 | complement factor H-related 4 |
| C1orf210 | ENST00000523677.1 | chromosome 1 open reading frame 210 |
| TCN2 | ENST00000215838.3 | transcobalamin II |
| GPI | ENST00000415930.3 | glucose-6-phosphate isomerase |
| SOCS5 | ENST00000306503.5 | suppressor of cytokine signaling 5 |
| TAL2 | ENST00000334077.3 | T-cell acute lymphocytic leukemia 2 |
| MYOM2 | ENST00000262113.4 | myomesin 2 |
| ZC4H2 | ENST00000545618.1 | zinc finger, C4H2 domain containing |
| FAM20A | ENST00000592554.1 | family with sequence similarity 20, member A |
| NRXN3 | ENST00000281127.7 | neurexin 3 |
| CRISPLD2 | ENST00000262424.5 | cysteine-rich secretory protein LCCL domain containing 2 |
| ANGPTL3 | ENST00000371129.3 | angiopoietin-like 3 |
| C18orf32 | ENST00000579820.1 | chromosome 18 open reading frame 32 |
| LRRC40 | ENST00000370952.3 | leucine rich repeat containing 40 |
| EBF4 | ENST00000380648.4 | early B-cell factor 4 |
| TYRP1 | ENST00000388918.5 | tyrosinase-related protein 1 |
| MYO10 | ENST00000513610.1 | myosin X |
| C9orf3 | ENST00000297979.5 | chromosome 9 open reading frame 3 |
| WDR75 | ENST00000314761.4 | WD repeat domain 75 |
| CRCP | ENST00000415001.2 | CGRP receptor component |
| SERPINA3 | ENST00000393080.4 | serpin peptidase inhibitor, clade A (alpha-1 antiproteinase, antitrypsin), member 3 |
| AMOTL2 | ENST00000249883.5 | angiomotin like 2 |
| SLC29A1 | ENST00000393844.1 | solute carrier family 29 (equilibrative nucleoside transporter), member 1 |
| IRF2BP2 | ENST00000366610.3 | interferon regulatory factor 2 binding protein 2 |
| RBM23 | ENST00000555209.1 | RNA binding motif protein 23 |
| ITGB1BP1 | ENST00000360635.3 | integrin beta 1 binding protein 1 |
| SAP30 | ENST00000296504.3 | Sin3A-associated protein, 30kDa |
| KCTD2 | ENST00000322444.6 | potassium channel tetramerization domain containing 2 |
| PIAS2 | ENST00000585916.1 | protein inhibitor of activated STAT, 2 |
| ZNF277 | ENST00000361822.3 | zinc finger protein 277 |
| CDC42EP4 | ENST00000335793.3 | CDC42 effector protein (Rho GTPase binding) 4 |
| RPS4Y1 | ENST00000250784.8 | ribosomal protein S4, Y-linked 1 |
| RAE1 | ENST00000395841.2 | ribonucleic acid export 1 |
| LRRC3 | ENST00000291592.4 | leucine rich repeat containing 3 |
| NDUFC2-KCTD14 | ENST00000528251.1 | NDUFC2-KCTD14 readthrough |
| MAP1A | ENST00000382031.1 | microtubule-associated protein 1A |
| CTNNA1 | ENST00000518825.1 | catenin (cadherin-associated protein), alpha 1, 102kDa |
| KCTD14 | ENST00000353172.5 | potassium channel tetramerization domain containing 14 |
| RPL23A | ENST00000422514.2 | ribosomal protein L23a |
| PRR5L | ENST00000527487.1 | proline rich 5 like |
| FXYD6-FXYD2 | ENST00000532984.1 | FXYD6-FXYD2 readthrough |
| FXYD2 | ENST00000528014.1 | FXYD domain containing ion transport regulator 2 |
| TLK2 | ENST00000582809.1 | tousled-like kinase 2 |
| NME5 | ENST00000265191.2 | NME/NM23 family member 5 |
| AKNA | ENST00000307564.4 | AT-hook transcription factor |
| DENND6B | ENST00000413817.3 | DENN/MADD domain containing 6B |
